# Controlling the Diradical Character of Thiele Like Compounds

**DOI:** 10.1021/acs.joc.3c00482

**Published:** 2023-06-20

**Authors:** Josep M. Anglada, Jordi Poater, Ibério de P. R. Moreira, Josep Maria Bofill

**Affiliations:** † Departament de Química Biològica (IQAC-CSIC), Carrer Jordi Girona, 18, 08034 Barcelona, Spain; ‡ Departament de Química Inorgànica i Orgànica & IQTCUB, Universitat de Barcelona, Martí i Franquès 1-11, 08028 Barcelona, Spain; § ICREA, Pg. Lluís Companys 23, 08010 Barcelona, Spain; ∥ Departament de Ciència de Materials i Química Física, Secció de Química Física, Universitat de Barcelona, 08028 Barcelona, Spain; ⊥ IQTCUB, Universitat de Barcelona, Martí i Franquès, 1-11, 08028 Barcelona, Spain

## Abstract

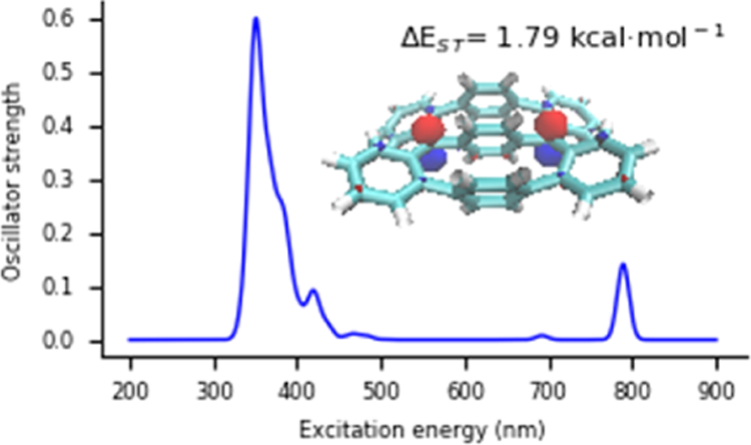

Organic diradicals play an important role in many fields of chemistry, biochemistry, and materials science. In this work, by means of high-level theoretical calculations, we have investigated the effect of representative chemical substituents in *p*-quinodimethane (*p*QDM) and Thiele’s hydrocarbons with respect to the singlet–triplet energy gap, a feature characterizing their diradical character. We show how the nature of the substituents has a very important effect in controlling the singlet–triplet energy gap so that several compounds show diradical features in their ground electronic state. Importantly, steric effects appear to play the most determinant role for *p*QDM analogues, with minor effects of the substituents in the central ring. For Thiele like compounds, we found that electron-withdrawing groups in the central ring favor the quinoidal form with a low or almost null diradical character, whereas electron-donating group substituents favor the aromatic-diradical form if the electron donation does not exceed 6-π electrons. In this case, if there is an excess of electron donation, the diradical character is reduced. The electronic spectrum of these compounds is also calculated, and we predict that the most intense bands occur in the visible region, although in some cases characteristic electronic transition in the near-IR region may appear.

## Introduction

**Figure 1:**
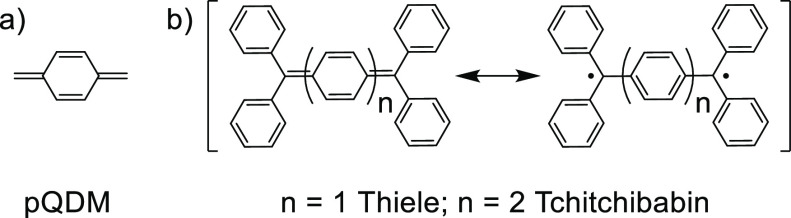
Structures of *p*QDM (a), and Thiele and Tschitschibabin compounds (b).

Organic aromatic hydrocarbons (OAHs) with the capacity of having a diradical character and exhibiting visible and/or near-infrared spectroscopy have attracted a lot of interest in the last few years because of their applicability in several areas ranging from chemistry to biochemistry and materials science. These species have potential applications in organic electronics and spintronics, molecular switches, singlet fission with solar energy conversion capability, batteries, nonlinear optics, functional dyes, and photodynamic therapy.^1−13^ An interesting family of OAHs is based on the Gomberg persistent radical, the triphenylmethyl (TPM) radical^14^ synthesized more than 100 years ago, which was the foundation of radical chemistry. Some years later, the first triarilphenyl (TAM) derivatives connecting two TPM units were synthesized, such as the Thiele,^15^ Tschitschibabin,^16^ and Müller^17^ hydrocarbons, as the first representatives of a vast family of stable diradical and polyradical OAH systems.^8,18,19^ Recently, the interest in TAM analogues as radical building blocks has increased since it provides a structural and/or synthetic basis to synthesize extended OAH molecules^20,21^ and 2D covalent organic frameworks (2D-COFs)^22,23^ with promising tuneable electronic and magnetic properties (see for instance our recent works on 2D covalent organic radical frameworks^24,25^). Despite the large number of studies of the electronic structure and properties of OAHs, the subtle balance between the quinoidal and diradical valence-bond forms shown in Figure 1 to produce a diradical ground state remains unclear. This is due to the different structural and electronic factors (i.e., electron-withdrawing or electron-donor groups (EWG/EDG), conjugation, electron correlation effects) involved even in the simpler diradical/diradicaloid hydrocarbon structures. A key issue regarding the chemical stability and applicability of diradical/diradicaloid OAHs is related to the singlet–triplet energy gap (Δ*E*_ST_), so that compounds with small Δ*E*_ST_ values (for instance, Δ*E*_ST_ < 4–6 kcal·mol^–1^) can show thermally accessible paramagnetic (or diradical) activity. For increasingly larger Δ*E*_ST_ values, the triplet state becomes thermally inaccessible and the diradical character of the singlet ground state is significantly reduced, thus gradually becoming a diradicaloid and, for very large values, a closed-shell, diamagnetic molecule. Therefore, the Δ*E*_ST_ parameter has been used to evaluate the diradical character of these compounds.^26^ A very important molecule that plays a key role in understanding the nature of the electronic structure and properties of species with possible diradical character is *para*-quinodimethane (*p*QDM) (Figure 1a), which serves as a building block for making π-extended diradical compounds, the well-known Thiele^15^ and Tschitschibabin^16^ hydrocarbons (Figure 1b) being the simplest chemically stable derivatives.

In recent years, different attempts have been carried out with the aim of controlling the relative singlet–triplet energy gap of OHAs and looking for magnetically active compounds having ground electronic states with a diradical character. These approaches consist of modifying the nature and features of the radical centers and/or extending and changing the number of rings that describe the core (main rings) of these compounds.^9,18,27−37^ However, there is still a lack of knowledge on the chemical factors that allow to tune the Δ*E*_ST_ gap of these species, and in this work, we have considered a set of model compounds derived from *p*QDM. We also focus on the Thiele’s hydrocarbon analogues due to their higher chemical stability, an essential ingredient for effective synthetic routes to produce stable extended systems. We note that π-conjugate molecules with a distinguishable quinoidal structure (as in *p*QDM) have a unique electronic structure, and these quinoidal forms typically have a pro-aromatic character, which makes them convertible into aromatic moieties, opening the dichotomic question of closed-shell quinoidal or open-shell diradical structures. Depending on the substituents and the length of the quinoidal platforms are closed-shell full quinoidal structures or open-shell diradicals.

In this work, we investigate the nature of the low-energy electronic states of several *p*QDM and Thiele’s hydrocarbon derivatives to disentangle the structural and electronic effects controlling the singlet–triplet energy gap and the electronic character of their ground electronic state. As we will show, structural effects due to bulky groups play the most determinant role when compared to the nature of the substituents (EWG/EDG/conjugated) in the bridging Ph group or as outer groups. Finally, we will explore the corresponding effects in their optical spectrum in the near-IR and visible region.

## Computational Methods

We have employed the density functional method B3LYP^38^ with the 6-31+G(d,p) basis set^39,40^ to optimize the structure of the compounds investigated in this work. For all investigated electronic states, we have employed the spin-unrestricted formalism with the broken symmetry approach to find the possible lowest energy states with a diradical character. At this level of theory, we have also performed harmonic vibrational frequency calculations to confirm the nature (minima) of the stationary points and to calculate the zero-point energy and the thermal contributions to the energy.

In order to predict more accurate relative energies, we have carried out a series of single-point energy calculations at the optimized geometry using different high-level theoretical methods. For all electronic states described by mono-referential wave functions (triplet and quinoidal singlet electronic states), we have used the DLPNO-CCSD(T) method^41^ with the cc-pVTZ basis set, which are proven to predict very accurate results for electronic states described by mono-referential methods.^42^ In these calculations, we have looked at the reliability of the results, with regard to the possible multi-reference character of the corresponding wave function, by checking the T1 diagnostic (T1d)^43^ value of the DLPNO-CCSD wave function. In all cases, the T1d values are smaller than 0.022. Although the effect of the dispersion in the relative quinoidal singlet–triplet energy gap has been taken into account in DLPNO-CCSD(T) calculations, we have further re-optimized the Thiele and Thiele like compounds including the Grimme dispersion GD3 parameters,^44^ and we have carried out DLPNO-CCSD(T) single point energy calculations at the optimized geometries. The Δ*E*_ST_ energy values obtained differ in less than 1 kcal·mol^–1^ from those obtained without considering the dispersion correction.

For most of the triplet and diradical singlet electronic states requiring the use of multi-referential methods, we have employed the fully internally contracted multireference configuration interaction (FICMR) method^45,46^ with the dev2-SVP basis set.^47^ The FICMR calculations have been done with the averaged quadratic coupled cluster (AQCC) variant^48,49^ over a complete active space self-consistent field (CASSCF(2,2)) function.^50^ The reliability of our computed energy differences has been extensively studied and discussed in the Supporting Information where we have shown that for diradical species the Δ*E*_ST_ values computed at FICMR compare very well with those obtained at an unrestricted B3LYP level. Therefore, for large systems where the FICMR calculations are not feasible we have taken the unrestricted B3LYP energy differences. The coefficients of the CASSCF wave function have been used to define the diradical character (BC) as defined in the Supporting Information. In addition, we have also studied the aromaticity of all electronic structures by means of the electronic-based multicenter index (MCI), which measures the electron sharing among the different atoms that form the ring under analysis.^51,52^ The bonding features have been also analyzed according to the atoms in molecules (AIM) theory by Bader^53−55^ and by the natural bond orbitals (NBO) by Weinhold.^56^ Finally, the electronic spectra has been computed by performing time-dependent DFT (TDDFT)^57,58^ calculations at the optimized geometries using the B3LYP approach with the 6-31+G(d,p) basis set.

All calculations carried out in his work have been done using the Gaussian 09^59^ and Orca 4.0^60^ program packages. The determination of the MCI values is computed by means of the ESI-3D software,^51,61^ and the bonding features following the AIM theory are performed with the AIMPAC program.^62^

## Results and Discussion

**Figure 2:**
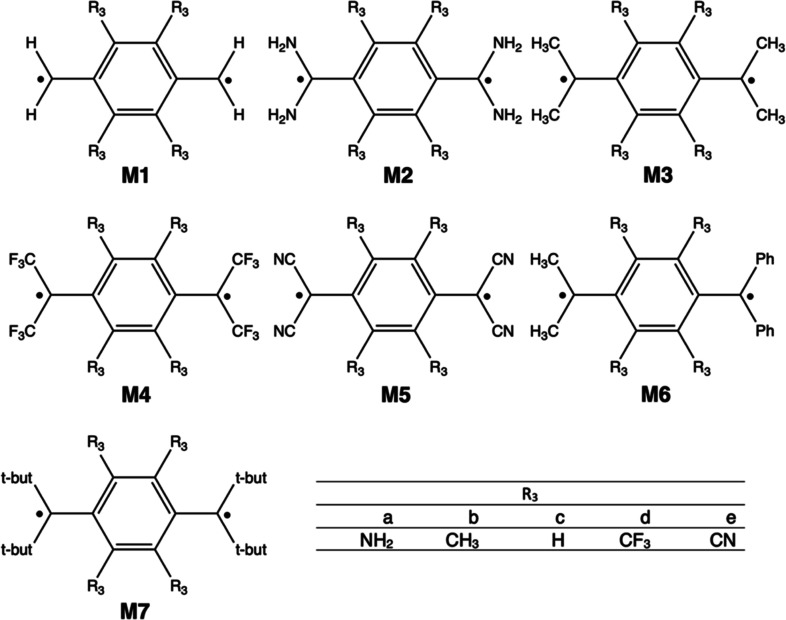
Compounds derived from *p*QDM investigated in this work.

Figure 2 encloses the set of systems derived from *p*QDM (**M1**) with the different substituents considered in this work. First, we have considered as external R_1_ and R_2_ substituents the electron-donating groups (EDG) and the electron-withdrawing groups (EWG) NH_2_ (**M2**), CH_3_ (**M3**), CF_3_ (**M4**) and CN (**M5**), CH_3_ in R_1_ and Ph in R_2_ (**M6**), and *t*-butyl (**M7**) in R_1_ and R_2_, combined with NH_2_, CH_3_, H, CF_3_, and CN substituents as R_3_, which are labeled by adding the suffixes a to e. Thus, for instance, **M2d** has R_1_ = R_2_ = NH_2_ and R_3_ = CF_3_, whereas **M4b** has R_1_ = R_2_ = CN, and R_3_ = CH_3_. Next, we have also investigated derivatives of Thiele’s hydrocarbon, with R_1_ = R_2_ = Ph, considering as R_3_ the NH_2_, CH_3_, H, F, CF_3_, and CN substituents (**T1** to **T6** in Figure 1b; here **T3** corresponds to Thiele’s hydrocarbon). Furthermore, we have considered a new compound in which two Ph substituents link the terminal Ph groups placed in opposite sides (**T7**), which allows to analyze the effects of π-stacking and a molecule resulting from the substitution of the main ring in Thiele’s hydrocarbon by anthracene (**T8**) to consider the effects of an extended π system. For these compounds, we have also calculated the electronic spectra. Our investigation has been carried out using high-level wavefunction methods with larger correlation consistent basis sets (sections S1 and S2 in the Supporting Information).

**Figure 3:**
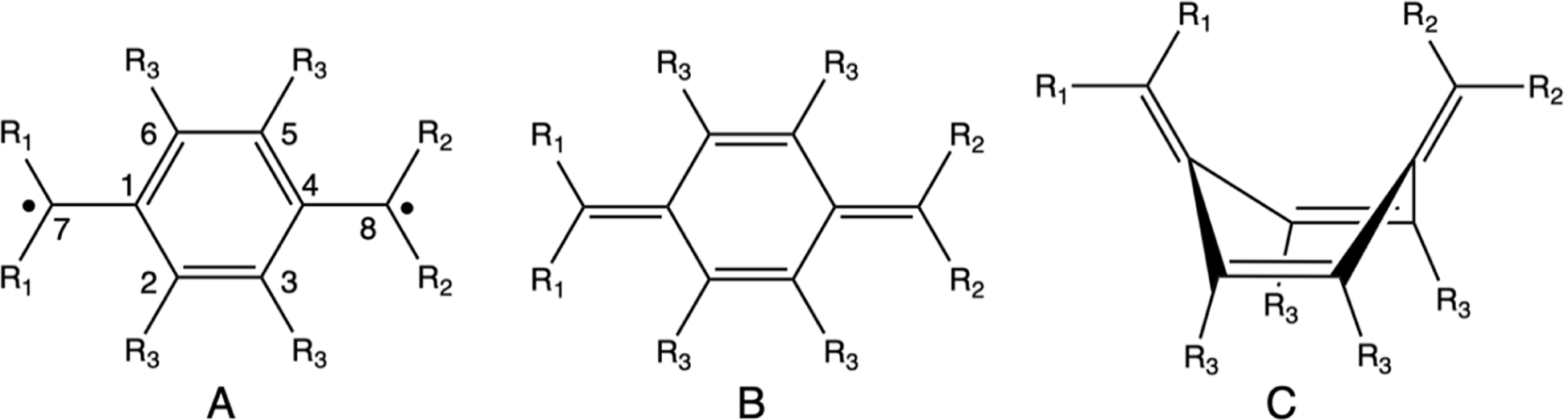
Possible conformations of the compounds investigated in this work. **A** is compatible with either diradical singlet or triplet states, whereas **B** and **C** correspond to singlet closed-shell states. Numbering of the carbon atoms is included in system **A**.

Different conformations have been explored during the structure optimizations of systems that we have faced in this work (Figure 3). Structure **A** is compatible with the triplet and singlet electronic states of diradical character, in which two unpaired electrons are mainly localized over the C_7_ and C_8_ carbon atoms, whereas structures **B** and **C** are quinoid forms and correspond to singlet closed-shell electronic states only. Notice that structure **A** shows aromatic features in the main ring, whereas no aromaticity is expected for structures **B** and **C**. Of course, some resonance may exist between structures **A** and **B** for those singlet electronic states having a diradical character and the measures of aromaticity should become an important tool in analyzing the singlet states of these compounds. Further details regarding the electronic features of structures **A**, **B**, and **C** are given in section S3 of the Supporting Information.

### Compounds **M1–M7**

**Figure 4:**
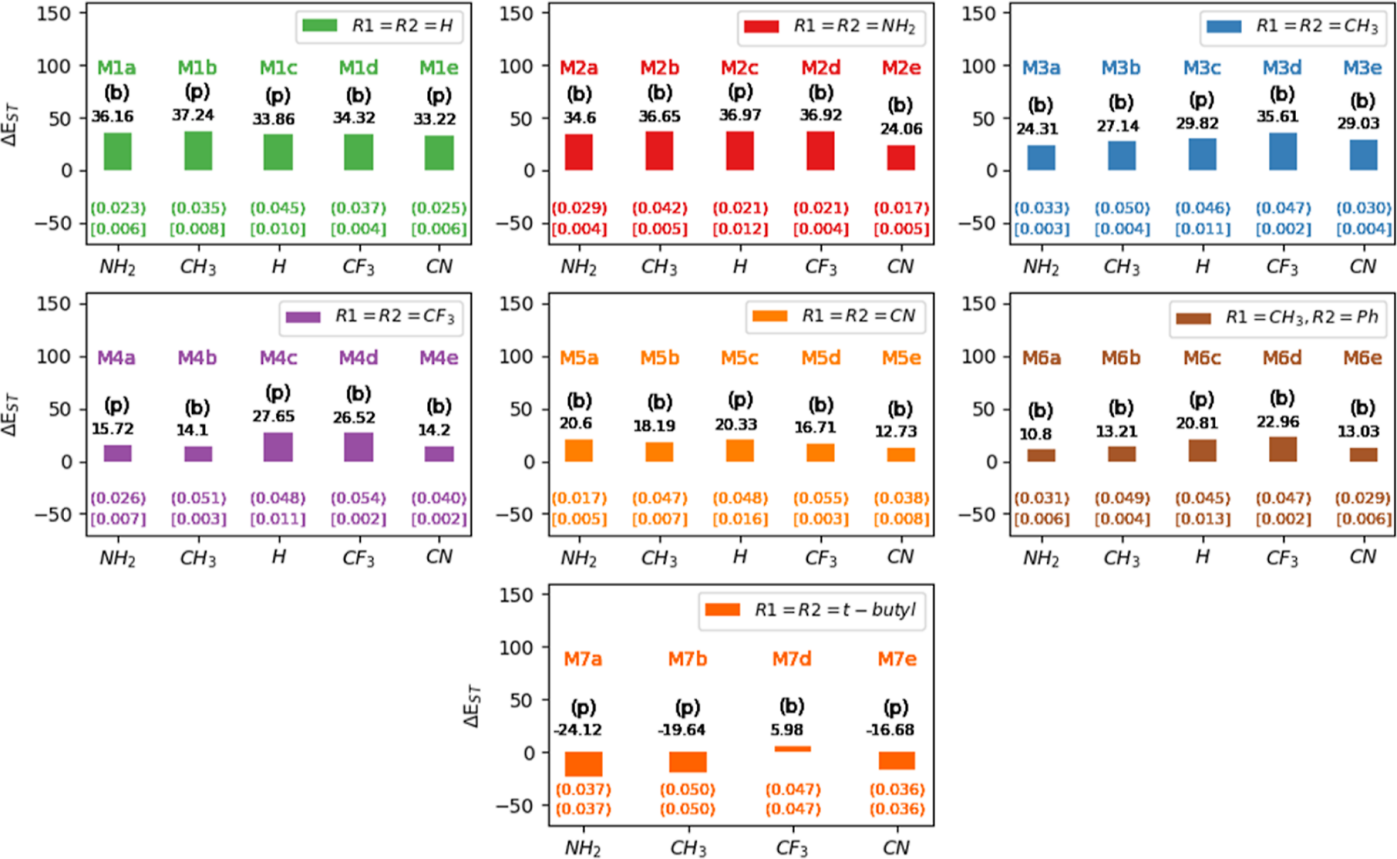
Calculated adiabatic Δ*E*_ST_ energy values (singlet quinoidal–triplet diradical, in kcal·mol^–1^) for the models **M1–M7** computed at the DLPNO-CCSD(T) level of theory. The values in parenthesis correspond to the MCI aromaticity index of the triplet state, and values in brackets correspond to the MCI aromaticity index of the quinoidal singlet state. The letters b and p stand for the “boat” or “planar” conformations of the quinoidal singlet electronic states (**B** and **C** conformations in Figure 3).

The main results regarding the **M1–M7** compounds are collected in Figure 4. The singlet electronic state with a quinoid structure is energetically the most stable for all **M1** to **M6** compounds, with Δ*E*_ST_ rising to 37 kcal·mol^–1^, depending on the nature of the substituents. At variance, for **M7** all compounds but **M7d** have a diradical character, the diradical triplet state lying up to 24 kcal·mol^–1^ below the singlet quinoidal electronic state in the case of **M7a**. Interestingly, the case of **M7c** results in a degenerated singlet–triplet ground state, at variance of the rest of **MXc** compounds, an indication of the large impact of steric interactions of outer groups to stabilize the triplet state.

All compounds with R_3_ = H (MXc, Figure 4) have a planar structure. The ground electronic state of the parent compound **M1c** (*p*QDM) is a quinoidal singlet state and the diradical triplet state lies higher in energy (Δ*E*_ST_ = 33.86 kcal·mol^–1^), as expected by the differential correlation effects associated to the closed-shell structure of the singlet state. These results are in very good agreement with the 33.34 kcal·mol^–1^ reported at the MRMP2(8,8) level in the literature.^63^ The singlet ground state of **M1c** has been also determined by NMR experiments.^64^ Our predicted Δ*E*_ST_ gap increases to 36.97 kcal·mol^–1^ for **M2c** (R_1_ = R_2_ = NH_2_) but decreases as the terminal substituents have more EWG character to the value of 20.33 kcal·mol^–1^ for **M5c** (R_1_ = R_2_ = CN), showing different effects of the substitutes in R_1_ and R_2_ on the singlet quinoid electronic states with respect to the triplet diradical electronic states. Thus, for instance, Figure 4 (and Table S1 in the Supporting Information) shows that the π donor character of the NH_2_ substituents (**M2c**) produces an important decrease of the aromaticity of the main ring in the triplet diradical (MCI = 0.021) and a slight increase of the π character of the C1–C7 and C4–C8 bonds (as described by the bond ellipticity value according to the AIM theory ε = 0.225; see Table S2 and Figure 3 for atom numbering), which causes a destabilization of the diradical with respect to the quinoidal structure, consequently increasing the Δ*E*_ST_ gap. For the sake of comparison, the aromatic MCI index of benzene is computed to be 0.073, whereas the MCI indexes of the MXc triplet states range between 0.045 and 0.048 and the ε values of the C1–C7 and C4–C8 bonds range between 0.120 and 0.153 (see Table S2). On the other side, the CN substituents produce a decrease of the π character of the C1–C7 and C4–C8 bonds (ε = 0.260) of the quinoidal valence-bond form producing a destabilization and thus reducing the Δ*E*_ST_ gap. Interestingly, no quinoidal structure was found when R_1_ = R_2_ = *t*-butyl (**M7**), where both the singlet and triplet electronic states have a diradical character. Here we remark that **M7** compounds show an important stabilization of the triplet electronic state, **M7c** being an interesting diradical candidate to be synthetized due to its inherent stability.

For compounds with substituents other than H in R_3_, the situation is much more complex and the Δ*E*_ST_ energy gap depends on multiple factors. In these cases, the different nature of the substituents in R_1_, R_2_, and R_3_ cause captodative effects^65,66^ in which the inductive effects of the substitutes are different in the diradical triplet electronic state compared to those in the quinoidal singlet electronic states. In this case, steric effects become the dominant force at determining whether we have a diradical ground state or not. These steric effects are found in both singlet and triplet states, leading to torsion of the substituents. Such torsion largely reduces the conjugation (Figure 3) in singlet states, although this is not the case for triplet states, as both C1–C7 and C4–C8 bonds are already single bonds. Singlet states can avoid steric repulsion through the adoption of the boat conformation but giving rise to an important decrease of conjugation. And then, we may obtain the diradical singlet or the triplet, depending on the energy difference of the two SOMOs (single-occupied molecular orbitals). For instance, in the case of the quinoidal singlet electronic states, there is a significant steric effect due to the size of the substituents which forces all compounds but **M4a** to adopt a boat structure, with boat angles (BA) ranging between 11 and 64°, indicating the deviation from planarity of the central ring. In order to estimate the amount of this effect,^67^ we have taken the quinoidal **M1b** and **M1c** systems, which have a planar structure in its ground state, and we have calculated the corresponding boat structure at restricted BAs of 20, 40, and 60°, allowing to fully relax all coordinates but the boat angle. The corresponding destabilization energies are 1.0, 8.1, and 28.8 kcal·mol^–1^ and 3.9, 17.0, and 44.9 kcal·mol^–1^ for **M1b** and **M1c**, respectively. And, in case of the triplet states, the steric effects may produce a distortion of the substituents and a significant loss of planarity in the main ring (PA angles between 15 and 21° when R_3_ = CF_3_). Now, we have estimated the effect of this loss of planarity, taking the structure of the triplet **M1c** and modifying the structure of the main ring as in **M2d** (PA 19°, Table S1 in the Supporting Information) with a destabilization energy of 7.35 kcal·mol^–1^ or modifying the structure of the main ring and the terminal substituents as in **M7d** (PA = 19°) and also modifying the dihedral angle between the R1/R2 groups and the main ring (DA = 75°, Table S1 in the Supporting Information), which lead to a destabilization of 22.42 kcal·mol^–1^. Furthermore, according to the AIM theory,^53−55,68,69^ the nature of the different substitutes generate other kinds of interactions such as hydrogen bonds, destabilizing interactions, and even hydrogen–hydrogen interactions, contributing the Δ*E*_ST_ energy gap.

Although it is very difficult to identify the individual contribution of each of these effects, we can conclude that compounds having EWG in the terminal position (i.e., **M4** and **M5**) present smaller Δ*E*_ST_ energy gaps, whereas the nature of the substituents in R_3_ plays a minor role, with the exception of R_3_ = CF_3_, which causes an important loss of planarity in the main ring of the diradical triplet state, producing a destabilization effect. However, the steric effects of the terminal groups also play a major role, as it is clearly seen in **M7** compounds. For the triplet electronic states, the terminal *t*-butyl groups are perpendicularly oriented with respect to the main ring in such a way that the corresponding unpaired electrons are orthogonal to the main ring so that no interaction is possible between the π system of the ring and the unpaired electrons and the triplet states are strongly stabilized. On the contrary, the singlet quinoidal states show a structure with a large BA (between 44 and 64°, see Table S1) due to the size of the *t*-butyl substitutes with the corresponding destabilization effect. Thus **M7a**, **M7b**, **M7c**, and **M7e** have a diradical (triplet or singlet open shell) ground state, whereas for **M7d** the ground state possesses a quinoidal structure because of the destabilization of the triplet state originated by the loss of planarity of the main ring. Further details of these effects along with the atoms in molecules and natural bond orbitals analyses are given in section S4 in the Supporting Information. For completeness, beyond Δ*E*_ST_, we have also looked for additional possible conformers of the singlet electronic states with quinoidal or diradical character (see e.g., section S4 in the Supporting Information).

### Compounds **T1–T8**

**Figure 5:**
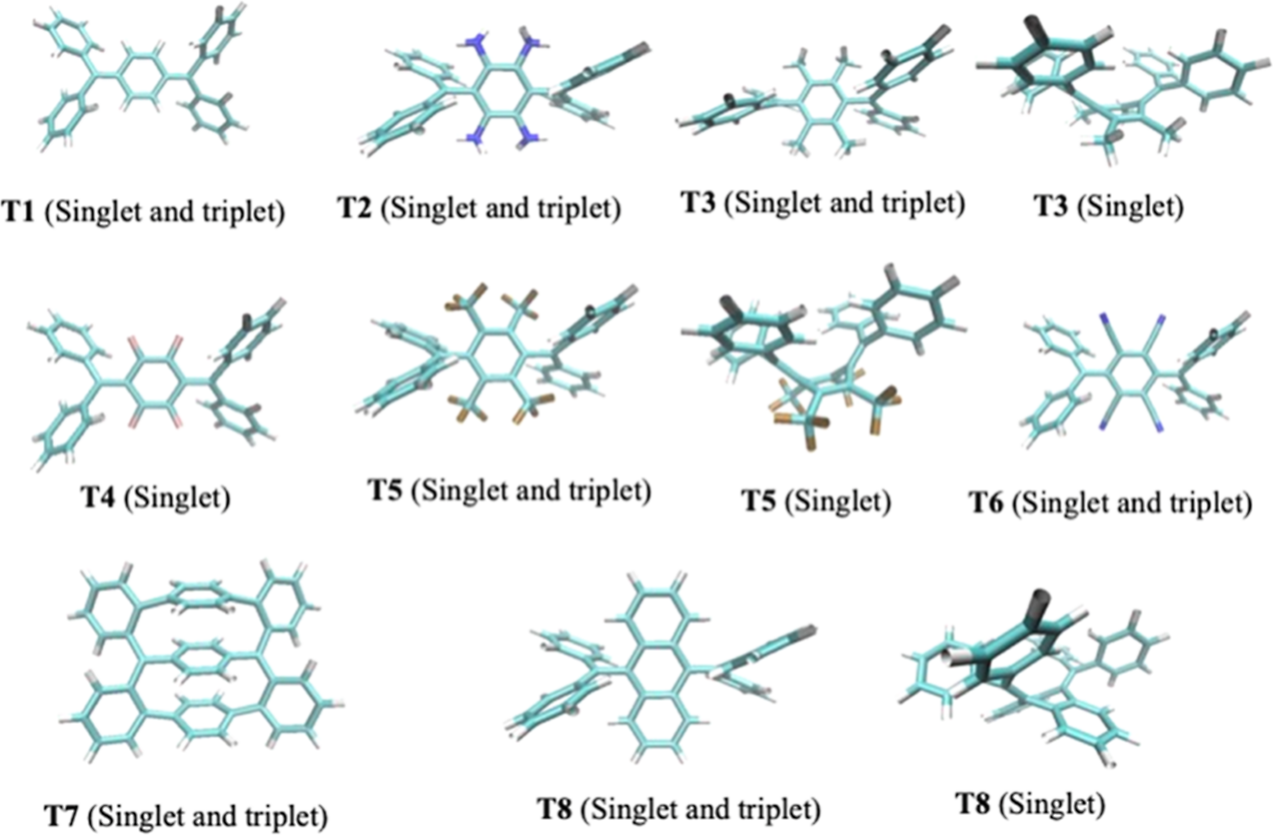
Structures of the Thiele like compounds investigated.

Steric hindrance of the radical centers is an essential ingredient for the chemical stability of Thiele’s hydrocarbons (as well as for Tschitschibabin’s and Müller’s) which is missing in the previous structures (except perhaps for **M7**). In addition, the possible conjugation of the unpaired electrons with the Ph groups provides an additional stabilization mechanism for Thiele’s hydrocarbon and its analogues. For the analysis of these systems, it is also important to remark that for compounds **M6** described above, having two EDG methyl substituents in R_1_ and two EWG phenyl substituents in R_2_, we predict Δ*E*_ST_ energy values of about 10–15 kcal·mol^–1^ lower than those of **M3** compounds (with terminal CH_3_ groups only), or even lower than for some compounds of **M4** and **M5** (with only EWG in R_1_), thus indicating a larger effect of the phenyl substituents. Thus, because of the interest of Thiele like compounds with four phenyl groups as terminal substitutes, we have further analyzed the series of compounds **T1–T8** in which we have considered the phenyl substituents in R_1_ and R_2_, combined with different substituents in R_3_ (H, NH_2_, CH_3_, F, CF_3_, CN, BRD—bridged phenyl groups (including a forced π-stacking interaction), and ANT—anthracene, Figure 5).

The ground state of the parent Thiele compound (**T1**) is a singlet state with a quinoidal structure^70^ and our optimized geometrical parameters compare very well with the crystallographic data reported in the literature,^71^ with differences smaller than 0.01 Å. Our computed Δ*E*_ST_ value is 16.89 kcal·mol^–1^, in very good agreement with the 16.14 kcal·mol^–1^ from the literature.^72^ This value is about 17 kcal·mol^–1^ smaller than the Δ*E*_ST_ value calculated for pDQM (**M1c**) due to the EWG and resonance role of the Ph substituent.

**Figure 6:**
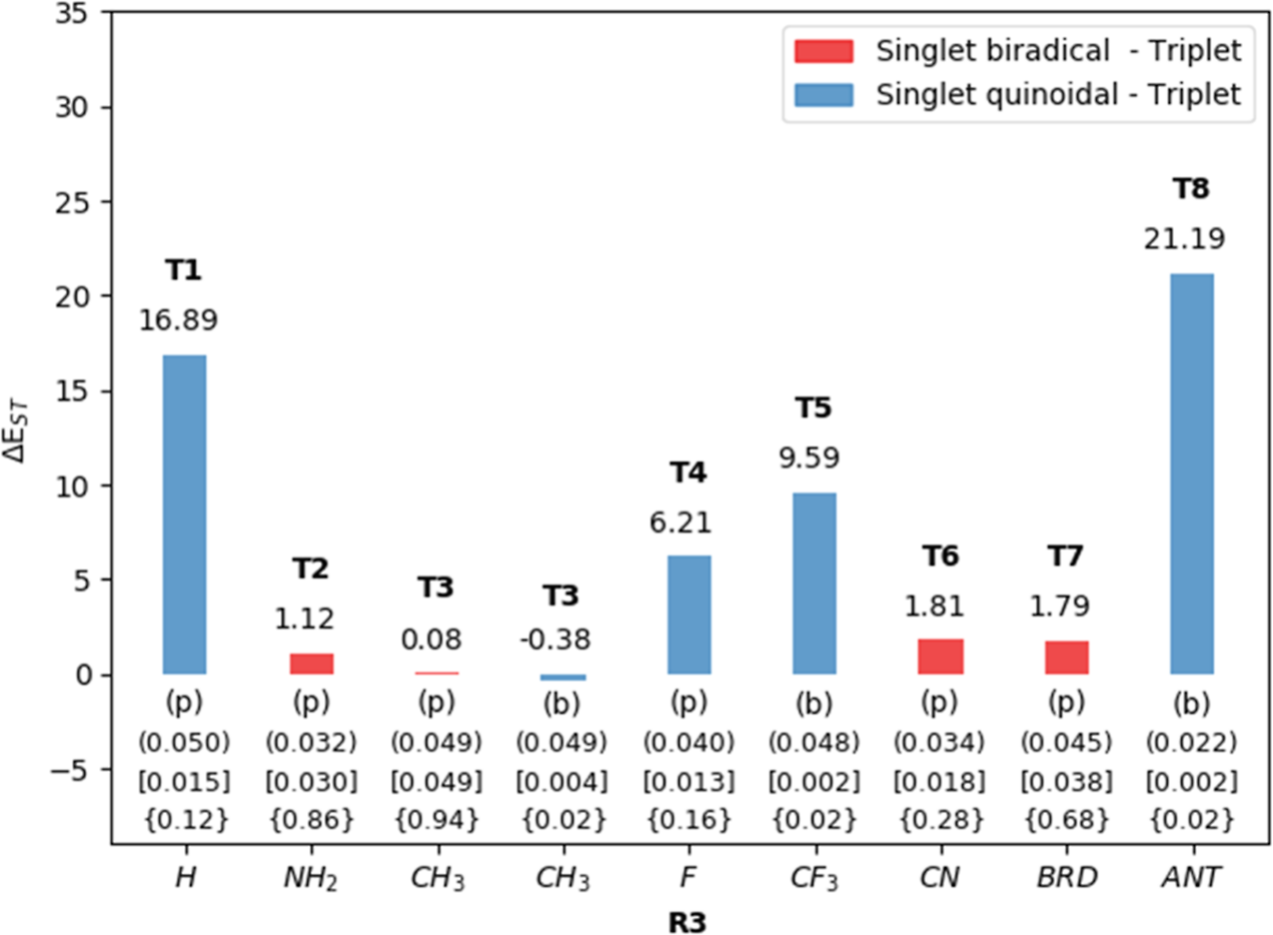
Calculated adiabatic Δ*E*_ST_ energy values (in kcal·mol^–1^) for the Thiele like compounds **T1** to **T7** computed at DLPNO-CCSD(T) for quinoidal structures and at B3LYP for the diradical structures. The values in parentheses correspond to the MCI aromaticity index of the triplet state, values in brackets correspond to the MCI aromaticity index of the singlet state, and values in braces correspond to the diradical character of the singlet states. The letters b and p stand for the “boat” or “planar” structures of the singlet electronic states. Please note that compounds with Δ*E*_ST_ < 4–6 kcal·mol^–1^ can show thermally accessible magnetic activity.

Figure 6 shows that **T2**, **T3**, **T6**, and **T7** have either a very small Δ*E*_ST_ or both states are almost degenerate, so that these species are expected to be magnetically active (EPR and paramagnetic response). A very interesting finding is that the ground singlet state of these compounds has a planar structure with a diradical character (BC = 0.86 for **T2**, 0.94 for **T3**, 0.28 for **T6**, and 0.68 for **T7**), with similar aromatic features than the corresponding triplet electronic state. No further conformers with quinoidal nature were found except for **T3** that has a boat (quinoidal) conformer, which is almost degenerate with respect to the singlet diradical and triplet states. The energy barrier connecting these two singlet conformers of **T3** is calculated to be 7 kcal·mol^–1^ (see section S5 in the Supporting Information). For all these compounds, the computed Δ*E*_ST_ values are smaller than 2 kcal·mol^–1^, and thus, they will be magnetically active.

On the other side, **T1**, **T4**, **T5**, and **T8** have quinoidal singlet ground states, and Δ*E*_ST_ ranges between 6.21 and 21.19 kcal·mol^–1^. Moreover, only **T5** and **T8** have conformers of singlet states with a diradical character in their potential energy surface but lying higher in energy (9.13 and 20.89 kcal·mol^–1^, see section S5 in the Supporting Information).

**Figure 7:**
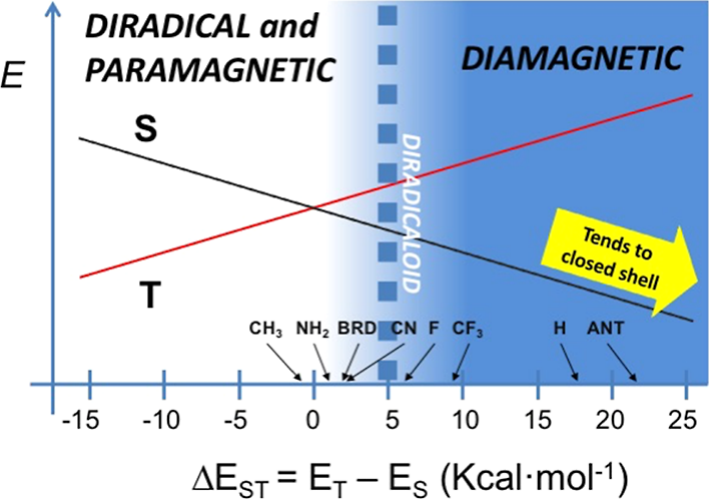
Scheme showing the tendency from paramagnetic to diamagnetic behavior of the Thiele like compounds investigated with respect to R_3_. The dashed line around 5 kcal·mol^–1^ suggests a limiting value for experimental observation of the paramagnetic response (e.g., EPR).

Importantly, the comparison of Thiele like compounds with models **M3** and **M7** clearly show that the nature of the substituent in R_3_ not only affects the Δ*E*_ST_ but also the electronic properties (diradical or quinoidal) of the lowest (ground) single state and the features of the potential energy surface. In particular, we have shown that by changing two terminal CH_3_ substituents in **M3** with two phenyl groups (**M6**), the Δ*E*_ST_ energy gap decreases by 10–15 kcal·mol^–1^, and the results in Figure 6 show that a further substitution of the two CH_3_ substituents in **M6** by two Ph groups produces an additional reduction of Δ*E*_ST_ by 5–13 kcal·mol^–1^. As a whole, the behavior of the Thiele like compounds can be summarized in Figure 7, which shows the changes from diradical-paramagnetic behavior to diamagnetic behavior of the different compounds studied according to the electronic nature of the substituents in R_3_, showing a blue diffuse region which indicates the evolution to diradicaloid structure as discussed in ref (73).

**Figure 8:**
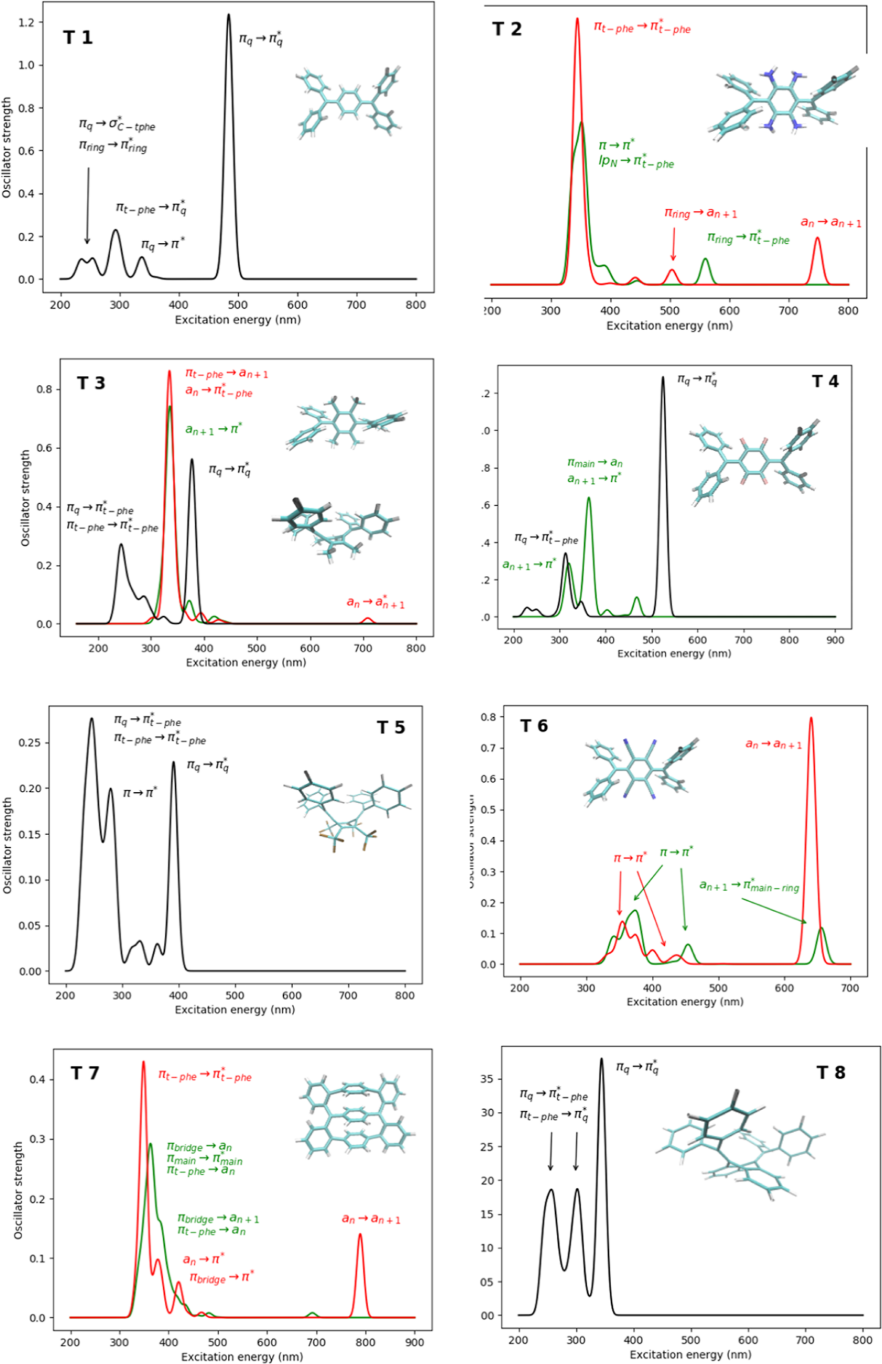
Calculated electronic spectra of the Thiele like compounds **T1–T8** computed at the TDDFT level of theory and approximate assignation of the corresponding transitions. Black lines correspond to absorption of quinoid singlet states; red lines correspond to absorption of diradical singlet states; and green lines correspond to absorption of triplet states. The labels *a*_*n*_ → *a*_*n*+1_ indicate the orbitals with unpaired electrons, characterizing the diradical character.

Finally, the electronic spectra of all Thiele like compounds have been computed by means of TDDFT (Figure 8). For **T1**, **T5**, and **T8**, only the quinoidal singlet state has been computed because the diradical states lie higher than 9 kcal·mol^–1^ in energy, and it is expected that they will not be populated. For the remaining compounds, the spectra of both the singlet and triplet electronic states have been computed, even in the case of **T4** where the triplet state lies 6.21 kcal·mol^–1^ above the singlet, but we assume that both states may be populated.

For the quinoid singlet states, we predict intense absorption bands in the near UV and in the visible region (λ_abs_ = 484 nm, *f*_osc_ = 1.24 for **T1**; λ_abs_ = 378 nm, *f*_osc_ = 0.56 for **T3**; λ_abs_ = 525 nm, *f*_osc_ = 1.29 for **T4**; λ_abs_ = 391 nm, *f*_osc_ = 0.22 for **T5**; and λ_abs_ = 343 nm, *f*_osc_ = 0.38 for **T8**), which corresponds to π_q_ → π_q_* transition (π_q_ = quinoid character), along with excitation band ranging between 230 and 290 nm region involving mainly π → π* excitation types. The triplet electronic states (**T2**, **T3**, **T4**, **T6**, and **T7**) absorb in the 300–400 nm region, mainly involving π → π* transitions and transition between the π system and the *a*_*n*_ and *a*_*n*+1_ orbitals with unpaired electrons in a system of 2*n* electrons (see Supporting Information). The singlet electronic states with a diradical character (**T2**, **T3**, **T6**, and **T7**) show similar absorption bands to the triplet states with the same features, but, in addition, they have a fingerprint in the near IR region involving transitions between the orbitals characterizing the diradical character (*a*_*n*_ → *a*_*n*+1_). Very interestingly, the intensity of these bands strongly depends on the diradical character, namely the occupation of the *a*_*n*_ and *a*_*n*+1_ orbitals. The larger the diradical character, the less intense the bands. Thus, for **T2** with BC = 0.86, λ_abs_ = 748 nm, *f*_osc_ = 0.11; for **T3** with BC = 0.946, λ_abs_ = 708 nm, *f*_osc_ = 0.02 (band negligible); for **T6** with BC = 0.28, λ_abs_ = 649 nm, *f*_osc_ = 0.80; and for **T7** with BC = 0.68, λ_abs_ = 789 nm, *f*_osc_ = 0.14.

## Conclusions

Our research has shown that the nature of the substituents in the derivatives of *p*QDM and Thiele like compounds, both in terminal position and in the main ring, have a great impact in the Δ*E*_ST_ energy gap and also in the electronic features (diradical or quinoidal) of the corresponding electronic state. All **M1** to **M6** model compounds have a quinoidal singlet state as ground state, and the corresponding diradical triplet state lies higher in energy between 11 and 37 kcal·mol^–1^. The situation is opposite for **M7** compounds with bulky *t*-butyl substituents, where the triplet and singlet diradical compounds lie lower in energy than the corresponding quinoidal singlet state, except **M7d**. These enormous changes in the Δ*E*_ST_ energy gap depend on steric effects and on the EWG character of the terminal substituents, the steric hindrance being the most significant effect as shown in the case of **M7**. Also, **M7** compounds show the most important stabilization of the triplet electronic state, **M7c** being an interesting diradical candidate to be synthetized due to its inherent stability.

For the Thiele like compounds, if the substituents R_3_ are EWG, the quinoidal form is preferred with low or almost null diradical character. On the other hand, EDG substituents in R_3_ favor the aromatic-diradical form if the electron donation does not exceed 6-π electrons. This situation can be extended for π-stacking in **T7**, where nearby Ph rings can be seen as electron-donating groups to the bridge. Finally, we predict that most of these species absorb in the visible region and that those low-lying singlet electronic states with a diradical character can also absorb in the near IR region. These results provide valuable data to interpret the optical and IR absorption experiments to identify possible bands due to the low-lying electronic states described for these systems when synthesized.

## Data Availability

The data underlying this study are available in the published article and its Supporting Information.

## Supporting Information Available

Computational methods, electronic features, and discussion on the model compounds and Thiele like compounds with energy values, cartesian coordinates, and AIM results (PDF)
