# Supplementary material for: Controlling the Diradical Character of Thiele Like Compounds
Source: J Org Chem. 2023 Jun 20;88(13):8553–62. doi: 10.1021/acs.joc.3c00482 (PMC10336959; doi:10.1021/acs.joc.3c00482)
Supplement: Supplementary file 1 — jo3c00482_si_001.pdf [file jo3c00482_si_001.pdf]

SUPPORTING INFORMATION

Controlling the Diradical Character of Thiele like Compounds

Josep M. Anglada,[a,*] Jordi Poater,[b,c] Ibério de P. R. Moreira,[c] and Josep Maria Bofill[b]

a) *Departament de Química Biològica (IQAC-CSIC), Carrer Jordi Girona, 18; 08034 Barcelona, Spain;*
b) *Departament de Química Inorgaǹica i Orgaǹica & IQTCUB, Universitat de Barcelona, Martí i Franquès 1-11, 08028 Barcelona, Spain*
c) *ICREA, Pg. Lluís Companys 23, 08010 Barcelona, Spain*
d) *Departament de Ciència de Materials i Química Física, Secció de Química Física, Universitat de Barcelona, 08028 Barcelona, Spain; & IQTCUB, Universitat de Barcelona,* Martí i Franquès, 1-11, *08028 Barcelona, Spain*

*email: anglada@iqac.csic.es

Contents

S1 Reliability of the computed ΔE_{ST} energy values S3

Figure S1. Differences (in kcal·mol^{-1}) of the ΔE_{ST} energies computed at B3LYP and at DLPNO-CCSD(T) **S3**

Figure S2. Differences (in kcal·mol^{-1}) of the ΔE_{ST} energies of singlet electronic states with diradical character, computed at FICMR and at B3LYP. **S4**

S2. Electronic features PS6

Figure S3. Most significant natural orbitals of the quinoidal singlet electronic states (M1c and M6b) and the singlet and triplet diradical electronic states of M6b. $C1^2$ and $C2^2$ stand for the coefficients of the CASSCF(2,2) wavefunction, and BC stands for the diradical character of the singlet electronic states. **S6**

S3. Further discussion on model compounds M1 to M7 PS5

Table S1. Adiabatic relative energies (in kcal·mol^{-1}), diradical character (BC) of the planar singlet electronic states, MCI aromaticity index of the central ring, boat angle (BA in degrees), [a] and the planarity angle (PA)[b] for the model compounds investigated. **S7**

Table S2. Density (ρ in a.u.), Laplacian ($\nabla^2\rho$ in a.u.), ellipticity (ε), energy density (H in hartree), of bond critical points of the C1-C7, and C4-C8 bonds along with the Wiberg index of these bonds and the natural charges over the main ring and over all the substituent. The natural charge over the same substituents on benzene are also give for comparison. See Figure 1c of the main text for atom numbering. **S10**

Figure S4. AIM critical points and paths connecting the bond critical points for the triplet and quinoidal singlet states of M7a and M7d.

Figure S5. Relative energies (in kcal·mol^{-1}) between different conformers of singlet electronic states.

S4. Further discussion on Thiele like compounds PS21

Table S3. Adiabatic relative energies (in kcal·mol^{-1}), diradical character (BC) of the planar singlet electronic states, MCI aromaticity index, the boat angle (BA),.[a] and the planarity angle (PA).[b]
Figure S6. Schematic potential energy surface for the singlet electronic states of **T3**, **T5**, and **T8**. The energies, including the zero-point energy corrections, are in kcal·mol^{-1}.
Table S4. Calculated absolute energy values (in hartree) for all model compounds obtained at different levels of theory, zero point energies (ZPE) obtained at B3LYP level or theory, and diradical character (BC) of the singlet electronic states.
Table S5. Calculated absolute energy values (in hartree) for all obtained at different levels of theory, zero point energies (ZPE), obtained at B3LYP level or theory, and diradical character (BC) of the singlet electronic states for all Thiele like compounds.
S5. Cartesian coordinates PS31
Table S6. Cartesian coordinates. (in Å) of all stationary points of the model compounds investigated in this work.
Table S7. Cartesian coordinates. (in Å) of all stationary points of the Thiele and Thiele like compounds investigated in this work.
Table S8. Cartesian coordinates of the AIM critical points, bonding features, density (ρ in a.u.), Laplacian of the density ($\nabla^2\rho$ in a.u.), ellipticity (ε), energy density (H in hartree), and signature of the AIM critical points for the triplet state and the quinoidal singlet states of compounds M1 – M5 and M7. Atom numbering according Table S6.

S1. Reliability of the computed ΔE_{ST} energy values

In the main text, we have pointed out the importance of predicting accurate ΔE_{ST} energy values. Provided that we are facing with electronic states with different multiplicities and electronic characteristics, we have to employ different theoretical methodologies in order to obtain relative energy values capable to predict very accurate results. Thus, depending on the electronic nature of the states we are dealing with, we have considered relative energies resulting from a given methodology and we have performed different cross test to ensure the accuracy of our results.

For all electronic states described by mono-referential methods, namely with triplet electronic states and singlet electronic states with quinoidal character, we have taken the energies obtained using the DLPNO-CCSD(T)/cc-pVTZ approach to calculate the ΔE_{ST} values. Regarding the energy gap obtained using the B3LYP approach, in Figure S1 we have plotted the $\Delta\Delta E_{ST}$ values obtained using both methods, which shows that the ΔE_{ST} values computed with DLPNO-CCSD(T) method are, in average, 7.52 kcal·mol^{-1} larger than those obtained using the B3LYP approach.

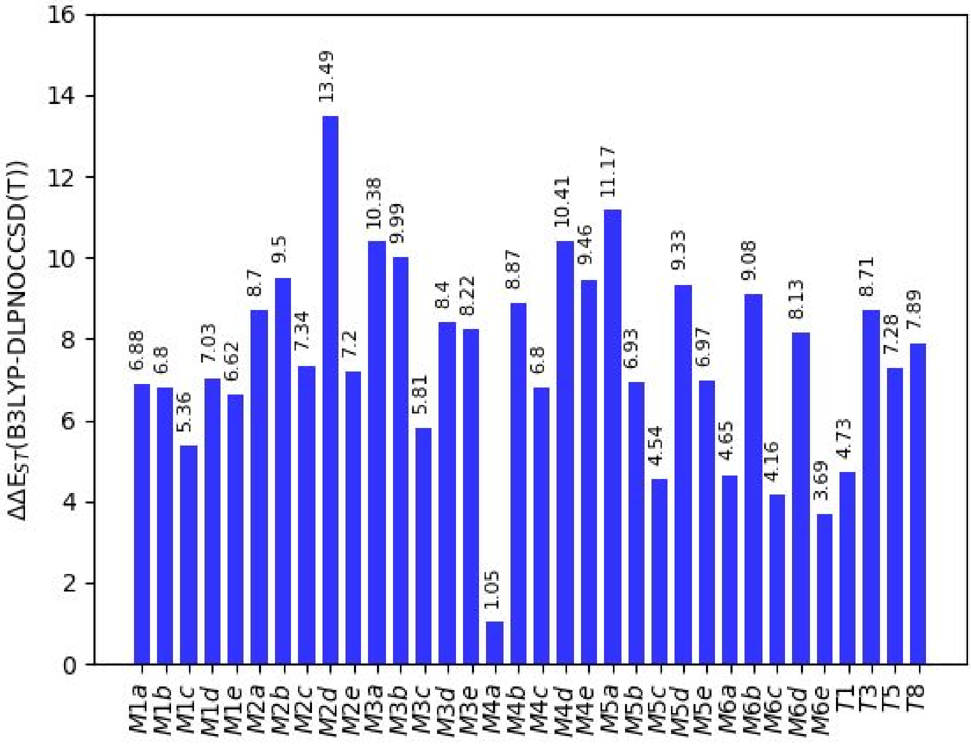

Figure S1. Differences (in kcal·mol^{-1}) of the ΔE_{ST} energies computed at B3LYP and at DLPNO-CCSD(T)

The singlet electronic states of diradical character requires the use of multi-referential methods for an accurate description and therefore, for several of the model compounds having these electronic features, we have employed the FICMR with the AQCC variant for calculating the corresponding ΔE_{ST} energy gap. Such calculations are computationally very demanding and are not feasible for all the compounds investigated in this work. However, we have compared the singlet triplet energy gap obtained at FICMR level with that computed at B3LYP level with broken symmetry. The errors in the ΔE_{ST} values (singlet diradicals – triplet states) obtained with UB3LYP approach with respect to the FICMR method are plotted in Figure S2 and are at most 0.6 kcal·mol^{-1}. These results indicate that the UB3LYP method correctly describe the ΔE_{ST} gap in the case of singlet with diradical character, which is to be expected because both electronic states have similar correlation effects.

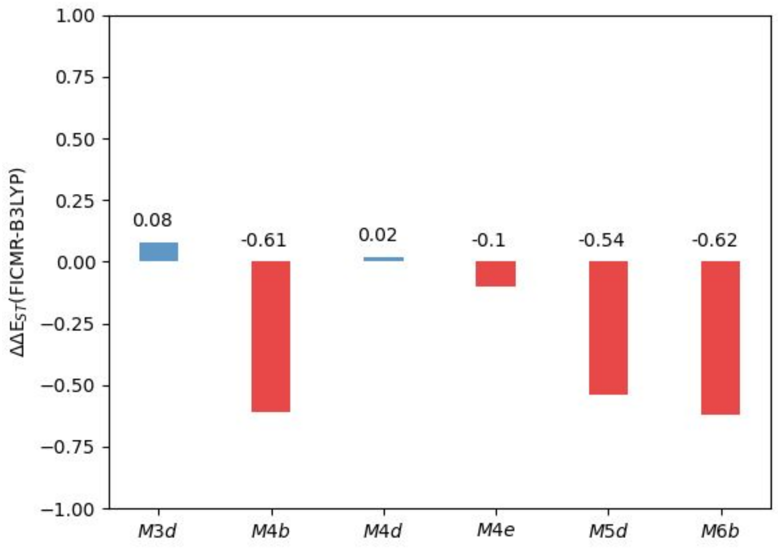

Figure S2. Differences (in kcal·mol^{-1}) of the ΔE_{ST} energies of singlet electronic states with diradical character, computed at FICMR and at B3LYP.

In view of the results discussed above, we have taken the results of the DLPNO-CCSD(T) level to calculate the ΔE_{ST} energies for electronic states described by mono-referential methods (triplet and quinoidal singlet states) and we have corrected the relative energy of the singlet diradical states taking the relative energy of the triplet obtained by DLPNO-CCSD(T) method and correcting the relative energy of the singlet diradical by the singlet-triplet difference either at FICMR or UB3LYP.

S2. Electronic features

In Figure 3 of the main text, we have pointed out that structure **A** describes triplet and singlet electronic states of diradical character, in which two unpaired electrons are mainly localized over the carbene atoms, and structures **B** and **C** are quinoid forms that do not have unpaired electrons and therefore they can describe singlet electronic states only. Please note from structure **A** that the main ring in the triplet state and singlet state with diradical character should show aromatic features, which forces it to have a planar structure. In order to illustrate the electronic features of these structures in more detail, we have plotted in Figure S3 the most significant natural orbitals of a CASSCF(2,2) wave-function describing the different electronic states. We have taken as examples **M1c** (pQDM) which is well described by structure **B**, and **M6b** whose triplet and singlet diradical electronic states are described by structure **A** and whose singlet quinoidal electronic state is described by structure **C**. For a compound having 2n electrons, the triplet electronic state is characterized by the electronic configuration ($a_1^2a_2^2a_3^2\cdots a_{n-1}^2a_n^1a_{n+1}^1$), and the singlet electronic state can be characterized by the C1($a_1^2a_2^2a_3^2\cdots a_{n-1}^2a_n^2$) + C2 ($a_1^2a_2^2a_3^2\cdots a_{n-1}^2a_{n+1}^2$) electronic configurations. According to the CASSCF(2,2) wave function, $C1^2 + C2^2 = 1$ and compounds having full diradical character fulfils $C1^2 = C2^2 = 0.5$. Consequently, the degree of the diradical character (BC) for a given singlet electronic states is determined by BC = $C2^2/0.5$ ($0 \leq BC \leq 1$). The diradical character for all compounds investigated in this work are collected in Tables S1 and S3 of this Supporting Information. Figure S3 contains the electronic features of the a_n and a_{n+1} orbitals, which shows the π character of the C1-C4, C4-C8, C2-C3, and C3-C6 bonds for the singlet quinoidal electronic state (structure B for M1c and structure C for M6b, see Figure 3 of the main text), and the unpaired electrons for the triplet and singlet electronic states (structure A for the diradical M6b). The value of the coefficients (C1 and C2) of these orbitals in the wave-function for the singlet states are also given, along the corresponding diradical character (BC).

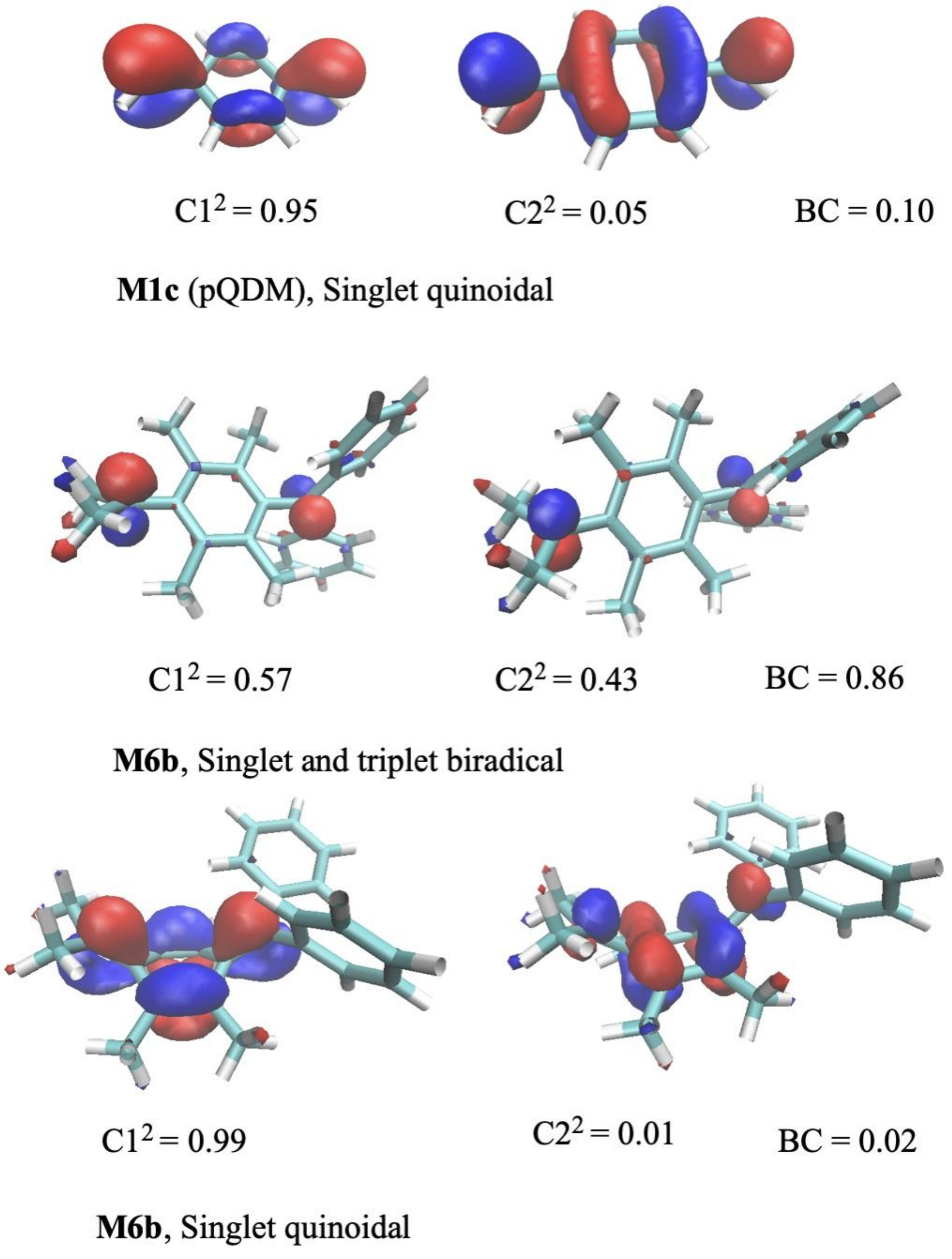

Figure S3. Most significant natural orbitals of the quinoidal singlet electronic states (M1c and M6b) and the singlet and triplet diradical electronic states of M6b. $C1^2$ and $C2^2$ stand for the coefficients of the CASSCF(2,2) wavefunction, and BC stands for the diradical character of the singlet electronic states.

S3. Further discussion on model compounds M1 to M7

In the main text we have outlined the main trends regarding the ΔE_{ST} energy gap of the models **M1** to **M7**, pointing out that that ground electronic state is predicted to be a singlet

state with quinoidal structure for all M1 to M6 compounds and a triplet diradical electronic state for all M7 compounds except M7d. In addition, we have also found additional conformers of the singlet electronic state for several of the models, either with quinoidal structure or with diradical character which are not fully discussed in the main text. The whole results regarding these models are collected in Table S1.

Table S1. Adiabatic relative energies (in kcal·mol^{-1}), diradical character (BC) of the planar singlet electronic states, MCI aromaticity index of the central ring, boat angle (BA in degrees), [a] and the planarity angle (PA)[b] for the model compounds investigated.

		**Singlet**						**Triplet**			
		**planar**			**boat**						
	**R3**	**E**	**BC**	**MCI**	**E**	**MCI**	**BA[a]**	**E**	**MCI**	**PA[b]**	**DA[c]**
					**M1 (R1=R2= H)**						
**M1a**	**NH$_2$**	0.77	0.08	0.007	0.00	0.006	11	36.16	0.023	5	8
**M1b**	**CH$_3$**	0.00	0.08	0.008	--	--	--	37.24	0.035	7	6
**M1c**	**H**	0.00	0.10	0.010	--	--	--	33.86	0.045	0	0
**M1d**	**CF$_3$**	--	--	--	0.00	0.004	33	34.32	0.037	21	14
**M1e**	**CN**	0.00	0.10	0.006	--	--	--	33.22	0.025	0	0
					**M2 (R1=R2= NH$_2$)**						
**M2a**	**NH$_2$**	6.47	0.08	0.008	0.00	0.004	27	34.60	0.029	4	53
**M2b**	**CH$_3$**	--	--	--	0.00	0.005	31	36.65	0.042	5	79
**M2c**	**H**	0.00	0.08	0.012	--	--	--	36.97	0.021	1	22
**M2d**	**CF$_3$**	--	--	--	0.00	0.004	39	36.92	0.021	19	39
**M2e**	**CN**	--	--	--	0.00	0.005	21	24.06	0.017	5	27
					**M3 (R1=R2= CH$_3$)**						
**M3a**	**NH$_2$**	6.97	0.12	0.008	0.00	0.003	33	24.31	0.033	4	62
**M3b**	**CH$_3$**	--	--	--	0.00	0.004	35	27.13	0.050	4	62
**M3c**	**H**	0.00	0.10		--	--	--	29.82	0.045	0	0
**M3d**	**CF$_3$**	34.88	0.86	0.040	0.00	0.002	44	35.61	0.047	16	62
**M3e**	**CN**	--	--	--	0.00	0.004	29	29.03	0.030	3	45
					**M4 (R1=R2= CF$_3$)**						
**M4a**	**NH$_2$**	0.00	0.02	0.007	4.55	0.005	22	15.72	0.026	4	49/81
**M4b**	**CH$_3$**	13.44	1.00	0.050	0.00	0.003	44	14.10	0.051	0	82
**M4c**	**H**	0.00	0.10	0.011	--	--	--	27.65	0.048	1	31
**M4d**	**CF$_3$**	26.50	1.00	0.054	0.00	0.002	48	26.52	0.054	15	75
**M4e**	**CN**	14.01	0.98	0.039	0.00	0.002	40	14.20	0.040	0	81
					**M5 (R1=R2= CN)**						

**M5a**	**NH_2**	--	--	--	0.00	0.005	17	20.60	0.017	8	25
**M5b**	**CH_3**	--	--	--	0.00	0.007	29	18.19	0.047	5	56
**M5c**	**H**	0.00		0.016	--	--	--	20.33	0.048	0	0
**M5d**	**CF_3**	16.09	0.96	0.055	0.00	0.003	40	16.71	0.055	16	68
**M5e**	**CN**	--	--	--	0.00	0.008	21	12.73	0.038	1	57
				**M6 (R1= CH_3 , M2 = Phe).** [c]							
**M6a**	**NH_2**	4.05	0.22	0.010	0.00	0.006	27	10.80	0.031	5	45
				(0.058)		(0.060)			(0.053)		69
**M6b**	**CH_3**	12.44	0.86	0.044	0.00	0.004	35	13.32	0.049	3	59
				(0.054)		(0.064)			(0.054)		72
**M6c**	**H**	0.00	0.10	0.013	--	--	--	20.81	0.045	0	0
				(0.063)					(0.054)		51
**M6d**	**CF_3**	22.44	0.88	0.044	0.00	0.002	43	22.96	0.047	16	61
				(0.054)		(0.066)			(0.054)		65
**M6e**	**CN**	--	--	--	0.00	0.006	26	13.03	0.029	2	36
						(0.061)			(0.053)		70
				**M7 (R1=R2= t-butyl)**							
**M7a**	**NH_2**	0.00	0.98	0.037	24.15	0.001	64	0.03	0.037	2	89
**M7b**	**CH_3**	0.09	1.00	0.050	19.66	0.002	47	0.02	0.050	2	88
**M7c**	**H**	0.00	0.96	0.058	--	--	--	0.09	0.058	0	81
**M7d**	**CF_3**	5.93	0.98	0.047	0.0	0.002	50	5.98	0.047	19	75
**M7e**	**CN**	0.00	0.96	0.036	16.74	0.002	44	0.06	0.036	0	87

a) BA (boat angle) corresponds to maximum deviation from planarity of the central ring.
b) PA corresponds to deviation of planarity of the main ring in the triplet electronic state
c) Values in parenthesis correspond to the MCI values of the terminal phenyl substituent.
d) DA stands for dihedral angle between the R1/R2 groups and the main ring.

One of the main focuses of this investigation refers to the study of the dramatic changes in the relative energies between the singlet quinoidal electronic states and the triplet diradical electronic states (ΔE_{ST}). We have pointed out in the main text that there are multiple causes for the important changes in the ΔE_{ST} energy gap as shown in Figure 4 of the main text and in Table S1. We have mentioned different factors, as steric effects, differential captodative effects in states of different multiplicity, hydrogen bond interactions, lone pair repulsion or even hydrogen – hydrogen interactions. In Table S2 we have collected the results of the AIM analysis of the C1-C7 and C4-C8 bonds (see Figure 3 of the main text for numbering), as well as the natural charges corresponding to all substitutes, C7, C8 and the main ring. For the sake of comparison, in Table S2 we have also collected the natural charges of benzene, 1,2,4,5-NH_2 benzene, 1,2,4,5-CH_3 benzene, 1,2,4,5-CF_3 benzene, and 1,2,4,5-CN benzene and thus, we can take the NBO charges as

an indirect measure of the inductive effects of the substitutes. The whole results of the AIM analysis are given below in Table S8.

Table S2. Density (ρ in a.u.), Laplacian ($\nabla^2\rho$ in a.u.), ellipticity (ε), energy density (H in hartree), of bond critical points of the C1-C7, and C4-C8 bonds along with the Wiberg index of these bonds and the natural charges over the main ring and over all the substituent. The natural charge over the same substituents on benzene are also give for comparison. See Figure 3 of the main text for atom numbering.

**Multiplicity**	**R3**	**bond**	***ρ***	$\nabla^2\rho$	***ε***	**H**	**Wiberg index**	**charge C7/C8**	**charge Ring**	**charge R3**	**charge R1+R2**
				**M1 (R1 = R2 = H)**							
**Triplet**	NH_2	C1-C7	0.2917	-0.7441	0.1849	-0.2735	1.27	-0.423	0.241	-0.239	0.830
		C4-C8	0.2909	-0.7446	0.1694	-0.2718	1.26	-0.409			
**Singlet quinoidal**	NH_2	C1-C7	0.3308	-0.9246	0.3046	-0.3533	1.71	-0.464	0.299	-0.267	0.895
		C4-C8	0.3308	-0.9245	0.3046	-0.3532	1.71	-0.464			
**Triplet**	CH_3	C1-C7	0.2859	-0.7262	0.1447	-0.2620	1.22	-0.357	-0.248	0.123	0.832
		C4-C8	0.2859	-0.7263	0.1448	-0.2620	1.21	-0.347			
**Singlet quinoidal**	CH_3	C1-C7	0.3314	-0.9280	0.2973	-0.3531	1.73	-0.424	-0.181	0.135	0.890
		C4-C8	0.3314	-0.9280	0.2973	-0.3531	1.71	-0.418			
**Triplet**	H	C1-C7	0.2875	-0.7410	0.1387	-0.2644	1.20	-0.344	-1.124	0.970	0.842
		C4-C8	0.2875	-0.7410	0.1387	-0.2644	1.20	-0.344			
**Singlet quinoidal**	H	C1-C7	0.3339	-0.9489	0.2973	-0.3582	1.72	-0.404	-1.073	0.978	0.903
		C4-C8	0.3339	-0.9489	0.2973	-0.3582	1.72	-0.404			
**Triplet**	CF_3	C1-C7	0.2863	-0.7294	0.1391	-0.2646	1.21	-0.298	-0.491	0.152	0.937
		C4-C8	0.2864	-0.7299	0.1398	-0.2649	1.21	-0.298			
**Singlet quinoidal**	CF_3	C1-C7	0.3375	-0.9601	0.3329	-0.3696	1.81	-0.327	-0.511	0.169	0.996

		C4-C8	0.3376	-0.9606	0.3334	-0.3698	1.81	-0.327		
**Triplet**	CN	C1-C7	0.2933	-0.7676	0.1449	-0.2771	1.23	-0.285	-0.439	0.060	0.953
		C4-C8	0.2933	-0.7676	0.1449	-0.2771	1.23	-0.289			
**Singlet quinoidal**	CN	C1-C7	0.3358	-0.9604	0.2882	-0.3642	1.71	-0.320	-0.462	0.077	1.021
		C4-C8	0.3358	-0.9604	0.2882	-0.3642	1.71	-0.316			
				**M2 (R1=R2=NH_2)**							
**Triplet**	NH_2	C1-C7	0.2751	-0.6722	0.1939	-0.2442	1.11	0.288	0.143	-0.300	-0.412
		C4-C8	0.2737	-0.6679	0.1825	-0.2421	1.10	0.281			
**Singlet quinoidal**	NH_2	C1-C7	0.3182	-0.8580	0.4207	-0.3373	1.52	0.386	-0.084	-0.430	-0.250
		C4-C8	0.3177	-0.8555	0.4183	-0.3360	1.53	0.377			
**Triplet**	CH_3	C1-C7	0.2677	-0.6498	0.1037	-0.2305	1.03	0.268	-0.325	0.122	-0.355
		C4-C8	0.2814	-0.7016	0.2159	-0.2556	1.14	0.290			
**Singlet quinoidal**	CH_3	C1-C7	0.3221	-0.8781	0.4325	-0.3474	1.57	0.367	-0.538	0.049	-0.245
		C4-C8	0.3221	-0.8783	0.4327	-0.3475	1.57	0.367			
**Triplet**	H	C1-C7	0.2854	-0.6915	0.3251	-0.2638	1.15	0.304	-1.257	0.975	-0.324
		C4-C8	0.2847	-0.6878	0.3234	-0.2622	1.15	0.303			
**Singlet quinoidal**	H	C1-C7	0.3192	-0.8657	0.3865	-0.3351	1.48	0.359	-1.365	0.888	-0.240
		C4-C8	0.3192	-0.8657	0.3865	-0.3351	1.48	0.358			
**Triplet**	CF_3	C1-C7	0.2878	-0.7431	0.2004	-0.2715	1.17	0.465	-0.994	-0.164	0.230
		C4-C8	0.2880	-0.7439	0.2014	-0.2718	1.17	0.464			

**Singlet quinoidal**	CF_3	C1-C7	0.3158	-0.8495	0.4214	-0.3316	1.47	0.434	-0.888	0.038	-0.017
		C4-C8	0.3157	-0.8492	0.4197	-0.3314	1.47	0.435			
**Triplet**	CN	C1-C7	0.2862	-0.7378	0.2025	-0.2681	1.19	0.461	-0.871	-0.389	0.329
		C4-C8	0.2863	-0.7382	0.2025	-0.2683	1.18	0.471			
**Singlet quinoidal**	CN	C1-C7	0.3086	-0.8153	0.3683	-0.3114	1.39	0.427	-0.799	-0.144	0.094
		C4-C8	0.3086	-0.8151	0.3682	-0.3113	1.39	0.422			
					**M3 (R1=R2=CH_3)**						
**Triplet**	NH_2	C1-C7	0.2738	-0.6563	0.1481	-0.2406	1.13	0.030	0.225	-0.276	-0.017
		C4-C8	0.2651	-0.6260	0.0837	-0.2260	1.05	0.038			
**Singlet quinoidal**	NH_2	C1-C7	0.3243	-0.8698	0.3547	-0.3386	1.70	0.005	0.211	-0.314	0.092
		C4-C8	0.3270	-0.8829	0.3661	-0.3446	1.72	0.006			
**Triplet**	CH_3	C1-C7	0.2667	-0.6365	0.0886	-0.2281	1.05	0.054	-0.225	0.131	-0.020
		C4-C8	0.2667	-0.6363	0.0889	-0.2280	1.06	0.060			
**Singlet quinoidal**	CH_3	C1-C7	0.3289	-0.8932	0.3685	-0.3488	1.74	0.006	-0.225	0.114	0.098
		C4-C8	0.3289	-0.8929	0.3685	-0.3487	1.74	0.006			
**Triplet**	H	C1-C7	0.2800	-0.6933	0.1534	-0.2503	1.16	0.069	-1.121	0.959	0.023
		C4-C8	0.2800	-0.6934	0.1534	-0.2503	1.16	0.070			
**Singlet quinoidal**	H	C1-C7	0.3224	-0.8685	0.3183	-0.3330	1.61	0.023	-1.093	0.939	0.098
		C4-C8	0.3224	-0.8685	0.3183	-0.3330	1.61	0.034			
**Triplet**	CF_3	C1-C7	0.2696	-0.6489	0.1021	-0.2357	1.07	0.080	-0.439	0.160	0.121

		C4-C8	0.2695	-0.6486	0.1014	-0.2356	1.07	0.098			
**Singlet quinoidal**	CF_3	C1-C7	0.3314	-0.9095	0.3681	-0.3539	1.73	0.099	-0.560	0.146	0.223
		C4-C8	0.3315	-0.9061	0.3796	-0.3546	1.75	0.092			
**Triplet**	CN	C1-C7	0.2776	-0.6857	0.1314	-0.2487	1.12	0.099	-0.394	0.023	0.191
		C4-C8	0.2777	-0.6859	0.1317	-0.2488	1.12	0.087			
**Singlet quinoidal**	CN	C1-C7	0.3224	-0.8685	0.3183	-0.3330	1.61	0.023	-1.093	0.939	0.098
		C4-C8	0.3224	-0.8685	0.3183	-0.3330	1.61	0.034			
					**M4 (R1=R2=CF_3)**						
**Triplet**	NH_2	C1-C7	0.2633	-0.6321	0.0213	-0.2261	1.02	-0.033	0.354	-0.091	-0.043
		C4-C8	0.2798	-0.6744	0.1784	-0.2531	1.22	-0.189			
**Singlet quinoidal**	NH_2	C1-C7	0.3006	-0.7569	0.2952	-0.2909	1.45	-0.334	0.614	0.108	-0.053
		C4-C8	0.3009	-0.7583	0.2957	-0.2915	1.45	-0.334			
**Triplet**	CH_3	C1-C7	0.2619	-0.6249	0.0266	-0.2251	1.03	-0.022	-0.182	0.249	-0.022
		C4-C8	0.2619	-0.6249	0.0266	-0.2251	1.03	-0.022			
**Singlet quinoidal**	CH_3	C1-C7	0.3275	-0.8813	0.3546	-0.3470	1.76	-0.238	0.065	0.275	0.135
		C4-C8	0.3275	-0.8815	0.3547	-0.3471	1.76	-0.238			
**Triplet**	H	C1-C7	0.2761	-0.6822	0.1199	-0.2459	1.14	-0.063	-0.963	1.066	0.024
		C4-C8	0.2760	-0.6820	0.1198	-0.2458	1.14	-0.063			
**Singlet quinoidal**	H	C1-C7	0.3210	-0.8634	0.2980	-0.3308	1.61	-0.187	-0.899	1.091	0.113
		C4-C8	0.3210	-0.8633	0.2980	-0.3308	1.61	-0.187			

**Triplet**	CF_3	C4-C8	0.2589	-0.6029	0.0394	-0.2156	1.02	-0.040	-0.297	0.274	0.095
		C4-C8	0.2589	-0.6029	0.0397	-0.2156	1.02	-0.031			
**Singlet quinoidal**	CF_3	C1-C7	0.3345	-0.9144	0.3924	-0.3624	1.82	-0.152	-0.274	0.292	0.281
		C4-C8	0.3346	-0.9146	0.3927	-0.3626	1.82	-0.146			
**Triplet**	CN	C1-C7	0.2660	-0.6397	0.0357	-0.2266	1.02	-0.029	-0.287	0.187	0.155
		C4-C8	0.2660	-0.6397	0.0357	-0.2266	1.02	-0.025			
**Singlet quinoidal**	CN	C1-C7	0.3325	-0.9083	0.3736	-0.3576	1.78	-0.147	-0.218	0.188	0.319
		C4-C8	0.3326	-0.9085	0.3743	-0.3577	1.79	-0.142			
				**M5 (R1=R2=CN)**							
**Triplet**	NH_2	C1-C7	0.2795	-0.6746	0.2338	-0.2516	1.20	-0.336	0.662	0.319	-0.310
		C4-C8	0.2795	-0.6746	0.2337	-0.2516	1.20	-0.330			
**Singlet quinoidal**	NH_2	C1-C7	0.3003	-0.7703	0.2692	-0.2895	1.38	-0.292	0.623	0.118	-0.155
		C4-C8	0.3003	-0.7699	0.2692	-0.2894	1.38	-0.294			
**Triplet**	CH_3	C1-C7	0.2607	-0.6245	0.0819	-0.2235	1.03	-0.111	-0.089	0.301	0.012
		C4-C8	0.2608	-0.6246	0.0821	-0.2236	1.03	-0.112			
**Singlet quinoidal**	CH_3	C1-C7	0.3158	-0.8463	0.2949	-0.3211	1.53	-0.238	0.149	0.322	0.005
		C4-C8	0.3158	-0.8464	0.2950	-0.3211	1.53	-0.238			
**Triplet**	H	C1-C7	0.2729	-0.6752	0.1329	-0.2413	1.11	-0.104	-0.898	1.084	0.043
		C4-C8	0.2729	-0.6752	0.1329	-0.2413	1.11	-0.125			
**Singlet quinoidal**	H	C1-C7	0.3140	-0.8485	0.2604	-0.3162	1.45	-0.177	-0.774	1.107	0.042

		C4-C8	0.3140	-0.8485	0.2604	-0.3162	1.45	-0.199			
**Triplet**	CF_3	C1-C7	0.2557	-0.5996	0.0540	-0.2110	0.99	-0.108	-0.279	0.317	0.178
		C4-C8	0.2557	-0.5996	0.0542	-0.2110	0.99	-0.108			
**Singlet quinoidal**	CF_3	C1-C7	0.3259	-0.8911	0.3555	-0.3419	1.64	-0.139	-0.228	0.336	0.175
		C4-C14	0.3260	-0.8915	0.3557	-0.3421	1.63	-0.145			
**Triplet**	CN	C1-C7	0.2634	-0.6349	0.0829	-0.2230	1.02	-0.078	-0.245	0.204	0.206
		C4-C8	0.2634	-0.6350	0.0829	-0.2230	1.01	-0.087			
**Singlet quinoidal**	CN	C1-C7	0.3171	-0.8539	0.3027	-0.3219	1.52	-0.145	-0.182	0.226	0.251
		C4-C8	0.3171	-0.8540	0.3026	-0.3219	1.51	-0.150			
				**M7 (R1= R2= t-butyl)**							
**Triplet**	NH_2	C1-C7	0.2548	-0.5774	0.0269	-0.2097	1.02	0.156	0.166	-0.272	-0.199
		C4-C8	0.2546	-0.5779	0.0299	-0.2095	1.02	0.149			
**Singlet quinoidal**	NH_2	C1-C7	0.3171	-0.8197	0.3679	-0.3248	1.81	0.094	0.194	-0.336	-0.033
		C4-C8	0.3213	-0.8404	0.3801	-0.3335	1.83	0.082			
**Triplet**	CH_3	C1-C7	0.2544	-0.5799	0.0373	-0.2087	1.02	0.180	-0.284	0.141	-0.216
		C4-C8	0.2545	-0.5802	0.0373	-0.2088	1.02	0.180			
**Singlet quinoidal**	CH_3	C1-C7	0.3209	-0.8356	0.3871	-0.3334	1.85	0.073	-0.254	0.115	-0.007
		C4-C8	0.3207	-0.8350	0.3869	-0.3331	1.85	0.072			
**Triplet**	CF_3	C1-C7	0.2432	-0.5288	0.0687	-0.1957	1.03	0.170	-0.446	0.202	-0.093
		C4-C8	0.2430	-0.5280	0.0687	-0.1955	1.03	0.168			

**Singlet quinoidal**	CF_3	C1-C7	0.3190	-0.8274	0.3806	-0.3298	1.81	0.157	-0.553	0.133	0.107
		C4-C8	0.3192	-0.8286	0.3809	-0.3303	1.81	0.157			
**Triplet**	CN	C1-C7	0.2598	-0.6099	0.0510	-0.2222	1.02	0.145	-0.456	0.166	0.000
		C4-C8	0.2598	-0.6098	0.0509	-0.2222	1.03	0.145			
**Singlet quinoidal**	CN	C1-C7	0.3181	-0.8321	0.3429	-0.3271	1.75	0.174	-0.522	-0.015	0.182
		C4-C8	0.3183	-0.8325	0.3441	-0.3274	1.75	0.180			
				**1,2,4,5 R3-benzene**							
**singlet**	NH_2								-0.150	-0.303	
**singlet**	CH_3								-0.599	0.136	
**singlet**	H								-1.473	0.982	
**singlet**	CF3								-0.775	0.184	
**singlet**	CN								-0.687	0.111	

Regarding the singlet quinoidal electronic states, our calculations show that, depending on the substituents in R_3, they adopt a boat structure, which occurs in most of the compounds investigated, having a destabilization effect, the boat angle ranging between 11° and 64° (see Table S1). In order to estimate the amount of this effect we have taken the quinoidal M1b and M1c systems, which have a planar structure in its ground state, and we have calculated the corresponding boat structure at restricted boat angles of 20°, 40°, and 60°, allowing to fully relax all coordinates but the boat angle. The corresponding destabilization energies are 1.0, 8.1, and 28.8 kcal·mol^{-1}, and 3.9, 17.0, and 44.9 kcal·mol^{-1}, for M1b and M1c, respectively.

Regarding the diradical electronic states, triplet and singlet states (structure A in Figure 3), the electron density of the unpaired electrons is mainly located over C7 and C8 atoms, and the corresponding dihedral angle depends on the relative orientation of the terminal substituents with respect to the main ring (see Table S1), which is associated to the steric effects of all substituents. Thus, for instance, the terminal CH_2 groups in M1c, M1e, M3c, and M5c, have a planar structure (DA = 0, Table S1), and the electronic density of the unpaired electrons lies perpendicular to the molecular plane as the π system of the main ring, facilitating, in the case of the singlet states, the formation of the quinoid form (structure B). For the remaining diradical compounds, the relative orientation of the terminal substituents with respect to the main ring changes between 6° and 89° (see DA values in Table S1 and the diradical M3b in Figure S3), thus preventing the formation of the quinoid form from these structures. Another steric effect refers to all triplet states and diradical singlet states having CF_3 as R_3. Table S1 shows that the triplet states of all compounds having trifluoro methyl substituents in R_3 loss the planarity of the main ring between 15° and 21°. We have estimated the effect of this loss of planarity taking the structure of the triplet M1c and modifying the structure of the main ring as in M2d (PA 19°, see Table S1) with a destabilization energy of 7.35 kcal·mol^{-1}, or modifying the structure of the main ring and the terminal substituents as M7d (PA 19° and DA 75°, see Table S1) with a destabilization energy of 22.42 kcal·mol^{-1}. Thus, these results point out the importance of steric effects in determining the ΔE_{ST} energy gap. Moreover, Tables S2 and S6 show the existence of further effects of the substituents in the triplet and singlet electronic states. Thus, for instance, comparing the NBO charges of the R3 groups in M1a-M1c with the corresponding NBO charges in 1,2,4,5 R_3-benzene (Table S2) we

show that R3 in M1a and M1d act as EDG with stronger character in the singlet quinoidal state whereas in M1c, M1d, and M1e R_3 act as EWG with stronger character in the triplet electronic state. It is also instructive to compare the bonding features of C1-C7 in M1c (WI=1.72, ε = 0.2973 for the singlet state and WI=1.20, ε = 0.1387 for the triplet state, see Table S2) with the corresponding values of ethene (WI=2.04, ε = 0.3594 for the singlet state and WI=1.03, ε = 0.044 for the triplet state), pointing out a polarization of the double bond and the unpaired electron in M1c. This kind of polarization is also important in other compounds with different substituents in R1, R2, and R3, as shown in Table S2.

The captodative effects originated by the different substituents can be envisaged comparing the bonding features M1 with M2 to M7 compounds as displayed in Tables S2 and S8. Thus, for instance, looking at the NBO charges of R3 groups, they have, in the case of the triplet states EWG character in M1a (-0.239 e), and in M4a (-0.091 e) and EDG character in M5a (0.319 e), and in the case of the singlet quinoidal states, EWG character in M1a (-0.267 e) and EDG character in M4a (0.108 e) and in M5a (0.118 e), the inductive effect being different in the triplet and quinoidal singlet electronic states. Looking, for instance at M5a – M5e, our results show that R1+R2 act as EWG when R3 = NH_2 and act as EDG for R3 = CH_3, H, CF_3, and CN in both, quinoidal singlet and triplet states but with different amount. In addition, the geometric configurations of the different substituents originate further interactions that play a very important role in the relative stability of the of the diradical-quinoidal electronic states such as hydrogen bond interactions and lone pair repulsion. The NBO and AIM analyses show that, in general, the singlet triplet energy gap diminishes as R1 and R1 have more electron withdrawing character, although, in most cases, R1 + R2 act as EDG groups, being larger in the quinoidal singlet states than in the diradical triplet states

In the case of M7, the size of t-butyl substituents in R1 and R2, results in a large boat angle for the quinoid singlet states producing a destabilization effect whereas in the triplet states, the t-butyl substituents adopt a perpendicular conformation respect to the main ring, leading the diradical states to lie lower in energy in the cases of M1a, M1b, and M1e. In M1d the singlet quinoid state lies lower in energy than the diradical by about 6 $kcal \cdot mol^{-1}$ because the loss of planarity of the main ring in the triplet state causing a destabilization effect. Beyond the steric effects, the different conformation in the

quinoidal singlet and diradical triplet electronic states originate distinct electronic interactions as pointed out above. Figure S4 displays the AIM critical points and bond paths for compounds M7a and M7d (quinoidal singlet and triplet electronic states). The analyses of Figure S4 and Table S8 show that, along the bonds linking the different atoms in each molecule there are two N···H hydrogen bonds in M7a and four F···H bonds in M7d and several repulsive or destabilizing interactions (with very small values of the electron density ρ, Laplacian of the density $\nabla^2\rho$, and small but positive energy density H)[1–3] originating up to 41ring critical points and up to 8 cage critical points (see Table S6), all of them contributing to the ΔE_{ST} energy gap. Finally, it is worth mentioning that when R3 = H, no quinoidal structure were found and the singlet electronic states has a diradical electronic structure as the triplet electronic state.

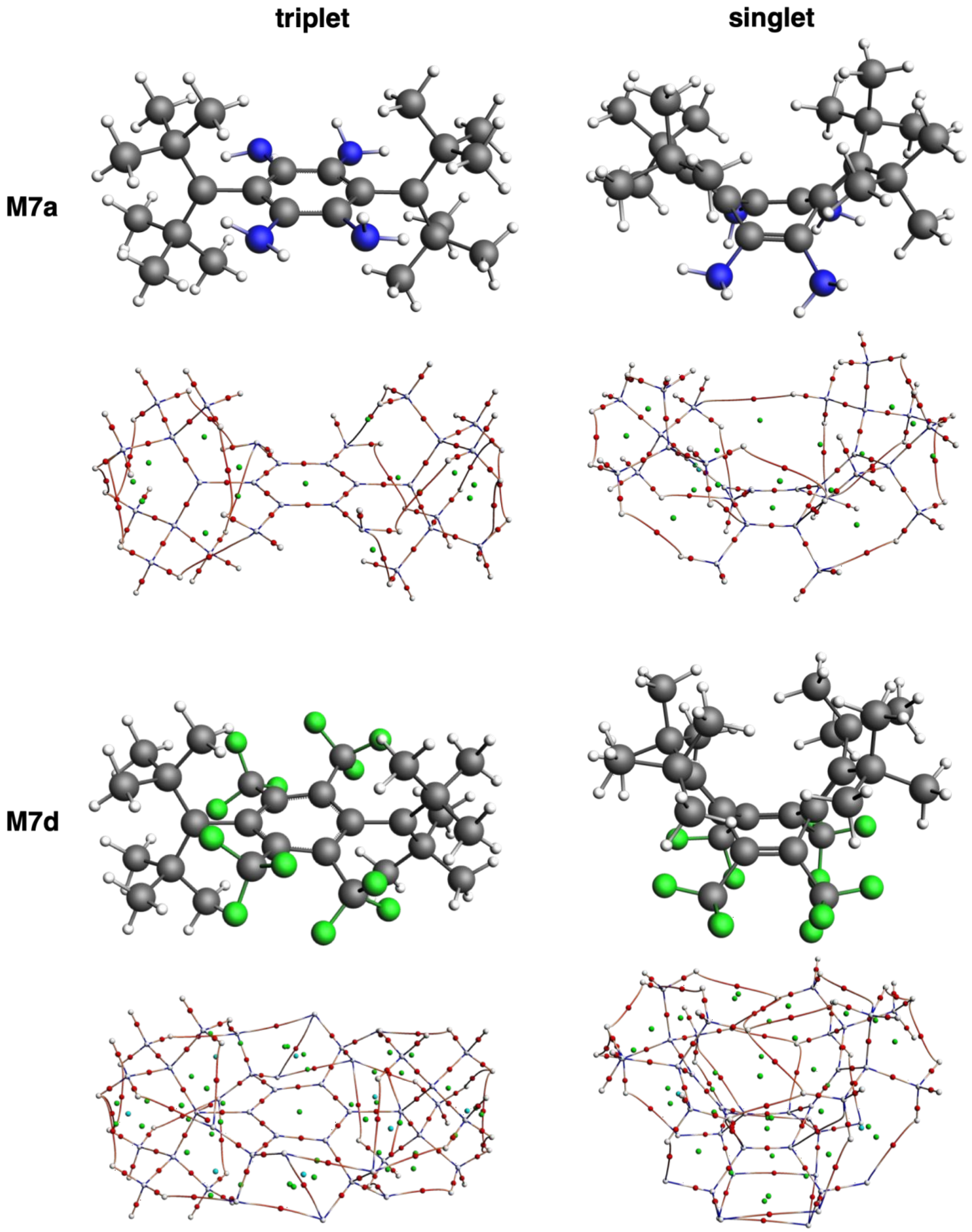

Figure S4. AIM critical points and paths connecting the bond critical points for the triplet and quinoidal singlet states of M7a and M7d.

After an exhaustive search, we have found two conformers of singlet multiplicity for 16 compounds out of 35 investigated, indicating that a potential energy surface connecting both conformers should exist in these cases, and their relative energy is displayed in Figure S5.

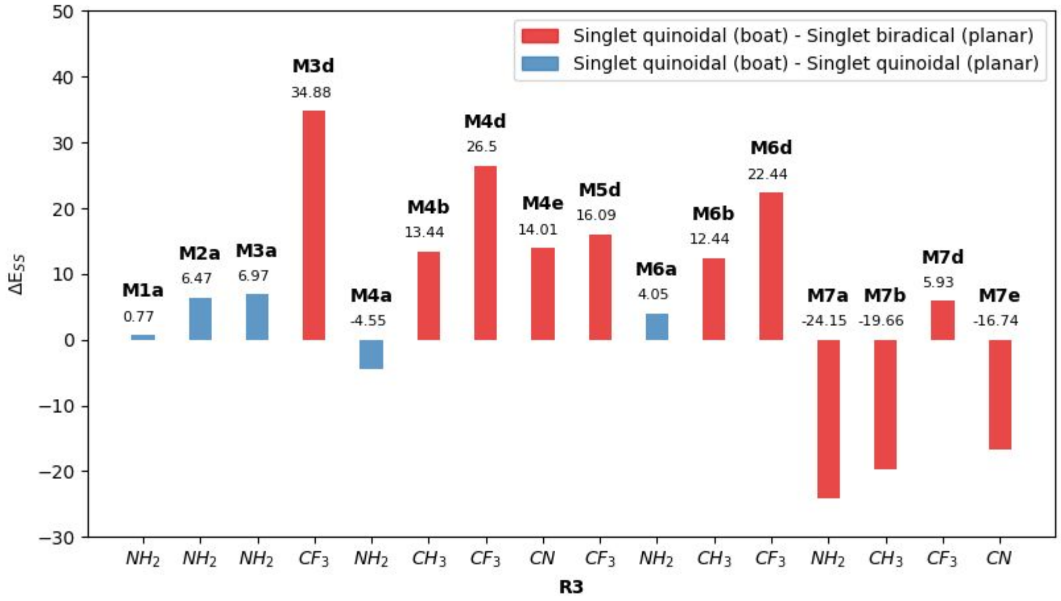

Figure S5. Relative energies (in kcal·mol^{-1}) between different conformers of singlet electronic states.

For compounds **M1a**, **M2a**, **M3a**, **M4a**, and **M6a** (all with NH_2 as R_3) we have found two conformers (boat and planar) with quinoidal structure, which differ at most in 7 kcal·mol^{-1}. In all cases but **M4a**, the lowest conformer has boat structure, but in **M1a**, the two conformers are almost degenerate. The different relative stability can be due to the repulsion of the NH_2 (R3) groups, which is minimized in the boat conformer. For compounds **M3d**, **M4b**, **M4d**, **M4e**, **M5d**, **M6b**, **M6d, and M7d** we have found a boat conformer, which is the lowest energetically, and a planar conformer with diradical character, lying between 6 and 35 kcal·mol^{-1} higher in energy. For compounds M7a, M7b, and M7e we have found a planar diradical singlet states ling lower in energy of the boat quinoidal singlet states between 17 and 24 kcal·mol^{-1}. In all cases, the diradical conformers with singlet electronic configuration lie close in energy to the corresponding triplet electronic states, both having similar electronic and aromatic features (see Table S1 and Figure 4 of the main text).

S4. Further discussion on Thiele like compounds

Table S3 contains the whole results regarding the Thiele and Thiele like compounds. As pointed out in the main text, the singlet state of **T1**, **T2**, **T4**, **T6**, and **T7** have planar structure and no other conformers (namely with boat structure) have been found. From

them, **T2**, **T6**, and **T7** have different degree of diradical character (BC = 0.86, 0.28, and 0.68, respectively) whereas **T1** and **T4** have quinoidal character.

Table S3. Adiabatic relative energies (in kcal·mol^{-1}), diradical character (BC) of the planar singlet electronic states, MCI aromaticity index, the boat angle (BA),[a] and the planarity angle (PA).[b]

		Singlet							Triplet		
		Planar			Boat						
	R3	E	BC	MCI	E	BC	MCI	BA	E	MCI	PA
**T1**	**H**	0.00	0.12	0.015 (0.063)	--	--	--	--	16.89	0.050 (0.055)	0
**T2**	**NH_2**	0.0	0.86	0.030 (0.054)	--	--	--	--	1.12	0.032 (0.054)	1
**T3**	**CH_3**	-0.30	0.94	0.049 (0.054)	0.00	0.02	0.004 (0.066)	33	-0.38	0.049 (0.054)	2
**T4**	**F**	0.00	0.16	0.013 (0.061)	--	--	--	--	6.21	0.040 (0.055)	0
**T5**	**CF_3**	9.13	0.90	0.046 (0.054)	0.00	0.02	0.002 (0.063)	44	9.59	0.048 (0.054)	17
**T6**	**CN**	0.0	0.28	0.018 (0.057)	--	--	--	--	1.81	0.034 (0.054)	1
**T7**	**BRD**	0.0	0.68	0.038 (0.051) {0.056}	--	--	--	--	1.79	0.045 (0.050) {0.057}	0
**T8**	**ANT**	20.89	0.90	0.021 (0.054) [0.031]	0.00	0.02	0.002 (0.066) [0.059]	37	21.19	0.022 (0.054) [0.030]	

a) Values in parenthesis correspond to the MCI values of the terminal phenyl groups. Values in braces correspond to the MCI values of the phenyl bridges. Values in brackets correspond to the MCI values of the lateral phenyls of the anthracene moiety.
b) PA corresponds to deviation of planarity of the main ring in the triplet electronic state

For **T3**, **T5**, and **T8** we have found two conformers of the singlet states, one with planar structure and diradical character (BC = 0.94, 0.90, and 0.90, respectively) and one with boat structure with quinoidal character. In the case of **T3**, both conformers are almost degenerate whereas in the cases of **T5** and **T8** the conformer with diradical structure is calculated to lie 9.13 and 20.89 kcal·mol^{-1} higher in energy than the quinoidal conformer,

which is the ground state. In all these three cases we have looked for the transition state connecting the two conformers and the corresponding results are displayed in Figure S6, which shows that the energy barriers, relative to the most stable conformers are predicted to be 7.04, 23.44, and 24.13 kcal·mol^{-1}, respectively.

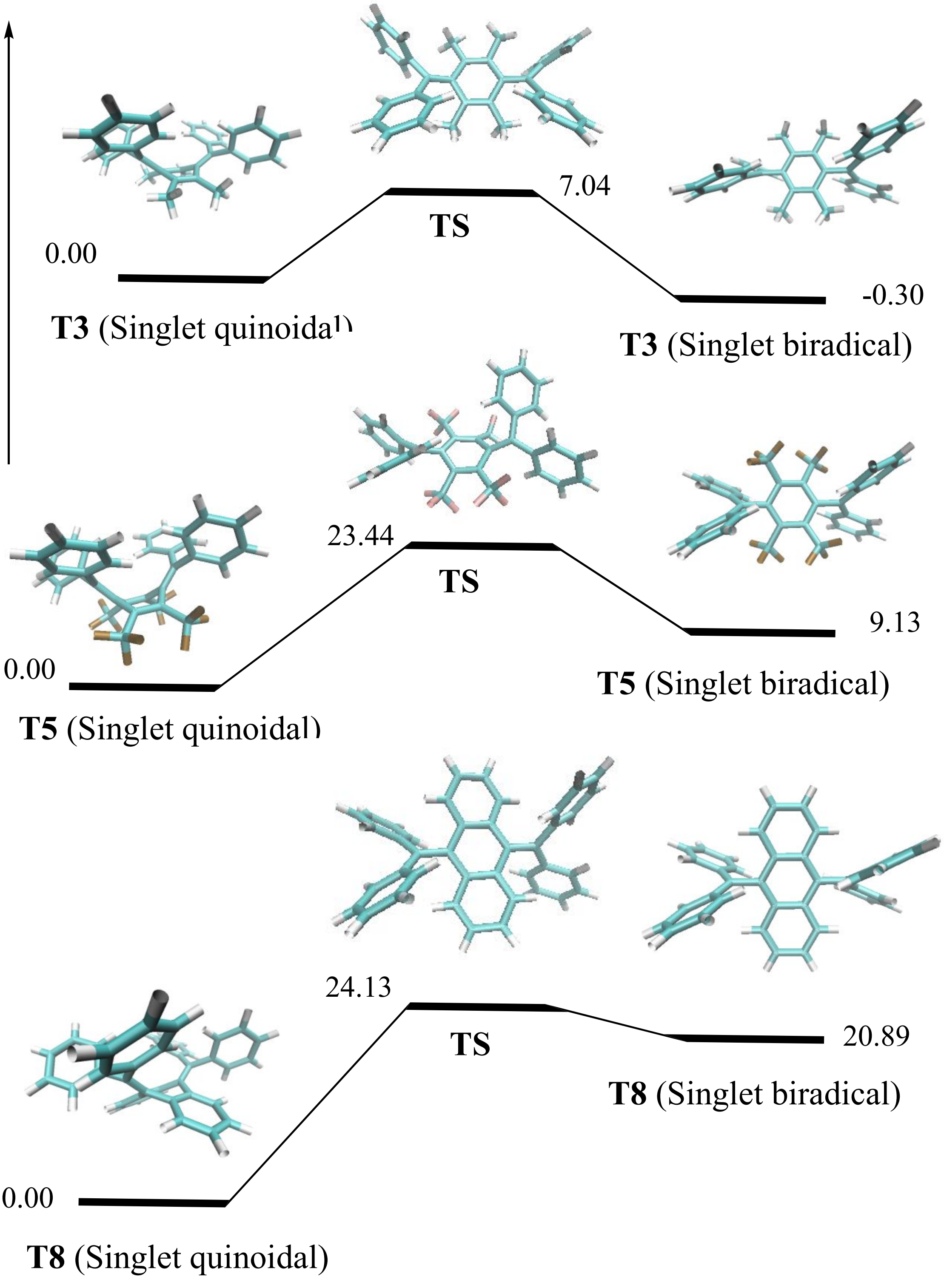

Figure S6. Schematic potential energy surface for the singlet electronic states of **T3**, **T5**, and **T8**. The energies, including the zero-point energy corrections, are in kcal·mol^{-1}.

Finally, the computed absolute energies are displayed in Tables S4, and S5, and the Cartesian coordinates are contained in Tables S6, and Tables S7 for the models and Thiele like compounds, respectively, whereas the results of the AIM analyses are collected in Table S8.

Table S4. Calculated absolute energy values (in hartree) for all model compounds obtained at different levels of theory, zero point energies (ZPE) obtained at B3LYP level or theory, and diradical character (BC) of the singlet electronic states.

	R1	R2	R3	E/B3LYP 6-31+G(d,p)	ZPE	E/DLPNO-CCSD(T) cc-pVTZ	E/FICMR-AQCC def2-SVP	BC
**M1a triplet**	**H**	**H**	**NH2**	-531.03432	0.19589	-530.059391	-529.01531	--
**M1a singlet boat**	**H**	**H**	**NH2**	-531.08567	0.20059	-530.12272	-529.07704	0.08
**M1a singlet planar**	**H**	**H**	**NH2**	-531.08462	0.20065	-530.12156	-529.07579	0.08
**M1b Triplet**	**H**	**H**	**CH3**	-466.85700	0.23913	-465.92341	-465.01432	--
**M1b singlet planar**	**H**	**H**	**CH3**	-466.91108	0.24471	-465.98832	-465.07581	0.08
**M1c triplet**	**H**	**H**	**H**	-309.59310	0.12791	-308.96661	-308.36967	--
**M1c singlet planar**	**H**	**H**	**H**	-309.64285	0.13224	-309.02490	-308.42694	0.10
**M1d triplet**	**H**	**H**	**CF3**	-1657.75635	0.14552	-1655.55263	-1652.38527	--
**M1d singlet boat**	**H**	**H**	**CF3**	-1657.80410	0.14979	-1655.61158	-1652.44317	0.40
**M1e triplet**	**H**	**H**	**CN**	-678.54655	0.12171	-677.31910	-676.02332	--
**M1e singlet planar**	**H**	**H**	**CN**	-678.59342	0.12619	-677.37652	-676.08017	0.10

**M2a triplet**	**NH_2**	**NH_2**	**NH_2**	-752.48197	0.26733	-751.15877		--
**M2a singlet boat**	**NH_2**	**NH_2**	**NH_2**	-752.52670	0.27080	-751.21737		0.02
**M2a singlet boat**	**NH_2**	**NH_2**	**NH_2**	-752.51929	0.27067	-751.20692		0.08
**M2b triplet**	**NH_2**	**NH_2**	**CH_3**	-688.29366	0.30690	-687.00913	-685.65606	--
**M2b singlet boat**	**NH_2**	**NH_2**	**CH_3**	-688.34193	0.31190	-687.07253	-685.71649	0.02
**M2c triplet**	**NH_2**	**NH_2**	**H**	-531.04760	0.19687	-530.06486	-529.02165	--
**M2c singlet planar**	**NH_2**	**NH_2**	**H**	-531.09951	0.20156	-530.12847	-529.08377	0.08
**M2d triplet**	**NH_2**	**NH_2**	**CF_3**	-1879.23769	0.21561	-1876.67091	-1873.06526	--
**M2d singlet boat**	**NH_2**	**NH_2**	**CF_3**	-1879.27751	0.21810	-1876.73223	-1873.12134	0.02
**M2e triplet**	**NH_2**	**NH_2**	**CN**	-900.04042	0.19345	-898.45673	-896.70684	--
**M2e singlet boat**	**NH_2**	**NH_2**	**CN**	-900.06820	0.19436	-898.49598	-896.75362	0.02
**M3a triplet**	**CH_3**	**CH_3**	**NH_2**	-688.30336	0.30811	-687.01982	-685.66916	--
**M3a singlet boat**	**CH_3**	**CH_3**	**NH_2**	-688.32889	0.31143	-687.06188	-685.70460	0.04
**M3a singlet planar**	**CH_3**	**CH_3**	**NH_2**	-688.32400	0.31334	-687.05268	-685.69826	0.12
**M3b triplet**	**CH_3**	**CH_3**	**CH_3**	-624.12697	0.35025	-622.88241	-621.66777	--

**M3b singlet boat**	**CH_3**	**CH_3**	**CH_3**	-624.15817	0.35414	-622.92953	-621.70819	0.04
**M3c triplet**	**CH_3**	**CH_3**	**H**	-466.87776	0.24032	-465.93625	-465.02955	--
**M3c singlet planar**	**CH_3**	**CH_3**	**H**	-466.91907	0.24337	-465.98682	-465.07856	0.10
**M3d triplet**	**CH_3**	**CH_3**	**CF_3**	-1815.03229	0.25731	-1812.52048	-1809.04783	--
**M3d singlet planar**	**CH_3**	**CH_3**	**CF_3**	-1815.03380	0.25753		-1809.04921	0.86
**M3d singlet boat**	**CH_3**	**CH_3**	**CF_3**	-1815.07884	0.26051	-1812.58042	-1809.10178	0.02
**M3e triplet**	**CH_3**	**CH_3**	**CN**	-835.82063	0.23372	-834.28053		--
**M3e singlet boat**	**CH_3**	**CH_3**	**CN**	-835.85691	0.23685	-834.32991		0.04
**M4a triplet**	**CF_3**	**CF_3**	**NH_2**	-1879.23444	0.21492	-1876.67497	-1873.06569	--
**M4a singlet boat**	**CF_3**	**CF_3**	**NH_2**	-1879.25486	0.21826	-1876.69611	-1873.08683	0.02
**M4a singlet planar**	**CF_3**	**CF_3**	**NH_2**	-1879.26128	0.21837	-1876.70347	-1873.09453	0.02
**M4b triplet**	**CF_3**	**CF_3**	**CH_3**	-1815.06047	0.25852	-1812.54583	-1809.07111	--
**M4b singlet boat**	**CF_3**	**CF_3**	**CH_3**	-1815.07125	0.26097	-1812.57075	-1809.09241	0.02
**M4b singlet planar**	**CF_3**	**CF_3**	**CH_3**	-1815.06078	0.25876		-1809.07216	1.00
**M4c triplet**	**CF_3**	**CF_3**	**H**	-1657.79695	0.14871	-1655.58368	-1652.41527	--
**M4c singlet planar**	**CF_3**	**CF_3**	**H**	-1657.83290	0.15143	-1655.63046	-1652.45972	0.10

**M4d triplet**	**CF_3**	**CF_3**	**CF_3**	-3005.92587	0.16509	-3002.15272		--
**M4d singlet planar**	**CF_3**	**CF_3**	**CF_3**	-3005.92589	0.16509			1.00
**M4d singlet boat**	**CF_3**	**CF_3**	**CF_3**	-3005.95327	0.16682	-3002.19671		0.02
**M4e triplet**	**CF_3**	**CF_3**	**CN**	-2026.70354	0.14048	-2023.90108	-2020.03977	--
**M4e singlet planar**	**CF_3**	**CF_3**	**CN**	-2026.70368	0.14048		-2020.04007	0.98
**M4e singlet boat**	**CF_3**	**CF_3**	**CN**	-2026.71350	0.14288	-2023.92612	-2020.06255	0.00
**M5a triplet**	**CN**	**CN**	**NH_2**	-900.03333	0.19487	-898.43558		--
**M5a singlet boat**	**CN**	**CN**	**NH_2**	-900.04874	0.19525	-898.46880		0.02
**M5b triplet**	**CN**	**CN**	**CH_3**	-835.83551	0.23575	-834.29674		--
**M5b singlet boat**	**CN**	**CN**	**CH_3**	-835.85570	0.23799	-834.32797		0.04
**M5c triplet**	**CN**	**CN**	**H**	-678.58090	0.12535	-677.34628		--
**M5c singlet planar**	**CN**	**CN**	**H**	-678.60827	0.12756	-677.38089		0.12
**M5d triplet**	**CN**	**CN**	**CF_3**	-2026.69833	0.14104	-2023.89866	-2020.03734	--
**M5d singlet planar**	**CN**	**CN**	**CF_3**	-2026.69843	0.14103		-2020.03831	0.96
**M5d singlet boat**	**CN**	**CN**	**CF_3**	-2026.71172	0.14267	-2023.92692	-2020.06347	0.02
**M5e triplet**	**CN**	**CN**	**CN**	-1047.47287	0.11665	-1045.64405		--

**M5e singlet boat**	**CN**	**CN**	**CN**	-1047.48426	0.11887	-1045.66655		0.08
**M6a triplet**	**CH_3**	**Phe**	**NH_2**	-1071.82130	0.41577	-1069.79177	-1067.70791	--
**M6a singlet boat**	**CH_3**	**Phe**	**NH_2**	-1071.83400	0.41866	-1069.81187	-1067.73302	0.1-0
**M6a singlet planar**	**CH_3**	**Phe**	**NH_2**	-1071.82803	0.41828	-1069.80504	-1067.72354	0.22
**M6b triplet**	**CH_3**	**Phe**	**CH_3**	-1007.64294	0.45793	-1005.65307	-1003.70587	--
**M6b singlet planar**	**CH_3**	**Phe**	**CH_3**	-1007.64389	0.45847		-1003.70781	0.86
**M6b singlet boat**	**CH_3**	**Phe**	**CH_3**	-1007.65186	0.46010	-1005.67645	-1003.72900	0.02
**M6c triplet**	**CH_3**	**Phe**	**H**	-850.38933	0.34769	-848.70083	-847.06133	--
**M6c singlet planar**	**CH_3**	**Phe**	**H**	-850.41840	0.35023	-848.73652	-847.10062	0.10
**M6d triplet**	**CH_3**	**Phe**	**CF_3**	-2198.53963	0.36409	-2195.28363		--
**M6d singlet planar**	**CH_3**	**Phe**	**CF_3**	-2198.54058	0.36420			0.88
**M6d singlet boat**	**CH_3**	**Phe**	**CF_3**	-2198.56552	0.36635	-2195.32248		0.04
**M6e triplet**	**CH_3**	**Phe**	**CN**	-1219.32995	0.34063	-1217.04782		--
**M6e singlet boat**	**CH_3**	**Phe**	**CN**	-1219.34722	0.34301	-1217.07098		0.06
**M7a triplet**	**t-butyl**	**t-butyl**	**NH_2**	-1160.05331	0.648122	-1157.87835		
**M7a singlet boat**	**t-butyl**	**t-butyl**	**NH_2**	-1159.99596	0.65008	-1157.84186		

**M7a singlet planar**	**t-butyl**	**t-butyl**	**NH_2**	-1160.05336	0.64811	--		0.98
**M7b triplet**	**t-butyl**	**t-butyl**	**CH_3**	-1095.87678	0.69234	-1093.74098		
**M7b singlet boat**	**t-butyl**	**t-butyl**	**CH_3**	-1095.82554	0.69490	-1093.71225		
**M7b singlet planar**	**t-butyl**	**t-butyl**	**CH_3**	-1095.87679	0.69233	--		1.00
**M7c triplet**	**t-butyl**	**t-butyl**	**H**	-938.62024	0.57996	--		
**M7c singlet planar**	**t-butyl**	**t-butyl**	**H**	-938.62046	0.58004	--		0.96
**M7d triplet**	**t-butyl**	**t-butyl**	**CF_3**	-2286.74561	059918	-2283.34796		
**M7d singlet boat**	**t-butyl**	**t-butyl**	**CF_3**	-2286.738919	0.60190	-2283.36023		
**M7d singlet planar**	**t-butyl**	**t-butyl**	**CF_3**	-2286.74565	0.59913	--		0.98
**M7e triplet**	**t-butyl**	**t-butyl**	**CN**	-1307.57132	057378	-1305.14186		
**M7e singlet boat**	**t-butyl**	**t-butyl**	**CN**	-1307.53129	0.57679	-1305.11829		
**M7e singlet planar**	**t-butyl**	**t-butyl**	**CN**	-1307.57143	0.57379			0.96

Table S5. Calculated absolute energy values (in hartree) for all obtained at different levels of theory, zero point energies (ZPE), obtained at B3LYP level or theory, and diradical character (BC) of the singlet electronic states for all Thiele like compounds.

	R3	E/B3LYP 6-31+G(d,p)	ZPE	E/DLPNO-CCSD(T) cc-pVTZ	BC
**T1 triplet**	H	-1233.89582	0.45489	-1231.45591	--
**T1 singlet quinoidal**	H	-1233.91721	0.45689	-1231.48483	0.12
**T2 triplet**	NH_2	-1455.33629	0.52234	--	--
**T2 singlet diradical**	NH_2	-1455.33834	0.52262	--	0.86
**T3 triplet**	CH_3	-1391.15799	0.56507	-1388.42335	--
**T3 singlet diradical**	CH_3	-1391.15819	0.56513	--	0.94
**T3 singlet quinoidal**	CH_3	-1391.14458	0.56614	-1388.42382	0.02
**T3 singlet TS**	CH_3	-1391.14076	0.56718	-1388.41365	0.12
**T4 triplet**	F	-1630.82842	0.42148	-1627.95840	--
**T4 singlet quinoidal**	F	-1630.83786	0.42293	-1627.96974	0.16
**T5 triplet**	CF_3	-2582.04636	0.47074	-2578.04797	--
**T5 singlet diradical**	CF_3	-2582.04705	0.47070	--	0.90
**T5 singlet quinoidal**	CF_3	-2582.05162	0.47233	-2578.06484	0.02
**T5 singlet TS**	CF_3	-2582.02464	0.47105	-2578.02621	0.14
**T6 triplet**	CN	-1602.83595	0.44686	-1599.80671	--
**T6 singlet diradical**	CN	-1602.84187	0.44774	-1599.81047	0.28
**T7 triplet**	BRD	-1693.51651	0.57421	-1690.22639	--
**T7 singlet diradical**	BRD	-1693.51937	0.57443	-1690.22129	0.68
**T8 triplet**	ANT	-1541.17670	0.54768	-1538.14951	--
**T8 singlet diradical**	ANT	-1541.17729	0.54779	--	0.90
**T8 singlet quinoidal**	ANT	-1541.19959	0.54938	-1538.18498	0.02
**T8 singlet TS**	ANT	-1541.17608	0.54825	-1538.14539	0.34

a) R3 stands for the substituents in the main ring

S5. Cartesian coordinates

Table S6. Cartesian coordinates. (in Å) of all stationary points of the model compounds investigated in this work.

M1a Triplet

C .011745 -.064822 .067433

C	-.005436	.027002	1.504618
C	1.170738	.171084	2.231691
C	2.449611	.212499	1.578453
C	2.474533	.207170	.162304
C	1.258405	.108900	-.583153
N	-1.224223	-.075697	2.183890
N	1.039389	.268926	3.661174
N	3.654374	.350795	-.594664
C	-1.187418	-.305770	-.664500
H	-2.108976	-.563154	-.159542
H	-1.200764	-.393323	-1.743988
C	3.658866	.244978	2.337777
H	3.642023	.223059	3.419335
H	4.638630	.207926	1.878086
H	-1.117582	.205978	3.153992
H	-2.001658	.388495	1.730588
H	1.264302	-.601326	4.139207
H	1.624075	1.001313	4.052555
H	3.796359	-.383480	-1.283177
H	4.491767	.487124	-.046247
N	1.371879	.132226	-1.970642
H	.517036	.316852	-2.476176
H	2.144687	.697330	-2.304804

M1a Singlet planar

C	.004739	-.000297	.039611
C	-.031835	.019924	1.516283
C	1.120132	.101037	2.249378
C	2.433292	.208662	1.617245
C	2.469869	.188417	.140573
C	1.317904	.107296	-.592523
N	-1.260228	-.081935	2.159049
N	.978494	.146273	3.678413
N	3.698271	.290274	-.502179
C	-1.136912	-.132302	-.685978
H	-2.100934	-.304491	-.224216
H	-1.130699	-.098392	-1.768859
C	3.574949	.340706	2.342818
H	3.568759	.306799	3.425699
H	4.538954	.512914	1.881029
H	-1.180418	.146010	3.145510
H	-2.032854	.381275	1.700064
H	1.205452	-.742874	4.119405
H	1.571999	.858272	4.095096
H	3.618454	.062376	-1.488651
H	4.470871	-.173004	-.043215
N	1.459538	.062039	-2.021560
H	.866104	-.650039	-2.438214
H	1.232462	.951146	-2.462571

M1a Singlet boat

C	-.117302	-.472344	.156752
C	.049655	.189173	1.465545
C	1.275039	.252448	2.066092
C	2.471511	-.286551	1.419132
C	2.338008	-.676719	.002107
C	1.107213	-.783589	-.581083
N	-1.080354	.675316	2.120984
N	1.343617	.863597	3.358660
N	3.485865	-1.018374	-.710953
N	1.060636	-1.220547	-1.943373
C	-1.353623	-.751709	-.332347
H	-2.254017	-.551192	.235153
H	-1.490430	-1.246120	-1.287108
C	3.658745	-.408416	2.068312
H	3.768869	-.135613	3.111516
H	4.530688	-.840953	1.593312
H	.998533	-2.232517	-2.035090
H	.288638	-.806057	-2.454973
H	3.302620	-1.059187	-1.709194
H	4.305932	-.470423	-.483877
H	1.199209	.203645	4.120173
H	2.225586	1.340836	3.512930
H	-.826483	1.286851	2.891296
H	-1.782611	1.078923	1.514139

M1b Triplet

C	-.004294	-.001573	-.002273
C	-.004248	-.028398	1.425015
C	1.213581	.023393	2.125883
C	2.447728	-.046116	1.411684
C	2.445823	-.094533	-.014616
C	1.231176	.002746	-.716677
C	-1.331389	-.117467	2.152466
C	1.251521	.159995	3.635175
C	3.765501	-.256559	-.742996
C	1.201691	.120506	-2.227771
C	-1.252810	.022316	-.722250
H	-1.289150	-.055284	-1.799759
H	-2.201177	.119192	-.212688
C	3.696354	-.065069	2.131719
H	3.729779	-.128026	3.210270
H	4.647818	-.015440	1.621137
H	3.631577	-.587098	-1.772669
H	4.342838	.677893	-.768893
H	4.390414	-1.007836	-.251562
H	1.996995	.901117	3.938366
H	.297896	.489915	4.046007
H	1.515278	-.784598	4.130327
H	-1.215068	-.419945	3.192684

H	-1.872406	.838675	2.145226
H	-1.982684	-.859440	1.681149
H	2.167381	.413209	-2.639065
H	.908521	-.821124	-2.712058
H	.482532	.882078	-2.543211

M1b Singlet

C	-.145653	.490701	.139967
C	-.026750	.030359	1.539934
C	1.184517	.032059	2.165459
C	2.394343	.493707	1.451774
C	2.275406	.954359	.051882
C	1.064031	.953176	-.573452
C	-1.306222	-.427901	2.205180
C	1.383786	-.423871	3.594339
C	3.555010	1.411739	-.613629
C	.864701	1.409447	-2.002207
C	-1.353955	.489096	-.484493
H	-1.475463	.818828	-1.507400
H	-2.257990	.157891	.008315
C	3.602763	.494513	2.075988
H	3.724334	.164539	3.098816
H	4.506910	.824940	1.582866
H	3.409490	1.740316	-1.640046
H	4.004866	2.247549	-.064744
H	4.296797	.604467	-.631925
H	1.793908	.385566	4.209908
H	.464139	-.757014	4.069680
H	2.095879	-1.256059	3.643749
H	-1.160528	-.757220	3.231343
H	-2.048355	.379049	2.223951
H	-1.755572	-1.263564	1.655725
H	1.783764	1.745516	-2.476634
H	.457111	.599357	-2.618639
H	.150521	2.239837	-2.051689

M1c Triplet

C	.038650	.000107	.025481
C	-.008063	-.000086	1.440272
C	1.156581	-.000187	2.202916
C	2.434774	-.000103	1.594480
C	2.481488	.000090	.179689
C	1.316843	.000193	-.582955
H	-.974541	-.000177	1.937436
H	1.087313	-.000351	3.287557
H	3.447965	.000177	-.317474
H	1.386111	.000362	-1.667595
C	-1.165628	.000218	-.763511
H	-1.122237	-.000386	-1.847333
H	-2.142087	.000924	-.291244

C	3.639052	-.000210	2.383473
H	3.595660	.000202	3.467295
H	4.615511	-.000714	1.911207

M1c Singlet

C	.023676	.000103	.015655
C	-.008180	-.000042	1.477348
C	1.122602	-.000130	2.217829
C	2.449748	-.000109	1.604307
C	2.481604	.000027	.142614
C	1.350822	.000137	-.597868
H	-.980470	-.000016	1.963590
H	1.065666	-.000181	3.303433
H	3.453894	-.000006	-.343629
H	1.407759	.000188	-1.683471
C	-1.111280	.000212	-.727919
H	-1.076836	.000308	-1.813223
H	-2.092634	.000244	-.263185
C	3.584703	-.000201	2.347881
H	3.550259	-.000277	3.433185
H	4.566058	-.000198	1.883148

M1d Triplet

C	-.012133	.005265	-.009042
C	-.000393	-.032971	1.416590
C	1.220065	.031945	2.110652
C	2.436155	-.313636	1.449987
C	2.386685	-.503548	.036967
C	1.238179	-.130237	-.681826
C	-1.329784	-.224295	2.145389
C	1.301043	.540119	3.549074
C	3.572112	-1.188162	-.642044
C	1.295918	.213410	-2.169454
C	-1.239927	.174189	-.741185
H	-1.292014	-.024012	-1.801018
H	-2.143028	.505272	-.251048
C	3.666561	-.465235	2.180918
H	3.672982	-.545194	3.257426
H	4.618708	-.502081	1.673553
F	-1.168474	-.751992	3.374489
F	-2.043181	.920890	2.268067
F	-2.115791	-1.106686	1.476482
F	2.541684	.516013	-2.581510
F	.817639	-.774515	-2.964087
F	.553163	1.320693	-2.428354
F	3.230350	-1.766354	-1.809912
F	4.612633	-.352034	-.873632
F	4.044804	-2.197812	.133180
F	2.361287	1.376204	3.696062
F	.223211	1.266475	3.901310

F	1.454912	-.452857	4.458153

M1d Singlet

C	-.153276	-.337069	.107275
C	-.064684	-.094180	1.573183
C	1.170874	-.125224	2.127545
C	2.337385	-.338082	1.225019
C	2.084911	-1.308297	.124878
C	.839177	-1.323335	-.406766
C	-1.350222	.112473	2.376538
C	1.498753	.073519	3.604132
C	3.202437	-2.262244	-.301753
C	.345993	-2.238050	-1.523178
C	-.975562	.327466	-.727704
H	-1.660156	1.087244	-.378010
H	-.959808	.136453	-1.794262
C	3.471068	.375650	1.366368
H	3.577108	1.101632	2.163961
H	4.304137	.272997	.685230
F	-2.422395	.363710	1.592429
F	-1.659570	-.992122	3.092081
F	-1.253268	1.156982	3.228521
F	.472702	-1.630779	-2.734426
F	.985385	-3.415647	-1.592365
F	-.967389	-2.525031	-1.364050
F	2.969621	-3.515981	.146978
F	3.342096	-2.310537	-1.645295
F	4.410193	-1.906527	.190723
F	1.768420	1.380716	3.867159
F	.521140	-.315199	4.437504
F	2.600321	-.631614	3.953251

M1e Triplet

C	-.013219	-.005676	-.009714
C	.000336	-.000214	1.415790
C	1.211384	.022418	2.144159
C	2.476674	.040837	1.487666
C	2.463119	.035376	.062162
C	1.252071	.012743	-.666207
C	-1.243100	-.017898	2.121647
C	1.171085	.027338	3.573488
C	3.706555	.053060	-.643695
C	-1.237615	-.028581	-.746158
H	-2.189776	-.042567	-.231662
H	-1.229315	-.032424	-1.828488
C	3.701069	.063741	2.224110
H	3.692770	.067584	3.306440
H	4.653231	.077727	1.709614
C	1.292370	.007823	-2.095536
N	-2.269918	-.032772	2.669013

N	1.168991	.031787	4.737173
N	1.294464	.003381	-3.259221
N	4.733374	.067933	-1.191060

M1e Singlet

C	-.024518	-.005904	-.016522
C	.003032	-.000011	1.453416
C	1.176756	.021834	2.159327
C	2.487973	.041089	1.494474
C	2.460423	.035148	.024536
C	1.286699	.013302	-.681375
C	-1.251475	-.017601	2.143970
C	1.155079	.027490	3.591271
C	3.714931	.052689	-.666017
C	-1.184381	-.027587	-.714324
H	-2.143583	-.042019	-.209898
H	-1.187793	-.031642	-1.798147
C	3.647834	.062856	2.192276
H	3.651246	.066950	3.276099
H	4.607035	.077328	1.687849
C	1.308377	.007597	-2.113319
N	-2.290140	-.032349	2.667127
N	1.180061	.032878	4.754060
N	1.283397	.002168	-3.276108
N	4.753596	.067400	-1.189174

M2a Triplet

C	.007391	-.083494	-.004400
C	-.006034	-.051865	1.418467
C	1.193310	-.007347	2.150034
C	2.453166	.037302	1.494639
C	2.467881	.066179	.073831
C	1.268258	.009864	-.658655
N	-1.199842	-.105120	2.175901
N	1.068965	.022106	3.564536
N	3.664750	.208131	-.676780
C	-1.215617	-.204085	-.798117
N	-2.366824	-.839151	-.257100
N	-1.431150	.680728	-1.882870
C	3.680130	.023844	2.296413
N	3.842461	.979975	3.319947
N	4.853837	-.622863	1.813054
H	-1.122112	.450020	3.023009
H	-2.037330	.110319	1.648695
H	.540093	-.766321	3.928016
H	1.960355	.121385	4.035926
H	3.693330	-.431431	-1.467293
H	4.493433	.077934	-.104833
N	1.376356	-.014969	-2.066407
H	.497847	.224058	-2.516838

H	2.117475	.598208	-2.393687
H	5.668783	-.474201	2.397301
H	4.723651	-1.617003	1.647371
H	4.684729	.885994	3.875125
H	3.685610	1.951296	3.051968
H	-2.209748	.421546	-2.479625
H	-1.513615	1.666663	-1.623859
H	-3.085707	-1.016939	-.950921
H	-2.148504	-1.697937	.240477

M2a Singlet

C	-.019004	.069730	-.052364
C	.032458	.214897	1.411565
C	1.233442	.282568	2.047028
C	2.483330	.135305	1.267757
C	2.325662	-.657582	.026129
C	1.115788	-.700957	-.595915
N	-1.190371	.273224	2.140050
N	1.228613	.331542	3.485408
N	3.472909	-1.376678	-.469300
N	.866713	-1.478640	-1.769603
C	-.962526	.701110	-.831810
N	-1.892255	1.608578	-.324752
N	-1.077696	.551142	-2.218526
C	3.663809	.762939	1.600705
N	3.849404	1.551535	2.753950
N	4.821136	.732574	.820508
H	1.590628	-2.169438	-1.943146
H	-.024167	-1.965807	-1.697229
H	3.673624	-2.226175	.055374
H	3.439996	-1.585356	-1.460111
H	2.072547	-.042996	3.902497
H	1.012350	1.237656	3.891098
H	-1.846733	-.430942	1.807922
H	-1.000975	.115299	3.128958
H	5.673530	.682399	1.369828
H	4.777153	.004473	.108865
H	4.460188	2.339599	2.563250
H	2.984996	1.871765	3.167909
H	-1.277640	1.427852	-2.687529
H	-.300451	.052572	-2.642077
H	-2.808943	1.544368	-.753656
H	-1.935829	1.631545	.690174

M2b Triplet

C	-.024472	.037753	.060145
C	-.001734	.093751	1.478228
C	1.214621	.024187	2.174260
C	2.452444	.013768	1.455665
C	2.424727	-.014079	.026510

C	1.195927	-.071941	-.654911
C	-1.303131	.202219	2.247053
C	1.192439	-.118187	3.683516
C	3.706503	.089330	-.776939
C	1.194492	-.252147	-2.162575
C	-1.332689	.082437	-.638184
N	-1.521851	1.029392	-1.688986
N	-2.094341	-1.103717	-.686622
C	3.715943	.025823	2.171072
N	3.933900	.903606	3.241634
N	4.757120	-.847396	1.827434
H	3.536384	.619039	-1.717921
H	4.475471	.626850	-.219805
H	4.134400	-.890680	-1.027321
H	1.179031	.848434	4.204468
H	.308969	-.674303	4.008020
H	2.076691	-.648501	4.040923
H	-1.220627	.908220	3.079918
H	-2.107275	.533456	1.586925
H	-1.612744	-.763742	2.666873
H	1.932462	-1.002551	-2.464570
H	.216513	-.562255	-2.528898
H	1.443925	.675605	-2.694934
H	-2.952013	-1.037565	-1.223632
H	-1.579522	-1.944193	-.953074
H	-2.495401	1.131443	-1.962088
H	-1.151315	1.942487	-1.443843
H	4.756600	.743581	3.807055
H	3.753274	1.885735	3.071956
H	5.660088	-.669037	2.246130
H	4.529966	-1.833864	1.784103

M2b Singlet

C	.040406	.038234	.036622
C	.017720	-.379401	1.462810
C	1.198849	-.377815	2.138643
C	2.425868	.035308	1.409055
C	2.447550	-.382307	-.017118
C	1.266494	-.377615	-.692957
C	-1.255983	-.918808	2.089735
C	1.310658	-.932782	3.550926
C	3.719597	-.926210	-.643641
C	1.153558	-.932864	-2.105048
C	-.919072	.823038	-.546733
N	-.760238	1.466828	-1.782601
N	-2.165069	1.157445	.005976
C	3.387473	.817828	1.991821
N	3.232012	1.461048	3.228438
N	4.633287	1.150293	1.437162
H	3.510870	-1.789841	-1.285121

H	4.229875	-.194036	-1.288847
H	4.430725	-1.259049	.118036
H	.790268	-.323094	4.303711
H	.862888	-1.932933	3.610185
H	2.354061	-1.033543	3.858093
H	-1.049789	-1.780663	2.734339
H	-1.765517	-.183452	2.731881
H	-1.966898	-1.252839	1.328319
H	1.596358	-1.935334	-2.163010
H	.110106	-1.028579	-2.413600
H	1.678068	-.326270	-2.857443
H	-2.386281	2.141088	-.107789
H	-2.313347	.854070	.956519
H	-1.543197	1.357477	-2.419006
H	.127544	1.310306	-2.235694
H	4.016175	1.349910	3.863052
H	2.345090	1.305259	3.683454
H	4.856413	2.133413	1.551972
H	4.778256	.848848	.485449

M2c Triplet

C	.053235	.017627	-.000818
C	.010787	-.002757	1.418725
C	1.170293	-.034673	2.179729
C	2.456031	-.032301	1.576976
C	2.500754	.002118	.162371
C	1.334546	.006196	-.604031
H	-.954061	.023754	1.915356
H	1.098738	-.077612	3.261970
H	3.460428	.041499	-.344232
H	1.418563	-.015331	-1.686425
C	-1.160797	.065571	-.777574
N	-1.096435	.235191	-2.175176
N	-2.369578	-.355144	-.209424
C	3.651675	-.081443	2.384461
N	3.604327	.336562	3.720458
N	4.911394	-.221630	1.766592
H	5.691683	-.252014	2.410825
H	4.966476	-.986424	1.101061
H	4.433616	.144301	4.267531
H	3.271046	1.287226	3.881395
H	-1.996874	.257045	-2.637098
H	-.519132	1.014319	-2.476143
H	-3.199448	-.164053	-.755781
H	-2.384417	-1.304245	.163176

M2c Singlet

C	.009703	.000823	.006115
C	-.002829	.005335	1.462857
C	1.132992	-.000361	2.207049

C	2.463714	.000457	1.613864
C	2.476255	.002572	.157117
C	1.340425	-.002784	-.587065
H	-.952885	-.003859	1.990741
H	1.028310	.012342	3.288809
H	3.426301	-.008848	-.370737
H	1.445144	.007977	-1.668841
C	-1.142863	.000113	-.749032
N	-1.192975	-.260762	-2.122354
N	-2.422221	.259237	-.246456
C	3.616274	-.000578	2.369020
N	3.666020	-.259364	3.742746
N	4.895963	.256164	1.866074
H	5.600335	-.417022	2.151158
H	4.942447	.465607	.879722
H	4.212969	.410222	4.274840
H	2.779408	-.463856	4.179775
H	-1.739182	.408646	-2.655433
H	-.306608	-.466881	-2.559114
H	-3.127434	-.413493	-.530546
H	-2.468414	.470094	.739610

M2d Triplet

C	-.018627	-.012541	.006192
C	-.017678	.014498	1.441052
C	1.218046	.008652	2.152137
C	2.418794	-.432308	1.500132
C	2.365170	-.707199	.093208
C	1.232743	-.197186	-.670333
C	-1.312018	-.162430	2.206627
C	1.290473	.668520	3.513062
C	3.280091	-1.704145	-.541405
C	1.467800	.363260	-2.035950
C	-1.224372	.161268	-.765571
N	-1.427471	-.527367	-1.915283
N	-2.197665	1.043774	-.433006
C	3.654063	-.625869	2.217780
N	3.713032	-1.160326	3.462287
N	4.850037	-.324413	1.654952
F	-1.129566	-.690704	3.435291
F	-2.033783	.998095	2.404701
F	-2.169917	-1.007965	1.566622
F	2.761716	.710022	-2.234468
F	1.167821	-.491712	-3.090887
F	.721277	1.483039	-2.293638
F	2.711109	-2.322103	-1.603760
F	4.473456	-1.187009	-1.033943
F	3.676521	-2.695418	.317082
F	2.430008	1.403083	3.661007
F	.272616	1.527073	3.736358

F	1.283104	-.202288	4.584489
H	5.656531	-.890133	1.886984
H	4.848039	.057078	.719509
H	4.469348	-.885290	4.075420
H	2.853410	-1.423372	3.920664
H	-1.938562	-.084213	-2.668072
H	-.712647	-1.179187	-2.206580
H	-3.151434	.842900	-.704022
H	-2.088754	1.596011	.404531

M2d Singlet

C	.126491	-.008038	-.003744
C	-.115094	-.357372	1.418240
C	.992993	-.563605	2.184438
C	2.293127	-.395673	1.486001
C	2.300706	-1.035520	.147222
C	1.188439	-.846494	-.617357
C	-1.536251	-.694621	1.856355
C	.993432	-1.067846	3.617178
C	3.408564	-2.032381	-.177019
C	.906255	-1.521335	-1.948335
C	-.422153	1.118519	-.586825
N	-.353806	1.437965	-1.922743
N	-1.061997	2.103215	.147002
C	3.309307	.443101	1.904004
N	3.447539	.957597	3.171375
N	4.286423	.916313	1.044849
F	-1.747258	-2.030056	1.921090
F	-1.841115	-.170809	3.073953
F	-2.469272	-.213190	.997664
F	1.420933	-2.753270	-2.084362
F	-.424504	-1.624404	-2.176582
F	1.403697	-.774231	-2.996058
F	3.001961	-3.315118	-.030370
F	3.876784	-1.891877	-1.446365
F	4.484029	-1.890392	.636764
F	.902764	-.020016	4.509307
F	-.000681	-1.915961	3.923799
F	2.153962	-1.698363	3.917947
H	5.232135	.907549	1.408346
H	4.228970	.608669	.084968
H	3.811199	1.899000	3.227644
H	2.761711	.732183	3.875405
H	-.263140	2.422150	-2.134820
H	.146482	.829094	-2.552133
H	-1.920271	2.460380	-.256065
H	-1.129650	1.948936	1.142392

M2e Triplet

C	-.041146	.027917	.012578

C	-.012151	.035335	1.448618
C	1.237480	.010808	2.172492
C	2.497206	.074955	1.485566
C	2.467287	.113435	.050101
C	1.219977	.042229	-.674896
C	-1.194370	.120849	2.222610
C	1.156651	-.135586	3.578298
C	3.643413	.277664	-.720508
C	-1.290460	.007802	-.713007
N	-2.420495	-.533826	-.202787
N	-1.419273	.529577	-1.954774
C	3.746370	.100538	2.210918
N	3.851015	.603229	3.462744
N	4.900692	-.375863	1.690004
C	1.307971	-.068183	-2.083473
H	5.776493	-.022531	2.051682
H	4.933572	-.677754	.726123
H	4.608778	.282848	4.050830
H	3.021349	.890521	3.963101
H	-2.162045	.186072	-2.548878
H	-.604090	.864138	-2.449531
H	-3.312184	-.218482	-.560683
H	-2.440029	-.854338	.755476
N	-2.182502	.164715	2.850071
N	1.105066	-.229638	4.744687
N	1.364304	-.133219	-3.251648
N	4.627803	.387115	-1.345819

M2e Singlet

C	-.033364	-.086341	.046047
C	.039603	-.405865	1.479862
C	1.246883	-.374175	2.145804
C	2.494742	-.040151	1.449142
C	2.436434	-.334118	.009169
C	1.228611	-.347274	-.656439
C	-1.119858	-.855551	2.186352
C	1.282607	-.793369	3.516573
C	3.615778	-.712547	-.706099
C	-1.166210	.415433	-.599330
N	-2.426511	.430802	-.067314
N	-1.119620	1.000136	-1.846420
C	3.603350	.500490	2.105383
N	3.529369	1.059579	3.362752
N	4.861898	.582677	1.575890
C	1.212363	-.740404	-2.035242
H	5.433893	1.367191	1.852934
H	5.082858	.160562	.686552
H	4.316807	.882016	3.974286
H	2.643930	1.044193	3.845636
H	-1.900834	.800124	-2.459000

H	-.235868	1.029342	-2.331777
H	-3.033165	1.194479	-.328438
H	-2.626296	-.016921	.814481
N	-2.103192	-1.192570	2.715352
N	1.341625	-1.089931	4.642103
N	1.167060	-1.016896	-3.166517
N	4.613789	-.990585	-1.241848

M3a Triplet

C	.027539	.035350	.006443
C	.012033	-.038093	1.426684
C	1.206080	-.133680	2.163397
C	2.461217	-.063945	1.520650
C	2.488663	.057299	.114353
C	1.297487	.058839	-.633147
N	-1.190910	.002516	2.160918
N	1.081804	-.139551	3.583986
N	3.707394	.018995	-.624930
C	-1.213847	.084103	-.765372
C	-2.361335	-.851263	-.493489
C	-1.422685	1.069037	-1.884287
C	3.718827	-.118481	2.302549
C	4.083608	-1.330881	3.113778
C	4.681458	1.036549	2.319899
H	-1.631397	.576337	-2.849057
H	-2.309498	1.690043	-1.678092
H	-.570595	1.737343	-2.022574
H	-2.594986	-1.433768	-1.399531
H	-2.148242	-1.556368	.312170
H	-3.293238	-.316658	-.243367
H	4.314260	-1.080292	4.162305
H	3.292921	-2.085781	3.105676
H	4.996565	-1.807869	2.719121
H	5.707362	.731778	2.055203
H	4.375195	1.839726	1.644361
H	4.757075	1.470271	3.331378
H	-1.051593	.439250	3.068345
H	-1.954958	.440901	1.662700
H	.563919	-.942104	3.933570
H	1.976242	-.084750	4.054596
H	3.834540	.832651	-1.221839
H	4.520218	-.094495	-.032520
N	1.424409	.062660	-2.037472
H	2.270716	-.418356	-2.330671
H	.614788	-.306983	-2.519062

M3a Singlet planar

C	-.029824	.029976	-.030367
C	.006238	-.276407	1.408777
C	1.179397	-.377225	2.121460

C	2.489139	-.072530	1.537340
C	2.454954	.278219	.114194
C	1.294521	.271156	-.625108
N	-1.195170	-.328937	2.133984
N	1.010766	-.455677	3.545154
N	3.661685	.307004	-.662424
C	-1.202867	.087626	-.759662
C	-2.481432	-.639285	-.394207
C	-1.384208	.890559	-2.031932
C	3.666342	-.111074	2.271702
C	3.905907	-1.010885	3.467832
C	4.884666	.744186	1.985398
H	-1.506034	.253373	-2.921754
H	-2.324968	1.451988	-1.945245
H	-.580099	1.599418	-2.221931
H	-2.861578	-1.130554	-1.300748
H	-2.351284	-1.398222	.375465
H	-3.282533	.046767	-.077361
H	3.956899	-.471234	4.427697
H	3.178204	-1.817594	3.559701
H	4.893034	-1.481459	3.349591
H	5.744825	.177866	1.592226
H	4.682194	1.580567	1.315771
H	5.232785	1.172452	2.936813
H	-1.004056	-.141270	3.117433
H	-1.914936	.282218	1.769561
H	.853161	-1.399636	3.887828
H	1.790366	-.051780	4.046377
H	3.949127	1.241921	-.939321
H	4.432502	-.146086	-.190662
N	1.418787	.357386	-2.021645
H	2.370361	.111864	-2.292067
H	.734165	-.194361	-2.523196

M3a Singlet boat

C	.054666	.242468	.112902
C	-.004594	.301584	1.601808
C	1.158853	.283003	2.305961
C	2.448340	.128449	1.579320
C	2.419907	.890221	.303074
C	1.268110	.922567	-.417145
N	-1.259712	.387191	2.249098
N	1.161344	.435912	3.724202
N	3.560819	1.587198	-.144875
C	-.855395	-.394966	-.684303
C	-2.093020	-1.114613	-.197044
C	-.676058	-.550141	-2.183309
C	3.495830	-.626010	2.016678
C	3.449683	-1.464663	3.276509
C	4.787065	-.806057	1.249446

H	.358610	-.444472	-2.509894
H	-1.017151	-1.547250	-2.485589
H	-1.290161	.163966	-2.752497
H	-2.045473	-2.168124	-.505403
H	-2.223272	-1.071318	.881181
H	-2.992964	-.700008	-.676344
H	4.158208	-1.092685	4.031846
H	2.460797	-1.517749	3.731013
H	3.772082	-2.487018	3.040877
H	4.787117	-.318772	.275681
H	5.643102	-.425981	1.826654
H	4.975486	-1.877840	1.101837
H	-1.231899	.325052	3.257760
H	-1.862141	1.137655	1.929988
H	2.075104	.706078	4.070090
H	.488592	1.138738	4.024063
H	3.287682	2.346690	-.766247
H	4.141305	1.928418	.613566
N	1.230790	1.745714	-1.587565
H	1.753935	1.373230	-2.376344
H	.285448	1.943863	-1.890320

M3b Triplet

C	-.007901	-.005095	-.005898
C	.001776	-.014254	1.410965
C	1.220752	.004251	2.114002
C	2.451631	-.047151	1.414061
C	2.441113	-.078405	-.002403
C	1.223835	-.018523	-.706181
C	-1.297975	-.071713	2.189769
C	1.198785	.106536	3.626500
C	3.737058	-.203496	-.779599
C	1.250508	.059551	-2.220059
C	-1.291660	.020635	-.746537
C	-1.693719	-1.100742	-1.660920
C	-2.249783	1.169350	-.614233
C	3.735426	-.066756	2.154765
C	4.087477	-1.190761	3.086343
C	4.741764	1.038187	2.007892
H	3.637139	-.914530	-1.605971
H	4.052629	.751902	-1.220689
H	4.550406	-.553839	-.141766
H	2.166513	.426119	4.017053
H	.451317	.831487	3.964425
H	.950146	-.850592	4.105050
H	-1.229857	-.777899	3.023569
H	-1.569414	.901667	2.620824
H	-2.126947	-.391339	1.556011
H	2.024552	.751284	-2.567848
H	1.464437	-.913220	-2.683585

H	.295658	.408435	-2.616964
H	5.744444	.657585	1.756199
H	4.451835	1.764765	1.244414
H	4.859320	1.588158	2.957013
H	4.376297	-.828368	4.085797
H	3.267366	-1.903323	3.206921
H	4.958237	-1.752606	2.707997
H	-3.267291	.835470	-.355638
H	-1.927715	1.893771	.138322
H	-2.345682	1.710016	-1.571083
H	-1.975399	-.740002	-2.662949
H	-.903320	-1.846765	-1.777830
H	-2.583054	-1.623110	-1.269314

M3b Singlet

C	.053351	.111857	.003560
C	.017465	.600383	1.416491
C	1.181060	.578588	2.112553
C	2.395718	.069985	1.403939
C	2.451779	.541517	-.014094
C	1.288140	.561049	-.710173
C	-1.237958	1.229614	1.988328
C	1.288467	1.181754	3.499662
C	3.732489	1.111618	-.592066
C	1.205680	1.153475	-2.103592
C	-.884438	-.708011	-.542507
C	-.794933	-1.269679	-1.944841
C	-2.087749	-1.229655	.211682
C	3.297977	-.783136	1.959022
C	3.183466	-1.325429	3.367114
C	4.478138	-1.363207	1.210978
H	3.559509	2.124759	-.978117
H	4.519595	1.180172	.160812
H	4.121015	.521773	-1.432830
H	2.325989	1.247423	3.832178
H	.877076	2.199624	3.506504
H	.728156	.615983	4.255845
H	-1.023209	2.240272	2.359897
H	-2.023587	1.319546	1.236142
H	-1.647914	.667584	2.837877
H	1.657749	2.153816	-2.120543
H	1.743052	.557726	-2.853373
H	.171816	1.257598	-2.437581
H	4.499282	-2.452701	1.341902
H	4.459661	-1.162700	.140881
H	5.430171	-.987309	1.613613
H	3.969698	-.917670	4.019246
H	2.219330	-1.127013	3.833273
H	3.327073	-2.413300	3.357491
H	-2.159348	-2.317661	.086471

H	-2.056681	-1.024950	1.280742
H	-3.022821	-.812494	-.190086
H	-1.567813	-.839755	-2.598744
H	.174090	-1.113667	-2.417033
H	-.979837	-2.351100	-1.922199

M3c Triplet

C	-.009322	.006302	-.006430
C	.000234	-.000261	1.409958
C	1.186524	.005989	2.138691
C	2.454052	.019302	1.506629
C	2.444492	.026006	.090239
C	1.258199	.019738	-.638493
H	-.937970	-.010625	1.954814
H	1.124457	.000370	3.221909
H	3.382697	.036420	-.454614
H	1.320275	.025419	-1.721709
C	-1.250484	-.000203	-.768039
C	-1.215662	.006784	-2.268072
C	-2.571285	-.013653	-.056083
C	3.695194	.025973	2.268228
C	3.660389	.018772	3.768276
C	5.016015	.039353	1.556276
H	-3.403761	-.017098	-.764490
H	-2.685620	-.896954	.591321
H	-2.699361	.862081	.598992
H	-2.224718	-.000120	-2.688325
H	-.698012	.892668	-2.667737
H	-.682624	-.866300	-2.675576
H	4.669447	.026241	4.188508
H	3.126867	.891479	4.175919
H	3.143241	-.867483	4.167793
H	5.848474	.043229	2.264697
H	5.144278	-.836658	.901608
H	5.130152	.922380	.908474

M3c Singlet

C	-.037905	.005548	-.022684
C	.005717	-.003465	1.438644
C	1.160481	.003109	2.146713
C	2.482646	.019858	1.522872
C	2.439020	.028853	.061547
C	1.284257	.022180	-.646525
H	-.925702	-.017359	1.992541
H	1.089592	-.005464	3.228106
H	3.370433	.042561	-.492362
H	1.355141	.030827	-1.727919
C	-1.208753	-.000829	-.740785
C	-1.272897	.005269	-2.247968
C	-2.581015	-.014385	-.114468

C	3.653487	.026399	2.240980
C	3.717620	.019481	3.748153
C	5.025741	.041240	1.614680
H	-3.163977	.852798	-.455569
H	-3.139910	-.903823	-.438446
H	-2.581249	-.003378	.974999
H	-1.835818	-.867239	-2.608673
H	-1.820763	.889677	-2.603194
H	-.302280	-.001907	-2.742972
H	4.269829	-.862511	4.102619
H	4.276208	.894454	4.109662
H	2.746963	.021285	4.243129
H	5.585176	.929107	1.942002
H	5.608192	-.827576	1.952501
H	5.025935	.034441	.525183

M3d Triplet

C	.037271	-.016929	.006081
C	.003149	-.002937	1.425965
C	1.194961	.118090	2.168410
C	2.453102	-.119408	1.554364
C	2.471565	-.310730	.147207
C	1.310331	-.088509	-.619420
C	-1.343273	-.220455	2.123550
C	1.174752	.597652	3.623129
C	3.750301	-.857253	-.495345
C	1.398327	.180509	-2.124815
C	-1.203537	.040031	-.789202
C	-1.594766	-1.077995	-1.709555
C	-2.131860	1.215231	-.706876
C	3.696726	-.164974	2.346314
C	3.905813	-1.194542	3.416779
C	4.805215	.818808	2.114887
H	-2.268811	1.642857	-1.710967
H	-3.130488	.919745	-.354985
H	-1.761569	2.007710	-.053824
H	-1.680774	-.735875	-2.750640
H	-.897889	-1.917727	-1.683466
H	-2.587202	-1.455467	-1.422390
H	4.062753	-.730911	4.401013
H	3.075949	-1.898680	3.500246
H	4.815585	-1.771368	3.194599
H	5.741177	.317186	1.830875
H	4.567940	1.557284	1.346863
H	5.012761	1.360224	3.049681
F	-1.189497	-.807904	3.329861
F	-2.057678	.913618	2.309469
F	-2.131674	-1.065750	1.411679
F	2.597849	.697250	-2.468753
F	1.188335	-.912859	-2.893780

F	.485809	1.108972	-2.509107
F	3.481999	-1.565221	-1.613663
F	4.654765	.094839	-.821499
F	4.380796	-1.726383	.335426
F	2.244394	1.389448	3.891589
F	.091865	1.361804	3.881598
F	1.194756	-.404527	4.532290

M3d Singlet planar

C	.032441	-.017268	.002309
C	.001921	.001034	1.427462
C	1.191993	.111192	2.171465
C	2.457936	-.120239	1.558970
C	2.473984	-.305214	.145456
C	1.312098	-.094849	-.620997
C	-1.337636	-.241139	2.133937
C	1.160364	.615162	3.619493
C	3.742706	-.871603	-.504072
C	1.414055	.193076	-2.123841
C	-1.199490	.041218	-.786736
C	-1.539402	-1.005952	-1.806278
C	-2.196412	1.150234	-.619849
C	3.691563	-.164782	2.346209
C	3.861387	-1.102881	3.504853
C	4.859247	.725821	2.038026
H	-2.349998	1.634892	-1.595139
H	-3.179006	.776146	-.300934
H	-1.874512	1.916979	.085906
H	-1.644535	-.580956	-2.813953
H	-.813015	-1.818271	-1.849458
H	-2.515863	-1.442653	-1.549934
H	4.041008	-.566108	4.446485
H	3.011253	-1.771242	3.646804
H	4.750940	-1.724508	3.326427
H	5.761499	.150928	1.787988
H	4.663026	1.428308	1.227186
H	5.102580	1.310754	2.937173
F	-1.163105	-.838759	3.333020
F	-2.074577	.874229	2.343144
F	-2.116052	-1.094704	1.419557
F	2.616077	.718603	-2.446704
F	1.218601	-.884919	-2.918052
F	.503690	1.127011	-2.502374
F	3.452791	-1.588092	-1.612189
F	4.660687	.058828	-.854215
F	4.368354	-1.742148	.329926
F	2.226991	1.415421	3.878606
F	.074361	1.383849	3.852917
F	1.171277	-.362543	4.554991

M3d Singlet boat

C	.083213	.000179	.041589
C	.012625	-.104315	1.538082
C	1.205996	-.161416	2.173454
C	2.401885	-.077130	1.273386
C	2.257970	-1.045996	.138356
C	1.068300	-.993312	-.504380
C	-1.339124	-.310636	2.213421
C	1.409143	-.531781	3.635517
C	3.309425	-2.130763	-.044624
C	.625217	-1.970035	-1.588935
C	-.542108	.951788	-.696023
C	-1.445958	2.000732	-.099981
C	-.368948	1.092594	-2.186736
C	3.322577	.916257	1.318773
C	3.318289	1.998523	2.369085
C	4.402874	1.088041	.280454
H	-1.312498	.860494	-2.697111
H	-.127893	2.133638	-2.430979
H	.409619	.460967	-2.610258
H	-2.481823	1.828463	-.419297
H	-1.429001	2.041230	.987632
H	-1.161064	2.989220	-.478174
H	3.378684	2.979221	1.882985
H	2.435212	1.991325	3.007054
H	4.202358	1.908838	3.012530
H	5.388107	.886637	.718995
H	4.283410	.443268	-.589663
H	4.416226	2.127921	-.065582
F	-2.314855	-.536851	1.304462
F	-1.337634	-1.370035	3.050395
F	-1.725513	.770750	2.940556
F	1.228119	-1.729165	-2.783011
F	.892130	-3.252635	-1.262725
F	-.707923	-1.894995	-1.804382
F	2.938877	-3.271723	.585583
F	3.536175	-2.432569	-1.342432
F	4.502163	-1.776797	.488374
F	.540787	.089135	4.464172
F	1.268888	-1.867469	3.816062
F	2.653212	-.222017	4.069767

M3e Triplet

C	-.018930	.005010	-.009542
C	.009989	.066749	1.411392
C	1.213863	-.058214	2.144803
C	2.459783	-.325158	1.512028
C	2.424945	-.421299	.092980
C	1.232860	-.226765	-.644412
C	-1.215448	.196811	2.142545

C	1.166616	.156395	3.560727
C	3.609953	-.787317	-.624652
C	1.320005	-.203309	-2.074463
C	-1.255197	.169425	-.768554
C	-1.613722	-.750361	-1.898627
C	-2.229875	1.267365	-.458818
C	3.695981	-.490488	2.270700
C	3.753966	-1.338363	3.507237
C	4.971962	.179961	1.853509
H	-2.506098	1.775280	-1.393199
H	-3.163197	.878128	-.028119
H	-1.833428	2.016883	.229099
H	-1.563074	-.242855	-2.872269
H	-.979643	-1.637599	-1.952876
H	-2.653627	-1.082640	-1.773901
H	3.885971	-.732402	4.414713
H	2.866622	-1.958847	3.647954
H	4.627237	-2.002344	3.445931
H	5.714542	-.543320	1.488099
H	4.831083	.933541	1.076115
H	5.420364	.672633	2.727363
N	4.559565	-1.120292	-1.208706
N	1.402757	-.148331	-3.233719
N	1.128785	.367654	4.704275
N	-2.210571	.265136	2.741550

M3e Singlet

C	-.037177	-.302751	.032374
C	-.095025	-.616684	1.482603
C	1.066002	-.771963	2.195078
C	2.373522	-.624709	1.507631
C	2.292528	-1.115882	.108477
C	1.131392	-.960926	-.604050
C	-1.356293	-.888599	2.102177
C	1.007116	-1.210292	3.556623
C	3.377237	-1.866785	-.446743
C	1.011467	-1.556919	-1.900114
C	-.931093	.507006	-.609312
C	-.845024	.864234	-2.068713
C	-2.086848	1.172620	.099314
C	3.499692	-.087721	2.064294
C	3.567684	.454936	3.466454
C	4.796564	.060400	1.304935
H	-2.340568	2.109728	-.403878
H	-2.979355	.534626	.064061
H	-1.888570	1.394215	1.148620
H	-.817393	1.956212	-2.166556
H	.011223	.453534	-2.597154
H	-1.754342	.530335	-2.585861
H	3.902774	1.498615	3.428452

H	2.635682	.416801	4.024142
H	4.327833	-.092901	4.038984
H	5.425014	-.829369	1.439600
H	4.662661	.203050	.232055
H	5.358717	.913776	1.693969
N	4.262353	-2.491340	-.872977
N	.899611	-2.058600	-2.944711
N	.966684	-1.588656	4.656891
N	-2.390026	-1.124919	2.582503

M4a Triplet

C	.128900	.001996	-.018243
C	.030839	-.034946	1.385299
C	1.195436	-.032404	2.170441
C	2.490960	-.083215	1.540832
C	2.546114	-.093145	.103643
C	1.364116	-.000867	-.670067
N	-1.275292	-.033120	1.951033
N	1.056435	.049459	3.542585
N	3.721876	-.237743	-.584670
C	-1.140941	.047369	-.783082
C	-1.931056	-1.202563	-1.064160
C	-1.695065	1.367984	-1.241404
C	3.702135	-.125299	2.324305
C	3.896543	-1.077688	3.474194
C	4.876696	.779087	2.057032
F	-2.583314	1.246536	-2.248303
F	-2.328892	2.019844	-.223982
F	-.709549	2.196516	-1.665931
F	-1.809237	-1.551637	-2.383172
F	-1.484548	-2.248450	-.346584
F	-3.251102	-1.059515	-.820932
F	3.702072	-.506472	4.710506
F	3.057633	-2.131264	3.405146
F	5.159420	-1.570711	3.495927
F	5.892483	.182132	1.350557
F	4.520870	1.878877	1.361465
F	5.440457	1.204022	3.215634
H	-1.389745	-.607119	2.777689
H	-1.723591	.871201	2.054111
H	1.857127	.306090	4.100606
H	.190698	.455326	3.869301
H	3.605867	-.516609	-1.552361
H	4.542837	-.588465	-.115514
N	1.517514	-.053456	-2.085687
H	.688696	-.378400	-2.570916
H	1.804389	.836838	-2.486829

M4a Singlet planar

C	.051156	.082395	-.052483

C	.040938	-.150226	1.421651
C	1.205862	-.343367	2.181701
C	2.485973	-.121222	1.609603
C	2.496221	.110705	.135426
C	1.331355	.304046	-.624668
N	-1.110030	.000871	2.092051
N	.977642	-.416564	3.591975
N	3.647085	-.041340	-.534889
C	-1.150628	.085511	-.783998
C	-2.284170	-.859852	-.502519
C	-1.400041	.990895	-1.955512
C	3.687804	-.123448	2.341059
C	3.937794	-1.027804	3.513169
C	4.820566	.822690	2.059144
F	-1.145719	.451425	-3.192490
F	-2.697931	1.381618	-1.992819
F	-.659799	2.131057	-1.887257
F	-2.865987	-1.285916	-1.648338
F	-1.878741	-1.967683	.164640
F	-3.313016	-.324686	.248987
F	3.682557	-.487832	4.749760
F	3.198714	-2.168794	3.445466
F	5.236077	-1.417167	3.551089
F	5.850032	.287983	1.308254
F	4.414315	1.929799	1.391112
F	5.401767	1.250120	3.204809
H	-1.033394	-.048095	3.103808
H	-1.960365	.319757	1.654188
H	1.081472	-1.360329	3.954310
H	1.611255	.181319	4.115137
H	3.570548	.007908	-1.546641
H	4.497638	-.359213	-.096743
N	1.559533	.376833	-2.034970
H	.926052	-.221536	-2.557783
H	1.455246	1.320333	-2.397755

M4a Singlet boat

C	.012267	.102611	-.166541
C	.075170	.333248	1.316404
C	1.277666	.453350	2.029672
C	2.506660	.218918	1.355893
C	2.344001	-.552765	.077693
C	1.164945	-.547382	-.683226
N	-1.072900	.401327	2.004750
N	1.117505	.905349	3.377001
N	3.363785	-1.310159	-.350188
N	1.298522	-1.144685	-1.975388
C	-1.148199	.465915	-.868329
C	-1.934491	1.701704	-.520377
C	-1.642712	-.233188	-2.099710

C	3.796330	.564950	1.787932
C	4.189352	.722390	3.227004
C	4.880209	.959471	.820069
F	-1.478728	-1.591626	-2.007290
F	-2.968808	-.041186	-2.282763
F	-1.043226	.134499	-3.273286
F	-3.104106	1.466303	.175806
F	-1.226937	2.576662	.237313
F	-2.304821	2.377772	-1.632532
F	5.522334	.573640	3.398245
F	3.866312	1.921093	3.802667
F	3.609601	-.238165	4.014916
F	5.829286	-.016140	.586648
F	4.394358	1.298839	-.400290
F	5.568888	2.036023	1.264757
H	1.400722	-.456544	-2.718001
H	.531438	-1.759459	-2.215069
H	3.213877	-1.791719	-1.231101
H	4.261957	-1.338902	.106047
H	1.659949	.373417	4.045758
H	1.348994	1.889633	3.490872
H	-.979298	.590594	2.997608
H	-1.981350	.372595	1.569400

M4b Triplet

C	.030101	.008264	.011052
C	-.019598	.084909	1.418814
C	1.183081	.077332	2.149524
C	2.408828	-.005079	1.456150
C	2.458532	-.081760	.048397
C	1.255836	-.074185	-.682317
C	-1.334704	.160365	2.162242
C	1.130756	.141105	3.659880
C	3.773636	-.157318	-.695017
C	1.308107	-.138043	-2.192668
C	-1.239543	.015410	-.760709
C	-1.867915	-1.267218	-1.241946
C	-1.957698	1.302384	-1.076607
C	3.678438	-.012131	2.227974
C	4.396711	-1.299021	2.543881
C	4.306507	1.270576	2.709399
F	-1.703073	1.715775	-2.347585
F	-3.302064	1.183221	-.963625
F	-1.567678	2.299775	-.255134
F	-2.824254	-1.705809	-.378499
F	-2.461861	-1.129686	-2.450243
F	-.954653	-2.256018	-1.347202
F	5.741079	-1.179672	2.431373
F	4.141709	-1.712653	3.814744
F	4.007092	-2.296358	1.722159

F	4.900729	1.132980	3.917533
F	5.262556	1.709616	1.845815
F	3.393004	2.259106	2.815049
H	1.899799	.688240	-2.602603
H	1.775738	-1.068553	-2.533906
H	.319209	-.079046	-2.648860
H	3.851108	-1.088201	-1.268172
H	3.871988	.668673	-1.408215
H	4.635672	-.107242	-.029018
H	-1.433160	-.665882	2.875134
H	-1.412064	1.091055	2.735729
H	-2.196744	.110646	1.496223
H	2.119626	.082139	4.116127
H	.663039	1.071562	4.001142
H	.539102	-.685262	4.069713

M4b Singlet planar

C	.028702	.010911	.012396
C	-.020399	.008556	1.422933
C	1.182989	.015507	2.151528
C	2.410292	-.000471	1.454640
C	2.459353	-.006403	.044093
C	1.256113	.012143	-.684515
C	-1.336623	-.012543	2.168733
C	1.133541	.051050	3.663160
C	3.775316	-.043530	-.701546
C	1.305823	.044127	-2.196211
C	-1.240451	.010165	-.757607
C	-1.778839	-1.253623	-1.378309
C	-2.046760	1.269612	-.946582
C	3.679459	-.013583	2.224560
C	4.203995	-1.281735	2.848205
C	4.498983	1.237614	2.411580
F	-1.825223	1.819071	-2.171901
F	-3.379453	1.049722	-.849566
F	-1.721915	2.209104	-.033852
F	-2.724868	-1.827740	-.585728
F	-2.356109	-1.033963	-2.582662
F	-.807367	-2.174519	-1.553846
F	5.146193	-1.866247	2.058722
F	4.780555	-1.065989	4.053669
F	3.223152	-2.192802	3.022761
F	4.280410	1.793221	3.634641
F	5.829388	1.002948	2.318489
F	4.186629	2.177820	1.495173
H	1.987574	.823772	-2.550591
H	1.663513	-.908774	-2.603980
H	.329813	.246426	-2.639877
H	3.780410	-.842462	-1.450166
H	3.958616	.898194	-1.232360

H	4.624596	-.219190	-.039877
H	-1.350138	-.809458	2.919387
H	-1.509697	.932414	2.697230
H	-2.187876	-.180761	1.507673
H	2.111994	.241451	4.106670
H	.461980	.840236	4.015955
H	.763300	-.896272	4.072706

M4b Singlet boat

C	.047241	.003086	.021417
C	-.002548	.083832	1.520977
C	1.190009	.111034	2.155190
C	2.376745	.003876	1.234396
C	2.238196	.975574	.096742
C	1.036050	1.002303	-.519234
C	-1.324163	.318378	2.223088
C	1.327453	.357819	3.638164
C	3.337215	1.981013	-.178349
C	.672762	2.027194	-1.566343
C	-.633747	-.897812	-.733398
C	-.614777	-.852067	-2.257386
C	-1.426039	-2.067551	-.173648
C	3.380325	-.901605	1.367815
C	3.509167	-1.788933	2.601142
C	4.431870	-1.185235	.307970
H	2.989724	2.988120	.085452
H	4.230574	1.778641	.413365
H	3.628697	1.996989	-1.231915
H	2.261433	.874170	3.869885
H	.499357	.978445	3.990237
H	1.314095	-.569003	4.219809
H	-1.342476	1.331326	2.645159
H	-2.169147	.235474	1.538846
H	-1.486040	-.385481	3.043764
H	1.221333	2.954909	-1.383919
H	.917193	1.694339	-2.579781
H	-.395274	2.253782	-1.546803
F	4.402282	-2.486136	-.067664
F	4.269930	-.471680	-.824195
F	5.686627	-.925520	.762136
F	3.876179	-1.062433	3.693697
F	2.347276	-2.412199	2.913937
F	4.442073	-2.750147	2.464899
F	-.962945	-3.244108	-.658617
F	-1.364308	-2.173998	1.168651
F	-2.742607	-1.985912	-.503757
F	-1.307710	.223878	-2.725087
F	.641961	-.759542	-2.756183
F	-1.181074	-1.932812	-2.826303

M4c Triplet

C	.006670	.012182	.010419
C	-.000441	.008410	1.423569
C	1.185828	.009314	2.145449
C	2.438077	.025069	1.490549
C	2.445195	.033636	.077479
C	1.259041	.022031	-.644436
H	-.939031	-.011041	1.964207
H	1.136947	.014447	3.227676
H	3.383921	.033801	-.463257
H	1.307758	.037332	-1.726546
C	-1.237481	.001676	-.751023
C	-1.316051	-.655519	-2.106030
C	-2.494105	.643725	-.220062
C	3.682581	.027820	2.251779
C	3.771455	-.640894	3.600383
C	4.928387	.694214	1.725462
F	-3.264814	1.144757	-1.207049
F	-3.256761	-.241529	.479382
F	-2.214260	1.666288	.621253
F	-2.553013	-1.116185	-2.379530
F	-.973932	.205171	-3.105290
F	-.470757	-1.709279	-2.195736
F	5.016494	-1.079549	3.872715
F	3.409779	.203164	4.607098
F	2.946889	-1.711995	3.677528
F	5.693204	1.197429	2.715751
F	5.703151	-.172677	1.016119
F	4.631417	1.720382	.894456

M4c Singlet

C	.006121	-.008458	-.070753
C	-.014501	-.147915	1.386440
C	1.104230	-.129267	2.144673
C	2.438645	.034240	1.570782
C	2.459313	.173347	.113547
C	1.340565	.154908	-.644664
H	-.957227	-.318156	1.886360
H	.998375	-.285591	3.206699
H	3.402053	.343417	-.386386
H	1.446430	.310986	-1.706721
C	-1.155951	-.028250	-.803790
C	-1.250974	-.081672	-2.318185
C	-2.511633	-.001962	-.106823
C	3.600665	.054191	2.303910
C	3.695449	.107828	3.818310
C	4.956505	.027923	1.607180
F	-3.525753	.248331	-.953405
F	-2.786604	-1.180124	.514453
F	-2.564480	.962511	.848584

F	-2.135828	-1.028794	-2.707803
F	-1.664154	1.100247	-2.838291
F	-.085351	-.389824	-2.930962
F	4.108090	-1.074208	4.338617
F	4.580612	1.054659	4.207906
F	2.529878	.416603	4.430860
F	5.970424	-.222758	2.453901
F	5.009486	-.936288	.651504
F	5.231763	1.206256	.986369

M4d Triplet

C	.020101	.001341	.013549
C	.001308	.007917	1.426652
C	1.212770	.006251	2.144993
C	2.421043	-.320191	1.488470
C	2.413663	-.481564	.084457
C	1.255307	-.166538	-.652499
C	-1.354581	-.026924	2.153729
C	1.255893	.396198	3.633083
C	3.669762	-1.039842	-.607737
C	1.312458	.038290	-2.176755
C	-1.245642	.166175	-.763997
C	-1.906236	-1.018478	-1.443689
C	-1.912090	1.521896	-.903198
C	3.684482	-.493932	2.267769
C	3.929852	-1.741846	3.094786
C	4.765429	.570540	2.259593
F	-2.227317	1.782196	-2.187832
F	-3.056782	1.605039	-.192065
F	-1.088934	2.499949	-.466385
F	-1.789528	-.968005	-2.787694
F	-1.342165	-2.173868	-1.028735
F	-3.224229	-1.071302	-1.165036
F	3.864890	-1.498090	4.421105
F	3.007392	-2.686362	2.808353
F	5.150117	-2.259845	2.849158
F	5.854961	.189011	1.559372
F	4.301565	1.710154	1.701812
F	5.180754	.854759	3.510259
F	-1.267251	-.667642	3.332324
F	-1.861482	1.199956	2.367454
F	-2.262282	-.718343	1.425925
F	2.508709	.510593	-2.567996
F	1.048640	-1.088241	-2.861864
F	.407024	.967235	-2.562838
F	3.354951	-1.751309	-1.704156
F	4.541991	-.077177	-.954674
F	4.320029	-1.897883	.212365
F	2.424212	1.013984	3.925288
F	.289645	1.278978	3.940495

F	1.146804	-.664434	4.452016

M4d Singlet planar

C	.020030	.001241	.013545
C	.001260	.007814	1.426677
C	1.212711	.005994	2.145039
C	2.421048	-.320338	1.488518
C	2.413652	-.481641	.084467
C	1.255268	-.166729	-.652486
C	-1.354586	-.027830	2.153822
C	1.255886	.396720	3.632934
C	3.669430	-1.040705	-.607697
C	1.312670	.038864	-2.176639
C	-1.245588	.166227	-.763954
C	-1.904902	-1.017736	-1.446161
C	-1.913466	1.521528	-.900799
C	3.684441	-.493881	2.267715
C	3.928969	-1.740444	3.097075
C	4.766494	.569513	2.257270
F	-2.227808	1.783903	-2.185255
F	-3.058817	1.602261	-.190537
F	-1.091585	2.499571	-.461580
F	-1.788957	-.964344	-2.790068
F	-1.339381	-2.173342	-1.033808
F	-3.222714	-1.072640	-1.166905
F	3.865867	-1.494259	4.422986
F	3.005097	-2.684375	2.813302
F	5.148326	-2.260397	2.850907
F	5.856072	.185504	1.558575
F	4.303810	1.708251	1.696719
F	5.181354	.856292	3.507536
F	-1.266725	-.668517	3.332410
F	-1.862413	1.198626	2.367650
F	-2.261770	-.719949	1.425993
F	2.509201	.510840	-2.567445
F	1.048427	-1.087058	-2.862546
F	.407710	.968548	-2.562155
F	3.354134	-1.751878	-1.704179
F	4.542453	-.078766	-.954571
F	4.318898	-1.899330	.212475
F	2.424008	1.015272	3.924435
F	.289290	1.279242	3.940011
F	1.147438	-.663403	4.452580

M4d Singlet boat

C	.009950	-.018163	-.010841
C	-.040277	-.090789	1.499520
C	1.169945	-.015849	2.080858
C	2.299977	.102532	1.079034
C	2.167096	-1.032677	.088385

C	.959713	-1.091979	-.500391
C	-1.339107	-.528899	2.168751
C	1.481009	-.335405	3.545708
C	3.232512	-2.123993	.081019
C	.422495	-2.265036	-1.324804
C	-.518817	.977919	-.746352
C	-1.361974	2.113767	-.156967
C	-.252198	1.162056	-2.239031
C	3.135575	1.148125	.934402
C	3.084970	2.400166	1.808185
C	4.161819	1.265564	-.197362
F	-1.374728	1.520736	-2.889794
F	.669808	2.131786	-2.429696
F	.222801	.050846	-2.826842
F	-2.673159	1.907768	-.379423
F	-1.180953	2.250781	1.173680
F	-1.032765	3.293049	-.719172
F	2.434893	3.391405	1.158563
F	2.438318	2.202942	2.969567
F	4.324152	2.836971	2.100746
F	5.401463	.981781	.242880
F	3.887361	.440233	-1.229552
F	4.180234	2.518335	-.693567
F	-2.413939	-.175164	1.438417
F	-1.357147	-1.880399	2.259033
F	-1.487941	-.023639	3.401185
F	1.031783	-2.413332	-2.510489
F	.576653	-3.419824	-.638900
F	-.900165	-2.111215	-1.547883
F	2.902021	-3.069167	.993552
F	3.362935	-2.720288	-1.112067
F	4.436782	-1.640862	.442188
F	.999784	.572190	4.408062
F	.954799	-1.535069	3.879664
F	2.815703	-.424585	3.728805

M4e Triplet

C	-.028160	-.008534	-.013402
C	.006753	-.006527	1.394760
C	1.242552	-.017050	2.083387
C	2.458146	-.033836	1.371924
C	2.423201	-.039120	-.036224
C	1.187439	-.024587	-.724873
C	-1.218628	-.008517	2.137522
C	1.255871	.004882	3.516052
C	3.648186	-.074085	-.778820
C	1.174530	-.011172	-2.157646
C	-1.323250	.007926	-.734541
C	-1.907810	-1.270763	-1.295097
C	-2.078770	1.305848	-.923404

C	3.753194	-.044360	2.093235
C	4.306476	-1.333514	2.661505
C	4.539949	1.235940	2.274951
F	-1.814474	1.838871	-2.137193
F	-3.406343	1.130395	-.827125
F	-1.701124	2.212784	.004672
F	-2.826026	-1.786737	-.448128
F	-2.504571	-1.069728	-2.481044
F	-.941605	-2.201748	-1.457114
F	5.210994	-1.877320	1.817195
F	4.908957	-1.139686	3.845739
F	3.317843	-2.239476	2.830073
F	4.291117	1.780355	3.486920
F	5.862775	1.028310	2.176824
F	4.182186	2.147673	1.343722
N	4.645163	-.110677	-1.375560
N	1.158360	.007231	-3.319878
N	-2.216066	-.018436	2.734530
N	1.272640	.030332	4.678142

M4e Singlet planar

C	-.027918	-.007191	-.014410
C	.006131	-.004075	1.394047
C	1.241463	-.015437	2.083593
C	2.457877	-.032419	1.372985
C	2.423818	-.036478	-.035465
C	1.188503	-.022857	-.725030
C	-1.219441	-.008430	2.136640
C	1.252878	.011162	3.516277
C	3.648944	-.073652	-.777870
C	1.177614	-.004647	-2.157849
C	-1.321723	.007463	-.736205
C	-1.891524	-1.267362	-1.320784
C	-2.095780	1.298019	-.900705
C	3.751587	-.044701	2.094949
C	4.290153	-1.329449	2.687362
C	4.556961	1.227536	2.252015
F	-1.856167	1.843339	-2.114193
F	-3.419720	1.106357	-.786175
F	-1.714654	2.202128	.028401
F	-2.803771	-1.807168	-.481834
F	-2.491548	-1.052273	-2.502434
F	-.916397	-2.185760	-1.499017
F	5.188031	-1.897018	1.851217
F	4.896317	-1.121569	3.867147
F	3.292898	-2.222432	2.872263
F	4.333429	1.783781	3.463577
F	5.875656	1.003397	2.135521
F	4.195709	2.136526	1.319761
N	4.645686	-.113717	-1.374754

N	1.163965	.020388	-3.319976
N	-2.216715	-.021915	2.733825
N	1.267244	.043129	4.678224

M4e Singlet boat

C	-.036818	-.052601	-.026227
C	.016559	-.056368	1.475416
C	1.248268	-.062827	2.058018
C	2.424225	-.089106	1.121053
C	2.193635	.921812	.033459
C	.957805	.951570	-.540582
C	-1.173661	.158034	2.233423
C	1.406720	.097771	3.466025
C	3.164698	1.936587	-.219924
C	.594815	1.973789	-1.466276
C	-.780468	-.871782	-.801063
C	-.807751	-.727744	-2.330565
C	-1.622885	-2.034089	-.276285
C	3.459316	-.952434	1.205926
C	3.617105	-1.906673	2.399930
C	4.539346	-1.120733	.137784
F	4.467453	-2.360715	-.389889
F	4.410083	-.259417	-.886431
F	5.764770	-.953413	.667426
F	4.085001	-1.234031	3.469500
F	2.433610	-2.466026	2.739148
F	4.471495	-2.906908	2.143705
F	-1.152837	-3.197398	-.772550
F	-1.590641	-2.148712	1.063196
F	-2.908096	-1.899115	-.651082
F	-1.566679	.329474	-2.679791
F	.436024	-.535757	-2.825336
F	-1.316323	-1.808678	-2.937486
N	.298708	2.837256	-2.187037
N	3.936820	2.793089	-.372058
N	1.526505	.273443	4.609526
N	-2.142122	.394034	2.832847

M5a Triplet

C	-.036120	-.011682	-.031144
C	.015781	-.276667	1.375316
C	1.256186	-.330782	2.103492
C	2.499320	.049231	1.508957
C	2.447450	.314230	.102524
C	1.207033	.368434	-.625655
N	-1.112390	-.449769	2.126686
N	1.146970	-.680816	3.423832
N	3.575662	.487269	-.648748
C	-1.280478	-.036188	-.761623
C	-1.504304	.672183	-1.967540

C	-2.397157	-.800132	-.343830
C	3.743688	.073652	2.239440
C	3.967669	-.635510	3.444857
C	4.860026	.838290	1.822014
H	-1.088849	-.148841	3.091418
H	-2.026337	-.469435	1.695578
H	.438037	-1.365071	3.657031
H	1.982484	-.759813	3.989759
H	3.552175	.186661	-1.613566
H	4.489555	.507046	-.217531
N	1.316288	.718708	-1.945925
H	.480850	.797670	-2.511974
H	2.025204	1.403027	-2.178982
N	4.148601	-1.222582	4.441525
N	5.778813	1.467278	1.460639
N	-3.316300	-1.428479	.017746
N	-1.685120	1.258655	-2.964583

M5a Singlet

C	-.050047	-.041463	-.021938
C	.067745	.264743	1.430403
C	1.329562	.354104	2.035706
C	2.519063	.024265	1.327770
C	2.327707	-.689850	.035256
C	1.090243	-.644790	-.622702
N	-1.005575	.488541	2.205375
N	1.308027	.728946	3.415305
N	3.318266	-1.374614	-.558521
N	1.060552	-1.306437	-1.889913
C	-1.221154	.243331	-.749519
C	-2.450690	.676842	-.178713
C	-1.284876	.201690	-2.176506
C	3.799460	.344688	1.817838
C	4.017308	1.241289	2.909028
C	5.025192	-.110594	1.255895
H	1.102666	-.677139	-2.687305
H	.247901	-1.902893	-2.007482
H	3.061799	-1.833144	-1.427551
H	4.253656	-1.457762	-.191970
H	1.909613	.150676	3.992952
H	1.554481	1.700657	3.584780
H	-.798051	.671158	3.182573
H	-1.958893	.475934	1.877826
N	4.269406	1.991886	3.765929
N	6.038659	-.496290	.822462
N	-3.473751	1.008416	.276409
N	-1.397586	.225468	-3.337519

M5b Triplet

C	.001071	-.001224	.000868

C	-.001750	-.023149	1.416014
C	1.225575	.015749	2.101576
C	2.429104	-.026269	1.357851
C	2.431287	-.056328	-.057173
C	1.205186	.007880	-.743017
C	-1.291322	-.115447	2.204865
C	1.231174	.129443	3.611691
C	3.718230	-.183735	-.845483
C	1.202075	.113088	-2.253742
C	-1.298008	.012880	-.725220
C	-1.611422	-.971053	-1.684474
C	-2.262576	1.010964	-.480306
C	3.728103	-.039320	2.083949
C	4.017216	-1.024253	3.049800
C	4.717044	.932969	1.832521
H	2.035683	.726922	-2.602319
H	1.294186	-.869462	-2.732826
H	.287411	.573511	-2.629272
H	3.574277	-.816481	-1.724210
H	4.080916	.790034	-1.197072
H	4.516745	-.634375	-.254837
H	-1.162485	-.746648	3.087041
H	-1.630637	.868592	2.551174
H	-2.100380	-.550050	1.616571
H	2.156715	.569698	3.984765
H	.412646	.765243	3.956636
H	1.115216	-.847869	4.096277
N	4.241752	-1.862288	3.835957
N	5.516875	1.760774	1.618577
N	-1.856512	-1.808568	-2.465029
N	-3.041531	1.859832	-.271924

M5b Singlet

C	-.078385	.167058	.051919
C	-.019809	-.010418	1.516392
C	1.213373	-.033988	2.100348
C	2.388595	.119166	1.220043
C	2.208725	.988199	.040362
C	.975660	1.011701	-.543897
C	-1.289173	-.140633	2.327046
C	1.385567	-.190476	3.594093
C	3.353062	1.829336	-.477945
C	.679121	1.879047	-1.746104
C	-1.033434	-.465435	-.723132
C	-1.063688	-.409161	-2.155499
C	-2.007386	-1.378974	-.200992
C	3.571948	-.556363	1.456858
C	3.754141	-1.494075	2.526089
C	4.700076	-.522584	.572631
H	2.989006	2.822416	-.755492

H	4.133364	1.964224	.271841
H	3.821279	1.394004	-1.367149
H	2.368681	.141722	3.928929
H	.638671	.411296	4.119398
H	1.257548	-1.227471	3.922211
H	-1.200441	.434158	3.253062
H	-2.158201	.237856	1.787850
H	-1.500492	-1.178049	2.607235
H	1.156643	2.855511	-1.627593
H	1.056105	1.443436	-2.677510
H	-.390385	2.050229	-1.872465
N	5.635815	-.551385	-.119389
N	3.950785	-2.281984	3.360276
N	-1.123523	-.418780	-3.318112
N	-2.803990	-2.146138	.162617

M5c Triplet

C	.018581	-.000001	.011167
C	-.001672	.000385	1.421870
C	1.184144	.000385	2.143782
C	2.428014	.000000	1.477912
C	2.448268	-.000385	.067208
C	1.262451	-.000385	-.654704
H	-.949111	.000684	1.950791
H	1.149482	.000680	3.228297
H	3.395706	-.000684	-.461713
H	1.297114	-.000680	-1.739219
C	-1.232730	-.000001	-.750816
C	-1.232044	-.000482	-2.161686
C	-2.485964	.000501	-.102787
C	3.679325	.000000	2.239894
C	3.678639	.000454	3.650764
C	4.932559	-.000473	1.591866
N	-3.513656	.000923	.455796
N	-1.207251	-.000885	-3.331108
N	3.653846	.000834	4.820187
N	5.960252	-.000871	1.033282

M5c Singlet

C	.004797	.000083	.002778
C	-.000845	.000467	1.450589
C	1.158215	.000397	2.156185
C	2.441799	-.000075	1.486300
C	2.447441	-.000460	.038490
C	1.288380	-.000389	-.667107
H	-.953288	.000823	1.970183
H	1.134178	.000697	3.240861
H	3.399883	-.000816	-.481104
H	1.312418	-.000688	-1.751783
C	-1.182334	.000145	-.720166

C	-1.195468	-.000403	-2.147996
C	-2.456993	.000411	-.076636
C	3.628930	-.000138	2.209244
C	3.642064	.000398	3.637074
C	4.903588	-.000424	1.565714
N	-3.492810	.000745	.456212
N	-1.197431	-.000889	-3.312831
N	3.644026	.000863	4.801909
N	5.939405	-.000746	1.032866

M5d Triplet

C	.060058	.030524	.027537
C	.001392	-.004581	1.437199
C	1.191562	.111321	2.181209
C	2.432617	-.070542	1.534253
C	2.481022	-.200149	.129640
C	1.311743	.016474	-.624679
C	-1.358990	-.225315	2.125536
C	1.173297	.489018	3.674631
C	3.800519	-.631972	-.537973
C	1.371722	.297708	-2.138143
C	-1.207609	.081856	-.778102
C	-1.594946	-1.014479	-1.568034
C	-2.023792	1.226071	-.753367
C	3.700329	-.125939	2.339524
C	3.951870	-1.208497	3.200151
C	4.651808	.904376	2.244399
F	-1.214694	-.855162	3.303561
F	-2.031128	.920982	2.325724
F	-2.143595	-1.029645	1.369903
F	2.535160	.875356	-2.482133
F	1.200730	-.809755	-2.879802
F	.404102	1.176621	-2.491350
F	3.574925	-1.308281	-1.676656
F	4.611888	.406127	-.802885
F	4.478685	-1.482368	.268606
F	2.245727	1.258247	3.977992
F	.093616	1.228387	3.979083
F	1.206256	-.584983	4.481892
N	5.424501	1.780198	2.170807
N	4.156667	-2.126498	3.896469
N	-1.914655	-1.943273	-2.204182
N	-2.682139	2.193478	-.738578

M5d Singlet planar

C	.059931	.030618	.027287
C	.001231	-.004048	1.437160
C	1.191347	.110909	2.181376
C	2.432761	-.070491	1.534513
C	2.481250	-.199654	.129659

C	1.311901	.016049	-.624807
C	-1.358840	-.226736	2.125780
C	1.172476	.490417	3.674470
C	3.800182	-.633395	-.538136
C	1.372687	.298929	-2.138071
C	-1.207022	.081970	-.778072
C	-1.588320	-1.008780	-1.578881
C	-2.030647	1.220787	-.743644
C	3.699727	-.125907	2.339536
C	3.945876	-1.201485	3.210667
C	4.658075	.897338	2.235063
F	-1.213077	-.857049	3.303427
F	-2.032824	.918162	2.327087
F	-2.142290	-1.032090	1.369890
F	2.536520	.876845	-2.480444
F	1.202113	-.807205	-2.881632
F	.405492	1.178754	-2.490480
F	3.573062	-1.310094	-1.676338
F	4.613134	.403032	-.804236
F	4.477173	-1.484525	.268795
F	2.244605	1.260577	3.976910
F	.092432	1.230030	3.977230
F	1.205170	-.582051	4.483617
N	5.436049	1.767923	2.155813
N	4.147905	-2.114368	3.914422
N	-1.904733	-1.933289	-2.222804
N	-2.694699	2.184133	-.722921

M5d Singlet boat

C	.004793	.010331	.035478
C	.018790	-.026392	1.528507
C	1.245831	-.008256	2.096063
C	2.383195	.016431	1.130358
C	2.200656	-.953218	.009211
C	.977934	-.946459	-.567880
C	-1.292662	-.282931	2.279602
C	1.533527	-.115154	3.599115
C	3.294423	-1.991784	-.264441
C	.515086	-1.892713	-1.683280
C	-.672695	.955478	-.680795
C	-.581914	1.065291	-2.108750
C	-1.422938	2.011579	-.061977
C	3.375449	.954537	1.150330
C	3.473411	1.965555	2.164030
C	4.326715	1.102622	.085232
F	-1.374283	-1.595953	2.591835
F	-1.385808	.431026	3.409548
F	-2.365155	-.000037	1.516945
F	.847582	-3.166670	-1.404694
F	-.829660	-1.847441	-1.799590

F	1.046491	-1.569770	-2.871926
F	2.957542	-3.157968	.330904
F	3.472436	-2.217202	-1.573216
F	4.476214	-1.618147	.260462
F	1.254660	1.025413	4.248333
F	.816341	-1.106359	4.159321
F	2.837322	-.403879	3.800422
N	-.517594	1.206252	-3.260926
N	-1.991344	2.914184	.400616
N	5.076752	1.290969	-.782949
N	3.576492	2.816308	2.949594

M5e Triplet

C	-.001519	.001069	-.000693
C	-.000833	-.004602	1.409966
C	1.218049	.005909	2.129335
C	2.453196	-.001731	1.447862
C	2.452486	-.008266	.037195
C	1.233630	.005022	-.682173
C	-1.235641	-.057362	2.133888
C	1.181106	.061026	3.560126
C	3.687117	-.064807	-.686739
C	1.270665	.059092	-2.113003
C	-1.282858	.003210	-.756632
C	-1.561559	-1.011192	-1.689024
C	-2.230843	1.020067	-.548194
C	3.734514	-.002542	2.203793
C	4.010300	-1.016799	3.137219
C	4.685478	1.011334	1.994374
N	4.239731	-1.887256	3.886057
N	5.452143	1.881631	1.833448
N	-1.793438	-1.881743	-2.437000
N	-2.994933	1.892772	-.388051
N	4.685508	-.135869	-1.277988
N	1.305986	.127925	-3.272927
N	1.145875	.130750	4.720000
N	-2.234274	-.125332	2.725090

M5e Singlet

C	-.068412	.026809	-.029981
C	.018745	-.218826	1.414188
C	1.243621	-.235333	2.045271
C	2.475210	-.007309	1.280667
C	2.285262	.768667	.049658
C	1.060387	.785292	-.581392
C	-1.166484	-.364658	2.200585
C	1.287789	-.398040	3.465150
C	3.341263	1.581570	-.468552
C	.886860	1.615039	-1.732834
C	-1.113441	-.435499	-.808089

C	-1.178321	-.278241	-2.229253
C	-2.181503	-1.245907	-.307006
C	3.705368	-.499543	1.675164
C	3.900063	-1.326441	2.827107
C	4.903729	-.358828	.905231
N	4.146456	-2.038768	3.713936
N	5.914604	-.334178	.328942
N	-1.294270	-.238683	-3.386651
N	-3.061332	-1.943457	-.000570
N	4.155072	2.302713	-.881345
N	.765795	2.349081	-2.626846
N	1.279815	-.468941	4.626030
N	-2.108772	-.422722	2.879883

M6a Triplet

C	.019968	.035032	-.001782
C	.038497	-.033271	1.408465
C	1.256759	-.011058	2.109549
C	2.504578	-.020579	1.422442
C	2.466830	-.001088	-.001301
C	1.247910	.076132	-.697387
N	-1.148306	-.028726	2.190522
N	1.180939	.026865	3.517237
N	3.641224	-.064559	-.779598
N	1.323570	.097340	-2.116472
C	-1.275450	.061793	-.744006
C	-1.638719	-1.097387	-1.548011
C	-1.091558	-2.373492	-1.248038
C	-1.408405	-3.494626	-2.007804
C	-2.267537	-3.388784	-3.108361
C	-2.797782	-2.136909	-3.442484
C	-2.493310	-1.012569	-2.680374
H	-.420233	-2.467518	-.400854
H	-.982124	-4.458049	-1.742204
H	-2.509345	-4.264358	-3.703480
H	-3.443772	-2.034640	-4.310228
H	-2.886831	-.047584	-2.980504
C	-2.116537	1.246407	-.628108
C	-1.538627	2.500961	-.297500
C	-2.317967	3.645311	-.164004
C	-3.706527	3.584094	-.333624
C	-4.304246	2.353792	-.631751
C	-3.529019	1.206904	-.778706
H	-.464417	2.560016	-.156850
H	-1.841286	4.591581	.076374
H	-4.314049	4.477312	-.222553
H	-5.383473	2.285909	-.738598
H	-4.018318	.259003	-.974436
C	3.769875	-.048585	2.147316
C	4.008328	-.970449	3.313966

C	4.926977	.842365	1.780331
H	5.832269	.273441	1.509200
H	4.694412	1.522136	.958583
H	5.217678	1.451900	2.650907
H	4.251994	-.426759	4.242301
H	3.159443	-1.625821	3.517070
H	4.885058	-1.606458	3.112007
H	.416068	.102788	-2.565623
H	1.883488	.870582	-2.466469
H	4.424864	-.485774	-.297419
H	3.469845	-.531410	-1.666279
H	-1.196404	-.815322	2.833072
H	-1.993581	.009541	1.634468
H	2.006589	.411595	3.958020
H	.345078	.515168	3.827702

M6a Singlet planar

C	.049579	.028140	.017811
C	.147497	.358381	1.433361
C	1.353188	.383214	2.108375
C	2.617231	-.022159	1.487313
C	2.500321	-.402973	.077086
C	1.309137	-.331705	-.620135
N	-.986999	.666954	2.231759
N	1.308554	.827841	3.446490
N	3.618038	-.870624	-.645632
N	1.414005	-.623119	-2.006877
C	-1.176057	.055902	-.684399
C	-1.486690	-.907540	-1.765040
C	-1.150204	-2.273829	-1.630472
C	-1.476764	-3.201666	-2.619009
C	-2.152378	-2.796719	-3.774188
C	-2.498417	-1.449164	-3.927366
C	-2.176420	-.521301	-2.938061
H	-.638116	-2.600841	-.730701
H	-1.209173	-4.245925	-2.482699
H	-2.408542	-3.519947	-4.542912
H	-3.019268	-1.119536	-4.822323
H	-2.447692	.521289	-3.072794
C	-2.230731	1.051931	-.386612
C	-1.895901	2.403055	-.140931
C	-2.879977	3.362320	.095853
C	-4.231709	3.005190	.092329
C	-4.586791	1.673605	-.152441
C	-3.604304	.714272	-.393040
H	-.849756	2.693341	-.150594
H	-2.589942	4.393927	.275621
H	-4.998098	3.752954	.274317
H	-5.633390	1.381143	-.153873
H	-3.894795	-.315470	-.577687

C	3.824298	-.042970	2.177097
C	3.957362	-.204192	3.677219
C	5.188115	.090906	1.531922
H	5.748176	-.858527	1.508962
H	5.159962	.503893	.524270
H	5.791921	.764825	2.154681
H	4.245428	.730033	4.186937
H	3.064848	-.603042	4.157778
H	4.783268	-.902183	3.870168
H	.540615	-.596643	-2.512675
H	2.100253	-.030175	-2.467314
H	4.311986	-1.334664	-.077139
H	3.316806	-1.474015	-1.407603
H	-1.054501	.058605	3.044121
H	-1.865581	.675762	1.734338
H	2.161555	1.264529	3.765353
H	.514469	1.449113	3.584081

M6a Singlet boat

C	.151084	.414975	.002372
C	.343888	.989461	1.339688
C	1.562156	.899759	1.975020
C	2.664798	.094735	1.411879
C	2.606540	.054496	-.065925
C	1.400538	.112139	-.710952
N	-.689645	1.585620	2.111024
N	1.683532	1.481421	3.251820
N	3.781611	-.029762	-.819079
N	1.458895	.109148	-2.144338
C	-1.089528	.214546	-.614245
C	-1.282668	-.869938	-1.612935
C	-.685383	-2.136489	-1.431799
C	-.886223	-3.170830	-2.345569
C	-1.698326	-2.976251	-3.468295
C	-2.311275	-1.733923	-3.658332
C	-2.111413	-.700026	-2.742575
H	-.069322	-2.304092	-.553422
H	-.418145	-4.136529	-2.173562
H	-1.857718	-3.782610	-4.178246
H	-2.946586	-1.567297	-4.524111
H	-2.594430	.258364	-2.907706
C	-2.297211	1.040077	-.348604
C	-2.241372	2.448139	-.434557
C	-3.381940	3.232088	-.242514
C	-4.612837	2.630341	.030294
C	-4.691661	1.234276	.105499
C	-3.554382	.451991	-.088809
H	-1.290359	2.922790	-.659167
H	-3.307295	4.313639	-.314796
H	-5.500631	3.238452	.177261

H	-5.643357	.754483	.317398
H	-3.627842	-.629811	-.029944
C	3.578365	-.610565	2.156724
C	3.599283	-.655722	3.672143
C	4.631643	-1.524228	1.565912
H	4.542802	-1.668447	.491709
H	5.641693	-1.142824	1.779977
H	4.571925	-2.503819	2.056799
H	4.391822	-.008474	4.078509
H	2.657219	-.363350	4.132608
H	3.845483	-1.671407	4.001163
H	1.488782	-.830904	-2.533629
H	.647945	.566605	-2.546187
H	3.605227	.254377	-1.781203
H	4.574208	.447255	-.405939
H	-.847288	1.102098	2.992142
H	-1.565899	1.707914	1.626478
H	2.628346	1.766591	3.477922
H	1.029365	2.253714	3.356824

M6b Triplet

C	-.015700	-.007276	-.011789
C	-.016878	-.050609	1.400691
C	1.201163	-.036553	2.106322
C	2.435073	-.045026	1.405620
C	2.427003	-.034674	-.013284
C	1.208649	.016533	-.716925
C	-1.321210	-.108793	2.170952
C	1.170558	.041466	3.620180
C	3.722173	-.132098	-.795854
C	1.227397	.094416	-2.230595
C	-1.313846	.014610	-.761295
C	-1.719852	-1.186194	-1.475692
C	-1.214492	-2.455219	-1.087163
C	-1.572816	-3.618166	-1.762473
C	-2.433825	-3.563913	-2.864785
C	-2.923447	-2.321516	-3.285900
C	-2.577234	-1.156331	-2.608100
H	-.544685	-2.514650	-.235749
H	-1.178388	-4.573732	-1.427628
H	-2.708922	-4.471532	-3.393826
H	-3.570127	-2.259614	-4.157024
H	-2.937627	-.203230	-2.978794
C	-2.104115	1.237287	-.734933
C	-1.477206	2.487951	-.492496
C	-2.209229	3.670691	-.443696
C	-3.598556	3.654321	-.613716
C	-4.244439	2.430328	-.825771
C	-3.516297	1.245707	-.887178
H	-.401208	2.517536	-.356614

H	-1.693842	4.611726	-.271647
H	-4.169530	4.577044	-.569828
H	-5.325545	2.397996	-.930909
H	-4.044638	.307090	-1.012229
C	3.715068	-.062923	2.145328
C	4.058127	-1.168450	3.102444
C	4.735926	1.025862	1.977839
H	3.619229	-.814517	-1.645139
H	4.036808	.837335	-1.205532
H	4.536959	-.504852	-.173233
H	2.126371	.381841	4.021310
H	.402155	.741149	3.963302
H	.944211	-.928371	4.083601
H	-1.266313	-.830461	2.992185
H	-1.576018	.863542	2.612884
H	-2.154007	-.399066	1.529343
H	1.980230	.806981	-2.582629
H	1.466260	-.876439	-2.684583
H	.261417	.407411	-2.628668
H	5.730282	.630693	1.716318
H	4.447582	1.754343	1.216069
H	4.871338	1.575798	2.924131
H	4.328652	-.788950	4.100581
H	3.243253	-1.886839	3.221010
H	4.939427	-1.726867	2.745205

M6b Singlet planar

C	-.013211	-.006529	-.008909
C	-.007072	.038574	1.408085
C	1.211078	.054872	2.110061
C	2.447773	-.043263	1.411692
C	2.431866	-.121958	-.009690
C	1.215584	-.069339	-.713230
C	-1.307578	.051759	2.188952
C	1.180783	.253157	3.614422
C	3.715751	-.336197	-.790425
C	1.241233	-.061895	-2.230000
C	-1.304553	.014028	-.753202
C	-1.676413	-1.150998	-1.545733
C	-1.167088	-2.433340	-1.213933
C	-1.497950	-3.561653	-1.959207
C	-2.338657	-3.455926	-3.073221
C	-2.836907	-2.198133	-3.434225
C	-2.515353	-1.068005	-2.687944
H	-.518204	-2.531631	-.349660
H	-1.101147	-4.530419	-1.667892
H	-2.594370	-4.336356	-3.655334
H	-3.471186	-2.096431	-4.310717
H	-2.883877	-.099581	-3.008282
C	-2.147206	1.200597	-.660382

C	-1.568983	2.466754	-.385668
C	-2.349414	3.614934	-.281095
C	-3.739245	3.544985	-.430875
C	-4.336214	2.303471	-.681203
C	-3.559395	1.154023	-.795709
H	-.492137	2.537399	-.271884
H	-1.871280	4.570789	-.084744
H	-4.347771	4.440445	-.345715
H	-5.416273	2.229548	-.775612
H	-4.047775	.198690	-.954567
C	3.719964	-.064234	2.146085
C	3.981187	-1.039399	3.259150
C	4.839425	.886999	1.830846
H	3.560516	-1.039029	-1.614043
H	4.099166	.592176	-1.234828
H	4.504258	-.746271	-.158303
H	2.135826	.623516	3.988490
H	.415525	.982194	3.896610
H	.950487	-.673218	4.157732
H	-1.245210	-.594329	3.069800
H	-1.563159	1.058809	2.544008
H	-2.144170	-.295359	1.582344
H	2.048790	.572740	-2.607203
H	1.399894	-1.066857	-2.642653
H	.305291	.310919	-2.646403
H	5.778798	.368160	1.582045
H	4.596704	1.565545	1.010268
H	5.064397	1.506377	2.714388
H	4.251986	-.541377	4.203674
H	3.130505	-1.696734	3.451696
H	4.840334	-1.680199	3.001776

M6b Singlet boat

C	.101260	-.741887	.029757
C	.115932	-1.002236	1.499843
C	1.201731	-.598689	2.205138
C	2.277964	.110327	1.453705
C	2.522279	-.503458	.112561
C	1.446012	-.930973	-.597484
C	-.985051	-1.824536	2.139323
C	1.374440	-.964931	3.665646
C	3.946626	-.741034	-.358034
C	1.591892	-1.637937	-1.927030
C	-1.017222	-.344282	-.646149
C	-1.050443	-.131890	-2.133886
C	-1.518773	-1.124174	-3.009867
C	-1.589510	-.892682	-4.385900
C	-1.192993	.339711	-4.912062
C	-.730335	1.338347	-4.050515
C	-.666800	1.105683	-2.674477

H	-1.813801	-2.091349	-2.611608
H	-1.949441	-1.677100	-5.046056
H	-1.244693	.520193	-5.981914
H	-.420888	2.300844	-4.448605
H	-.311782	1.886751	-2.008067
C	-2.275127	.081721	.046307
C	-2.226415	.991725	1.118991
C	-3.393746	1.435225	1.743009
C	-4.641866	.980582	1.309079
C	-4.709099	.089234	.234395
C	-3.541680	-.343194	-.396585
H	-1.262068	1.357354	1.456543
H	-3.327161	2.143201	2.564760
H	-5.550737	1.324150	1.794659
H	-5.673072	-.266450	-.118910
H	-3.615057	-1.022269	-1.239801
C	2.900455	1.236620	1.897951
C	2.596232	1.899695	3.223563
C	3.911673	2.017473	1.086058
H	3.454256	1.830766	3.908191
H	2.414301	2.970887	3.068916
H	1.725239	1.489397	3.731498
H	3.667943	3.086498	1.124653
H	4.924439	1.918725	1.503438
H	3.948016	1.728421	.036649
H	2.561162	-2.139594	-1.995878
H	.814576	-2.394439	-2.059989
H	1.519106	-.952428	-2.778890
H	-.564635	-2.740908	2.575043
H	-1.493676	-1.289796	2.949804
H	-1.744014	-2.120302	1.414865
H	4.102407	-1.802717	-.585597
H	4.197745	-.183493	-1.268949
H	4.670509	-.468912	.411424
H	2.383814	-.747240	4.019634
H	1.197360	-2.037954	3.811742
H	.666196	-.443269	4.323300

M6c Triplet

C	-.022496	.012100	-.012934
C	-.009301	-.244090	1.373178
C	1.173442	-.243705	2.102489
C	2.430658	.019672	1.488523
C	2.404263	.279756	.089102
C	1.216762	.272613	-.632233
H	-.946627	-.452849	1.881010
H	1.127089	-.453453	3.165650
H	3.329042	.491152	-.436713
H	1.241683	.478368	-1.698519
C	-1.290369	.007012	-.790577

C	-1.361435	-.789273	-2.007734
C	-.567755	-1.956575	-2.163446
C	-.621119	-2.720021	-3.325678
C	-1.452113	-2.342608	-4.387445
C	-2.229636	-1.184560	-4.266301
C	-2.190075	-.423608	-3.101564
H	.077387	-2.268124	-1.348783
H	-.012793	-3.616959	-3.404216
H	-1.487724	-2.936242	-5.296096
H	-2.863151	-.866855	-5.090034
H	-2.777059	.486488	-3.040901
C	-2.411976	.799550	-.307674
C	-2.198625	1.968031	.470969
C	-3.263641	2.728904	.943376
C	-4.584257	2.348004	.676167
C	-4.821368	1.188929	-.072686
C	-3.760402	.430377	-.558360
H	-1.182125	2.282498	.682540
H	-3.063761	3.626611	1.522089
H	-5.413988	2.939630	1.051453
H	-5.840848	.868519	-.269675
H	-3.966665	-.480293	-1.110206
C	3.656140	.021364	2.238280
C	3.645286	-.240721	3.718979
C	4.969127	.283672	1.553778
H	4.649106	-.159110	4.143174
H	2.999370	.465968	4.259865
H	3.268314	-1.246656	3.957919
H	5.803932	.203882	2.254463
H	5.157123	-.424472	.733802
H	5.009867	1.288824	1.107615

M6c Singlet

C	.004780	.017081	.001446
C	.061568	.344251	1.419776
C	1.216703	.329844	2.130992
C	2.514622	.019999	1.538198
C	2.453531	-.288995	.112494
C	1.294981	-.305640	-.592912
H	-.866653	.558506	1.937125
H	1.160300	.548493	3.190890
H	3.372436	-.503958	-.420264
H	1.334576	-.519111	-1.654975
C	-1.177135	.011468	-.722652
C	-1.270953	-.600714	-2.075096
C	-.776096	-1.894147	-2.334296
C	-.890502	-2.469421	-3.601222
C	-1.502082	-1.765410	-4.642211
C	-2.008424	-.484332	-4.400176
C	-1.903158	.085418	-3.131399

H	-.317494	-2.457114	-1.527034
H	-.509005	-3.472402	-3.771863
H	-1.590538	-2.212366	-5.628220
H	-2.488228	.071773	-5.200801
H	-2.304155	1.078653	-2.952770
C	-2.430706	.614966	-.197093
C	-2.448781	1.906718	.365292
C	-3.636098	2.472902	.833057
C	-4.836458	1.761704	.750585
C	-4.838989	.482267	.185615
C	-3.654135	-.078778	-.290245
H	-1.525218	2.475503	.413890
H	-3.623879	3.474796	1.253381
H	-5.760352	2.201855	1.114561
H	-5.766275	-.079373	.113774
H	-3.668227	-1.070736	-.731776
C	3.687774	.020052	2.257113
C	3.770959	.334912	3.729536
C	5.037481	-.293362	1.662335
H	4.338125	-.447420	4.252038
H	4.324058	1.271688	3.890365
H	2.806119	.430966	4.226592
H	5.758174	.493029	1.924920
H	5.436168	-1.226532	2.086101
H	5.040468	-.395283	.577580

M6d Triplet

C	-.005304	.003749	-.006116
C	.014355	-.027108	1.411678
C	1.232160	.109033	2.108884
C	2.470842	-.070194	1.436435
C	2.437779	-.212303	.023017
C	1.238101	-.004754	-.688186
C	-1.296067	-.291204	2.170677
C	1.261355	.563167	3.572720
C	3.705333	-.704093	-.685167
C	1.265609	.293190	-2.195968
C	-1.298272	.042381	-.757691
C	-1.648598	-1.095536	-1.597299
C	-1.162822	-2.394927	-1.307531
C	-1.485981	-3.483725	-2.110643
C	-2.296556	-3.315104	-3.238585
C	-2.774306	-2.038205	-3.554583
C	-2.459019	-.946844	-2.750931
H	-.542643	-2.551079	-.431013
H	-1.106840	-4.468854	-1.854753
H	-2.544733	-4.164821	-3.867396
H	-3.384094	-1.889926	-4.441214
H	-2.804729	.040861	-3.034243
C	-2.150910	1.215497	-.614071

C	-1.602007	2.486293	-.311168
C	-2.412379	3.606809	-.159893
C	-3.801048	3.499057	-.293270
C	-4.367515	2.251066	-.576275
C	-3.560621	1.128691	-.736098
H	-.526909	2.595640	-.213571
H	-1.959486	4.569066	.060694
H	-4.433244	4.373136	-.169549
H	-5.445932	2.149235	-.657581
H	-4.020499	.163341	-.915038
C	3.742814	-.109438	2.178232
C	4.005898	-1.150781	3.225258
C	4.830817	.890807	1.922648
F	3.416333	-1.391539	-1.809483
F	4.570342	.281417	-1.019115
F	4.391774	-1.575830	.098837
F	2.322264	1.379307	3.806126
F	.173587	1.295705	3.890097
F	1.347811	-.449760	4.465640
F	-1.055308	-.907819	3.351993
F	-2.019059	.816245	2.427193
F	-2.095996	-1.140646	1.484088
F	2.436799	.873050	-2.551705
F	1.091732	-.790566	-2.977092
F	.309209	1.188252	-2.536885
H	5.762834	.405025	1.601338
H	4.559184	1.637017	1.174122
H	5.061641	1.420805	2.858452
H	4.199292	-.699605	4.208595
H	3.188484	-1.865883	3.332918
H	4.911964	-1.713435	2.956270

M6d Singlet planar

C	-.003701	.002209	-.003758
C	.017793	-.012819	1.416127
C	1.235671	.117758	2.112763
C	2.477075	-.070285	1.441767
C	2.441186	-.223561	.026590
C	1.241763	-.023068	-.685513
C	-1.292370	-.273241	2.178165
C	1.260357	.599228	3.568620
C	3.698279	-.743504	-.681647
C	1.271689	.267173	-2.195462
C	-1.292161	.041724	-.752901
C	-1.635123	-1.081698	-1.616637
C	-1.160478	-2.386645	-1.336594
C	-1.480657	-3.463647	-2.157005
C	-2.278376	-3.275758	-3.290852
C	-2.747828	-1.992810	-3.594959
C	-2.434924	-.913479	-2.774273

H	-.552851	-2.556059	-.453735
H	-1.110320	-4.454235	-1.909496
H	-2.524771	-4.116085	-3.932891
H	-3.349224	-1.830816	-4.484902
H	-2.775162	.079572	-3.045787
C	-2.160945	1.201815	-.591861
C	-1.624605	2.477158	-.287945
C	-2.447360	3.587466	-.126770
C	-3.835143	3.463308	-.253363
C	-4.388693	2.210385	-.540660
C	-3.569375	1.098393	-.709619
H	-.549931	2.597738	-.198543
H	-2.004913	4.554300	.094930
H	-4.477034	4.329315	-.122764
H	-5.466183	2.096975	-.618659
H	-4.017835	.128622	-.894200
C	3.744037	-.105995	2.179809
C	3.985273	-1.089041	3.287245
C	4.867675	.836974	1.865740
F	3.389177	-1.449925	-1.789417
F	4.575452	.220389	-1.045717
F	4.379554	-1.609456	.113852
F	2.324798	1.414575	3.790924
F	.174977	1.345278	3.863804
F	1.334052	-.392782	4.485917
F	-1.049580	-.876363	3.366149
F	-2.023671	.830896	2.424362
F	-2.086171	-1.135891	1.499436
F	2.449980	.829537	-2.555761
F	1.081801	-.814731	-2.975277
F	.327381	1.176138	-2.536447
H	5.779451	.302887	1.564682
H	4.618858	1.557845	1.085763
H	5.122616	1.400387	2.775337
H	4.190925	-.588014	4.243236
H	3.156604	-1.783058	3.435159
H	4.880396	-1.681218	3.046837

M6d Singlet boat

C	-.092373	-.172993	.067845
C	-.072997	-.227067	1.568327
C	1.148876	-.116946	2.139512
C	2.277011	.074244	1.169151
C	2.184780	-.946162	.073049
C	.964500	-1.083510	-.494614
C	-1.340838	-.653228	2.297866
C	1.452764	-.316909	3.617886
C	3.359424	-1.893322	-.141843
C	.576117	-2.172902	-1.488461
C	-.821613	.706626	-.669806

C	-.732986	.741931	-2.169253
C	-1.672733	.086616	-2.978372
C	-1.600680	.187681	-4.369348
C	-.591170	.943845	-4.969483
C	.346743	1.602270	-4.170513
C	.272613	1.506755	-2.779746
H	-2.446557	-.521969	-2.521516
H	-2.329690	-.333874	-4.982597
H	-.533981	1.016820	-6.051423
H	1.136437	2.191088	-4.627996
H	.997348	2.028704	-2.161245
C	-1.652483	1.791516	-.067257
C	-1.160353	2.578501	.989320
C	-1.926149	3.612703	1.530727
C	-3.200718	3.879689	1.028192
C	-3.695160	3.116450	-.034151
C	-2.923984	2.095920	-.587299
H	-.164245	2.397085	1.375432
H	-1.522195	4.209541	2.343140
H	-3.799847	4.679942	1.452254
H	-4.682521	3.320203	-.437908
H	-3.317495	1.524160	-1.420140
C	3.123364	1.133191	1.163445
C	3.141015	2.185925	2.242536
C	4.102046	1.403750	.048054
H	4.088298	2.134391	2.794822
H	3.095979	3.183566	1.790454
H	2.329164	2.104720	2.963276
H	3.992824	2.442810	-.284568
H	5.133086	1.292538	.405195
H	3.971649	.756594	-.818854
F	1.125398	-3.367997	-1.178246
F	-.764053	-2.365394	-1.492637
F	.949074	-1.881243	-2.754638
F	-1.333013	-1.996154	2.501465
F	-1.478021	-.059058	3.502241
F	-2.460014	-.385885	1.594297
F	3.173425	-3.054293	.531282
F	3.558927	-2.194450	-1.444121
F	4.522983	-1.378895	.320719
F	2.781840	-.468141	3.823297
F	.853844	-1.414843	4.124194
F	1.059698	.738906	4.379804

M6e Triplet

C	-.012413	-.003110	-.005312
C	.011260	-.031189	1.405658
C	1.222957	-.058871	2.133248
C	2.496400	-.101309	1.476991
C	2.455793	-.092158	.044416

C	1.235040	-.024993	-.665253
C	-1.233381	-.037538	2.121748
C	1.138749	.071968	3.556450
C	3.652863	-.266033	-.721452
C	1.263271	.029304	-2.099902
C	-1.294498	.048483	-.762730
C	-1.692273	-1.136201	-1.505618
C	-1.245151	-2.425071	-1.115274
C	-1.609223	-3.562625	-1.828261
C	-2.420169	-3.456341	-2.963595
C	-2.856703	-2.192897	-3.379463
C	-2.502735	-1.052207	-2.667053
H	-.626846	-2.533200	-.229431
H	-1.262427	-4.536706	-1.495995
H	-2.698603	-4.344144	-3.523012
H	-3.463262	-2.095382	-4.275101
H	-2.812935	-.079152	-3.030236
C	-2.063791	1.281508	-.709938
C	-1.427423	2.526925	-.471305
C	-2.156288	3.709937	-.406590
C	-3.546785	3.693114	-.562190
C	-4.198757	2.473215	-.777716
C	-3.475319	1.287756	-.851727
H	-.347976	2.565863	-.362303
H	-1.638503	4.649493	-.237405
H	-4.115635	4.616038	-.504778
H	-5.280464	2.444601	-.870969
H	-4.001850	.348112	-.973549
C	3.737757	-.150288	2.210562
C	3.883107	-.914977	3.494775
C	4.976471	.559017	1.744581
H	5.718340	-.136244	1.328277
H	4.784665	1.328255	.995010
H	5.450026	1.037686	2.611595
H	3.915687	-.250747	4.369300
H	3.090006	-1.645436	3.661823
H	4.841158	-1.450520	3.474170
N	1.047862	.219910	4.707246
N	-2.234333	-.072133	2.712518
N	4.607537	-.447287	-1.361939
N	1.299725	.101890	-3.259861

M6e Singlet boat

C	-.031391	-.022757	.009369
C	-.016410	-.009373	1.481077
C	1.151154	.061481	2.203541
C	2.438961	.288866	1.514174
C	2.427808	-.295172	.154606
C	1.259567	-.403981	-.577670
C	-1.236631	-.304886	2.179994

C	1.122449	-.219090	3.606696
C	3.639803	-.807448	-.407047
C	1.403107	-.798145	-1.949573
C	-1.189341	.259880	-.712404
C	-1.476625	-.311821	-2.047153
C	-1.466653	-1.707709	-2.227566
C	-1.818803	-2.265571	-3.455476
C	-2.167893	-1.438438	-4.525501
C	-2.187278	-.049411	-4.357016
C	-1.865190	.509596	-3.122747
H	-1.199744	-2.352322	-1.395301
H	-1.812304	-3.344202	-3.577935
H	-2.427633	-1.872851	-5.486197
H	-2.457587	.596236	-5.187110
H	-1.890609	1.586900	-2.990354
C	-2.219986	1.158214	-.154652
C	-1.854123	2.326354	.547854
C	-2.824977	3.190327	1.049436
C	-4.180349	2.901910	.866917
C	-4.559230	1.753466	.162988
C	-3.591649	.898405	-.356332
H	-.802941	2.567464	.670504
H	-2.524612	4.090040	1.577826
H	-4.937553	3.570327	1.265624
H	-5.611024	1.523488	.023138
H	-3.891488	.005321	-.894033
C	3.481654	1.009179	2.034154
C	3.435634	1.687012	3.379261
C	4.777208	1.248284	1.292289
H	4.156164	1.217922	4.063260
H	3.753098	2.729874	3.262556
H	2.464527	1.682133	3.867480
H	5.294307	2.111154	1.718935
H	5.451602	.387618	1.375564
H	4.633285	1.436325	.226096
N	1.624796	-1.091315	-3.053614
N	4.621931	-1.272735	-.825456
N	-2.205424	-.582503	2.761293
N	1.116348	-.478188	4.742093

Table S7. Cartesian coordinates. (in Å) of all stationary points of the Thiele and Thiele like compounds investigated in this work.

T1 (triplet state)

C	-.017601	-.006771	.000567
C	.184875	.874800	1.085350
C	1.368465	.869425	1.816231
C	2.428848	-.007298	1.498211
C	2.228639	-.885135	.410241

C	1.039366	-.889971	-.311496
H	-.605479	1.565533	1.362096
H	1.484033	1.563455	2.642875
H	3.016988	-1.579535	.136739
H	.926927	-1.579855	-1.142013
C	-1.274029	-.002205	-.772743
C	-1.854652	-1.268813	-1.211797
C	-1.709041	-2.451927	-.443599
C	-2.257372	-3.660613	-.864109
C	-2.959998	-3.739709	-2.072176
C	-3.108911	-2.587249	-2.852532
C	-2.571987	-1.373897	-2.430794
H	-1.177392	-2.406434	.501136
H	-2.140427	-4.545348	-.244322
H	-3.382035	-4.684592	-2.401426
H	-3.639079	-2.636795	-3.799676
H	-2.678048	-.496216	-3.059604
C	-1.915688	1.270696	-1.093865
C	-1.151860	2.445010	-1.313980
C	-1.761336	3.658852	-1.619825
C	-3.155344	3.751904	-1.704567
C	-3.931279	2.608446	-1.481482
C	-3.325772	1.389853	-1.186543
H	-.069454	2.388211	-1.265023
H	-1.146032	4.536435	-1.798161
H	-3.629526	4.700670	-1.937640
H	-5.015104	2.669161	-1.528986
H	-3.944092	.519082	-.995396
C	3.688692	-.005986	2.266783
C	3.645028	.139591	3.719720
C	2.557803	-.364998	4.477835
C	2.513647	-.225771	5.862393
C	3.544003	.433079	6.543152
C	4.622671	.949231	5.815546
C	4.676607	.803036	4.431994
H	1.756490	-.889521	3.968062
H	1.673597	-.637733	6.414807
H	3.505394	.545292	7.622619
H	5.421975	1.476695	6.328887
H	5.509509	1.229212	3.882780
C	4.960354	-.147913	1.561856
C	5.134607	.358944	.248840
C	6.345850	.224882	-.424429
C	7.424998	-.431166	.179137
C	7.273712	-.949884	1.470403
C	6.067955	-.808460	2.151991
H	4.312647	.881468	-.229212
H	6.451144	.638608	-1.423589
H	8.367450	-.539241	-.349489
H	8.097818	-1.475218	1.945247

H	5.962254	-1.235943	3.143496

T1 (Singlet state, quinoidal)

C	-.038022	.005950	-.025694
C	.200280	.904461	1.092979
C	1.348041	.882651	1.820299
C	2.461703	.001662	1.505232
C	2.234297	-.877016	.368666
C	1.064506	-.894869	-.322801
H	-.584513	1.594500	1.380344
H	1.452285	1.573914	2.648509
H	3.017283	-1.570317	.084104
H	.961629	-1.583589	-1.153310
C	-1.222552	.006914	-.752270
C	-1.578623	-1.097589	-1.681630
C	-1.488105	-2.448074	-1.289619
C	-1.852944	-3.476822	-2.159738
C	-2.315708	-3.181512	-3.445084
C	-2.420931	-1.846235	-3.847640
C	-2.067015	-.818383	-2.973959
H	-1.149112	-2.686563	-.286100
H	-1.782974	-4.509464	-1.829273
H	-2.598571	-3.981791	-4.122771
H	-2.781306	-1.604683	-4.843707
H	-2.157878	.215287	-3.293904
C	-2.213390	1.109588	-.648389
C	-1.823377	2.461761	-.724705
C	-2.765593	3.488776	-.649479
C	-4.122515	3.190594	-.495923
C	-4.528944	1.853988	-.430173
C	-3.588241	.827961	-.515406
H	-.774480	2.703441	-.866658
H	-2.439208	4.522702	-.719741
H	-4.855710	3.989722	-.436453
H	-5.581222	1.609872	-.314153
H	-3.914759	-.206535	-.468425
C	3.645853	-.001517	2.232205
C	3.762902	.668826	3.553908
C	2.805451	.469485	4.568456
C	2.938084	1.083099	5.815155
C	4.031236	1.912985	6.079302
C	4.997101	2.113170	5.087755
C	4.869855	1.491058	3.845978
H	1.963668	-.189868	4.380170
H	2.190413	.905089	6.583115
H	4.134219	2.391515	7.048896
H	5.852755	2.753722	5.282501
H	5.628239	1.648792	3.084972
C	4.874346	-.676976	1.738343
C	5.346029	-.482138	.424730

C	6.514206	-1.102719	-.020945
C	7.240353	-1.935406	.835200
C	6.792961	-2.131231	2.145772
C	5.631945	-1.501982	2.594098
H	4.800127	.179076	-.241290
H	6.861173	-.928038	-1.035615
H	8.148622	-2.419667	.488425
H	7.350692	-2.774065	2.821106
H	5.296245	-1.656139	3.615152

T2 (Triplet state)

C	.030952	.042565	-.001039
C	.037810	.131221	1.410827
C	1.227761	-.071808	2.133403
C	2.436562	-.361758	1.459076
C	2.431371	-.444173	.048643
C	1.237254	-.256686	-.673909
N	-1.117584	.444809	2.160096
N	1.140641	.005135	3.541044
N	3.583573	-.786182	-.694001
N	1.327429	-.304047	-2.083002
C	-1.236062	.222539	-.763486
C	-1.770803	-.914912	-1.505774
C	-1.467279	-2.240994	-1.100024
C	-1.949506	-3.341949	-1.801234
C	-2.740379	-3.166887	-2.943361
C	-3.035885	-1.868886	-3.376421
C	-2.563928	-.763045	-2.674018
H	-.856182	-2.389473	-.215655
H	-1.709727	-4.343508	-1.454866
H	-3.112853	-4.027037	-3.491672
H	-3.629270	-1.717637	-4.274061
H	-2.777218	.232835	-3.047203
C	-1.896565	1.521532	-.734937
C	-1.144004	2.700210	-.491896
C	-1.751367	3.950608	-.443953
C	-3.134784	4.076177	-.619045
C	-3.902930	2.926275	-.837311
C	-3.299742	1.672948	-.896158
H	-.070753	2.615493	-.357305
H	-1.143572	4.834081	-.269424
H	-3.608050	5.052636	-.576190
H	-4.980624	3.006301	-.950913
H	-3.919397	.793152	-1.032929
C	3.704519	-.534339	2.222869
C	3.830319	-1.669314	3.128585
C	3.070638	-2.847309	2.904785
C	3.158647	-3.939707	3.761128
C	3.992830	-3.896888	4.884755
C	4.734010	-2.737272	5.141519

C	4.658384	-1.644428	4.282506
H	2.421493	-2.892732	2.036677
H	2.573758	-4.831239	3.552555
H	4.056570	-4.748877	5.555083
H	5.366817	-2.680913	6.023053
H	5.215792	-.744642	4.519967
C	4.766211	.449551	2.038378
C	4.452799	1.769678	1.620323
C	5.447243	2.722287	1.421256
C	6.794756	2.395195	1.616024
C	7.131472	1.093808	2.006919
C	6.140604	.137985	2.215536
H	3.411911	2.035658	1.467804
H	5.171170	3.727332	1.114227
H	7.569827	3.138951	1.456859
H	8.174463	.818891	2.138517
H	6.427244	-.873027	2.484147
H	3.696443	-.204368	-1.519893
H	4.434454	-.777919	-.145570
H	1.872982	-1.097652	-2.408387
H	.427479	-.289760	-2.546306
H	-1.225910	-.152500	2.975014
H	-1.969164	.469329	1.614137
H	2.034789	-.035892	4.013047
H	.601424	.807407	3.854333

T2 (Singlet state, diradical)

C	.017489	.008835	.003738
C	.033175	.130642	1.415429
C	1.220026	-.084907	2.138921
C	2.442724	-.320861	1.463402
C	2.436555	-.390764	.048355
C	1.230882	-.281541	-.667950
N	-1.139098	.358858	2.169573
N	1.144327	.057513	3.542761
N	3.598391	-.680294	-.701524
N	1.318264	-.362138	-2.075496
C	-1.244442	.183942	-.756709
C	-1.753858	-.931576	-1.549292
C	-1.450554	-2.267938	-1.180392
C	-1.908354	-3.347655	-1.929491
C	-2.676551	-3.138740	-3.081314
C	-2.975728	-1.829048	-3.474852
C	-2.526723	-.744545	-2.725353
H	-.863414	-2.441261	-.284396
H	-1.670008	-4.358904	-1.611506
H	-3.031282	-3.982270	-3.666046
H	-3.554480	-1.651740	-4.377247
H	-2.743815	.262512	-3.065401
C	-1.937720	1.467360	-.690587

C	-1.211188	2.660198	-.439386
C	-1.850532	3.893393	-.355682
C	-3.239359	3.985654	-.507798
C	-3.980233	2.820827	-.740142
C	-3.345408	1.584588	-.831629
H	-.133275	2.600800	-.329050
H	-1.263038	4.789560	-.176052
H	-3.737018	4.948471	-.439955
H	-5.060903	2.875056	-.839655
H	-3.941933	.690703	-.980423
C	3.705658	-.491472	2.223580
C	3.819526	-1.603530	3.163263
C	3.069005	-2.790394	2.958539
C	3.147068	-3.858541	3.846979
C	3.966047	-3.782321	4.980087
C	4.702602	-2.614969	5.213486
C	4.634564	-1.545469	4.324050
H	2.436812	-2.862798	2.079575
H	2.570075	-4.758644	3.653412
H	4.024147	-4.616076	5.673450
H	5.326390	-2.533962	6.099487
H	5.189218	-.638452	4.540174
C	4.794620	.456528	2.007849
C	4.515333	1.781360	1.582443
C	5.536288	2.699148	1.355173
C	6.875653	2.330718	1.531477
C	7.177607	1.024470	1.934291
C	6.160096	.103305	2.169962
H	3.480311	2.080100	1.451672
H	5.287483	3.709840	1.043463
H	7.671219	3.047664	1.351643
H	8.213324	.718918	2.055231
H	6.417204	-.912430	2.451175
H	3.668616	-.107211	-1.538047
H	4.454785	-.618049	-.166266
H	1.909073	-1.130952	-2.380359
H	.419680	-.410947	-2.537976
H	-.986522	1.019947	2.926378
H	-1.933212	.646417	1.612344
H	.337850	-.421057	3.934910
H	1.985426	-.229043	4.026858

T3 (Triplet state)

C	.039082	.010419	.042056
C	-.013420	-.015327	1.454415
C	1.179737	.022672	2.202986
C	2.427859	.043892	1.539711
C	2.480671	.048584	.127275
C	1.286676	.053098	-.620937
C	-1.340292	-.098569	2.181521

C	1.101941	.055734	3.715916
C	3.808981	.032757	-.601908
C	1.362301	.119680	-2.132798
C	-1.230335	-.008925	-.754542
C	-1.570873	-1.222705	-1.482090
C	-1.053915	-2.477070	-1.063776
C	-1.347926	-3.650527	-1.751958
C	-2.154565	-3.620850	-2.895396
C	-2.656120	-2.392851	-3.343653
C	-2.373598	-1.217482	-2.653887
H	-.427402	-2.517890	-.178965
H	-.946600	-4.594916	-1.394432
H	-2.379530	-4.536437	-3.434261
H	-3.261932	-2.350075	-4.244733
H	-2.740956	-.274797	-3.044082
C	-2.059618	1.187422	-.761346
C	-1.483820	2.458769	-.501230
C	-2.256261	3.616449	-.483288
C	-3.636901	3.553099	-.704788
C	-4.233256	2.307954	-.937824
C	-3.464325	1.148267	-.967970
H	-.414766	2.525290	-.328048
H	-1.779313	4.574708	-.296233
H	-4.239468	4.456328	-.685382
H	-5.307761	2.239613	-1.084690
H	-3.955374	.192209	-1.111968
C	3.697439	.062241	2.336207
C	4.091427	-1.151533	3.035848
C	3.626031	-2.417128	2.591432
C	3.972221	-3.591997	3.252393
C	4.781429	-3.553480	4.393802
C	5.232203	-2.315510	4.867889
C	4.897440	-1.138237	4.205376
H	2.997666	-2.464495	1.708230
H	3.609763	-4.544408	2.875170
H	5.047159	-4.470404	4.911402
H	5.839027	-2.266994	5.768002
H	5.225790	-.189733	4.615666
C	4.472503	1.294174	2.371571
C	3.840108	2.544132	2.140859
C	4.559589	3.735528	2.150814
C	5.941674	3.729213	2.372042
C	6.593568	2.506988	2.575865
C	5.877647	1.313417	2.577829
H	2.769248	2.566723	1.968130
H	4.039911	4.675457	1.985902
H	6.502949	4.658865	2.374649
H	7.670120	2.483647	2.721765
H	6.411407	.377280	2.698509
H	3.802400	-.693615	-1.421045

H	4.040872	1.010929	-1.043072
H	4.632563	-.225233	.064327
H	2.053369	.345066	4.163087
H	.341960	.765794	4.057649
H	.832852	-.925032	4.129436
H	-1.301121	-.828994	2.995895
H	-1.618676	.865132	2.627627
H	-2.149553	-.390597	1.511705
H	2.096095	.863208	-2.460036
H	1.665838	-.842238	-2.566190
H	.400720	.383953	-2.573804

T3 (Singlet state diradical)

C	.038307	.014714	.041544
C	-.013540	-.011294	1.454794
C	1.179510	.027575	2.203081
C	2.428656	.048296	1.539918
C	2.480805	.053528	.126585
C	1.286886	.058796	-.621333
C	-1.339597	-.101026	2.183368
C	1.100709	.065315	3.716235
C	3.808675	.032580	-.603984
C	1.363419	.130972	-2.133265
C	-1.229596	-.007806	-.754243
C	-1.547937	-1.206744	-1.516355
C	-1.011714	-2.463323	-1.130718
C	-1.285933	-3.622454	-1.850744
C	-2.092133	-3.574949	-2.993863
C	-2.613459	-2.343672	-3.409377
C	-2.350053	-1.182819	-2.688096
H	-.386415	-2.517769	-.245707
H	-.870048	-4.569624	-1.518088
H	-2.302157	-4.479270	-3.557236
H	-3.219795	-2.286619	-4.309312
H	-2.733045	-.235956	-3.052396
C	-2.084878	1.170427	-.728332
C	-1.534709	2.447401	-.442992
C	-2.331588	3.587632	-.394416
C	-3.711691	3.499575	-.610502
C	-4.282527	2.247685	-.869851
C	-3.489114	1.105734	-.930007
H	-.466096	2.532810	-.275122
H	-1.874206	4.551476	-.188194
H	-4.333328	4.388968	-.567532
H	-5.356012	2.159814	-1.013769
H	-3.959969	.142822	-1.094763
C	3.696800	.063551	2.335617
C	4.067546	-1.137229	3.070559
C	3.586226	-2.406850	2.656408
C	3.911095	-3.568884	3.350267

C	4.715010	-3.512043	4.494604
C	5.182320	-2.268894	4.938136
C	4.868292	-1.104719	4.242984
H	2.963009	-2.468092	1.770330
H	3.536759	-4.525562	2.996153
H	4.964550	-4.418834	5.037517
H	5.785936	-2.205744	5.839491
H	5.209630	-.150452	4.628734
C	4.498887	1.278929	2.337797
C	3.892431	2.536243	2.080243
C	4.637489	3.711760	2.059014
C	6.019900	3.680679	2.275935
C	6.645991	2.449993	2.507886
C	5.904480	1.272576	2.540475
H	2.821310	2.577618	1.912207
H	4.137600	4.658614	1.873791
H	6.601086	4.597776	2.254564
H	7.722190	2.407053	2.651928
H	6.417848	.328165	2.683723
H	3.793017	-.683498	-1.431771
H	4.051787	1.013468	-1.032889
H	4.629842	-.243060	.057950
H	2.046907	.372118	4.162584
H	.329466	.764712	4.054115
H	.847693	-.917780	4.134368
H	-1.291871	-.823366	3.004262
H	-1.628470	.863706	2.620431
H	-2.146076	-.408082	1.517139
H	2.106700	.866595	-2.456413
H	1.654087	-.832642	-2.571665
H	.405665	.410018	-2.573173

T3 (Singlet state, quinoidal)

C	-.016873	-.016932	.001279
C	.036928	-.034352	1.489467
C	1.257292	-.011441	2.082718
C	2.460614	.031206	1.205928
C	2.302645	-.795008	-.030556
C	1.077672	-.820320	-.624539
C	-1.228153	-.206472	2.305344
C	1.399988	-.159759	3.583750
C	3.469840	-1.611922	-.543651
C	.781780	-1.669763	-1.843133
C	-.955100	.685896	-.702780
C	-1.029053	.706605	-2.205054
C	-1.945751	-.086101	-2.914359
C	-2.036367	-.009124	-4.306148
C	-1.211739	.866937	-5.017232
C	-.299466	1.666291	-4.323587
C	-.214583	1.589923	-2.931161

H	-2.579612	-.784037	-2.373957
H	-2.748042	-.637754	-4.834305
H	-1.280153	.926210	-6.099601
H	.345977	2.352510	-4.864921
H	.492501	2.218621	-2.397415
C	-1.895082	1.659847	-.058180
C	-1.407368	2.659609	.802526
C	-2.261594	3.608168	1.367890
C	-3.629587	3.580045	1.086009
C	-4.129574	2.601865	.221497
C	-3.271159	1.663393	-.352788
H	-.344956	2.693851	1.019335
H	-1.855918	4.373574	2.023880
H	-4.296107	4.316249	1.525847
H	-5.190195	2.572073	-.012255
H	-3.676559	.921662	-1.033021
C	3.570519	.774655	1.499024
C	3.613891	1.765564	2.623167
C	4.798599	.818410	.632093
H	1.388190	-2.578920	-1.845168
H	-.266034	-1.974098	-1.861863
H	.981544	-1.145267	-2.784038
H	-1.147076	-1.100691	2.936801
H	-1.414648	.640486	2.975876
H	-2.107594	-.323781	1.671954
H	3.124267	-2.517470	-1.047411
H	4.089611	-1.060083	-1.258725
H	4.122677	-1.918942	.275970
H	2.444383	-.201843	3.892495
H	.914188	-1.087278	3.914022
H	.925070	.661063	4.133365
C	4.704535	1.824572	3.510952
C	4.754375	2.775922	4.530632
C	3.726597	3.712635	4.673456
C	2.649806	3.686020	3.783794
C	2.595978	2.724618	2.772924
H	5.520227	1.116539	3.408027
H	5.601478	2.788828	5.210865
H	3.769732	4.459006	5.461344
H	1.852189	4.418759	3.871109
H	1.760215	2.715860	2.081578
C	4.850189	1.699394	-.459702
C	5.998063	1.797722	-1.249966
C	7.123341	1.023669	-.954326
C	7.089487	.150546	.136354
C	5.938428	.051203	.921435
H	3.980138	2.307612	-.690365
H	6.013003	2.480891	-2.094704
H	8.017149	1.100299	-1.566675
H	7.957415	-.458168	.374631

H	5.916702	-.644799	1.755682

T3 (Transition state connecting the two conformers of the singlet states)

C	.172877	.113347	-.160087
C	.084410	.059109	1.310210
C	1.195549	-.178887	2.070834
C	2.504396	-.419886	1.440065
C	2.471199	-.768044	.010007
C	1.318898	-.617227	-.734730
C	-1.284818	.083010	1.966277
C	1.021105	-.392786	3.564142
C	3.757158	-1.091209	-.736508
C	1.300664	-1.086301	-2.175501
C	-.710039	.897358	-.896947
C	-1.006814	.757706	-2.357875
C	-1.790234	-.309239	-2.830953
C	-2.143386	-.400166	-4.179706
C	-1.719970	.577594	-5.083776
C	-.954380	1.654210	-4.624551
C	-.615279	1.749608	-3.273853
H	-2.124502	-1.070311	-2.130773
H	-2.750734	-1.233511	-4.522273
H	-1.990702	.506624	-6.133282
H	-.626478	2.423886	-5.317786
H	-.033048	2.596256	-2.920960
C	-1.411769	2.062014	-.286934
C	-.749806	2.922287	.613412
C	-1.386708	4.044280	1.143909
C	-2.705840	4.341123	.789303
C	-3.374135	3.509462	-.115105
C	-2.732561	2.395529	-.654827
H	.279501	2.711234	.883209
H	-.847250	4.693971	1.827767
H	-3.202578	5.214240	1.202394
H	-4.397575	3.731378	-.404741
H	-3.262836	1.765956	-1.362320
C	3.681065	-.353958	2.204643
C	3.805496	.616021	3.319798
C	4.848551	-1.244946	2.001730
H	2.052173	-1.858696	-2.339887
H	.334980	-1.522536	-2.426666
H	1.479864	-.281369	-2.898534
H	-1.454979	-.856526	2.507018
H	-1.391274	.893121	2.696024
H	-2.085000	.187166	1.235639
H	3.712717	-.732755	-1.766043
H	4.610997	-.604370	-.268710
H	3.983165	-2.163412	-.765356
H	1.902336	-.839186	4.020870
H	.177413	-1.064428	3.755011

H	.809937	.540517	4.099450
C	4.492519	.303687	4.513347
C	4.623218	1.239630	5.537805
C	4.084651	2.523367	5.396543
C	3.415556	2.858263	4.215691
C	3.280840	1.919619	3.192850
H	4.909332	-.690175	4.642217
H	5.144509	.966050	6.451060
H	4.192709	3.254371	6.192578
H	3.009500	3.857626	4.084720
H	2.780850	2.195058	2.269853
C	6.175241	-.766795	2.054430
C	7.258694	-1.632147	1.912473
C	7.048700	-3.004681	1.735482
C	5.742157	-3.499916	1.702772
C	4.657551	-2.631670	1.834900
H	6.350937	.295969	2.190732
H	8.270080	-1.236036	1.940503
H	7.893678	-3.679349	1.632817
H	5.566300	-4.565581	1.583740
H	3.644717	-3.023605	1.826815

T4 (Triplet state)

C	-.011760	.012408	-.008634
C	.009630	.006035	1.396619
C	1.193813	.011658	2.124296
C	2.456815	.023994	1.508085
C	2.435464	.030671	.102890
C	1.251319	.025241	-.624811
F	-1.150534	-.028542	2.081134
F	1.107371	.026783	3.469013
F	3.596017	.020418	-.581749
F	1.337593	.054671	-1.969289
C	-1.273189	.005467	-.783380
C	-1.476898	-1.044875	-1.771271
C	-.867130	-2.315499	-1.620211
C	-1.051147	-3.322632	-2.562278
C	-1.840829	-3.096658	-3.695410
C	-2.440953	-1.844691	-3.873470
C	-2.266538	-.836381	-2.930328
H	-.261883	-2.512999	-.740888
H	-.580277	-4.289856	-2.411382
H	-1.980023	-3.881775	-4.432497
H	-3.038372	-1.649627	-4.759604
H	-2.710328	.137890	-3.102968
C	-2.253238	1.051493	-.522724
C	-1.847687	2.330100	-.065586
C	-2.775981	3.333873	.192251
C	-4.143625	3.095991	.014483
C	-4.568745	1.835911	-.422165

C	-3.643750	.831005	-.689398
H	-.789891	2.536505	.064729
H	-2.432490	4.307542	.529879
H	-4.867817	3.878305	.220921
H	-5.628951	1.631812	-.542590
H	-3.993739	-.149355	-.993413
C	3.718512	.029113	2.282631
C	3.940166	-1.029054	3.258036
C	3.349852	-2.307213	3.093496
C	3.551649	-3.322239	4.023348
C	4.340224	-3.097185	5.157444
C	4.921052	-1.838121	5.348927
C	4.728815	-.821731	4.418037
H	2.745914	-2.503974	2.213113
H	3.095582	-4.294873	3.862117
H	4.493305	-3.888563	5.885018
H	5.517258	-1.644064	6.236101
H	5.157662	.157291	4.601207
C	4.680749	1.094363	2.033458
C	4.253263	2.371180	1.591537
C	5.164218	3.393422	1.344607
C	6.535862	3.176562	1.518367
C	6.982593	1.918802	1.939855
C	6.074967	.895374	2.196155
H	3.192007	2.561181	1.464461
H	4.803990	4.365002	1.018609
H	7.246574	3.973298	1.320412
H	8.046225	1.731177	2.056823
H	6.441889	-.082478	2.488024

T4 (Singlet state, quinoidal)

C	-.025906	.009584	-.019289
C	.013759	.016646	1.430280
C	1.158930	.015312	2.165323
C	2.493062	.072520	1.599421
C	2.452191	.102729	.150120
C	1.308823	.044359	-.585459
F	-1.146065	-.058907	2.116058
F	1.017249	.039777	3.507309
F	3.614076	.110744	-.536324
F	1.447728	.101519	-1.926724
C	-1.199538	-.027255	-.774033
C	-1.231096	-.578306	-2.148582
C	-.594905	-1.797657	-2.454717
C	-.670012	-2.343864	-3.734742
C	-1.377926	-1.681005	-4.741805
C	-2.019799	-.472289	-4.453521
C	-1.958025	.066482	-3.169433
H	-.055352	-2.326113	-1.674396
H	-.178659	-3.289376	-3.945428

H	-1.433352	-2.104821	-5.740270
H	-2.570478	.050891	-5.230019
H	-2.460079	1.004128	-2.953185
C	-2.486690	.481852	-.245649
C	-2.556566	1.724826	.413746
C	-3.774940	2.229814	.864547
C	-4.952395	1.500878	.672728
C	-4.901290	.267224	.015651
C	-3.685316	-.230661	-.449646
H	-1.648307	2.303402	.553935
H	-3.805705	3.194438	1.362915
H	-5.901342	1.892279	1.027573
H	-5.810543	-.307514	-.135558
H	-3.654190	-1.188164	-.959922
C	3.666865	.093445	2.354595
C	3.730604	-.479453	3.719234
C	3.171384	-1.741587	4.000900
C	3.277036	-2.305315	5.271137
C	3.938977	-1.617753	6.292779
C	4.504434	-.366088	6.028821
C	4.412837	.190945	4.754293
H	2.667877	-2.288146	3.209064
H	2.845634	-3.283565	5.462801
H	4.018016	-2.055311	7.283700
H	5.018742	.176553	6.816823
H	4.855330	1.162274	4.557273
C	4.921397	.688086	1.837723
C	4.916999	1.940706	1.192947
C	6.103446	2.524788	.752730
C	7.322942	1.867326	.940749
C	7.345634	.625402	1.583599
C	6.161077	.048413	2.038342
H	3.975282	2.463761	1.055559
H	6.076411	3.495246	.265606
H	8.247156	2.320415	.594133
H	8.287999	.105887	1.731876
H	6.187536	-.915169	2.537249

T5 (Triplet state)

C	-.015463	-.003525	-.012890
C	.007882	-.080271	1.403171
C	1.223098	.073942	2.102985
C	2.460198	-.046311	1.420174
C	2.433346	-.142365	.005396
C	1.225522	.052709	-.697171
C	-1.290954	-.426434	2.150744
C	1.228915	.465863	3.590135
C	3.717126	-.552105	-.735983
C	1.236104	.419460	-2.190720
C	-1.309978	.017450	-.760623

C	-1.620075	-1.094386	-1.649433
C	-1.087571	-2.386905	-1.417387
C	-1.375289	-3.450785	-2.266103
C	-2.195888	-3.262226	-3.383572
C	-2.720328	-1.990805	-3.642376
C	-2.440394	-.924764	-2.793017
H	-.459596	-2.558939	-.549450
H	-.960804	-4.432115	-2.054251
H	-2.416499	-4.092158	-4.048061
H	-3.338951	-1.826201	-4.519953
H	-2.822573	.061312	-3.031616
C	-2.207450	1.149562	-.565317
C	-1.705385	2.428095	-.218225
C	-2.556816	3.509958	-.018793
C	-3.941165	3.353498	-.147957
C	-4.461417	2.096400	-.475814
C	-3.613248	1.012789	-.682958
H	-.634355	2.574699	-.125470
H	-2.139726	4.480005	.235630
H	-4.605623	4.197079	.012853
H	-5.535671	1.956403	-.555212
H	-4.036990	.037957	-.896498
C	3.754038	-.071069	2.169176
C	4.015270	-1.183786	3.072378
C	3.427987	-2.455192	2.855940
C	3.669343	-3.519560	3.718352
C	4.496271	-3.352251	4.834571
C	5.074324	-2.101221	5.078122
C	4.841018	-1.035060	4.214917
H	2.793703	-2.611150	1.989540
H	3.213722	-4.484975	3.518113
H	4.680649	-4.182484	5.509630
H	5.698617	-1.952049	5.954436
H	5.264607	-.063233	4.441696
C	4.698526	1.019796	1.961196
C	4.249956	2.313164	1.596110
C	5.145186	3.356735	1.385197
C	6.521635	3.145809	1.521022
C	6.989330	1.872906	1.866669
C	6.097253	.827325	2.084732
H	3.185980	2.501913	1.498772
H	4.768611	4.339526	1.116857
H	7.220344	3.959516	1.351668
H	8.056775	1.690420	1.951418
H	6.480585	-.161126	2.311634
F	3.428235	-1.210473	-1.881977
F	4.530160	.475918	-1.047475
F	4.444759	-1.426529	-.000395
F	2.251520	1.310656	3.866210
F	.108148	1.150314	3.915779

F	1.334877	-.575290	4.438211
F	-1.027526	-1.079175	3.306132
F	-2.061422	.638027	2.447483
F	-2.054012	-1.281450	1.428241
F	2.383691	1.053100	-2.526124
F	1.089499	-.630971	-3.021103
F	.248375	1.299243	-2.483141

T5(singlet state bitradical)

C	-.016600	-.003386	-.013407
C	.008351	-.078402	1.403850
C	1.223728	.073268	2.102950
C	2.462003	-.045860	1.419698
C	2.433671	-.140351	.003715
C	1.225503	.051792	-.698156
C	-1.287311	-.433707	2.152943
C	1.228403	.474648	3.587734
C	3.714156	-.558598	-.738873
C	1.237530	.427409	-2.189641
C	-1.308605	.016889	-.758785
C	-1.608876	-1.079644	-1.670746
C	-1.075848	-2.374427	-1.455416
C	-1.356018	-3.425031	-2.323190
C	-2.170327	-3.219584	-3.442155
C	-2.697327	-1.945586	-3.682769
C	-2.424624	-.892969	-2.814504
H	-.454373	-2.558726	-.585300
H	-.941210	-4.409009	-2.124704
H	-2.385294	-4.038983	-4.121431
H	-3.312322	-1.768634	-4.560488
H	-2.809360	.095868	-3.037440
C	-2.221831	1.133608	-.543880
C	-1.734139	2.414870	-.187866
C	-2.598597	3.483585	.026616
C	-3.981234	3.310050	-.097811
C	-4.486929	2.049906	-.436870
C	-3.625728	.979473	-.658357
H	-.664382	2.573842	-.100658
H	-2.193053	4.456586	.288301
H	-4.655874	4.143282	.074293
H	-5.559636	1.897370	-.513954
H	-4.037188	.001508	-.881783
C	3.753060	-.071140	2.166779
C	4.004498	-1.167953	3.093035
C	3.416832	-2.441407	2.892735
C	3.650150	-3.491864	3.774410
C	4.470227	-3.307308	4.892837
C	5.050711	-2.054025	5.118732
C	4.825144	-1.001913	4.236415

H	2.789501	-2.609971	2.023602
H	3.194274	-4.459753	3.587140
H	4.648580	-4.126533	5.582829
H	5.670877	-1.892276	5.995739
H	5.251122	-.027607	4.447987
C	4.712652	1.003521	1.940153
C	4.278819	2.298804	1.565470
C	5.186725	3.328540	1.340470
C	6.560679	3.100701	1.473190
C	7.013441	1.825609	1.830739
C	6.108682	.793894	2.062231
H	3.216684	2.499783	1.472358
H	4.822041	4.313641	1.064308
H	7.269268	3.903554	1.293279
H	8.078770	1.630747	1.914460
H	6.479353	-.197000	2.299647
F	3.418991	-1.215443	-1.884418
F	4.536318	.461253	-1.052749
F	4.434749	-1.439397	-.003011
F	2.247700	1.326796	3.855947
F	.104825	1.157273	3.908376
F	1.338436	-.557770	4.445697
F	-1.017035	-1.085087	3.307811
F	-2.067763	.622516	2.452437
F	-2.043281	-1.295710	1.430327
F	2.387633	1.059425	-2.520319
F	1.087549	-.614840	-3.029501
F	.253147	1.314091	-2.474834

T5 (Singlet state quinoidal)

C	-.090142	.323696	-.018070
C	-.110555	.310209	1.335516
C	1.225678	.184960	2.007688
C	2.193445	1.152286	1.380060
C	2.230152	1.117807	.027104
C	1.276837	.161028	-.626730
C	1.550613	-.775066	2.917749
C	.626877	-1.879585	3.314141
C	.575702	-2.311342	4.653318
C	-.281925	-3.339024	5.040820
C	-1.085027	-3.983138	4.094546
C	-1.019219	-3.589685	2.756897
C	-.172801	-2.549163	2.370455
H	1.200568	-1.830408	5.397626
H	-.319526	-3.640700	6.083424
H	-1.747881	-4.788560	4.396547
H	-1.625613	-4.090495	2.007995
H	-.125267	-2.270685	1.324981
C	2.918550	-.873286	3.533682
C	3.865048	-1.744649	2.971479

C	5.120691	-1.906822	3.559336
C	5.440511	-1.217214	4.731885
C	4.496497	-.366879	5.311050
C	3.243398	-.195350	4.717755
H	3.613016	-2.305537	2.076099
H	5.843893	-2.578380	3.105707
H	6.415785	-1.345121	5.192042
H	4.733913	.171024	6.224200
H	2.520669	.474985	5.170341
C	-1.320977	.663193	2.196319
F	-1.168460	.287583	3.483494
F	-1.507026	2.007448	2.219598
F	-2.462051	.094514	1.752072
C	2.902660	2.184774	2.246047
F	4.194209	1.863697	2.491598
F	2.281143	2.312789	3.443576
F	2.899407	3.415806	1.691744
C	2.960763	2.126229	-.856339
F	3.070573	1.709304	-2.135005
F	4.210110	2.393544	-.419821
F	2.276415	3.297184	-.904220
C	-1.280378	.662918	-.905647
F	-.863994	1.103871	-2.117691
F	-2.042826	1.652186	-.391871
F	-2.094304	-.397078	-1.118052
C	1.637544	-.818806	-1.501070
C	3.054433	-1.110594	-1.871767
C	.641082	-1.774547	-2.095659
C	4.085808	-1.096608	-.915581
C	5.402304	-1.388838	-1.275962
C	5.716123	-1.701311	-2.599722
C	4.698035	-1.736942	-3.557688
C	3.380931	-1.461234	-3.195733
H	3.860325	-.873731	.119932
H	6.179559	-1.371711	-.517724
H	6.740976	-1.923431	-2.881906
H	4.929159	-1.985368	-4.589354
H	2.601513	-1.505471	-3.948331
C	-.028805	-1.488697	-3.294001
C	-.891088	-2.428092	-3.864852
C	-1.094927	-3.664187	-3.248322
C	-.421264	-3.962602	-2.060875
C	.448743	-3.028960	-1.495284
H	.115597	-.527435	-3.775083
H	-1.407369	-2.187456	-4.789555
H	-1.769551	-4.390938	-3.690991
H	-.564549	-4.925212	-1.578518
H	.990907	-3.277671	-.587547

T5 (Transition state connecting the two conformers of the singlet states)

C	-.070849	.171864	.076293
C	.028608	.341803	1.524354
C	1.151534	-.150807	2.158747
C	2.120350	-1.040426	1.478430
C	2.247939	-.781861	.047211
C	1.231021	-.089314	-.579681
C	-1.130964	.903231	2.354018
C	1.612297	.422808	3.514702
C	3.378053	-1.392878	-.788112
C	1.492250	.692082	-1.883757
C	-1.221014	.200774	-.740191
C	-1.286525	-.684765	-1.925453
C	-.826510	-2.017451	-1.846144
C	-.939030	-2.881850	-2.932204
C	-1.509458	-2.435260	-4.128211
C	-1.979426	-1.121013	-4.222768
C	-1.880651	-.259828	-3.133118
H	-.403462	-2.378391	-.914621
H	-.587195	-3.905424	-2.843831
H	-1.591657	-3.106820	-4.977722
H	-2.418507	-.765468	-5.150252
H	-2.235322	.761359	-3.220848
C	-2.385135	1.097827	-.576148
C	-2.190552	2.482107	-.418847
C	-3.279533	3.353906	-.370090
C	-4.581149	2.858444	-.467576
C	-4.789743	1.483381	-.632768
C	-3.704486	.615452	-.703494
H	-1.180983	2.872872	-.361224
H	-3.107541	4.420388	-.258845
H	-5.428297	3.536644	-.422636
H	-5.799614	1.090879	-.708471
H	-3.871310	-.449259	-.833276
C	2.774546	-2.018398	2.257998
C	2.046254	-2.651584	3.380496
C	.692553	-3.025123	3.230189
C	.010355	-3.679625	4.252678
C	.661927	-3.973959	5.454335
C	2.005209	-3.619383	5.618743
C	2.692725	-2.977081	4.592397
H	.187121	-2.821913	2.292010
H	-1.027292	-3.966263	4.109704
H	.129109	-4.479778	6.254135
H	2.515801	-3.839797	6.551544
H	3.729872	-2.693480	4.734670
C	4.182648	-2.444575	2.112425
C	5.204773	-1.480118	2.045675
C	6.546398	-1.863987	2.017817
C	6.890473	-3.216705	2.045425

C	5.883881	-4.187819	2.119749
C	4.546840	-3.806210	2.170365
H	4.942621	-.428176	2.044535
H	7.320697	-1.103620	1.977524
H	7.934372	-3.515231	2.016747
H	6.144713	-5.241936	2.140692
H	3.770256	-4.562357	2.229027
F	3.116197	-1.393867	-2.111096
F	4.568592	-.774088	-.626744
F	3.541112	-2.702488	-.471034
F	2.971048	.405640	3.597677
F	1.270999	1.733629	3.611581
F	1.164876	-.188450	4.630100
F	-1.025981	.627936	3.670004
F	-1.272059	2.243354	2.245983
F	-2.300257	.326931	1.978042
F	2.775768	1.134436	-1.917200
F	1.262842	.053125	-3.048994
F	.728356	1.818587	-1.916940

T6 (Triplet state)

C	-0.029593	-0.006687	-0.021094
C	0.004915	-0.007185	1.394026
C	1.229372	-0.014302	2.103684
C	2.473803	-0.050421	1.430077
C	2.438971	-0.066005	0.015027
C	1.215099	-0.030436	-0.694770
C	-1.223927	-0.043848	2.133576
C	1.199715	0.059252	3.536146
C	3.664944	-0.163128	-0.723850
C	1.247193	0.024606	-2.128015
C	-1.312319	0.017393	-0.763791
C	-1.627169	-1.105295	-1.635630
C	-1.115648	-2.400072	-1.366688
C	-1.409439	-3.478242	-2.195300
C	-2.214345	-3.300855	-3.325874
C	-2.718868	-2.028346	-3.618596
C	-2.434099	-0.947864	-2.790475
H	-0.505572	-2.561943	-0.483205
H	-1.014153	-4.461247	-1.956904
H	-2.438281	-4.141411	-3.975520
H	-3.324325	-1.875334	-4.507118
H	-2.799134	0.038461	-3.053761
C	-2.193954	1.162003	-0.580080
C	-1.668764	2.437898	-0.253811
C	-2.502907	3.535829	-0.069034
C	-3.889640	3.397445	-0.193081
C	-4.431106	2.144127	-0.502156
C	-3.601883	1.044298	-0.695170
H	-0.594133	2.569653	-0.172220

H	-2.071029	4.503709	0.167785
H	-4.540691	4.253050	-0.042143
H	-5.507579	2.020890	-0.576238
H	-4.039990	0.072606	-0.893406
C	3.756524	-0.071443	2.172833
C	4.023386	-1.195217	3.059147
C	3.455769	-2.469979	2.807343
C	3.702741	-3.548851	3.650158
C	4.514917	-3.391813	4.778546
C	5.074705	-2.138782	5.054333
C	4.836987	-1.058002	4.211880
H	2.838749	-2.616849	1.926057
H	3.265013	-4.516809	3.424644
H	4.702103	-4.232653	5.439348
H	5.686631	-2.000488	5.940829
H	5.244700	-0.085137	4.462259
C	4.685282	1.032831	1.975310
C	4.213449	2.324870	1.631302
C	5.092174	3.385030	1.433401
C	6.471870	3.191514	1.561882
C	6.960967	1.921203	1.888480
C	6.087072	0.858907	2.094241
H	3.145242	2.499616	1.546084
H	4.700724	4.366545	1.182840
H	7.157753	4.017547	1.400825
H	8.031373	1.754848	1.966125
H	6.484930	-0.127258	2.305507
N	4.643767	-0.274802	-1.341933
N	1.297726	0.100552	-3.287437
N	-2.206398	-0.106623	2.752766
N	1.152406	0.151252	4.694543

T6 (singlet state, diradical)

C	-0.059312	-0.012447	-0.035618
C	0.007007	-0.001147	1.410935
C	1.215333	-0.029204	2.109846
C	2.501456	-0.055735	1.445014
C	2.435296	-0.059592	-0.001630
C	1.226608	-0.047089	-0.700421
C	-1.195656	-0.101892	2.189046
C	1.143788	0.114200	3.536811
C	3.632627	-0.219035	-0.778136
C	1.303475	0.074677	-2.129107
C	-1.290569	0.011544	-0.746621
C	-1.465612	-0.762639	-1.984704
C	-0.933436	-2.066517	-2.094066
C	-1.127127	-2.824086	-3.246074
C	-1.849984	-2.295656	-4.319782
C	-2.389040	-1.007662	-4.226914
C	-2.209931	-0.253451	-3.071105

H	-0.389735	-2.491280	-1.255525
H	-0.717412	-3.827902	-3.305513
H	-1.993526	-2.882930	-5.221773
H	-2.943321	-0.588761	-5.061432
H	-2.617304	0.750300	-3.012981
C	-2.425190	0.814324	-0.264771
C	-2.211223	2.105757	0.265738
C	-3.279903	2.892249	0.687691
C	-4.587283	2.405346	0.598029
C	-4.818381	1.130100	0.070322
C	-3.753640	0.347479	-0.366690
H	-1.200147	2.498331	0.318874
H	-3.093633	3.886200	1.083181
H	-5.420290	3.014803	0.935270
H	-5.831068	0.743349	0.006798
H	-3.939802	-0.646314	-0.760115
C	3.732539	-0.074619	2.156250
C	3.874938	-0.839731	3.404178
C	3.289381	-2.119033	3.529697
C	3.452297	-2.869699	4.690971
C	4.196865	-2.358308	5.758080
C	4.788167	-1.094695	5.649401
C	4.639931	-0.348161	4.484277
H	2.727906	-2.531136	2.696552
H	3.001524	-3.854935	4.762817
H	4.316618	-2.939810	6.667250
H	5.359529	-0.688622	6.478727
H	5.088349	0.637148	4.413812
C	4.900339	0.673037	1.664976
C	4.741864	1.966346	1.119882
C	5.843243	2.701132	0.688784
C	7.128376	2.159103	0.783648
C	7.304558	0.881064	1.325702
C	6.207356	0.150264	1.771959
H	3.748528	2.401354	1.062578
H	5.699857	3.697629	0.282000
H	7.986737	2.728052	0.439284
H	8.299644	0.451642	1.393202
H	6.350402	-0.846077	2.176740
N	4.565037	-0.416060	-1.445650
N	1.418890	0.240158	-3.274987
N	-2.135141	-0.252526	2.858709
N	1.035226	0.299773	4.680288

T7 (Triplet state)

C	-.029819	.162422	.057822
C	.040286	-.233789	1.397874
C	1.256554	-.243083	2.049850
C	2.415960	.143904	1.369332
C	2.345836	.540190	.029338

C	1.129554	.549525	-.622626
H	-.868936	-.517751	1.917907
H	1.323247	-.534624	3.093075
C	3.700484	.132714	2.056472
H	3.254996	.824173	-.490781
H	1.062943	.841081	-1.665849
C	-1.314209	.173423	-.629547
C	-2.067435	-1.092264	-.638248
C	-1.724903	1.449376	-1.239691
C	-2.655040	1.419486	-2.317053
C	-3.166817	2.556952	-2.926579
C	-2.755670	3.816515	-2.488850
C	-1.781290	3.895191	-1.492826
C	-1.220519	2.771227	-.865483
H	-2.953332	.452709	-2.703402
H	-3.873076	2.456826	-3.745935
H	-3.155999	4.722616	-2.933659
H	-1.420333	4.872835	-1.186486
C	-3.479975	-1.041562	-.808465
C	-4.287563	-2.167179	-.898409
C	-3.712905	-3.435510	-.808757
C	-2.343881	-3.534899	-.556533
C	-1.494377	-2.423525	-.434986
H	-3.953870	-.068099	-.835510
H	-5.360092	-2.050899	-1.026233
H	-4.318204	-4.332655	-.897498
H	-1.902262	-4.519539	-.432698
C	-.101267	-2.761160	.056589
C	4.110933	-1.143159	2.666850
C	4.453711	1.398373	2.065242
C	5.040945	-1.113212	3.744326
C	5.552262	-2.250630	4.354363
C	5.140763	-3.510209	3.917101
C	4.166579	-3.588957	2.920849
C	3.606312	-2.465111	2.292948
H	5.339495	-.146426	4.130440
H	6.258412	-2.150404	5.173804
H	5.540717	-4.416272	4.362327
H	3.805482	-4.566631	2.614776
C	5.866240	1.347749	2.235544
C	6.673744	2.473394	2.325662
C	6.099000	3.741708	2.236104
C	4.729999	3.841021	1.983754
C	3.880587	2.729592	1.862025
H	6.340153	.374287	2.262579
H	7.746265	2.357162	2.453599
H	6.704219	4.638891	2.324991
H	4.288323	4.825637	1.859922
C	2.487449	3.067204	1.370274
C	1.289433	2.815561	2.059306

C	.055239	2.824815	1.398618
C	-.033051	3.086586	.021452
C	1.121943	3.623821	-.580865
C	2.353987	3.613855	.078254
H	1.323739	2.497959	3.097027
H	-.831117	2.514276	1.943449
H	1.086849	3.926671	-1.623965
H	3.245134	3.908542	-.469511
C	.032215	-3.308013	1.348469
C	1.264226	-3.317847	2.007702
C	2.419193	-2.780403	1.405574
C	2.330961	-2.518545	.028382
C	1.096801	-2.509250	-.632353
H	-.858902	-3.602866	1.896192
H	3.217348	-2.207922	-.516346
H	1.062482	-2.191566	-1.670045
H	1.299235	-3.620810	3.050768

T7 (Singlet state, diradical)

C	-.003704	.001518	.001716
C	.002428	-.057701	1.405819
C	1.195040	-.058393	2.087948
C	2.409088	-.001095	1.382442
C	2.402957	.058183	-.021661
C	1.210344	.058874	-.703791
H	-.941966	-.069665	1.939498
H	1.213427	-.071382	3.172548
C	3.660206	-.003647	2.098728
H	3.347352	.070142	-.555340
H	1.191957	.071858	-1.788391
C	-1.254825	.003964	-.714564
C	-2.207196	-1.075187	-.388661
C	-1.452315	1.084066	-1.700139
C	-2.361423	.870261	-2.771962
C	-2.682241	1.838190	-3.714764
C	-2.087078	3.097671	-3.636348
C	-1.131412	3.329488	-2.646297
C	-.762039	2.373489	-1.685995
H	-2.804862	-.113004	-2.873396
H	-3.384799	1.604231	-4.509623
H	-2.335569	3.879459	-4.347900
H	-.633257	4.294155	-2.612573
C	-3.591204	-.857866	-.629061
C	-4.567898	-1.826109	-.435720
C	-4.200456	-3.089176	.029212
C	-2.863598	-3.324073	.353451
C	-1.847588	-2.367619	.194171
H	-3.900884	.127842	-.955006
H	-5.608582	-1.589904	-.638996
H	-4.940571	-3.871644	.167844

H	-2.583613	-4.291523	.760293
C	-.520142	-2.761766	.812159
C	3.857669	-1.083795	3.084263
C	4.612619	1.075466	1.772843
C	4.766770	-.870027	4.156101
C	5.087633	-1.838005	5.098836
C	4.492544	-3.097514	5.020326
C	3.536903	-3.329315	4.030248
C	3.167474	-2.373266	3.070018
H	5.210171	.113249	4.257598
H	5.790180	-1.604066	5.893712
H	4.741077	-3.879341	5.731821
H	3.038811	-4.294013	3.996443
C	5.996617	.858117	2.013278
C	6.973341	1.826318	1.819881
C	6.605943	3.089356	1.354837
C	5.269097	3.324262	1.030547
C	4.253057	2.367852	1.189897
H	6.306262	-.127579	2.339293
H	8.014015	1.590099	2.023191
H	7.346083	3.871791	1.216151
H	4.989148	4.291683	.623611
C	2.925638	2.761949	.571823
C	1.696034	2.864804	1.246320
C	.481403	2.866653	.550444
C	.442186	2.764345	-.851607
C	1.668119	2.967005	-1.516654
C	2.880474	2.966638	-.822001
H	1.675530	2.829882	2.331207
H	-.444278	2.833380	1.116648
H	1.687657	2.984949	-2.603013
H	3.807989	2.984036	-1.387988
C	-.474957	-2.966748	2.205939
C	.737399	-2.967119	2.900587
C	1.963302	-2.764169	2.235572
C	1.924098	-2.866259	.833505
C	.709464	-2.864406	.137634
H	-1.402469	-2.984340	2.771926
H	2.849772	-2.832769	.267304
H	.729961	-2.829263	-.947246
H	.717868	-2.985259	3.986944

T8 (Triple state)

C	-.027401	-.014069	-.013694
C	-.020416	-.012626	1.402922
C	1.232734	-.029784	2.125420
C	2.462203	-.054920	1.421770
C	2.455068	-.058792	.004979
C	1.201889	-.035293	-.717417

C	-1.238635	-.015136	2.158665
C	1.189270	.001356	3.558055
C	3.672040	-.107612	-.751219
C	1.245688	-.013060	-2.150230
C	-1.323318	.008109	-.760780
C	-1.763920	-1.213290	-1.418692
C	-1.312534	-2.478610	-.960364
C	-1.705945	-3.658573	-1.583959
C	-2.551930	-3.625492	-2.698591
C	-2.992355	-2.388522	-3.184500
C	-2.609053	-1.205188	-2.560069
H	-.660253	-2.523078	-.094579
H	-1.352254	-4.610612	-1.198046
H	-2.854690	-4.546979	-3.186872
H	-3.628638	-2.345744	-4.064282
H	-2.931239	-.257920	-2.978250
C	-2.081230	1.251468	-.791397
C	-1.421832	2.497326	-.626294
C	-2.126498	3.697171	-.631706
C	-3.517762	3.703352	-.785343
C	-4.194253	2.485858	-.926785
C	-3.493964	1.282946	-.931694
H	-.343170	2.511077	-.510705
H	-1.588128	4.633812	-.516639
H	-4.067029	4.640127	-.784295
H	-5.276725	2.473603	-1.020630
H	-4.043555	.350875	-1.003759
C	3.757772	-.077787	2.169118
C	4.148540	-1.310449	2.838157
C	3.652070	-2.560905	2.386164
C	3.995498	-3.750265	3.021354
C	4.835170	-3.741082	4.141154
C	5.320499	-2.518330	4.620150
C	4.987173	-1.326000	3.984116
H	3.004219	-2.587020	1.516331
H	3.607623	-4.690935	2.640429
H	5.098816	-4.669694	4.638543
H	5.952951	-2.493254	5.503365
H	5.343692	-.388621	4.396800
C	4.565945	1.133340	2.189037
C	3.959223	2.403588	2.008234
C	4.712896	3.573239	2.003279
C	6.102757	3.523707	2.161997
C	6.727787	2.280895	2.319152
C	5.978392	1.108091	2.334471
H	2.882419	2.460630	1.888210
H	4.214042	4.529979	1.876058
H	6.690254	4.436942	2.152839
H	7.808500	2.224909	2.417314
H	6.488907	.155085	2.419271

C	-.000058	.015169	4.239950
C	-1.232776	-.002673	3.529535
H	2.124208	.010784	4.106220
H	-2.171136	-.002869	4.076536
H	-2.181245	-.023626	1.623913
H	-.002972	.036569	5.325884
C	2.434448	-.043921	-2.832511
C	3.666010	-.101176	-2.122165
H	4.614171	-.146695	-.217038
H	4.603631	-.136876	-2.669274
H	2.437698	-.028217	-3.918536
H	.311221	.025943	-2.698015

T8 (Singlet state, diradical)

C	-.031646	-.005955	-.013947
C	-.021124	-.007482	1.406986
C	1.232310	-.015715	2.128196
C	2.466088	-.049055	1.423934
C	2.455308	-.057945	.002877
C	1.202349	-.022863	-.718288
C	-1.236914	-.031925	2.165397
C	1.188306	.040799	3.559556
C	3.668918	-.133617	-.755695
C	1.247787	.023636	-2.149973
C	-1.322042	.011256	-.757167
C	-1.693738	-1.159642	-1.542470
C	-1.198838	-2.442376	-1.193724
C	-1.528335	-3.572723	-1.935550
C	-2.355272	-3.467744	-3.059902
C	-2.842420	-2.209895	-3.434649
C	-2.521095	-1.076761	-2.692501
H	-.562391	-2.540604	-.320352
H	-1.141242	-4.542063	-1.634034
H	-2.609396	-4.350135	-3.639640
H	-3.467192	-2.110281	-4.318058
H	-2.880431	-.107656	-3.021356
C	-2.165709	1.197509	-.670428
C	-1.590534	2.467245	-.408309
C	-2.375415	3.612002	-.307734
C	-3.765474	3.534494	-.452242
C	-4.358740	2.289736	-.694000
C	-3.577648	1.142582	-.803256
H	-.513577	2.544477	-.300649
H	-1.901077	4.570886	-.118218
H	-4.377412	4.427805	-.369769
H	-5.438620	2.211238	-.785663
H	-4.061495	.183968	-.956288
C	3.756358	-.077104	2.166816
C	4.081930	-1.255356	2.961612

C	3.535794	-2.520184	2.623924
C	3.820748	-3.656658	3.374718
C	4.652534	-3.575669	4.497471
C	5.190134	-2.335306	4.861291
C	4.913332	-1.196304	4.110222
H	2.895103	-2.600285	1.751828
H	3.394962	-4.612205	3.081433
H	4.871817	-4.462740	5.084245
H	5.819528	-2.253498	5.743246
H	5.311461	-.239697	4.430855
C	4.646478	1.073978	2.069808
C	4.121752	2.363539	1.798935
C	4.951395	3.475194	1.686870
C	6.337629	3.343392	1.828535
C	6.881344	2.077966	2.079399
C	6.055394	.963838	2.200083
H	3.048409	2.482886	1.693232
H	4.515146	4.450639	1.490512
H	6.984577	4.210767	1.736964
H	7.957470	1.957148	2.169213
H	6.501073	-.012010	2.360012
C	.000015	.046194	4.244305
C	-1.231380	-.007496	3.536595
H	2.121335	.076180	4.108537
H	-2.169793	-.024144	4.083249
H	-2.180730	-.066356	1.635276
H	-.000449	.086475	5.329700
C	2.435145	-.020008	-2.834934
C	3.663762	-.116799	-2.127043
H	4.611062	-.201757	-.225872
H	4.600648	-.172955	-2.673681
H	2.436782	.013943	-3.920545
H	.316457	.091449	-2.698841

T8 (singlet state quinoidal)

C	-.021375	.056685	-.026066
C	-.012232	.066033	1.390511
C	1.306377	.025279	2.088843
C	2.299324	.904245	1.403119
C	2.302691	.861007	-.012577
C	1.311384	-.021691	-.695023
C	1.568775	-.762071	3.168375
C	.618809	-1.804026	3.678857
C	.334649	-1.917571	5.051297
C	-.521576	-2.911401	5.529583
C	-1.093036	-3.832526	4.648078
C	-.798244	-3.749545	3.284234
C	.046712	-2.747669	2.806309
H	.783973	-1.221057	5.751909
H	-.735582	-2.968944	6.593174

H	-1.752744	-4.611102	5.020068
H	-1.225381	-4.467094	2.589283
H	.267673	-2.692468	1.745515
C	2.889654	-.750928	3.886659
C	3.908785	-1.633034	3.495950
C	5.124887	-1.671148	4.181510
C	5.337432	-.838696	5.283692
C	4.325336	.033257	5.692535
C	3.112747	.076000	4.998932
H	3.744495	-2.291544	2.647587
H	5.904434	-2.354554	3.856608
H	6.281477	-.870847	5.819758
H	4.478779	.684326	6.548740
H	2.334892	.765549	5.317138
C	-1.220692	.198844	2.089212
C	3.162875	1.795332	2.057501
C	3.163514	1.705558	-.728373
C	-1.247201	.178312	-.697452
C	1.616312	-.830735	-1.747187
C	3.017820	-1.063438	-2.227394
C	.590679	-1.667276	-2.460360
C	4.039595	-1.412556	-1.325520
C	5.333589	-1.680233	-1.772778
C	5.638887	-1.604493	-3.134701
C	4.631993	-1.272675	-4.044728
C	3.334120	-1.018572	-3.596754
H	3.814858	-1.473060	-.265841
H	6.103550	-1.949873	-1.055330
H	6.646852	-1.810407	-3.483070
H	4.853102	-1.218253	-5.107035
H	2.560235	-.779470	-4.319076
C	-.083022	-1.188838	-3.595268
C	-.988910	-1.998572	-4.285789
C	-1.237511	-3.302740	-3.850783
C	-.568755	-3.792090	-2.725454
C	.343133	-2.984008	-2.042988
H	.096448	-.171568	-3.933865
H	-1.502298	-1.607705	-5.159966
H	-1.943916	-3.932106	-4.384179
H	-.753344	-4.805441	-2.379995
H	.870134	-3.374437	-1.176920
C	-2.430910	.312510	1.407744
C	-2.442817	.300930	.010636
H	-1.267195	.195559	-1.779166
H	-3.379088	.403775	-.530301
H	-3.357097	.423175	1.964149
H	-1.206398	.221792	3.173669
C	4.020342	2.623447	1.332989
C	4.021247	2.579812	-.063620
H	3.152626	1.856989	3.137737

H	3.149696	1.681139	-1.812824
H	4.675915	3.233222	-.632972
H	4.674004	3.312502	1.859989

T8 (Transition state connecting the two conformers of the singlet states)

C	-.015385	-.274850	-.047360
C	-.103790	-.338200	1.402232
C	1.072221	-.481222	2.213469
C	2.375075	-.708920	1.610136
C	2.448936	-.733387	.158269
C	1.295561	-.453369	-.649785
C	-1.366117	-.326431	2.064991
C	.924288	-.323550	3.622690
C	3.652204	-1.100666	-.512432
C	1.496729	-.290645	-2.051847
C	-1.182200	-.041804	-.852021
C	-1.450355	-.842810	-2.061202
C	-1.141327	-2.221911	-2.080844
C	-1.410716	-3.006453	-3.199445
C	-1.996948	-2.438928	-4.336362
C	-2.315815	-1.076813	-4.335864
C	-2.053784	-.291444	-3.214268
H	-.695198	-2.672056	-1.199583
H	-1.168789	-4.065468	-3.182862
H	-2.206059	-3.050646	-5.209068
H	-2.766535	-.623166	-5.214301
H	-2.294839	.766617	-3.234179
C	-2.156683	1.007391	-.498100
C	-1.714288	2.232466	.051133
C	-2.612369	3.249384	.364813
C	-3.982926	3.076420	.140875
C	-4.441550	1.873565	-.406591
C	-3.543306	.856935	-.727419
H	-.651853	2.379572	.217880
H	-2.242152	4.183272	.778482
H	-4.683151	3.869598	.386013
H	-5.503801	1.724713	-.580012
H	-3.916210	-.075570	-1.138781
C	3.548456	-.900403	2.416052
C	3.534963	-1.796909	3.587518
C	2.803215	-3.005470	3.547289
C	2.798574	-3.882825	4.628780
C	3.524562	-3.581693	5.786583
C	4.259996	-2.393085	5.845112
C	4.271417	-1.516928	4.760857
H	2.246303	-3.252072	2.648783
H	2.232691	-4.808153	4.566470
H	3.520168	-4.265905	6.630018
H	4.823537	-2.145410	6.740466
H	4.835659	-.592112	4.826709

C	4.812680	-.209010	2.101158
C	4.795100	1.114661	1.605242
C	5.976559	1.797372	1.327725
C	7.215461	1.180835	1.536412
C	7.255649	-.126437	2.032527
C	6.074089	-.809370	2.316829
H	3.838941	1.604720	1.451085
H	5.932074	2.816810	.954762
H	8.136625	1.713669	1.319389
H	8.211071	-.618033	2.194026
H	6.121726	-1.828387	2.687217
C	-.309700	-.216135	4.225907
C	-1.476637	-.273346	3.437094
H	1.807105	-.297748	4.245061
H	-2.458177	-.258987	3.901764
H	-2.270570	-.351237	1.473896
H	-.377624	-.107013	5.304366
C	2.703541	-.561342	-2.658812
C	3.783862	-1.026407	-1.881717
H	4.495963	-1.441172	.070759
H	4.720836	-1.309369	-2.352435
H	2.810921	-.432633	-3.731890
H	.674056	.046008	-2.666111

M7a Triplet

C	0.001780	0.014246	0.001472
C	0.003305	-0.026248	1.413842
C	1.212393	0.011833	2.135003
C	2.455996	0.048936	1.469021
C	2.454721	0.074345	0.061833
C	1.244265	0.054626	-0.658917
N	-1.204084	-0.183596	2.121107
N	1.118867	-0.034344	3.564145
N	3.651746	0.081554	-0.706051
N	1.344805	0.112002	-2.070434
C	-1.288229	-0.018749	-0.770806
C	-1.878555	-1.407898	-1.137661
C	-1.937325	1.343600	-1.144178
C	3.745575	0.060517	2.244203
C	4.377242	-1.315075	2.599907
C	4.371821	1.438534	2.597048
C	5.890631	1.518868	2.288580
C	3.693989	2.576052	1.792353
C	4.174466	1.799745	4.100178
C	4.997296	-1.370189	4.019693
C	3.314740	-2.443474	2.540139
C	5.485890	-1.712933	1.578289
C	-3.475999	1.369898	-0.957145
C	-1.366729	2.486725	-0.265805
C	-1.629745	1.742492	-2.619646

C	-2.369014	-1.494922	-2.607000
C	-0.811800	-2.517510	-0.958412
C	-3.068468	-1.800936	-0.210776
H	-3.850969	2.370979	-1.199806
H	-3.996991	0.662610	-1.605536
H	-3.762452	1.153523	0.077669
H	-1.834109	3.434381	-0.556577
H	-1.570511	2.332453	0.798943
H	-0.286018	2.591466	-0.386204
H	-2.092730	2.712764	-2.839008
H	-0.553635	1.847627	-2.782753
H	-2.022976	1.021772	-3.339345
H	-3.413724	-2.808452	-0.474629
H	-2.753225	-1.811741	0.835224
H	-3.920789	-1.126726	-0.311878
H	-2.735611	-2.508797	-2.805407
H	-3.188061	-0.806463	-2.826976
H	-1.558866	-1.296440	-3.317569
H	-1.247128	-3.485239	-1.232133
H	0.063000	-2.353910	-1.595271
H	-0.468387	-2.582356	0.076631
H	4.148314	3.534701	2.067021
H	3.815942	2.445413	0.713541
H	2.622530	2.637581	1.999316
H	4.602898	2.790343	4.296161
H	3.111834	1.848777	4.356329
H	4.658930	1.092159	4.774226
H	6.256218	2.524401	2.527236
H	6.483567	0.810965	2.871369
H	6.096835	1.340110	1.227791
H	5.383843	-2.379263	4.202515
H	5.830834	-0.677042	4.153478
H	4.252260	-1.162492	4.795307
H	5.896379	-2.692476	1.852405
H	5.078796	-1.800188	0.567004
H	6.316040	-1.003475	1.560468
H	3.790898	-3.398178	2.790707
H	2.496693	-2.284006	3.248479
H	2.878425	-2.536813	1.542771
H	2.032876	-0.132464	3.990978
H	0.689362	0.807125	3.945533
H	-1.109995	-0.188319	3.126416
H	-1.996257	0.348033	1.789709
H	2.091660	-0.480609	-2.423573
H	0.473125	-0.105516	-2.537492
H	3.628799	0.783767	-1.441888
H	4.479426	0.210527	-0.137413

M7a Singlet quinoidal

C	-0.050729	-0.176796	0.057242

C	0.132150	-0.211148	1.567582
C	1.427057	-0.188980	1.962191
C	2.528138	-0.111591	0.933588
C	2.105810	1.053422	0.053032
C	0.837969	0.950854	-0.413272
N	-0.943610	-0.086782	2.502417
N	1.812022	-0.031261	3.312958
N	2.772771	2.316854	0.061807
N	0.176488	2.049540	-1.013779
C	-0.794685	-0.993359	-0.764333
C	-0.530152	-1.084449	-2.318718
C	-1.967848	-1.860798	-0.141699
C	3.634081	-0.916271	0.851945
C	3.731314	-2.292219	1.608136
C	4.872810	-0.398870	0.009924
C	6.111364	-1.320451	-0.173773
C	4.472544	-0.057301	-1.450320
C	5.444374	0.840127	0.764365
C	4.689595	-2.205870	2.824527
C	2.381776	-2.842492	2.140712
C	4.215302	-3.417509	0.636113
C	-2.767414	-2.836511	-1.055086
C	-1.453506	-2.786045	0.992024
C	-3.053397	-0.866371	0.364670
C	-1.652401	-0.392573	-3.137306
C	0.814836	-0.488589	-2.813241
C	-0.415063	-2.585131	-2.755554
H	6.903051	-0.706983	-0.618608
H	6.511156	-1.718833	0.759579
H	5.932396	-2.146994	-0.862415
H	6.218126	1.328317	0.157355
H	4.684049	1.575174	1.021559
H	5.920502	0.517272	1.697531
H	5.315549	0.422248	-1.962642
H	4.240084	-0.979225	-1.994292
H	3.599614	0.585833	-1.541368
H	3.894623	-4.386773	1.029021
H	3.768438	-3.307667	-0.357773
H	5.295034	-3.470247	0.525398
H	2.562116	-3.850424	2.530439
H	1.967285	-2.256514	2.956305
H	1.639672	-2.921715	1.342248
H	4.828503	-3.202952	3.260314
H	5.677674	-1.818025	2.567496
H	4.265819	-1.562687	3.602290
H	-3.638218	-3.164665	-0.477541
H	-3.153339	-2.384909	-1.969911
H	-2.210766	-3.737949	-1.312930
H	-3.858134	-1.417248	0.866997
H	-2.652862	-0.146203	1.070440

H	-3.497470	-0.331089	-0.483442
H	-2.301926	-3.316342	1.440949
H	-0.774126	-3.540229	0.578611
H	-0.934691	-2.259878	1.784827
H	0.200554	-2.649896	-3.657250
H	0.074737	-3.189576	-1.985828
H	-1.369733	-3.043183	-2.998449
H	0.916290	-0.731682	-3.876556
H	0.880453	0.593064	-2.736390
H	1.664205	-0.927373	-2.285674
H	-1.518746	-0.601970	-4.205701
H	-2.654831	-0.721129	-2.858547
H	-1.617727	0.695764	-3.014443
H	0.723182	2.562213	-1.698854
H	-0.735543	1.817174	-1.382834
H	3.558254	2.419087	-0.574328
H	2.117182	3.078871	-0.084861
H	-0.733815	-0.485960	3.411606
H	-1.269045	0.871098	2.621673
H	2.738132	0.368665	3.404661
H	1.141820	0.503633	3.857702

M7a Singlet diradical

C	0.001776	0.014273	0.001417
C	0.003446	-0.026928	1.413802
C	1.212424	0.010899	2.134865
C	2.456060	0.048782	1.468898
C	2.454647	0.074998	0.061674
C	1.244311	0.055415	-0.658955
N	-1.204117	-0.184103	2.120729
N	1.119353	-0.036130	3.563937
N	3.651771	0.082447	-0.705989
N	1.344438	0.113267	-2.070410
C	-1.288208	-0.018575	-0.770872
C	-1.879284	-1.407666	-1.136944
C	-1.936725	1.343917	-1.144825
C	3.745682	0.060266	2.244004
C	4.377503	-1.315364	2.599416
C	4.371727	1.438288	2.597338
C	5.890587	1.519023	2.289294
C	3.693889	2.575990	1.792904
C	4.173921	1.799016	4.100548
C	4.997169	-1.370874	4.019374
C	3.315246	-2.443968	2.538917
C	5.486480	-1.712735	1.577974
C	-3.475285	1.371000	-0.956780
C	-1.365027	2.487035	-0.267185
C	-1.629962	1.742108	-2.620636
C	-2.369882	-1.494887	-2.606237
C	-0.812852	-2.517581	-0.957609

C	-3.069296	-1.800175	-0.209981
H	-3.849938	2.372171	-1.199563
H	-3.997024	0.663724	-1.604585
H	-3.761161	1.155147	0.078298
H	-1.832116	3.434839	-0.557940
H	-1.568217	2.333107	0.797730
H	-0.284333	2.591198	-0.388283
H	-2.093121	2.712248	-2.840157
H	-0.553957	1.847251	-2.784381
H	-2.023575	1.021048	-3.339788
H	-3.415022	-2.807515	-0.473861
H	-2.753965	-1.811147	0.835981
H	-3.921335	-1.125588	-0.310997
H	-2.736776	-2.508711	-2.804334
H	-3.188770	-0.806267	-2.826301
H	-1.559721	-1.296796	-3.316890
H	-1.248540	-3.485265	-1.230930
H	0.061841	-2.354415	-1.594736
H	-0.469208	-2.582231	0.077367
H	4.147781	3.534643	2.068273
H	3.816366	2.445959	0.714086
H	2.622314	2.637061	1.999428
H	4.602344	2.789526	4.296985
H	3.111213	1.848002	4.356356
H	4.658148	1.091183	4.774509
H	6.255847	2.524612	2.528208
H	6.483581	0.811199	2.872105
H	6.097066	1.340497	1.228516
H	5.383851	-2.379938	4.201964
H	5.830521	-0.677614	4.153686
H	4.251840	-1.163590	4.794822
H	5.897109	-2.692242	1.851983
H	5.079618	-1.799880	0.566583
H	6.316483	-1.003099	1.560462
H	3.791564	-3.398683	2.789131
H	2.497027	-2.285034	3.247168
H	2.879153	-2.536955	1.541416
H	2.033772	-0.133850	3.990136
H	0.689607	0.804943	3.945950
H	-1.110332	-0.189320	3.126064
H	-1.995818	0.348251	1.789306
H	2.091503	-0.478824	-2.423978
H	0.472582	-0.104878	-2.536959
H	3.629415	0.785514	-1.441029
H	4.479219	0.210586	-0.136720

M7b Triplet

C	-0.006608	0.013241	-0.006345
C	0.002285	0.042250	1.407755
C	1.216641	-0.012945	2.123869

C	2.456187	-0.066747	1.444424
C	2.448026	-0.074536	0.029736
C	1.232329	-0.051346	-0.685952
C	-1.288482	0.133935	2.196472
C	1.151773	-0.010376	3.638007
C	3.740354	-0.110012	-0.760949
C	1.294199	-0.098100	-2.199581
C	-1.306613	0.048045	-0.770740
C	-1.963199	-1.311649	-1.146262
C	-1.905067	1.439272	-1.127082
C	3.754495	-0.117284	2.210559
C	4.299084	-1.514960	2.622866
C	4.463379	1.229720	2.529742
H	3.848740	-1.048711	-1.319881
H	3.779903	0.697961	-1.500890
H	4.611686	-0.011924	-0.115787
H	2.131598	-0.168968	4.085277
H	0.763186	0.940647	4.025476
H	0.483074	-0.793978	4.013210
H	-1.466458	-0.771415	2.791434
H	-1.268106	0.971136	2.904261
H	-2.149202	0.270957	1.544344
H	1.761752	0.804364	-2.614839
H	1.892157	-0.946969	-2.551930
H	0.303403	-0.185798	-2.642107
C	5.994228	1.197668	2.277112
C	3.898688	2.380027	1.656953
C	4.241817	1.673269	4.007850
C	4.853123	-1.561459	4.071557
C	3.190296	-2.595849	2.543352
C	5.433172	-2.004195	1.671304
C	-3.433100	1.536690	-0.875962
C	-1.247975	2.563455	-0.285514
C	-1.649066	1.823024	-2.616260
C	-2.517493	-1.351910	-2.595127
C	-0.943954	-2.475054	-1.035218
C	-3.134245	-1.683574	-0.186294
H	-3.772009	2.553480	-1.106342
H	-4.012252	0.854095	-1.501619
H	-3.684573	1.336391	0.170358
H	-1.667333	3.530107	-0.586610
H	-1.432861	2.441473	0.784738
H	-0.166187	2.605113	-0.435940
H	-2.088187	2.808080	-2.816706
H	-0.579309	1.891896	-2.828559
H	-2.092860	1.115532	-3.318093
H	-3.568405	-2.640574	-0.500424
H	-2.781612	-1.805242	0.840993
H	-3.934366	-0.941911	-0.188095
H	-2.917878	-2.351750	-2.799492

H	-3.328646	-0.639897	-2.762975
H	-1.733032	-1.156003	-3.332811
H	-1.446112	-3.418182	-1.278899
H	-0.106922	-2.357618	-1.727875
H	-0.534546	-2.563483	-0.025635
H	4.401480	3.315909	1.925939
H	4.065378	2.209102	0.590509
H	2.825414	2.519448	1.810006
H	4.757595	2.625826	4.180634
H	3.181356	1.832169	4.219006
H	4.630039	0.953151	4.729574
H	6.415309	2.187256	2.489586
H	6.515865	0.480861	2.914685
H	6.227299	0.958935	1.234585
H	5.173691	-2.584320	4.301350
H	5.718713	-0.911938	4.219612
H	4.089596	-1.284814	4.805377
H	5.790817	-2.984811	2.008687
H	5.067440	-2.123343	0.648449
H	6.290711	-1.329852	1.651632
H	3.618411	-3.569188	2.808346
H	2.367938	-2.397068	3.235297
H	2.769984	-2.676907	1.537690

M7b Singlet quinoidal

C	0.028395	0.084362	0.032923
C	-0.043726	0.310756	1.538627
C	1.149352	0.305006	2.171076
C	2.364299	0.074664	1.276220
C	2.199845	1.165389	0.224564
C	1.007233	1.168523	-0.408948
C	-1.263227	0.957520	2.163025
C	1.325768	0.970669	3.525451
C	3.083485	2.395808	0.245958
C	0.486744	2.415458	-1.103593
C	-0.562573	-0.900457	-0.711208
C	-0.238871	-1.116368	-2.243654
C	-1.610547	-1.903284	-0.056753
C	3.309458	-0.913327	1.338593
C	3.241002	-2.088844	2.394621
C	4.538186	-0.935691	0.328214
H	2.639972	3.160538	0.902545
H	4.083133	2.192385	0.629259
H	3.187513	2.853499	-0.744038
H	2.372554	0.990359	3.833024
H	0.981666	2.013536	3.475685
H	0.749202	0.485971	4.321945
H	-1.153190	2.053057	2.144579
H	-2.185099	0.724124	1.630740
H	-1.397282	0.672888	3.212545

H	0.481851	3.259457	-0.398996
H	1.102066	2.722049	-1.957618
H	-0.537237	2.284632	-1.457207
C	5.074202	-2.343002	-0.075986
C	4.251169	-0.302974	-1.062176
C	5.724139	-0.154086	0.963844
C	4.589277	-2.324223	3.134862
C	2.239754	-1.908423	3.561196
C	2.759080	-3.393023	1.692297
C	-1.639232	-3.350596	-0.635004
C	-1.388154	-2.178314	1.456270
C	-3.039986	-1.312130	-0.224279
C	-1.513571	-1.261208	-3.123808
C	0.536832	0.021251	-2.952140
C	0.691362	-2.355501	-2.407633
H	5.989857	-2.195348	-0.658796
H	5.330409	-3.004047	0.746971
H	4.364265	-2.864498	-0.725326
H	6.531809	-0.047665	0.229118
H	5.423204	0.847676	1.278897
H	6.138924	-0.660470	1.837345
H	5.085522	-0.548464	-1.729050
H	3.342055	-0.712295	-1.506661
H	4.171739	0.779497	-1.055524
H	2.679639	-4.196007	2.434842
H	1.764814	-3.237975	1.262767
H	3.408826	-3.749515	0.898124
H	2.263537	-2.821273	4.166893
H	2.517252	-1.085728	4.219512
H	1.213229	-1.766554	3.225828
H	4.464916	-3.153239	3.840844
H	5.434780	-2.574263	2.498827
H	4.861577	-1.437526	3.717853
H	-2.484860	-3.874197	-0.176020
H	-1.772630	-3.426310	-1.710649
H	-0.736010	-3.904645	-0.361763
H	-3.760455	-1.923655	0.332871
H	-3.100487	-0.290398	0.157387
H	-3.363418	-1.291366	-1.266615
H	-2.025105	-3.020440	1.750070
H	-0.353588	-2.459041	1.662209
H	-1.653813	-1.351311	2.106940
H	0.964255	-2.466145	-3.463804
H	1.613799	-2.203860	-1.838978
H	0.255860	-3.297202	-2.086084
H	0.733038	-0.299166	-3.981357
H	-0.043946	0.941006	-3.013913
H	1.498689	0.236979	-2.488684
H	-1.208507	-1.416862	-4.164806
H	-2.172651	-2.085056	-2.860863

H	-2.104444	-0.339196	-3.091315

M7b Singlet diradical

C	-0.006686	0.013244	-0.006355
C	0.002332	0.042183	1.407787
C	1.216641	-0.012942	2.123833
C	2.456255	-0.066762	1.444435
C	2.447967	-0.074553	0.029708
C	1.232323	-0.051288	-0.685916
C	-1.288425	0.133762	2.196416
C	1.151929	-0.010292	3.637916
C	3.740278	-0.110097	-0.760890
C	1.294047	-0.097957	-2.199492
C	-1.306697	0.048047	-0.770741
C	-1.963289	-1.311664	-1.146265
C	-1.905148	1.439291	-1.127111
C	3.754565	-0.117283	2.210566
C	4.299186	-1.514969	2.622856
C	4.463438	1.229739	2.529786
H	3.848641	-1.048746	-1.319919
H	3.780082	0.697978	-1.500708
H	4.611490	-0.012218	-0.115481
H	2.131957	-0.168660	4.084896
H	0.763257	0.940679	4.025431
H	0.483493	-0.794024	4.013324
H	-1.466378	-0.771559	2.791433
H	-1.268297	0.971053	2.904111
H	-2.149030	0.270589	1.544044
H	1.761639	0.804463	-2.614804
H	1.891768	-0.946918	-2.552032
H	0.303071	-0.185455	-2.641731
C	5.994292	1.197656	2.277205
C	3.898785	2.380030	1.656956
C	4.241858	1.673326	4.007876
C	4.853182	-1.561478	4.071558
C	3.190405	-2.595859	2.543300
C	5.433320	-2.004174	1.671338
C	-3.433192	1.536669	-0.876065
C	-1.248124	2.563454	-0.285469
C	-1.649093	1.823104	-2.616261
C	-2.517476	-1.351950	-2.595168
C	-0.944037	-2.475052	-1.035127
C	-3.134410	-1.683589	-0.186394
H	-3.772112	2.553457	-1.106435
H	-4.012306	0.854080	-1.501766
H	-3.684698	1.336331	0.170239
H	-1.667485	3.530108	-0.586553
H	-1.433074	2.441424	0.784768
H	-0.166329	2.605143	-0.435830
H	-2.088331	2.808105	-2.816727

H	-0.579333	1.892120	-2.828484
H	-2.092744	1.115561	-3.318133
H	-3.568557	-2.640583	-0.500560
H	-2.781863	-1.805265	0.840919
H	-3.934525	-0.941919	-0.188257
H	-2.917872	-2.351786	-2.799532
H	-3.328598	-0.639922	-2.763103
H	-1.732950	-1.156083	-3.332793
H	-1.446161	-3.418189	-1.278839
H	-0.106947	-2.357614	-1.727716
H	-0.534711	-2.563468	-0.025509
H	4.401574	3.315914	1.925941
H	4.065519	2.209080	0.590521
H	2.825506	2.519465	1.809963
H	4.757713	2.625838	4.180677
H	3.181404	1.832320	4.218987
H	4.629987	0.953177	4.729619
H	6.415388	2.187233	2.489700
H	6.515898	0.480834	2.914787
H	6.227378	0.958920	1.234682
H	5.173779	-2.584332	4.301336
H	5.718747	-0.911933	4.219660
H	4.089617	-1.284872	4.805352
H	5.791029	-2.984753	2.008763
H	5.067616	-2.123382	0.648483
H	6.290812	-1.329769	1.651657
H	3.618520	-3.569201	2.808279
H	2.368037	-2.397095	3.235243
H	2.770105	-2.676899	1.537631

M7c Triplet

C	-0.007153	-0.010289	-0.000440
C	0.015693	-0.014881	1.405746
C	1.216288	-0.004833	2.118833
C	2.461817	0.020094	1.466036
C	2.438917	0.030786	0.059828
C	1.238436	0.011371	-0.653287
H	-0.922878	-0.024403	1.953532
H	1.184487	-0.018538	3.205102
H	3.377177	0.057538	-0.487892
H	1.270572	0.011125	-1.739626
C	-1.292361	-0.027884	-0.764195
C	-1.987443	-1.397627	-0.950741
C	-1.818016	1.325070	-1.299521
C	3.746794	0.035751	2.230177
C	4.490819	-1.311112	2.393846
C	4.225606	1.398081	2.786027
C	5.749454	1.632557	2.619207
C	3.532650	2.570753	2.045755
C	3.859487	1.535982	4.292399

C	5.102560	-1.516014	3.803818
C	3.525246	-2.503597	2.173259
C	5.624016	-1.443412	1.334550
C	-3.350083	1.502768	-1.137708
C	-1.170794	2.509300	-0.536596
C	-1.449850	1.500552	-2.801609
C	-2.583955	-1.603337	-2.367134
C	-0.982100	-2.558682	-0.740954
C	-3.121381	-1.583948	0.099701
H	-3.643788	2.481740	-1.534142
H	-3.933109	0.750352	-1.671190
H	-3.641199	1.473191	-0.082516
H	-1.580519	3.452331	-0.915739
H	-1.379607	2.459057	0.536848
H	-0.085895	2.539630	-0.664047
H	-1.763147	2.492975	-3.149619
H	-0.367583	1.424284	-2.949664
H	-1.933050	0.755950	-3.437254
H	-3.556791	-2.586790	0.005592
H	-2.731756	-1.486110	1.118314
H	-3.926294	-0.857197	-0.026723
H	-3.046368	-2.595828	-2.422472
H	-3.354481	-0.874505	-2.624541
H	-1.803397	-1.560243	-3.133852
H	-1.492846	-3.514592	-0.902787
H	-0.147240	-2.499079	-1.446596
H	-0.565057	-2.567783	0.268993
H	3.911672	3.521369	2.437545
H	3.736004	2.544701	0.970436
H	2.448254	2.561557	2.180243
H	4.136776	2.534252	4.653984
H	2.781829	1.415739	4.444484
H	4.374321	0.800943	4.914103
H	6.010815	2.614845	3.029880
H	6.361563	0.892623	3.137285
H	6.034788	1.629653	1.562053
H	5.595999	-2.494208	3.843221
H	5.851727	-0.767023	4.066396
H	4.326072	-1.507292	4.575764
H	6.098097	-2.429682	1.415963
H	5.223903	-1.351140	0.319531
H	6.401597	-0.687620	1.462397
H	4.070563	-3.443249	2.316662
H	2.693920	-2.484373	2.885431
H	3.101033	-2.512267	1.166324

M7c Singlet diradical

C	-0.007718	-0.015515	0.000741
C	0.014246	-0.024147	1.406944

C	1.214650	-0.012228	2.120714
C	2.460380	0.014856	1.468389
C	2.438411	0.026410	0.062203
C	1.238063	0.008672	-0.651603
H	-0.924601	-0.035960	1.954328
H	1.182473	-0.026926	3.207038
H	3.376846	0.054958	-0.485179
H	1.270788	0.010799	-1.737964
C	-1.291908	-0.029525	-0.763620
C	-2.005411	-1.392802	-0.928878
C	-1.798918	1.321365	-1.322950
C	3.744180	0.033921	2.233256
C	4.506892	-1.305482	2.372498
C	4.207661	1.393376	2.809895
C	5.726747	1.650380	2.632230
C	3.493066	2.568754	2.094758
C	3.854621	1.502729	4.321613
C	5.130177	-1.521782	3.775683
C	3.553715	-2.506480	2.143115
C	5.635000	-1.411207	1.304941
C	-3.329252	1.519120	-1.167522
C	-1.140590	2.509243	-0.575090
C	-1.425793	1.470905	-2.826441
C	-2.598042	-1.613203	-2.344802
C	-1.015893	-2.563329	-0.697043
C	-3.146228	-1.548719	0.118889
H	-3.610344	2.495627	-1.578870
H	-3.919768	0.765967	-1.691602
H	-3.622876	1.508122	-0.112695
H	-1.540807	3.451237	-0.966729
H	-1.350712	2.474621	0.498731
H	-0.055390	2.527657	-0.701815
H	-1.728381	2.461316	-3.189337
H	-0.344160	1.381611	-2.971035
H	-1.915238	0.722142	-3.452335
H	-3.595694	-2.546309	0.035824
H	-2.759425	-1.443591	1.137804
H	-3.940253	-0.812306	-0.019826
H	-3.070963	-2.601356	-2.387329
H	-3.359436	-0.880033	-2.616596
H	-1.813708	-1.590060	-3.108507
H	-1.538181	-3.514961	-0.846816
H	-0.177167	-2.524873	-1.399658
H	-0.603180	-2.562896	0.314662
H	3.863744	3.517738	2.498237
H	3.686841	2.562109	1.017372
H	2.410149	2.544034	2.238472
H	4.120900	2.499497	4.695325
H	2.780526	1.364001	4.482591
H	4.386513	0.766515	4.927281

H	5.979311	2.629925	3.054821
H	6.353245	0.910926	3.133524
H	6.001471	1.666544	1.572417
H	5.634378	-2.494972	3.798973
H	5.872982	-0.768193	4.043054
H	4.358856	-1.531526	4.552712
H	6.123164	-2.391522	1.373863
H	5.227568	-1.315009	0.293299
H	6.402980	-0.646014	1.435262
H	4.110307	-3.441549	2.272633
H	2.726128	-2.504758	2.859980
H	3.123868	-2.508461	1.138592

M7d Triplet

C	-0.038377	-0.001742	-0.019306
C	0.009795	0.089460	1.401430
C	1.222868	-0.090658	2.118800
C	2.492579	0.032841	1.485104
C	2.440652	0.142260	0.065582
C	1.234900	-0.071572	-0.654524
C	-1.257420	0.475175	2.182174
C	1.148782	-0.506158	3.596838
C	3.693370	0.583370	-0.709186
C	1.324276	-0.463521	-2.138403
C	-1.351157	-0.026027	-0.798727
C	-2.196153	-1.345709	-0.780913
C	-1.778185	1.267728	-1.573997
C	3.805967	0.042523	2.264556
C	4.695498	-1.247255	2.226491
C	4.189366	1.338836	3.056793
F	4.485818	-0.425219	-1.135821
F	3.363391	1.313735	-1.798358
F	4.473366	1.398894	0.033020
F	2.186264	-1.299644	3.940646
F	1.139001	0.521495	4.475386
F	0.046417	-1.248059	3.844884
F	-2.009242	-0.566266	2.603189
F	-0.952444	1.208981	3.276456
F	-2.070796	1.264885	1.448392
F	1.292524	0.575856	-3.002489
F	2.455392	-1.157351	-2.396634
F	0.319601	-1.293511	-2.493895
C	5.638740	1.816861	2.747352
C	3.237293	2.510036	2.699934
C	4.151370	1.189814	4.611124
C	5.130121	-1.715691	3.647376
C	3.931385	-2.429484	1.574945
C	6.036767	-1.093973	1.441230
C	-3.241889	1.695381	-1.258735

C	-0.864476	2.464654	-1.202424
C	-1.737115	1.138640	-3.130003
C	-2.619165	-1.799343	-2.209832
C	-1.391507	-2.513677	-0.153382
C	-3.537710	-1.252884	0.012637
H	-3.420995	2.671334	-1.723298
H	-3.966808	1.002389	-1.690769
H	-3.445903	1.790756	-0.193119
H	-1.223388	3.360730	-1.718375
H	-0.875294	2.677578	-0.130631
H	0.171114	2.310046	-1.514086
H	-1.971370	2.121383	-3.555033
H	-0.766651	0.840890	-3.515363
H	-2.483203	0.440488	-3.507199
H	-4.016914	-2.238000	-0.022707
H	-3.403231	-0.995374	1.058949
H	-4.233611	-0.540909	-0.428627
H	-3.068073	-2.795743	-2.133712
H	-3.381310	-1.141466	-2.632738
H	-1.787119	-1.861008	-2.910232
H	-1.992335	-3.427256	-0.202311
H	-0.457451	-2.706183	-0.686608
H	-1.158714	-2.342060	0.900361
H	3.563569	3.409615	3.231243
H	3.244685	2.739627	1.631684
H	2.206295	2.315559	3.004351
H	4.374180	2.170187	5.047670
H	3.184513	0.876406	4.993491
H	4.905680	0.496136	4.979970
H	5.783416	2.797206	3.214632
H	6.385708	1.148128	3.180057
H	5.842808	1.921500	1.682659
H	5.623776	-2.688895	3.550129
H	5.859709	-1.035392	4.091340
H	4.297593	-1.831731	4.340139
H	6.531156	-2.071999	1.427843
H	5.902015	-0.784399	0.409294
H	6.720700	-0.395434	1.921588
H	4.559495	-3.324960	1.614137
H	3.000447	-2.658842	2.099221
H	3.699960	-2.248601	0.522481

M7d Singlet quinoidal

C	-0.040803	0.009174	0.126820
C	0.044407	0.056652	1.658647
C	1.317868	0.083939	2.116760
C	2.402090	0.073343	1.048454
C	2.021587	1.302577	0.212743
C	0.754618	1.248053	-0.261338
C	-1.155224	0.521003	2.485654

C	1.750463	0.621461	3.483148
C	2.811375	2.605365	0.350103
C	-0.081119	2.462357	-0.673406
C	-0.553894	-0.943974	-0.714374
C	-0.232699	-0.949294	-2.268503
C	-1.473145	-2.120391	-0.181602
C	3.360661	-0.885956	0.850355
C	3.354638	-2.252415	1.656299
C	4.513531	-0.706755	-0.221348
F	2.130090	3.531811	1.060372
F	3.977928	2.411735	1.010393
F	3.122280	3.184751	-0.836435
F	3.072865	0.431741	3.712842
F	1.568061	1.963000	3.540337
F	1.083463	0.078034	4.523165
F	-0.989606	1.780750	2.946936
F	-2.290518	0.539594	1.747735
F	-1.403906	-0.255289	3.569985
F	-0.400747	3.204250	0.414727
F	0.537234	3.278620	-1.552584
F	-1.268466	2.108011	-1.223545
C	4.956052	-1.988719	-0.988228
C	4.179999	0.269864	-1.375995
C	5.765995	-0.132624	0.508520
C	4.756509	-2.667422	2.186800
C	2.473479	-2.346620	2.929488
C	2.766834	-3.352349	0.720284
C	-1.300180	-3.505693	-0.872449
C	-1.274163	-2.466508	1.314421
C	-2.962894	-1.692287	-0.356317
C	-1.486680	-1.186061	-3.155587
C	0.385880	0.330814	-2.889966
C	0.850024	-2.039909	-2.535131
H	5.839258	-1.724648	-1.578786
H	5.235866	-2.836927	-0.370355
H	4.187956	-2.316733	-1.695255
H	6.504916	0.164713	-0.244460
H	5.514784	0.748184	1.099077
H	6.243426	-0.858824	1.165651
H	4.936984	0.147463	-2.157195
H	3.212985	0.057334	-1.834843
H	4.223802	1.314484	-1.094116
H	2.770027	-4.309757	1.253004
H	1.729747	-3.113198	0.471380
H	3.304867	-3.495969	-0.211552
H	2.501858	-3.387989	3.266212
H	2.853396	-1.744917	3.751672
H	1.424312	-2.111207	2.762340
H	4.654620	-3.619396	2.718149
H	5.520696	-2.810373	1.427857

H	5.125858	-1.930644	2.906675
H	-2.087981	-4.160469	-0.486514
H	-1.395898	-3.506649	-1.954428
H	-0.344382	-3.968534	-0.609068
H	-3.597043	-2.404359	0.184316
H	-3.148227	-0.699998	0.054173
H	-3.284393	-1.695511	-1.397631
H	-1.777018	-3.417589	1.517036
H	-0.222732	-2.601570	1.572838
H	-1.724162	-1.752049	1.992482
H	1.061327	-2.071113	-3.609803
H	1.779094	-1.776967	-2.022666
H	0.576780	-3.044346	-2.228402
H	0.649736	0.093144	-3.925513
H	-0.313168	1.162386	-2.931238
H	1.306466	0.653220	-2.408080
H	-1.174558	-1.165291	-4.204914
H	-1.997489	-2.132263	-2.997576
H	-2.212711	-0.379527	-3.015258

M7d Singlet diradical

C	-0.038446	-0.001493	-0.019227
C	0.009709	0.090051	1.401465
C	1.222823	-0.090127	2.118913
C	2.492534	0.033190	1.485266
C	2.440599	0.142612	0.065770
C	1.234827	-0.071447	-0.654353
C	-1.256795	0.478065	2.182240
C	1.148285	-0.507461	3.596417
C	3.692484	0.585913	-0.709111
C	1.324785	-0.465795	-2.137565
C	-1.350980	-0.026034	-0.798580
C	-2.197592	-1.344655	-0.777845
C	-1.776523	1.266379	-1.576867
C	3.805742	0.042575	2.264554
C	4.696802	-1.246087	2.223423
C	4.187911	1.337485	3.059613
F	4.487686	-0.420763	-1.135020
F	3.360888	1.314701	-1.798887
F	4.470062	1.404142	0.032872
F	2.184782	-1.303086	3.938625
F	1.139915	0.518579	4.476802
F	0.044872	-1.248252	3.843327
F	-2.011153	-0.561560	2.603091
F	-0.950169	1.210888	3.276770
F	-2.068152	1.270015	1.448449
F	1.291639	0.571574	-3.003933
F	2.456972	-1.158522	-2.394337
F	0.321265	-1.298129	-2.491167
C	5.636408	1.817740	2.749415

C	3.234382	2.508354	2.705555
C	4.151750	1.185458	4.613682
C	5.129914	-1.718222	3.643562
C	3.934074	-2.427234	1.568280
C	6.038971	-1.089374	1.440414
C	-3.239395	1.696489	-1.261015
C	-0.861219	2.462971	-1.208162
C	-1.737082	1.134114	-3.132645
C	-2.619289	-1.801394	-2.206204
C	-1.394368	-2.511917	-0.147151
C	-3.540025	-1.248815	0.013823
H	-3.417488	2.672089	-1.726712
H	-3.965623	1.003968	-1.691551
H	-3.442341	1.793494	-0.195337
H	-1.219605	3.358549	-1.725342
H	-0.871029	2.677631	-0.136694
H	0.173956	2.306783	-1.520293
H	-1.968926	2.116674	-3.559408
H	-0.767865	0.832772	-3.518367
H	-2.485543	0.437405	-3.507819
H	-4.018199	-2.234590	-0.016554
H	-3.407162	-0.985190	1.058809
H	-4.236164	-0.540107	-0.432338
H	-3.069273	-2.797163	-2.128160
H	-3.380220	-1.143848	-2.631756
H	-1.786332	-1.865610	-2.905301
H	-1.995594	-3.425273	-0.195219
H	-0.459806	-2.705764	-0.679054
H	-1.162679	-2.338645	0.906533
H	3.560475	3.407471	3.237752
H	3.240478	2.739341	1.637588
H	2.203889	2.312617	3.010742
H	4.371679	2.165746	5.051873
H	3.186380	0.867985	4.996446
H	4.908803	0.493689	4.980526
H	5.780361	2.797558	3.218016
H	6.384638	1.149283	3.180292
H	5.839149	1.924169	1.684637
H	5.625550	-2.690189	3.543991
H	5.857443	-1.038054	4.091017
H	4.296310	-1.838062	4.334396
H	6.531056	-2.068407	1.418806
H	5.906040	-0.770174	0.411198
H	6.724189	-0.396832	1.927652
H	4.562211	-3.322703	1.607031
H	3.002293	-2.657534	2.090710
H	3.704371	-2.244621	0.515773

M7e Triplet

C	-0.027305	-0.001976	-0.010439

C	0.004371	-0.006045	1.402021
C	1.223003	0.009978	2.126769
C	2.479151	0.034105	1.480571
C	2.447469	0.046784	0.068266
C	1.228799	0.025672	-0.656587
C	-1.227777	-0.024034	2.137885
C	1.165651	-0.003949	3.560788
C	3.678640	0.090889	-0.668215
C	1.287779	0.023597	-2.090594
C	-1.311801	-0.027118	-0.775481
C	-1.849104	-1.418825	-1.208201
C	-1.985906	1.340985	-1.069628
C	3.763130	0.042358	2.246964
C	4.350423	-1.337880	2.651212
C	4.394820	1.427073	2.556781
C	5.919855	1.462209	2.281660
C	3.762967	2.546789	1.690037
C	4.152707	1.836234	4.040268
C	4.899148	-1.355468	4.100104
C	3.280822	-2.458052	2.572065
C	5.496553	-1.761527	1.685199
C	-3.517502	1.317466	-0.834624
C	-1.418337	2.462860	-0.162051
C	-1.720286	1.791237	-2.537168
C	-2.361806	-1.433835	-2.670646
C	-0.750399	-2.509074	-1.114829
C	-3.004748	-1.886516	-0.274112
H	-3.919324	2.317594	-1.031179
H	-4.036248	0.625045	-1.500234
H	-3.761432	1.055651	0.198736
H	-1.899433	3.409470	-0.427166
H	-1.621131	2.283416	0.897364
H	-0.340716	2.601818	-0.293990
H	-2.193047	2.767102	-2.698419
H	-0.652447	1.900456	-2.740524
H	-2.131195	1.097304	-3.270388
H	-3.344648	-2.876314	-0.600509
H	-2.675623	-1.972083	0.764184
H	-3.863217	-1.215534	-0.298467
H	-2.687361	-2.449874	-2.920010
H	-3.218011	-0.774485	-2.826458
H	-1.573200	-1.153190	-3.374330
H	-1.185663	-3.473762	-1.393356
H	0.082706	-2.321850	-1.797892
H	-0.354172	-2.616455	-0.100145
H	4.215914	3.504659	1.963932
H	3.942779	2.398748	0.621667
H	2.684881	2.642314	1.853460
H	4.584178	2.830148	4.206850
H	3.087652	1.890117	4.277413

H	4.619005	1.149565	4.746499
H	6.291901	2.472297	2.485922
H	6.479937	0.777025	2.920983
H	6.144607	1.226523	1.237813
H	5.256842	-2.365444	4.329053
H	5.741929	-0.676124	4.242641
H	4.121313	-1.103857	4.826333
H	5.877809	-2.740830	1.997117
H	5.144273	-1.853623	0.655138
H	6.331646	-1.061071	1.691679
H	3.748270	-3.412290	2.834237
H	2.457294	-2.298510	3.273542
H	2.866437	-2.569317	1.565086
N	1.092356	-0.021922	4.721509
N	-2.212654	-0.030199	2.756683
N	4.662013	0.139297	-1.287513
N	1.363190	0.012366	-3.251252

M7e Singlet quinoidal

C	-0.073036	-0.254454	0.002894
C	0.118034	-0.283588	1.503727
C	1.418945	-0.289346	1.925753
C	2.538355	-0.150012	0.932054
C	2.084821	0.988664	0.045769
C	0.813097	0.874619	-0.444281
C	-0.904229	-0.088703	2.484906
C	1.750359	-0.109282	3.306983
C	2.766834	2.232633	-0.134591
C	0.183692	1.962547	-1.129696
C	-0.840473	-1.067683	-0.798335
C	-0.595220	-1.150855	-2.348060
C	-2.009173	-1.898357	-0.138216
C	3.676971	-0.917573	0.857498
C	3.781987	-2.297220	1.601654
C	4.910346	-0.372566	0.036111
C	6.168131	-1.273266	-0.123884
C	4.531813	-0.049732	-1.436023
C	5.442747	0.871781	0.811107
C	4.724457	-2.186938	2.829737
C	2.439941	-2.884851	2.116860
C	4.295941	-3.396313	0.613596
C	-2.837289	-2.873062	-1.022841
C	-1.492935	-2.822982	0.999700
C	-3.065881	-0.861548	0.351839
C	-1.719521	-0.413777	-3.123688
C	0.755797	-0.565570	-2.841894
C	-0.520269	-2.648168	-2.796390
H	6.959826	-0.634500	-0.528107
H	6.546907	-1.684404	0.811269
H	6.024950	-2.084423	-0.838514

H	6.174821	1.404748	0.195382
H	4.674837	1.583986	1.100737
H	5.947786	0.547675	1.727018
H	5.417054	0.329763	-1.956469
H	4.213472	-0.964514	-1.947529
H	3.750470	0.692237	-1.567824
H	3.963803	-4.374275	0.971143
H	3.883124	-3.264563	-0.392121
H	5.378083	-3.444055	0.539092
H	2.635070	-3.904494	2.461722
H	2.018654	-2.364422	2.972966
H	1.688772	-2.944454	1.324253
H	4.848547	-3.178856	3.278866
H	5.717694	-1.813688	2.576845
H	4.299652	-1.525248	3.589368
H	-3.709286	-3.168227	-0.430479
H	-3.219824	-2.426048	-1.939817
H	-2.300651	-3.789939	-1.267988
H	-3.805533	-1.360047	0.986812
H	-2.653368	-0.030229	0.916471
H	-3.593647	-0.439097	-0.509655
H	-2.341755	-3.360871	1.434022
H	-0.803548	-3.569093	0.589528
H	-0.987317	-2.321919	1.819415
H	0.043794	-2.707085	-3.730602
H	0.006234	-3.267235	-2.062312
H	-1.491120	-3.093391	-2.990524
H	0.872655	-0.839702	-3.894408
H	0.814815	0.519552	-2.815966
H	1.605669	-0.985724	-2.296865
H	-1.570772	-0.562060	-4.199245
H	-2.718800	-0.776057	-2.877488
H	-1.692748	0.661106	-2.926578
N	-1.691655	0.094435	3.323152
N	2.068372	0.054176	4.415283
N	-0.373817	2.826790	-1.675966
N	3.267770	3.272517	-0.289311

M7e Singlet diradical

C	-0.027298	-0.002413	-0.009986
C	0.004749	-0.006990	1.402372
C	1.223928	0.008487	2.126649
C	2.479513	0.033139	1.479539
C	2.447464	0.045706	0.067313
C	1.228269	0.024821	-0.657050
C	-1.227191	-0.026938	2.138770
C	1.167258	-0.005373	3.560817
C	3.678575	0.086789	-0.669672
C	1.286393	0.024234	-2.091213
C	-1.312047	-0.027011	-0.774185

C	-1.847146	-1.417924	-1.212102
C	-1.988667	1.340945	-1.063068
C	3.763850	0.041971	2.244883
C	4.348817	-1.337434	2.655242
C	4.397396	1.426911	2.549919
C	5.923525	1.458863	2.281089
C	3.770913	2.544319	1.676344
C	4.149484	1.842237	4.030802
C	4.893474	-1.351638	4.105725
C	3.279422	-2.457704	2.575352
C	5.497328	-1.763533	1.693040
C	-3.520423	1.314557	-0.829719
C	-1.423457	2.460930	-0.151725
C	-1.722004	1.796130	-2.528967
C	-2.359727	-1.429406	-2.674598
C	-0.747737	-2.507646	-1.121281
C	-3.002410	-1.889141	-0.279174
H	-3.923450	2.314715	-1.023615
H	-4.037520	0.623485	-1.498004
H	-3.765095	1.049284	0.202584
H	-1.905499	3.407622	-0.414819
H	-1.627203	2.278456	0.906947
H	-0.345885	2.601596	-0.282218
H	-2.196067	2.771754	-2.687820
H	-0.654038	1.907628	-2.730596
H	-2.130855	1.103773	-3.264810
H	-3.340709	-2.878832	-0.607548
H	-2.673337	-1.976160	0.759045
H	-3.861911	-1.219465	-0.302458
H	-2.684488	-2.445020	-2.926709
H	-3.216438	-0.770318	-2.828751
H	-1.571290	-1.146306	-3.377482
H	-1.182624	-3.471941	-1.401732
H	0.085104	-2.318721	-1.804140
H	-0.351277	-2.616939	-0.106897
H	4.223913	3.502505	1.949065
H	3.955543	2.392368	0.609384
H	2.692244	2.641844	1.834548
H	4.583705	2.835175	4.196082
H	3.083418	1.900950	4.262352
H	4.609545	1.156251	4.741775
H	6.296037	2.469215	2.483135
H	6.480060	0.775376	2.925285
H	6.152339	1.219123	1.239046
H	5.250872	-2.360969	4.337969
H	5.735588	-0.671665	4.249211
H	4.113463	-1.098710	4.829166
H	5.876330	-2.743051	2.007022
H	5.147852	-1.856032	0.662028
H	6.333389	-1.064304	1.701332

H	3.746412	-3.411451	2.840081
H	2.454248	-2.297375	3.274644
H	2.867507	-2.570512	1.567534
N	1.093720	-0.022900	4.721533
N	-2.211418	-0.035629	2.758583
N	4.661523	0.131312	-1.289951
N	1.361771	0.015103	-3.251897

Table S8. Cartesian coordinates of the AIM critical points, bonding features, density (ρ in a.u.), Laplacian of the density ($\nabla^2\rho$ in a.u.), ellipticity (ε), energy density (H in hartree), and signature of the AIM critical points for the triplet state and the quinoidal singlet states of compounds M1 – M5 and M7. Atom numbering according Table S6.

bcp	X	Y	Z	bonding	ρ	$\nabla^2\rho$	ε	H	Signature
M1a Triplet state									
XX	0.510116	1.132228	0.027606	C 2 C 1	0.289311	-0.742828	0.185720	-0.266932	(3 -1)
XX	-0.005331	0.007916	-0.002265	RING	0.017917	0.137803	-1.173312	0.008034	(3 1)
XX	1.224857	0.098714	-0.018408	C 3 C 2	0.318277	-0.867352	0.351796	-0.323571	(3 -1)
XX	0.705601	-1.000941	-0.002611	C 4 C 3	0.289742	-0.732289	0.225464	-0.268168	(3 -1)
XX	-0.506263	-1.131048	0.010784	C 5 C 4	0.300547	-0.784923	0.232049	-0.289437	(3 -1)
XX	-0.684263	1.048324	0.013525	C 6 C 1	0.300629	-0.790915	0.229840	-0.290625	(3 -1)
XX	-1.221955	-0.104089	-0.017136	C 6 C 5	0.298480	-0.788714	0.231427	-0.282744	(3 -1)
XX	1.597479	1.106329	-0.013233	N 7 C 2	0.302981	-0.984320	0.089876	-0.419015	(3 -1)
XX	2.523114	0.242595	-0.145440	RING	0.019148	0.108153	-1.763027	0.005258	(3 1)
XX	1.794188	-0.801123	-0.052111	N 8 C 3	0.277490	-0.819732	0.045672	-0.340728	(3 -1)
XX	-1.178875	-2.451005	0.052227	RING	0.012291	0.065548	-1.862058	0.003805	(3 1)
XX	-1.606972	-1.085253	-0.012405	N 9 C 5	0.293614	-0.912395	0.098988	-0.405028	(3 -1)
XX	-0.185438	2.154163	0.109537	C 10 C 1	0.291726	-0.744137	0.184861	-0.273486	(3 -1)
XX	-1.595854	2.256311	-0.040334	RING	0.011561	0.062977	-2.025424	0.003789	(3 1)
XX	0.310104	3.244897	0.287343	H 11 C 10	0.284208	-1.011641	0.042970	-0.298669	(3 -1)
XX	-1.744339	0.807621	-0.059571	H 12 N 9	0.304061	-0.956162	0.092234	-0.443468	(3 -1)
XX	-0.836015	3.170724	0.205853	H 12 C 10	0.281913	-0.984902	0.041612	-0.294043	(3 -1)
XX	0.182727	-2.134525	0.068102	C 13 C 4	0.290931	-0.744597	0.169448	-0.271776	(3 -1)
XX	0.863387	-3.136844	0.143766	H 14 C 13	0.284116	-1.009975	0.042639	-0.298431	(3 -1)
XX	-0.281607	-3.251611	0.182316	H 15 C 13	0.282072	-0.985626	0.041469	-0.294298	(3 -1)
XX	2.932867	1.172130	-0.181401	H 16 N 7	0.339956	-1.805155	0.044331	-0.504994	(3 -1)
XX	2.862822	0.208317	-0.190413	H 16 N 8	0.020530	0.072649	0.423535	0.001605	(3 -1)

XX 2.267844 2.227424 -0.395726 H 17 N 7 0.342153 -1.778836 0.048821 -0.503373 (3 -1)
XX 2.859457 -1.366972 0.564213 H 18 N 8 0.337398 -1.725615 0.052561 -0.491500 (3 -1)
XX 2.661708 -1.680985 -0.596332 H 19 N 8 0.340716 -1.755680 0.051065 -0.498583 (3 -1)
XX -2.861762 -1.344778 0.480882 H 20 N 9 0.338334 -1.758240 0.049796 -0.496996 (3 -1)
XX -2.280196 -2.282052 -0.097295 H 21 N 9 0.345261 -1.814340 0.047182 -0.510355 (3 -1)
XX -1.350773 -2.803880 0.056341 H 21 H 15 0.013215 0.055099 0.620652 0.002650 (3 -1)
XX -1.784562 2.516832 -0.052909 H 23 H 12 0.012032 0.053835 0.779042 0.002880 (3 -1)
XX -2.612891 1.863254 -0.281752 H 23 N 22 0.344766 -1.817472 0.046298 -0.510344 (3 -1)
XX -3.055919 0.693384 -0.458517 H 24 N 22 0.340826 -1.798733 0.047760 -0.504773 (3 -1)

M1a Singlet state
XX -0.464104 -1.144698 0.018930 C 2 C 1 0.273044 -0.687113 0.100128 -0.238342 (3 -1)
XX -0.000032 -0.000114 -0.007040 RING 0.017337 0.132517 -1.149414 0.007716 (3 1)
XX -1.245315 -0.155884 -0.046026 C 3 C 2 0.332535 -0.935905 0.408103 -0.354303 (3 -1)
XX -0.771077 0.965451 0.030399 C 4 C 3 0.277506 -0.691680 0.156440 -0.245965 (3 -1)
XX 0.464028 1.144492 0.018890 C 5 C 4 0.273117 -0.687476 0.100164 -0.238464 (3 -1)
XX 0.771029 -0.965687 0.029657 C 6 C 1 0.277489 -0.691576 0.156461 -0.245937 (3 -1)
XX 1.245192 0.155709 -0.046734 C 6 C 5 0.332497 -0.935688 0.408059 -0.354224 (3 -1)
XX -1.553431 -1.172588 -0.097926 N 7 C 2 0.305699 -0.995652 0.090365 -0.432352 (3 -1)
XX -2.567155 -0.363946 -0.265419 RING 0.018113 0.097676 -2.022703 0.004735 (3 1)
XX -1.849829 0.705400 -0.059631 N 8 C 3 0.281963 -0.849708 0.060789 -0.353932 (3 -1)
XX 1.553226 1.172497 -0.098018 N 9 C 5 0.305716 -0.995700 0.090289 -0.432434 (3 -1)
XX 2.567221 0.364227 -0.265364 RING 0.018113 0.097677 -2.023582 0.004735 (3 1)
XX 1.849831 -0.705430 -0.060365 N 10 C 6 0.281977 -0.849784 0.060578 -0.353935 (3 -1)
XX 0.288072 -2.103394 0.201501 C 11 C 1 0.330816 -0.924577 0.304613 -0.353256 (3 -1)
XX -0.157334 -3.178542 0.385882 H 12 C 11 0.283886 -1.013262 0.028354 -0.297471 (3 -1)
XX 0.987097 -3.031481 0.382404 H 13 C 11 0.282821 -1.002803 0.028146 -0.295328 (3 -1)
XX -0.287920 2.103182 0.201482 C 14 C 4 0.330801 -0.924474 0.304604 -0.353221 (3 -1)
XX -0.986623 3.031682 0.382579 H 15 C 14 0.282793 -1.002600 0.028155 -0.295266 (3 -1)
XX 0.157773 3.178407 0.384670 H 16 C 14 0.283877 -1.013114 0.028362 -0.297448 (3 -1)

XX 3.007567 -1.061823 0.548570 H 17 N 10 0.336898 -1.727213 0.051986 -0.491350 (3 -1)
XX 2.714449 -1.651684 -0.482697 H 18 N 10 0.341787 -1.771905 0.049296 -0.501678 (3 -1)
XX 2.870847 1.334544 -0.370652 H 19 N 9 0.339632 -1.802036 0.044768 -0.504007 (3 -1)
XX 2.829757 0.349946 -0.323001 H 19 N 10 0.018717 0.070213 0.677613 0.002017 (3 -1)
XX 2.114271 2.343402 -0.520051 H 20 C 5 0.342594 -1.790085 0.048626 -0.505130 (3 -1)
XX -3.008666 1.061902 0.547904 H 21 N 8 0.336912 -1.727357 0.052020 -0.491382 (3 -1)
XX -2.713606 1.652015 -0.482804 H 22 N 8 0.341819 -1.772204 0.049321 -0.501755 (3 -1)
XX -2.871061 -1.334204 -0.370516 H 23 N 7 0.339610 -1.801826 0.044756 -0.503958 (3 -1)
XX -2.829890 -0.349558 -0.323121 H 23 N 8 0.018719 0.070191 0.676626 0.002014 (3 -1)
XX -2.114657 -2.342732 -0.521559 H 24 C 2 0.342592 -1.790063 0.048613 -0.505128 (3 -1)

M1b Triplet state
XX 0.619119 -1.059078 -0.021777 C 2 C 1 0.295803 -0.762631 0.183717 -0.278660 (3 -1)
XX 0.000359 0.000068 -0.000708 RING 0.018609 0.143954 -1.175168 0.008309 (3 1)
XX 1.239015 -0.000153 -0.001141 C 3 C 2 0.306768 -0.806613 0.230811 -0.299955 (3 -1)
XX 0.619588 1.058985 0.019989 C 4 C 3 0.295775 -0.762460 0.183731 -0.278606 (3 -1)
XX -0.618525 1.059344 -0.021853 C 5 C 4 0.296013 -0.763558 0.184297 -0.279051 (3 -1)
XX -0.618983 -1.058984 0.020312 C 6 C 1 0.296003 -0.763518 0.184231 -0.279030 (3 -1)
XX -1.238148 0.000282 -0.000922 C 6 C 5 0.306703 -0.806593 0.229819 -0.299799 (3 -1)
XX 1.888023 -1.096796 -0.097803 C 7 C 2 0.250220 -0.581069 0.033482 -0.203135 (3 -1)
XX 2.703618 -0.000813 -0.000372 RING 0.013125 0.070847 -1.649491 0.004262 (3 1)
XX 1.888456 1.096132 0.096498 C 8 C 3 0.250273 -0.581308 0.033495 -0.203216 (3 -1)
XX -1.450196 2.475299 -0.205205 RING 0.012626 0.062609 -1.665436 * 0.003709 (3 1)
XX -1.887880 1.095692 -0.099365 C 9 C 5 0.250238 -0.581170 0.033315 -0.203161 (3 -1)
XX -2.707371 0.000845 -0.001436 RING 0.013131 0.070629 -1.668366 0.004242 (3 1)
XX 0.000626 2.131221 0.001226 C 11 C 1 0.285880 -0.726205 0.144731 -0.261979 (3 -1)
XX -1.888223 -1.095011 0.098209 C 10 C 6 0.250264 -0.581277 0.033404 -0.203202 (3 -1)
XX -0.000213 -2.131050 -0.000169 C 14 C 4 0.285908 -0.726323 0.144800 -0.262029 (3 -1)
XX -0.578239 -3.210325 -0.043242 H 12 C 11 0.286272 -1.027709 0.034964 -0.302151 (3 -1)
XX 0.577323 -3.210572 0.044849 H 13 C 11 0.286259 -1.027624 0.034954 -0.302117 (3 -1)

XX 0.578603 3.210656 -0.040242 H 15 C 14 0.286285 -1.027807 0.034966 -0.302173 (3 -1)
XX -0.576787 3.210663 0.049596 H 16 C 14 0.286247 -1.027507 0.034984 -0.302097 (3 -1)
XX -3.042621 1.090562 -0.366889 H 17 C 9 0.280383 -0.970855 0.009467 -0.289092 (3 -1)
XX -2.707256 1.777594 0.425650 H 18 C 9 0.272111 -0.911072 0.010741 -0.273747 (3 -1)
XX -2.459929 1.982705 -0.626456 H 19 C 9 0.277146 -0.947813 0.009159 -0.283019 (3 -1)
XX -1.465887 2.499952 -0.212529 H 19 H 16 0.012626 0.061751 11.887566 0.003672 (3 -1)
XX 2.462444 1.976188 0.632493 H 20 C 8 0.276953 -0.946348 0.009185 -0.282660 (3 -1)
XX 3.173710 -0.001082 -0.000730 H 21 C 3 0.014807 0.060897 0.351314 0.003047 (3 -1)
XX 3.046453 1.091509 0.350883 H 21 C 8 0.280511 -0.971774 0.009467 -0.289338 (3 -1)
XX 2.698632 1.790709 -0.425557 H 22 C 8 0.272213 -0.911768 0.010733 -0.273926 (3 -1)
XX 3.046083 -1.093298 -0.352491 H 23 C 7 0.280493 -0.971633 0.009468 -0.289302 (3 -1)
XX 2.698110 -1.789711 0.426333 H 24 C 7 0.272222 -0.911828 0.010706 -0.273947 (3 -1)
XX 2.461892 -1.978691 -0.631188 H 25 C 7 0.276978 -0.946550 0.009179 -0.282707 (3 -1)
XX -3.044127 -1.088824 0.361189 H 26 C 10 0.280406 -0.971059 0.009461 -0.289126 (3 -1)
XX -3.161040 0.001208 -0.002912 H 26 H 17 0.014623 0.060821 0.361625 0.003119 (3 -1)
XX -2.704865 -1.782173 -0.424243 H 27 C 10 0.272138 -0.911260 0.010752 -0.273792 (3 -1)
XX -2.461195 -1.978188 0.630311 H 28 C 10 0.277049 -0.947089 0.009163 -0.282839 (3 -1)

M1b Singlet state
XX 0.638859 1.051917 0.000022 C 2 C 1 0.270653 -0.665607 0.095243 -0.234376 (3 -1)
XX -0.000011 -0.000003 -0.000102 RING 0.017458 0.131654 -1.177596 0.007689 (3 1)
XX 1.277123 -0.000100 -0.000224 C 3 C 2 0.330432 -0.910795 0.331474 -0.349824 (3 -1)
XX 0.638289 -1.051996 -0.000226 C 4 C 3 0.270621 -0.665450 0.095227 -0.234323 (3 -1)
XX -0.638870 -1.051923 -0.000060 C 5 C 4 0.270639 -0.665536 0.095239 -0.234352 (3 -1)
XX -0.638290 1.051998 -0.000144 C 6 C 1 0.270614 -0.665414 0.095229 -0.234311 (3 -1)
XX -1.277142 0.000097 -0.000265 C 6 C 5 0.330427 -0.910763 0.331480 -0.349813 (3 -1)
XX 1.915852 1.100016 0.000144 C 7 C 2 0.251581 -0.587543 0.033330 -0.205265 (3 -1)
XX 2.712543 -0.000648 0.001378 RING 0.013136 0.073611 -1.541288 0.004496 (3 1)
XX 1.915088 -1.100595 -0.000448 C 8 C 3 0.251636 -0.587796 0.033348 -0.205348 (3 -1)
XX -1.915856 -1.100021 0.000078 C 9 C 5 0.251598 -0.587627 0.033331 -0.205292 (3 -1)

XX -2.712618 0.000660 -0.002136 RING 0.013134 0.073599 -1.541432 0.004496 (3 1)
XX -0.000304 -2.111983 0.000227 C 14 C 4 0.331377 -0.927991 0.297256 -0.353073 (3 -1)
XX -1.915093 1.100591 -0.000517 C 10 C 6 0.251639 -0.587812 0.033351 -0.205352 (3 -1)
XX 0.000331 2.111985 0.000292 C 11 C 1 0.331370 -0.927954 0.297255 -0.353059 (3 -1)
XX -0.574508 3.148954 0.000802 H 12 C 11 0.286168 -1.030457 0.021428 -0.301428 (3 -1)
XX 0.575180 3.148970 0.000428 H 13 C 11 0.286158 -1.030392 0.021423 -0.301411 (3 -1)
XX 0.574549 -3.148959 0.000299 H 15 C 14 0.286164 -1.030427 0.021428 -0.301419 (3 -1)
XX -0.575151 -3.148937 0.001046 H 16 C 14 0.286159 -1.030396 0.021424 -0.301413 (3 -1)
XX -3.101491 -1.134683 -0.000417 H 17 C 9 0.281656 -0.979816 0.008976 -0.291589 (3 -1)
XX -2.560267 -1.919568 -0.548601 H 18 C 9 0.274586 -0.929304 0.009535 -0.278319 (3 -1)
XX -2.560633 -1.917983 0.550501 H 19 C 9 0.274561 -0.929134 0.009526 -0.278277 (3 -1)
XX 2.560419 -1.917624 -0.551655 H 20 C 8 0.274538 -0.928964 0.009530 -0.278236 (3 -1)
XX 3.100821 -1.136309 0.002376 H 21 C 8 0.281647 -0.979736 0.008982 -0.291573 (3 -1)
XX 2.557578 -1.921911 0.547419 H 22 C 8 0.274646 -0.929712 0.009512 -0.278439 (3 -1)
XX 3.101496 1.134659 0.001614 H 23 C 7 0.281651 -0.979779 0.008971 -0.291578 (3 -1)
XX 3.313254 -0.000936 0.002762 H 23 H 21 0.017267 0.061731 0.296446 0.001964 (3 -1)
XX 2.561066 1.918039 -0.549735 H 24 C 7 0.274556 -0.929113 0.009524 -0.278267 (3 -1)
XX 2.559816 1.919452 0.549387 H 25 C 7 0.274608 -0.929468 0.009521 -0.278367 (3 -1)
XX -3.100854 1.136362 -0.005444 H 26 C 10 0.281642 -0.979696 0.008991 -0.291562 (3 -1)
XX -3.313242 0.000956 -0.003960 H 26 H 17 0.017263 0.061723 0.296588 0.001965 (3 -1)
XX -2.561403 1.916459 0.551334 H 27 C 10 0.274515 -0.928802 0.009544 -0.278194 (3 -1)
XX -2.556645 1.923092 -0.547716 H 28 C 10 0.274657 -0.929776 0.009513 -0.278455 (3 -1)

M1c Triplet state
XX -1.065437 -0.606583 -0.000020 C 2 C 1 0.302443 -0.804344 0.174860 -0.291590 (3 -1)
XX -0.000000 0.000000 -0.000001 RING 0.019614 0.154041 -1.196773 0.008711 (3 1)
XX 0.000131 -1.206563 -0.000050 C 3 C 2 0.314265 -0.860534 0.219409 -0.315931 (3 -1)
XX 1.065564 -0.606288 -0.000018 C 4 C 3 0.302416 -0.804193 0.174854 -0.291538 (3 -1)
XX 1.065437 0.606584 0.000020 C 5 C 4 0.302443 -0.804343 0.174860 -0.291590 (3 -1)
XX -1.065564 0.606288 0.000016 C 6 C 1 0.302416 -0.804193 0.174854 -0.291538 (3 -1)

XX -0.000131 1.206564 0.000049 C 6 C 5 0.314265 -0.860531 0.219409 -0.315930 (3 -1)
XX -1.032568 -1.801196 -0.000088 H 7 C 2 0.283987 -1.016197 0.018542 -0.295777 (3 -1)
XX 1.033006 -1.800925 -0.000081 H 8 C 3 0.283990 -1.016222 0.018544 -0.295784 (3 -1)
XX 1.032568 1.801196 0.000084 H 9 C 5 0.283988 -1.016203 0.018542 -0.295779 (3 -1)
XX -1.033006 1.800925 0.000084 H 10 C 6 0.283991 -1.016227 0.018544 -0.295785 (3 -1)
XX -2.152173 -0.000140 -0.000001 C 11 C 1 0.287483 -0.741043 0.138705 -0.264411 (3 -1)
XX -3.219467 0.586333 -0.000446 H 12 C 11 0.283039 -1.007234 0.038476 -0.295870 (3 -1)
XX -3.219785 -0.586208 0.000458 H 13 C 11 0.283049 -1.007327 0.038475 -0.295894 (3 -1)
XX 2.152173 0.000140 0.000001 C 14 C 4 0.287483 -0.741042 0.138705 -0.264411 (3 -1)
XX 3.219466 -0.586333 0.000328 H 15 C 14 0.283039 -1.007234 0.038476 -0.295870 (3 -1)
XX 3.219786 0.586207 -0.000330 H 16 C 14 0.283049 -1.007330 0.038475 -0.295895 (3 -1)

M1c Singlet state
XX -1.062279 -0.620687 -0.000013 C 2 C 1 0.278708 -0.710819 0.090077 -0.248297 (3 -1)
XX -0.000000 0.000000 -0.000003 RING 0.018477 0.142866 -1.187822 0.008192 (3 1)
XX 0.000109 -1.235802 -0.000013 C 3 C 2 0.337897 -0.969645 0.316274 -0.367152 (3 -1)
XX 1.062387 -0.620412 -0.000005 C 4 C 3 0.278683 -0.710687 0.090074 -0.248253 (3 -1)
XX 1.062279 0.620687 0.000001 C 5 C 4 0.278708 -0.710819 0.090077 -0.248297 (3 -1)
XX -1.062387 0.620412 0.000004 C 6 C 1 0.278683 -0.710686 0.090074 -0.248253 (3 -1)
XX -0.000109 1.235802 0.000007 C 6 C 5 0.337896 -0.969643 0.316274 -0.367151 (3 -1)
XX -1.020855 -1.828465 0.000003 H 7 C 2 0.283972 -1.018606 0.016489 -0.295807 (3 -1)
XX 1.021253 -1.828210 0.000018 H 8 C 3 0.283975 -1.018629 0.016484 -0.295812 (3 -1)
XX 1.020855 1.828465 -0.000024 H 9 C 5 0.283972 -1.018604 0.016489 -0.295807 (3 -1)
XX -1.021253 1.828210 -0.000013 H 10 C 6 0.283976 -1.018633 0.016484 -0.295813 (3 -1)
XX -2.133330 -0.000123 -0.000001 C 11 C 1 0.333867 -0.948885 0.297317 -0.358211 (3 -1)
XX -3.160598 0.582503 0.000003 H 12 C 11 0.282759 -1.010329 0.022906 -0.294783 (3 -1)
XX -3.160870 -0.582391 0.000029 H 13 C 11 0.282768 -1.010414 0.022900 -0.294804 (3 -1)
XX 2.133330 0.000123 0.000002 C 14 C 4 0.333868 -0.948888 0.297317 -0.358213 (3 -1)
XX 3.160597 -0.582504 0.000018 H 15 C 14 0.282759 -1.010329 0.022906 -0.294783 (3 -1)
XX 3.160870 0.582391 0.000001 H 16 C 14 0.282768 -1.010412 0.022900 -0.294803 (3 -1)

M1d Triplet state

XX -0.598037 -1.070288 0.056847 C 2 C 1 0.294621 -0.753244 0.184822 -0.277507 (3 -1)
XX 0.000012 0.000465 -0.005348 RING 0.019272 0.147710 -1.176725 0.008334 (3 1)
XX -1.223861 -0.001843 -0.003605 C 3 C 2 0.304296 -0.789540 0.230667 -0.295943 (3 -1)
XX -0.601614 1.068957 -0.069836 C 4 C 3 0.294436 -0.752251 0.184867 -0.277194 (3 -1)
XX 0.598093 1.071091 0.056923 C 5 C 4 0.294414 -0.752307 0.184397 -0.277119 (3 -1)
XX 0.601532 -1.068088 -0.070336 C 6 C 1 0.294531 -0.752713 0.184932 -0.277377 (3 -1)
XX 1.223916 0.002471 -0.004116 C 6 C 5 0.304363 -0.789837 0.230895 -0.296075 (3 -1)
XX -1.104985 -2.211950 0.675407 RING 0.013602 0.074825 -1.498799 0.003331 (3 1)
XX -1.809251 -1.046565 0.305804 C 7 C 2 0.260419 -0.655433 0.040656 -0.218963 (3 -1)
XX -2.647431 0.110958 0.134599 RING 0.010763 0.067965 -1.028929 0.003381 (3 1)
XX -1.814309 1.041549 -0.308018 C 8 C 3 0.260618 -0.656456 0.040662 -0.219300 (3 -1)
XX -1.120138 2.210726 -0.696328 RING 0.013413 0.073599 -1.492256 0.003296 (3 1)
XX 1.105434 2.213199 0.676908 RING 0.013567 0.074609 -1.498554 0.003324 (3 1)
XX 1.809597 1.046934 0.305342 C 9 C 5 0.260426 -0.655460 0.040636 -0.218973 (3 -1)
XX 2.647712 -0.119712 0.142228 RING 0.010772 0.067788 -1.041505 0.003364 (3 1)
XX 1.120047 -2.208956 -0.695485 RING 0.013455 0.073876 -1.490694 0.003306 (3 1)
XX 2.652959 0.160879 -0.161552 RING 0.010825 0.066995 -1.101585 0.003286 (3 1)
XX 1.814132 -1.041353 -0.308329 C 10 C 6 0.260524 -0.656013 0.040609 -0.219148 (3 -1)
XX 0.002950 -2.171271 -0.013400 C 10 C 7 0.286276 -0.729354 0.139130 -0.264600 (3 -1)
XX 0.586850 -3.224494 0.072959 H 12 C 11 0.290342 -1.077991 0.026709 -0.310693 (3 -1)
XX -0.578974 -3.224144 -0.114386 H 13 C 11 0.290246 -1.077055 0.026681 -0.310465 (3 -1)
XX -0.002950 2.172098 -0.012669 C 14 C 4 0.286425 -0.729937 0.139816 -0.264871 (3 -1)
XX -0.587249 3.225407 0.070544 H 15 C 14 0.290333 -1.077964 0.026594 -0.310668 (3 -1)
XX 0.579471 3.225097 -0.109467 H 16 C 14 0.290290 -1.077456 0.026564 -0.310555 (3 -1)
XX -2.780034 -1.163109 0.741619 F 17 C 7 0.277142 -0.337084 0.113235 -0.414623 (3 -1)
XX -2.644688 0.048548 0.067650 CAGE 0.010761 0.069248 -0.980070 0.003488 (3 3)
XX -2.678480 -1.671399 0.209061 F 18 C 7 0.272799 -0.372568 0.118125 -0.403889 (3 -1)
XX -2.654176 -0.168544 -0.167177 RING 0.010838 0.066810 -1.116088 0.003267 (3 1)

XX -2.367335 -1.753412 0.878075 F 19 C 7 0.270705 -0.367035 0.116769 -0.399779 (3 -1)
XX -1.200736 -2.701086 0.704075 F 19 C 11 0.015279 0.068244 1.139137 0.002258 (3 -1)
XX 2.794645 -1.157407 -0.723473 F 20 C 10 0.277532 -0.334015 0.112995 -0.415493 (3 -1)
XX 2.675393 -1.675366 -0.204608 F 21 C 10 0.272600 -0.372599 0.118425 -0.403447 (3 -1)
XX 2.644114 -0.032403 0.048158 CAGE 0.010765 0.069482 -0.975667 0.003506 (3 3)
XX 2.379480 -1.740075 -0.882835 F 22 C 10 0.270543 -0.368813 0.117163 -0.399366 (3 -1)
XX 1.218262 -2.689328 -0.732178 F 22 C 11 0.015041 0.067319 1.119584 0.002268 (3 -1)
XX 2.781264 1.163334 0.739645 F 23 C 9 0.277176 -0.336923 0.113131 -0.414695 (3 -1)
XX 3.373334 0.003630 0.019669 F 23 F 20 0.017963 0.078966 0.007545 0.000444 (3 -1)
XX 2.677901 1.672946 0.208745 F 24 C 9 0.272793 -0.372405 0.118151 -0.403880 (3 -1)
XX 2.367926 1.752544 0.878585 F 25 C 9 0.270717 -0.366950 0.116839 -0.399810 (3 -1)
XX 1.204040 2.702994 0.705081 F 25 C 14 0.015236 0.068179 1.146911 0.002274 (3 -1)
XX -2.379858 1.739011 -0.883552 F 26 C 8 0.270546 -0.368746 0.117258 -0.399371 (3 -1)
XX -1.220858 2.691113 -0.731994 F 26 C 14 0.014985 0.067163 1.126529 0.002279 (3 -1)
XX -2.794842 1.156465 -0.723146 F 27 C 8 0.277512 -0.334087 0.113022 -0.415449 (3 -1)
XX -3.371803 -0.004498 0.022616 F 27 F 17 0.017890 0.078664 0.009027 0.000447 (3 -1)
XX -2.675839 1.675315 -0.205202 F 28 C 8 0.272618 -0.372518 0.118410 -0.403488 (3 -1)

M1d Singlet state
XX -0.607032 -1.041347 -0.462105 C 2 C 1 0.263224 -0.629839 0.087193 -0.223508 (3 -1)
XX 0.000785 0.000189 -0.393412 RING 0.020060 0.146685 -1.154744 0.007994 (3 1)
XX -1.259828 -0.021367 -0.228681 C 3 C 2 0.332087 -0.907293 0.359812 -0.355957 (3 -1)
XX -0.620627 1.018467 -0.448226 C 4 C 3 0.263699 -0.633336 0.082042 -0.224154 (3 -1)
XX 0.608401 1.041853 -0.462511 C 5 C 4 0.263294 -0.630180 0.087166 -0.223627 (3 -1)
XX 0.622220 -1.017892 -0.446552 C 6 C 1 0.263507 -0.632470 0.081979 -0.223853 (3 -1)
XX 1.261277 0.022484 -0.227441 C 6 C 5 0.332079 -0.907194 0.359982 -0.355956 (3 -1)
XX -1.124036 -2.312923 -0.561120 RING 0.012966 0.070315 -1.773767 0.003054 (3 1)
XX -1.812627 -1.104422 -0.052796 C 7 C 2 0.259232 -0.651788 0.032389 -0.216846 (3 -1)
XX -1.803407 1.073260 -0.031395 C 8 C 3 0.261590 -0.662956 0.032633 -0.220679 (3 -1)
XX 1.119076 2.317612 -0.553067 RING 0.013037 0.070388 -1.803773 0.003040 (3 1)

XX 1.813331 1.106293 -0.051945 C 9 C 5 0.259197 -0.651630 0.032296 -0.216784 (3 -1)
XX 1.804276 -1.072969 -0.030262 C 10 C 6 0.261740 -0.663691 0.032701 -0.220933 (3 -1)
XX 0.047258 -1.925877 -1.059949 C 11 C 1 0.337523 -0.960080 0.332888 -0.369577 (3 -1)
XX 1.646901 -2.160710 -1.020165 RING 0.008117 0.043846 -1.554543 0.002554 (3 1)
XX -0.484967 -2.768740 -1.689731 H 12 C 11 0.290206 -1.088575 0.008027 -0.310207 (3 -1)
XX 0.689336 -2.716364 -1.642171 H 13 C 11 0.288176 -1.068445 0.006999 -0.305645 (3 -1)
XX -1.677606 2.184018 -1.041203 RING 0.007779 0.041301 -1.672100 0.002404 (3 1)
XX -0.044830 1.925178 -1.062505 C 14 C 4 0.337612 -0.960624 0.333356 -0.369761 (3 -1)
XX -0.685454 2.715157 -1.646980 H 15 C 14 0.288041 -1.067142 0.007065 -0.305344 (3 -1)
XX 0.489022 2.766739 -1.692290 H 16 C 14 0.290102 -1.087631 0.008162 -0.309978 (3 -1)
XX -2.396011 -1.995861 0.056837 F 17 C 7 0.273407 -0.335333 0.110927 -0.406329 (3 -1)
XX -2.493947 -1.551064 0.634552 F 18 C 7 0.275140 -0.372849 0.117283 -0.409464 (3 -1)
XX -2.891241 -0.275991 1.258184 F 18 C 8 0.012688 0.056469 0.184122 0.000618 (3 -1)
XX -2.835196 -1.410883 -0.012862 F 19 C 7 0.274970 -0.360088 0.114457 -0.409389 (3 -1)
XX 2.665527 -1.677235 -0.187877 F 20 C 10 0.268970 -0.382980 0.120659 -0.394969 (3 -1)
XX 1.861153 -2.392131 -1.314633 F 20 H 12 0.008909 0.039821 0.365912 0.001659 (3 -1)
XX 2.602896 0.133232 0.434322 RING 0.009227 0.056672 -1.174336 0.002985 (3 1)
XX 3.012885 0.342461 0.734328 RING 0.010712 0.061816 -1.578706 0.002322 (3 1)
XX 2.704468 -1.358206 0.480323 F 21 C 10 0.280422 -0.318327 0.113336 -0.421647 (3 -1)
XX 2.265591 -1.937089 0.382518 F 22 C 10 0.273505 -0.361584 0.114358 -0.406259 (3 -1)
XX 2.498220 1.548656 0.634554 F 23 C 9 0.275223 -0.372187 0.117244 -0.409668 (3 -1)
XX 2.908282 0.271929 1.247028 F 23 F 21 0.012964 0.057446 0.175112 0.000585 (3 -1)
XX 2.834467 1.418244 -0.017676 F 24 C 9 0.274833 -0.361277 0.114493 -0.409055 (3 -1)
XX 3.399879 0.083067 0.325544 F 24 F 21 0.014335 0.063336 0.096661 0.000550 (3 -1)
XX 2.392409 1.999886 0.063397 F 25 C 9 0.273390 -0.336127 0.110845 -0.406290 (3 -1)
XX -2.673650 1.665783 -0.185866 F 26 C 8 0.269283 -0.382405 0.120498 -0.395704 (3 -1)
XX -1.863249 2.370348 -1.301713 F 26 H 16 0.008238 0.037457 0.500453 0.001685 (3 -1)
XX -2.603799 -0.126979 0.429672 RING 0.009219 0.056723 -1.159147 0.002993 (3 1)
XX -2.996161 -0.340283 0.757042 RING 0.010613 0.061216 -1.582039 0.002313 (3 1)
XX -2.697150 1.358736 0.488916 F 27 C 8 0.280213 -0.319970 0.113712 -0.421198 (3 -1)

XX -3.390189 -0.072565 0.345718 F 27 F 19 0.014350 0.063412 0.087381 0.000552 (3 -1)
XX -2.267354 1.940942 0.372315 F 28 C 8 0.273495 -0.360833 0.114040 -0.406246 (3 -1)

M1e Triplet state
XX 0.598071 1.085966 0.000017 C 2 C 1 0.296290 -0.773377 0.179373 -0.280453 (3 -1)
XX -0.000000 0.000000 0.000000 RING 0.018436 0.140406 -1.196502 0.008077 (3 1)
XX 1.217002 0.000072 0.000119 C 3 C 2 0.301645 -0.791204 0.227524 -0.290449 (3 -1)
XX 0.598308 -1.085897 0.000102 C 4 C 3 0.296293 -0.773388 0.179370 -0.280457 (3 -1)
XX -0.598071 -1.085966 -0.000016 C 5 C 4 0.296290 -0.773377 0.179373 -0.280453 (3 -1)
XX -0.598308 1.085897 -0.000101 C 6 C 1 0.296293 -0.773388 0.179370 -0.280457 (3 -1)
XX -1.217002 -0.000072 -0.000118 C 6 C 5 0.301645 -0.791204 0.227524 -0.290449 (3 -1)
XX 1.800430 1.035434 0.000048 C 7 C 2 0.284698 -0.769721 0.074202 -0.272348 (3 -1)
XX 1.800656 -1.035216 0.000068 C 8 C 3 0.284707 -0.769771 0.074199 -0.272364 (3 -1)
XX -1.800430 -1.035434 -0.000048 C 9 C 5 0.284698 -0.769721 0.074202 -0.272348 (3 -1)
XX -0.000245 2.187592 -0.000073 C 10 C 1 0.293316 -0.767565 0.144901 -0.277084 (3 -1)
XX -1.800656 1.035216 -0.000067 C 10 C 9 0.284707 -0.769771 0.074199 -0.272364 (3 -1)
XX 0.597767 3.230067 0.000084 H 11 C 10 0.288294 -1.072558 0.024305 -0.307524 (3 -1)
XX -0.598686 3.229763 -0.000161 H 12 C 10 0.288294 -1.072543 0.024305 -0.307522 (3 -1)
XX 0.000245 -2.187592 0.000074 C 13 C 4 0.293316 -0.767567 0.144901 -0.277085 (3 -1)
XX 0.598686 -3.229763 0.000163 H 14 C 13 0.288294 -1.072543 0.024305 -0.307522 (3 -1)
XX -0.597767 -3.230067 -0.000082 H 15 C 13 0.288294 -1.072554 0.024304 -0.307523 (3 -1)
XX 2.800353 1.613457 0.000012 N 17 C 7 0.463531 0.277756 0.034579 -0.827481 (3 -1)
XX 2.800672 -1.613055 -0.000055 N 18 C 8 0.463531 0.277755 0.034580 -0.827480 (3 -1)
XX -2.800672 1.613055 0.000054 N 19 C 16 0.463531 0.277754 0.034580 -0.827481 (3 -1)
XX -2.800353 -1.613458 -0.000011 N 20 C 9 0.463531 0.277752 0.034579 -0.827481 (3 -1)

M1e Singlet state
XX 0.607918 -1.082586 -0.000107 C 2 C 1 0.273104 -0.681567 0.109960 -0.239919 (3 -1)
XX -0.000000 0.000000 0.000008 RING 0.017473 0.130997 -1.189600 0.007634 (3 1)
XX 1.247220 -0.000117 -0.000377 C 3 C 2 0.326425 -0.901438 0.321961 -0.341191 (3 -1)

XX 0.608078 1.082474 -0.000257 C 4 C 3 0.273075 -0.681414 0.109960 -0.239870 (3 -1)
XX -0.607919 1.082586 0.000140 C 5 C 4 0.273104 -0.681567 0.109960 -0.239919 (3 -1)
XX -0.608078 -1.082474 0.000289 C 6 C 1 0.273075 -0.681415 0.109960 -0.239870 (3 -1)
XX -1.247220 0.000117 0.000383 C 6 C 5 0.326425 -0.901438 0.321961 -0.341191 (3 -1)
XX 1.827345 -1.022824 -0.000210 C 7 C 2 0.283732 -0.768215 0.062771 -0.270520 (3 -1)
XX 1.827512 1.022534 -0.000327 C 8 C 3 0.283731 -0.768213 0.062766 -0.270519 (3 -1)
XX -1.827344 1.022824 0.000195 C 9 C 5 0.283732 -0.768216 0.062771 -0.270520 (3 -1)
XX -0.000246 -2.159091 0.000125 C 10 C 1 0.335798 -0.960361 0.288246 -0.364227 (3 -1)
XX 0.593711 -3.176431 -0.000298 H 11 C 10 0.288196 -1.077810 0.006910 -0.306718 (3 -1)
XX -1.827512 -1.022534 0.000311 H 12 C 9 0.283731 -0.768212 0.062766 -0.270519 (3 -1)
XX -0.594866 -3.175903 0.000358 H 12 C 10 0.288208 -1.077872 0.006917 -0.306741 (3 -1)
XX 0.000246 2.159092 -0.000027 C 13 C 4 0.335798 -0.960363 0.288246 -0.364228 (3 -1)
XX 0.594866 3.175904 -0.000193 H 14 C 13 0.288207 -1.077872 0.006917 -0.306741 (3 -1)
XX -0.593711 3.176431 0.000463 H 15 C 13 0.288196 -1.077810 0.006910 -0.306718 (3 -1)
XX 2.818552 -1.617109 -0.000036 N 17 C 7 0.464014 0.289194 0.031206 -0.828261 (3 -1)
XX 2.818774 1.616724 -0.000014 N 18 C 8 0.464016 0.289219 0.031206 -0.828265 (3 -1)
XX -2.818775 -1.616724 -0.000042 N 19 C 16 0.464016 0.289218 0.031206 -0.828265 (3 -1)
XX -2.818551 1.617109 -0.000018 N 20 C 9 0.464015 0.289199 0.031206 -0.828262 (3 -1)

M2a Triplet state
XX -1.101051 -0.575925 -0.031765 C 2 C 1 0.294626 -0.748689 0.251670 -0.278321 (3 -1)
XX -0.003796 0.003575 -0.016170 RING 0.018146 0.140181 -1.170429 0.008124 (3 1)
XX -0.030033 -1.219162 -0.007307 C 3 C 2 0.310443 -0.834865 0.293467 -0.306261 (3 -1)
XX 1.059328 -0.627640 -0.014614 C 4 C 3 0.296838 -0.759647 0.260311 -0.281908 (3 -1)
XX 1.092640 0.585448 -0.014420 C 5 C 4 0.295710 -0.751933 0.256911 -0.280168 (3 -1)
XX -1.067944 0.633656 -0.029230 C 6 C 1 0.295456 -0.755252 0.248742 -0.279298 (3 -1)
XX 0.030126 1.226680 -0.005948 C 6 C 5 0.309796 -0.832510 0.284652 -0.304865 (3 -1)
XX -2.312436 -1.304414 -0.082710 RING 0.012096 0.068351 -1.283011 0.003621 (3 1)
XX -0.993022 -1.678157 0.006650 N 7 C 2 0.292610 -0.918314 0.105327 -0.385809 (3 -1)
XX -0.034096 -2.667932 0.235317 RING 0.016844 0.087354 -6.083706 0.004249 (3 1)

XX	0.907830	-1.723260	-0.005734	N	8	C	3	0.289112	-0.894799	0.100921	-0.374877	(3 -1)
XX	2.279099	-1.403394	0.029026	RING				0.013424	0.071373	-1.478734	0.003481	(3 1)
XX	2.282847	1.269128	-0.184096	RING				0.012826	0.073277	-1.286503	0.003797	(3 1)
XX	0.993791	1.690970	0.016254	N	9	C	5	0.289952	-0.899080	0.105153	-0.373420	(3 -1)
XX	-2.148530	0.065242	-0.073817	C	10	C	1	0.275060	-0.672198	0.193897	-0.244204	(3 -1)
XX	-2.221511	1.373025	0.150708	RING				0.013226	0.078195	-1.262545	0.004083	(3 1)
XX	-0.914967	1.727459	-0.011788	C	10	N	9	0.295294	-0.933257	0.104010	-0.388724	(3 -1)
XX	-3.182249	-0.278319	-0.345134	N	11	C	10	0.285854	-0.852999	0.280752	-0.388372	(3 -1)
XX	-3.170880	0.418241	0.215859	N	12	C	10	0.289247	-0.872305	0.213365	-0.394860	(3 -1)
XX	2.140146	-0.062542	-0.047650	C	13	C	4	0.273725	-0.667854	0.182524	-0.242083	(3 -1)
XX	2.745170	-1.860248	0.397207	N	14	N	8	0.019783	0.061363	0.135881	0.000580	(3 -1)
XX	3.155183	-0.383912	0.268296	N	14	C	13	0.291368	-0.865524	0.265194	-0.410246	(3 -1)
XX	2.842838	1.815733	-0.350480	N	15	N	9	0.024919	0.072119	0.048209	-0.000087	(3 -1)
XX	3.181237	0.249163	-0.354411	N	15	C	13	0.283453	-0.837697	0.274312	-0.381353	(3 -1)
XX	-1.006475	-2.960930	0.415363	H	16	N	7	0.339804	-1.776981	0.047258	-0.501054	(3 -1)
XX	-2.129379	-2.419304	0.187903	H	17	N	7	0.342931	-1.814347	0.044801	-0.509312	(3 -1)
XX	1.046688	-2.958505	-0.536661	H	18	N	8	0.338405	-1.742805	0.050102	-0.494935	(3 -1)
XX	-0.025097	-2.720951	0.256139	H	19	N	7	0.016848	0.082044	4.742419	0.003765	(3 -1)
XX	2.036249	-2.481645	0.075451	H	19	N	8	0.342686	-1.816394	0.044839	-0.509139	(3 -1)
XX	1.100539	2.963265	-0.398205	H	20	N	9	0.338647	-1.744141	0.047967	-0.495643	(3 -1)
XX	2.150765	2.394257	-0.043554	H	21	N	9	0.341742	-1.812279	0.040985	-0.508468	(3 -1)
XX	-2.735171	1.902039	0.442615	N	22	N	12	0.024792	0.070463	0.046717	-0.000142	(3 -1)
XX	-2.028955	2.474309	0.163012	H	23	N	22	0.341040	-1.812412	0.039997	-0.507845	(3 -1)
XX	-0.915723	3.006825	0.385160	H	24	N	22	0.339836	-1.761655	0.046552	-0.499260	(3 -1)
XX	4.388073	0.804414	-0.698992	H	25	N	15	0.341568	-1.773787	0.049377	-0.501618	(3 -1)
XX	3.470015	0.915116	-1.524220	H	26	N	15	0.339624	-1.770983	0.046973	-0.499073	(3 -1)
XX	4.325465	-0.929029	0.771394	H	27	N	14	0.341774	-1.770458	0.055088	-0.501397	(3 -1)
XX	3.403246	-0.752243	1.596232	H	28	N	14	0.334496	-1.729645	0.053534	-0.489244	(3 -1)
XX	-4.326532	1.045499	0.598944	H	29	N	12	0.341177	-1.760444	0.054201	-0.499390	(3 -1)
XX	-3.529288	0.781040	1.505045	H	30	N	12	0.332075	-1.696550	0.053538	-0.482631	(3 -1)

XX -4.368729 -0.773273 -0.811616 H 31 N 11 0.340819 -1.766064 0.046632 -0.499663 (3 -1)
XX -2.847355 -1.842811 -0.133941 H 31 H 16 0.020600 0.069988 0.082992 0.001114 (3 -1)
XX -3.337738 -1.207532 -1.348937 H 32 N 11 0.340237 -1.786507 0.043010 -0.501569 (3 -1)

M2a Singlet state
XX -1.071771 -0.602471 -0.272442 C 2 C 1 0.270870 -0.659929 0.141349 -0.235993 (3 -1)
XX -0.002377 0.009488 -0.400644 RING 0.018929 0.138772 -1.136507 0.007714 (3 1)
XX -0.002674 -1.233211 -0.456046 C 3 C 2 0.334297 -0.931713 0.463902 -0.358522 (3 -1)
XX 1.049178 -0.620275 -0.251933 C 4 C 3 0.265532 -0.628154 0.152246 -0.227138 (3 -1)
XX 1.054756 0.617581 -0.246109 C 5 C 4 0.265558 -0.631293 0.163671 -0.227282 (3 -1)
XX -1.069315 0.617208 -0.255944 C 6 C 1 0.268986 -0.651275 0.141328 -0.233225 (3 -1)
XX 0.006413 1.251806 -0.433090 C 6 C 5 0.331638 -0.909377 0.476424 -0.353889 (3 -1)
XX -0.978399 -1.654242 -0.626469 N 7 C 2 0.286466 -0.884478 0.100303 -0.373279 (3 -1)
XX -0.024658 -2.469743 -1.034382 RING 0.017316 0.091123 -2.026428 0.004308 (3 1)
XX 0.931771 -1.692498 -0.588618 N 8 C 3 0.273601 -0.798313 0.095134 -0.346226 (3 -1)
XX 2.112128 1.336409 0.099772 RING 0.013315 0.080490 -1.229672 0.004239 (3 1)
XX 0.964057 1.695582 -0.536616 N 9 C 5 0.272082 -0.792079 0.082986 -0.344700 (3 -1)
XX -0.000764 2.690684 -0.914014 RING 0.013111 0.061805 -2.846681 0.003081 (3 1)
XX -0.979215 1.674520 -0.575942 N 10 C 6 0.281646 -0.856696 0.095812 -0.367255 (3 -1)
XX -1.973024 -0.000040 0.231303 C 11 C 1 0.318245 -0.857952 0.420712 -0.337268 (3 -1)
XX -2.042757 -1.413462 0.169235 RING 0.012496 0.071562 -1.300320 0.003831 (3 1)
XX -2.096016 1.400401 0.170995 RING 0.011925 0.068251 -1.269006 0.003664 (3 1)
XX -2.255492 -2.150938 0.156170 N 12 N 7 0.021940 0.065458 0.061395 0.000318 (3 -1)
XX -2.831878 -0.459951 0.786906 N 12 C 11 0.308919 -1.039844 0.135239 -0.428427 (3 -1)
XX -2.338698 2.140449 0.174741 N 13 N 10 0.022067 0.067862 0.038844 0.000481 (3 -1)
XX -2.887899 0.427469 0.740005 N 13 C 11 0.306701 -1.029786 0.141868 -0.417499 (3 -1)
XX 2.051520 -1.476403 0.209687 RING 0.011229 0.057332 -1.338085 0.002830 (3 1)
XX 1.971966 -0.002031 0.216722 C 14 C 4 0.317677 -0.855455 0.418312 -0.336044 (3 -1)
XX 2.228623 -2.098407 0.175760 N 15 N 8 0.014853 0.051061 0.157676 0.001341 (3 -1)
XX 2.886604 -0.446962 0.711400 N 15 C 14 0.299657 -0.987786 0.134731 -0.403835 (3 -1)

XX 2.381907 2.125924 0.099168 N 16 N 9 0.030529 0.081880 0.032846 -0.000817 (3 -1)
XX 2.883799 0.445333 0.720091 N 16 C 14 0.309299 -1.039247 0.142696 -0.421032 (3 -1)
XX -1.057218 2.918795 -1.124995 H 17 N 10 0.341086 -1.758016 0.046048 -0.499124 (3 -1)
XX -2.034400 2.189179 -1.246282 H 18 N 10 0.338635 -1.754322 0.047249 -0.496464 (3 -1)
XX 1.744596 2.412932 -1.413806 H 19 C 5 0.336434 -1.725888 0.053319 -0.490481 (3 -1)
XX 0.010416 2.865747 -0.941266 H 19 H 17 0.013201 0.055603 1.608104 0.002520 (3 -1)
XX 1.075679 3.068859 -0.612105 H 20 N 9 0.342399 -1.765107 0.054629 -0.501475 (3 -1)
XX 2.030772 -2.298590 -1.144939 H 21 N 8 0.342561 -1.786542 0.051558 -0.504142 (3 -1)
XX 1.344236 -2.997519 -0.393650 H 22 N 8 0.338512 -1.722606 0.058184 -0.492443 (3 -1)
XX -1.990505 -2.174048 -1.339837 H 23 N 7 0.338243 -1.739070 0.045665 -0.493819 (3 -1)
XX -0.966297 -2.861823 -1.210494 H 24 N 7 0.338031 -1.772531 0.041439 -0.498446 (3 -1)
XX 0.026909 -2.703303 -1.154588 H 24 N 8 0.017883 0.066152 0.672798 0.001851 (3 -1)
XX 4.030182 1.106295 0.921478 H 25 N 16 0.339191 -1.749429 0.048064 -0.496666 (3 -1)
XX 2.988616 1.794343 0.665402 H 26 N 16 0.336589 -1.798220 0.039026 -0.502252 (3 -1)
XX 3.681618 -1.069944 1.589812 H 27 N 15 0.340500 -1.773430 0.048342 -0.500893 (3 -1)
XX 2.907676 -1.786707 0.931476 H 28 N 15 0.345108 -1.813852 0.046176 -0.510905 (3 -1)
XX -3.634961 1.090202 1.634375 H 29 C 11 0.340263 -1.771411 0.048816 -0.500388 (3 -1)
XX -2.986879 1.788486 0.792056 H 30 N 13 0.339587 -1.811090 0.043060 -0.505628 (3 -1)
XX -3.930288 -1.196040 1.068060 H 31 N 12 0.340032 -1.765328 0.050313 -0.499461 (3 -1)
XX -2.846320 -1.829119 0.852815 H 32 N 12 0.339284 -1.814760 0.042634 -0.505689 (3 -1)

M2b Triplet state
XX -1.038048 0.681771 -0.040903 C 2 C 1 0.298837 -0.770267 0.216144 -0.285108 (3 -1)
XX 0.005756 0.030970 -0.006750 RING 0.018774 0.144984 -1.172917 0.008295 (3 1)
XX 0.044775 1.260044 -0.003355 C 3 C 2 0.309111 -0.818642 0.230298 -0.304482 (3 -1)
XX 1.087318 0.614045 0.013416 C 4 C 3 0.291630 -0.736378 0.199024 -0.271916 (3 -1)
XX 1.043184 -0.627474 -0.017217 C 5 C 4 0.292436 -0.739661 0.202824 -0.273409 (3 -1)
XX -1.085388 -0.541592 -0.005042 C 6 C 1 0.298203 -0.762954 0.218885 -0.284489 (3 -1)
XX -0.044816 -1.196498 -0.000813 C 6 C 5 0.307248 -0.808658 0.227598 -0.300827 (3 -1)
XX -2.267505 1.431438 -0.247038 RING 0.015752 0.074739 -2.570577 0.003719 (3 1)

XX	-1.012757	1.957934	-0.067475	C	7	C	2	0.250820	-0.584951	0.031768	-0.204120	(3 -1)
XX	1.133900	1.888846	0.128703	C	8	C	3	0.249899	-0.578945	0.037552	-0.202784	(3 -1)
XX	2.518595	1.364901	0.147540	RING				0.012037	0.057093	-1.730002	0.002911	(3 1)
XX	2.405485	-1.469315	-0.140920	RING				0.011975	0.057448	-1.658428	0.002948	(3 1)
XX	0.998588	-1.904375	-0.103166	C	9	C	5	0.249741	-0.577818	0.037758	-0.202568	(3 -1)
XX	-2.653409	-1.294190	-0.004142	RING				0.012137	0.053732	-3.386571	0.002624	(3 1)
XX	-1.145513	-1.818678	0.082692	C	10	C	6	0.249197	-0.577600	0.033110	-0.201695	(3 -1)
XX	-2.139687	0.117458	-0.056999	C	11	C	1	0.267728	-0.649835	0.103670	-0.230519	(3 -1)
XX	-2.472735	1.497397	-0.289175	N	12	C	7	0.015976	0.060687	1.187864	0.002433	(3 -1)
XX	-2.883630	-1.541383	-0.205428	N	12	C	10	0.012564	0.047664	0.665764	0.001960	(3 -1)
XX	-3.166445	-0.101025	-0.458664	N	12	C	11	0.282253	-0.829044	0.282859	-0.376119	(3 -1)
XX	-3.154877	0.270692	0.355399	N	13	C	11	0.291404	-0.868160	0.260186	-0.408649	(3 -1)
XX	2.126167	-0.043740	-0.007742	C	14	C	4	0.281445	-0.701594	0.214930	-0.255785	(3 -1)
XX	2.777675	1.672104	-0.050897	N	15	C	8	0.012773	0.049805	0.164561	0.002155	(3 -1)
XX	3.156843	0.223140	-0.322165	N	15	C	14	0.295255	-0.869198	0.276488	-0.427053	(3 -1)
XX	2.672015	-1.808744	0.060524	N	16	C	9	0.012885	0.049817	0.129674	0.002132	(3 -1)
XX	3.142269	-0.374455	0.298029	N	16	C	14	0.295305	-0.871944	0.273490	-0.426400	(3 -1)
XX	0.933736	-3.000390	-0.533078	H	17	C	9	0.277797	-0.949187	0.008318	-0.283862	(3 -1)
XX	1.924275	-2.510107	-0.517431	H	18	C	9	0.279598	-0.969410	0.007257	-0.287621	(3 -1)
XX	1.472143	-2.867629	0.414480	H	19	C	9	0.272061	-0.909002	0.009708	-0.273662	(3 -1)
XX	1.674264	2.832985	-0.357536	H	20	C	8	0.272174	-0.910017	0.009542	-0.273871	(3 -1)
XX	1.143296	2.971554	0.593841	H	21	C	8	0.277825	-0.949943	0.008292	-0.283914	(3 -1)
XX	2.097946	2.415219	0.562658	H	22	C	8	0.279463	-0.967880	0.007316	-0.287322	(3 -1)
XX	-1.045118	3.049144	-0.516627	H	23	C	7	0.275631	-0.933497	0.009483	-0.279974	(3 -1)
XX	-2.010003	2.535966	-0.313034	H	24	C	7	0.279824	-0.975235	0.007156	-0.288194	(3 -1)
XX	-1.391536	2.916212	0.516872	H	24	H	21	0.272411	-0.912464	0.009480	-0.274493	(3 -1)
XX	-1.240278	-2.874514	0.598653	H	26	C	10	0.275905	-0.935674	0.008692	-0.280453	(3 -1)
XX	-2.178234	-2.339104	0.346982	H	27	C	10	0.281824	-0.989436	0.007426	-0.291909	(3 -1)
XX	-1.569750	-2.796132	-0.443701	H	28	C	10	0.272057	-0.908376	0.009432	-0.273678	(3 -1)
XX	-4.324755	0.468772	1.056087	H	29	N	13	0.341463	-1.761266	0.054498	-0.499922	(3 -1)

XX -3.354216 0.081583 1.720388 H 30 N 13 0.333505 -1.705711 0.054602 -0.485394 (3 -1)
XX -4.319719 -0.376005 -1.135820 H 31 N 12 0.339842 -1.749771 0.047042 -0.496813 (3 -1)
XX -3.265278 -0.543784 -1.745006 H 32 N 12 0.341130 -1.778297 0.044919 -0.501637 (3 -1)
XX 4.367905 0.796239 -0.728586 H 33 N 15 0.342452 -1.781926 0.058210 -0.503862 (3 -1)
XX 3.446669 0.756074 -1.590111 H 34 N 15 0.340374 -1.777494 0.056461 -0.501072 (3 -1)
XX 4.320033 -1.014821 0.695415 H 35 N 16 0.342416 -1.781023 0.058087 -0.503693 (3 -1)
XX 3.416878 -0.906795 1.568379 H 36 N 16 0.340126 -1.774982 0.056441 -0.500524 (3 -1)

M2b Singlet state
XX 1.036218 0.632392 -0.281534 C   2 C   1 0.262668 -0.617517 0.105756 -0.222499 (3 -1)
XX 0.000010 0.000282 -0.387861 RING   0.020012 0.146120 -1.126198 0.007951 (3 1)
XX -0.000615 1.246907 -0.501685 C   3 C   2 0.331600 -0.908929 0.372024 -0.353196 (3 -1)
XX -1.034808 0.622034 -0.278627 C   4 C   3 0.262881 -0.617218 0.110466 -0.222862 (3 -1)
XX -1.036191 -0.631939 -0.281638 C   5 C   4 0.262690 -0.617621 0.105646 -0.222534 (3 -1)
XX 1.034769 -0.621601 -0.278627 C   6 C   1 0.262889 -0.617242 0.110460 -0.222875 (3 -1)
XX 0.000572 -1.246402 -0.501879 C   6 C   5 0.331646 -0.909225 0.371927 -0.353289 (3 -1)
XX 1.073326 1.838058 -0.760078 C   7 C   2 0.247610 -0.567157 0.043251 -0.199643 (3 -1)
XX -0.047516 2.555925 -1.480893 RING   0.011141 0.054735 -2.998604 0.003301 (3 1)
XX -1.084557 1.824187 -0.763326 C   8 C   3 0.246190 -0.560989 0.043793 -0.197609 (3 -1)
XX -1.073306 -1.837247 -0.761157 C   9 C   5 0.247585 -0.567042 0.043280 -0.199608 (3 -1)
XX 0.047662 -2.556064 -1.486130 RING   0.011122 0.054525 -3.073300 0.003290 (3 1)
XX 1.084379 -1.823613 -0.763817 C 10 C   6 0.246187 -0.560974 0.043837 -0.197606 (3 -1)
XX 1.898725 -0.003637 0.281879 C 11 C   1 0.322060 -0.878072 0.432510 -0.347445 (3 -1)
XX 1.928427 -1.721647 0.282758 RING   0.010958 0.049959 -2.314369 0.002578 (3 1)
XX 2.688295 -0.457281 0.935956 N 12 C 11 0.302048 -0.995621 0.135746 -0.418402 (3 -1)
XX 2.096330 1.531724 0.133320 RING   0.009598 0.049487 -1.423535 0.002755 (3 1)
XX 2.794525 0.406291 0.838727 N 13 C 11 0.302314 -1.000796 0.144809 -0.415562 (3 -1)
XX -1.928597 1.717751 0.283405 RING   0.010945 0.049978 -2.282312 0.002581 (3 1)
XX -1.898634 0.003706 0.281912 C 14 C   4 0.322097 -0.878268 0.432655 -0.347535 (3 -1)
XX -2.688892 0.456693 0.935817 N 15 C 14 0.302038 -0.995598 0.135734 -0.418337 (3 -1)

XX -2.094141 -1.532706 0.134568 RING 0.009614 0.049553 -1.426714 0.002758 (3 1)
XX -2.793765 -0.407301 0.839169 N 16 C 14 0.302251 -1.000461 0.144755 -0.415223 (3 -1)
XX -1.149610 -2.708586 -1.576155 H 17 C 9 0.275138 -0.933633 0.008135 -0.279217 (3 -1)
XX -1.538572 -2.929541 -0.582168 H 18 C 9 0.269693 -0.886540 0.013648 -0.269334 (3 -1)
XX -2.089215 -2.238360 -1.241968 H 19 C 9 0.277855 -0.953848 0.009857 -0.283802 (3 -1)
XX -1.439753 2.969854 -0.667232 H 20 C 8 0.271418 -0.900448 0.012175 -0.272215 (3 -1)
XX -2.143721 1.988566 0.194751 H 20 N 15 0.011270 0.047724 0.450794 0.002272 (3 -1)
XX -0.063384 2.686923 -1.685116 H 21 C 3 0.011275 0.048682 1.990953 0.002881 (3 -1)
XX -1.260581 2.576771 -1.669812 H 21 C 8 0.273531 -0.920777 0.007651 -0.276351 (3 -1)
XX -2.146135 2.243823 -1.106487 H 22 C 8 0.278978 -0.962948 0.009031 -0.285996 (3 -1)
XX 1.148927 2.711717 -1.572699 H 23 C 7 0.275173 -0.933898 0.008134 -0.279285 (3 -1)
XX 1.540184 2.929258 -0.578892 H 24 C 7 0.269700 -0.886581 0.013647 -0.269351 (3 -1)
XX 2.088637 2.239408 -1.241887 H 25 C 7 0.277816 -0.953521 0.009867 -0.283727 (3 -1)
XX 0.063129 -2.682677 -1.685161 H 26 C 6 0.011245 0.048638 2.068658 0.002883 (3 -1)
XX 1.261172 -2.574441 -1.671563 H 26 C 10 0.273507 -0.920600 0.007647 -0.276305 (3 -1)
XX 2.146095 -2.243946 -1.105831 H 27 C 10 0.278988 -0.963021 0.009027 -0.286017 (3 -1)
XX 2.141671 -1.986060 0.194763 H 27 N 12 0.011265 0.047724 0.474238 0.002276 (3 -1)
XX 1.437853 -2.969916 -0.669445 H 28 C 10 0.271437 -0.900612 0.012159 -0.272250 (3 -1)
XX 3.483356 1.094396 1.774328 H 29 N 13 0.340232 -1.776074 0.050367 -0.500990 (3 -1)
XX 2.393466 2.133993 0.013012 H 29 H 23 0.012903 0.052695 0.507575 0.002086 (3 -1)
XX 3.034057 1.757489 0.811175 H 30 N 13 0.346374 -1.827766 0.047501 -0.513572 (3 -1)
XX 3.725938 -1.283674 1.263215 H 31 N 12 0.339226 -1.763669 0.052223 -0.498375 (3 -1)
XX 2.576229 -1.795018 1.227500 H 32 N 12 0.346040 -1.828192 0.048804 -0.512857 (3 -1)
XX -3.727997 1.281121 1.262799 H 33 N 15 0.339228 -1.763668 0.052216 -0.498379 (3 -1)
XX -2.579544 1.794953 1.226033 H 34 N 15 0.346044 -1.828187 0.048795 -0.512863 (3 -1)
XX -3.481427 -1.095428 1.775227 H 35 N 16 0.340221 -1.775924 0.050336 -0.500959 (3 -1)
XX -2.391135 -2.133154 0.013016 H 35 H 17 0.012892 0.052696 0.501840 0.002088 (3 -1)
XX -3.029974 -1.758892 0.813616 H 36 N 16 0.346346 -1.827513 0.047469 -0.513510 (3 -1)

M2c Triplet state

XX 1.036218 0.632392 -0.281534 C 2 C 1 0.262668 -0.617517 0.105756 -0.222499 (3 -1)
XX 0.000010 0.000282 -0.387861 RING 0.020012 0.146120 -1.126198 0.007951 (3 1)
XX -0.000615 1.246907 -0.501685 C 3 C 2 0.331600 -0.908929 0.372024 -0.353196 (3 -1)
XX -1.034808 0.622034 -0.278627 C 4 C 3 0.262881 -0.617218 0.110466 -0.222862 (3 -1)
XX -1.036191 -0.631939 -0.281638 C 5 C 4 0.262690 -0.617621 0.105646 -0.222534 (3 -1)
XX 1.034769 -0.621601 -0.278627 C 6 C 1 0.262889 -0.617242 0.110460 -0.222875 (3 -1)
XX 0.000572 -1.246402 -0.501879 C 6 C 5 0.331646 -0.909225 0.371927 -0.353289 (3 -1)
XX 1.073326 1.838058 -0.760078 C 7 C 2 0.247610 -0.567157 0.043251 -0.199643 (3 -1)
XX -0.047516 2.555925 -1.480893 RING 0.011141 0.054735 -2.998604 0.003301 (3 1)
XX -1.084557 1.824187 -0.763326 C 8 C 3 0.246190 -0.560989 0.043793 -0.197609 (3 -1)
XX -1.073306 -1.837247 -0.761157 C 9 C 5 0.247585 -0.567042 0.043280 -0.199608 (3 -1)
XX 0.047662 -2.556064 -1.486130 RING 0.011122 0.054525 -3.073300 0.003290 (3 1)
XX 1.084379 -1.823613 -0.763817 C 10 C 6 0.246187 -0.560974 0.043837 -0.197606 (3 -1)
XX 1.898725 -0.003637 0.281879 C 11 C 1 0.322060 -0.878072 0.432510 -0.347445 (3 -1)
XX 1.928427 -1.721647 0.282758 RING 0.010958 0.049959 -2.314369 0.002578 (3 1)
XX 2.688295 -0.457281 0.935956 N 12 C 11 0.302048 -0.995621 0.135746 -0.418402 (3 -1)
XX 2.096330 1.531724 0.133320 RING 0.009598 0.049487 -1.423535 0.002755 (3 1)
XX 2.794525 0.406291 0.838727 N 13 C 11 0.302314 -1.000796 0.144809 -0.415562 (3 -1)
XX -1.928597 1.717751 0.283405 RING 0.010945 0.049978 -2.282312 0.002581 (3 1)
XX -1.898634 0.003706 0.281912 C 14 C 4 0.322097 -0.878268 0.432655 -0.347535 (3 -1)
XX -2.688892 0.456693 0.935817 N 15 C 14 0.302038 -0.995598 0.135734 -0.418337 (3 -1)
XX -2.094141 -1.532706 0.134568 RING 0.009614 0.049553 -1.426714 0.002758 (3 1)
XX -2.793765 -0.407301 0.839169 N 16 C 14 0.302251 -1.000461 0.144755 -0.415223 (3 -1)
XX -1.149610 -2.708586 -1.576155 H 17 C 9 0.275138 -0.933633 0.008135 -0.279217 (3 -1)
XX -1.538572 -2.929541 -0.582168 H 18 C 9 0.269693 -0.886540 0.013648 -0.269334 (3 -1)
XX -2.089215 -2.238360 -1.241968 H 19 C 9 0.277855 -0.953848 0.009857 -0.283802 (3 -1)
XX -1.439753 2.969854 -0.667232 H 20 C 8 0.271418 -0.900448 0.012175 -0.272215 (3 -1)
XX -2.143721 1.988566 0.194751 H 20 N 15 0.011270 0.047724 0.450794 0.002272 (3 -1)
XX -0.063384 2.686923 -1.685116 H 21 C 3 0.011275 0.048682 1.990953 0.002881 (3 -1)
XX -1.260581 2.576771 -1.669812 H 21 C 8 0.273531 -0.920777 0.007651 -0.276351 (3 -1)

XX -2.146135 2.243823 -1.106487 H 22 C 8 0.278978 -0.962948 0.009031 -0.285996 (3 -1)
XX 1.148927 2.711717 -1.572699 H 23 C 7 0.275173 -0.933898 0.008134 -0.279285 (3 -1)
XX 1.540184 2.929258 -0.578892 H 24 C 7 0.269700 -0.886581 0.013647 -0.269351 (3 -1)
XX 2.088637 2.239408 -1.241887 H 25 C 7 0.277816 -0.953521 0.009867 -0.283727 (3 -1)
XX 0.063129 -2.682677 -1.685161 H 26 C 6 0.011245 0.048638 2.068658 0.002883 (3 -1)
XX 1.261172 -2.574441 -1.671563 H 26 C 10 0.273507 -0.920600 0.007647 -0.276305 (3 -1)
XX 2.146095 -2.243946 -1.105831 H 27 C 10 0.278988 -0.963021 0.009027 -0.286017 (3 -1)
XX 2.141671 -1.986060 0.194763 H 27 N 12 0.011265 0.047724 0.474238 0.002276 (3 -1)
XX 1.437853 -2.969916 -0.669445 H 28 C 10 0.271437 -0.900612 0.012159 -0.272250 (3 -1)
XX 3.483356 1.094396 1.774328 H 29 N 13 0.340232 -1.776074 0.050367 -0.500990 (3 -1)
XX 2.393466 2.133993 0.013012 H 29 H 23 0.012903 0.052695 0.507575 0.002086 (3 -1)
XX 3.034057 1.757489 0.811175 H 30 N 13 0.346374 -1.827766 0.047501 -0.513572 (3 -1)
XX 3.725938 -1.283674 1.263215 H 31 N 12 0.339226 -1.763669 0.052223 -0.498375 (3 -1)
XX 2.576229 -1.795018 1.227500 H 32 N 12 0.346040 -1.828192 0.048804 -0.512857 (3 -1)
XX -3.727997 1.281121 1.262799 H 33 N 15 0.339228 -1.763668 0.052216 -0.498379 (3 -1)
XX -2.579544 1.794953 1.226033 H 34 N 15 0.346044 -1.828187 0.048795 -0.512863 (3 -1)
XX -3.481427 -1.095428 1.775227 H 35 N 16 0.340221 -1.775924 0.050336 -0.500959 (3 -1)
XX -2.391135 -2.133154 0.013016 H 35 H 17 0.012892 0.052696 0.501840 0.002088 (3 -1)
XX -3.029974 -1.758892 0.813616 H 36 N 16 0.346346 -1.827513 0.047469 -0.513510 (3 -1)

M2c Singlet state
XX -1.080143 0.612760 -0.003782 C 2 C 1 0.277474 -0.690761 0.131151 -0.247240 (3 -1)
XX 0.000001 -0.000008 -0.001175 RING 0.018651 0.142373 -1.206734 0.008124 (3 1)
XX -0.000151 1.217987 -0.001231 C 3 C 2 0.332304 -0.934225 0.328750 -0.355801 (3 -1)
XX 1.079988 0.612868 0.001965 C 4 C 3 0.277397 -0.690374 0.131115 -0.247108 (3 -1)
XX 1.080146 -0.612776 -0.003724 C 5 C 4 0.277474 -0.690764 0.131149 -0.247240 (3 -1)
XX -1.079986 -0.612882 0.001985 C 6 C 1 0.277396 -0.690366 0.131116 -0.247105 (3 -1)
XX 0.000157 -1.218002 -0.001150 C 6 C 5 0.332304 -0.934222 0.328750 -0.355800 (3 -1)
XX -2.170130 1.529904 -0.147100 RING 0.011253 0.057238 -2.108822 0.003389 (3 1)
XX -0.995906 1.819503 0.001119 H 7 C 2 0.281680 -0.984728 0.023425 -0.291899 (3 -1)

XX 0.995456 1.819649 -0.004480 H   8 C   3 0.281681 -0.984726 0.023439 -0.291907 (3 -1)
XX 2.169754 1.530114 0.145914 RING   0.011242 0.057207 -2.106667 0.003388 (3 1)
XX 2.170154 -1.530047 -0.147119 RING   0.011251 0.057224 -2.110012 0.003388 (3 1)
XX 0.995918 -1.819516 0.001295 H   9 C   5 0.281682 -0.984745 0.023425 -0.291902 (3 -1)
XX -2.169764 -1.530079 0.145883 RING   0.011241 0.057209 -2.106181 0.003388 (3 1)
XX -0.995440 -1.819667 -0.004357 H 10 C   6 0.281682 -0.984726 0.023439 -0.291908 (3 -1)
XX -2.114775 -0.000139 -0.000201 C 11 C   1 0.319197 -0.865713 0.386462 -0.335124 (3 -1)
XX -2.179228 -1.836053 0.184497 N 12 H 10 0.011643 0.049485 0.851273 0.002595 (3 -1)
XX -3.139235 -0.425414 0.085757 N 12 C 11 0.303657 -0.996110 0.155652 -0.428259 (3 -1)
XX -2.179747 1.835155 -0.186414 N 13 H   7 0.011652 0.049525 0.853874 0.002598 (3 -1)
XX -3.139324 0.425126 -0.084597 N 13 C 11 0.303674 -0.996253 0.155343 -0.428317 (3 -1)
XX 2.114778 0.000128 -0.000176 C 14 C   4 0.319197 -0.865716 0.386457 -0.335124 (3 -1)
XX 2.179217 1.835950 0.184525 N 15 H   8 0.011643 0.049486 0.851733 0.002596 (3 -1)
XX 3.139223 0.425405 0.085796 N 15 C 14 0.303658 -0.996108 0.155655 -0.428271 (3 -1)
XX 2.179770 -1.834999 -0.186402 N 16 H   9 0.011649 0.049518 0.855030 0.002598 (3 -1)
XX 3.139327 -0.425112 -0.084605 N 16 C 14 0.303675 -0.996242 0.155348 -0.428325 (3 -1)
XX 4.194631 -1.232119 0.248116 H 17 N 16 0.338904 -1.767934 0.051349 -0.498615 (3 -1)
XX 3.260156 -1.758256 -0.411764 H 18 N 16 0.345318 -1.818187 0.049786 -0.510909 (3 -1)
XX 4.196877 1.230750 -0.243197 H 19 N 15 0.338910 -1.768090 0.051308 -0.498635 (3 -1)
XX 3.259399 1.759125 0.410537 H 20 N 15 0.345329 -1.818267 0.049749 -0.510935 (3 -1)
XX -4.196955 -1.230643 -0.243212 H 21 N 12 0.338911 -1.768097 0.051305 -0.498637 (3 -1)
XX -3.259430 -1.759169 0.410307 H 22 N 12 0.345330 -1.818271 0.049746 -0.510937 (3 -1)
XX -4.194616 1.232085 0.248204 H 23 N 13 0.338902 -1.767919 0.051347 -0.498611 (3 -1)
XX -3.260113 1.758311 -0.411533 H 24 N 13 0.345317 -1.818178 0.049783 -0.510908 (3 -1)

M2d Triplet state
XX 0.605538 -1.080886 -0.086356 C   2 C   1 0.285904 -0.701721 0.228355 -0.262802 (3 -1)
XX -0.006444 0.000657 -0.001746 RING   0.018630 0.133569 -1.170593 0.007590 (3 1)
XX 1.222592 0.003563 -0.002209 C   3 C   2 0.290844 -0.717541 0.255541 -0.271312 (3 -1)
XX 0.599650 1.084928 0.080192 C   4 C   3 0.285609 -0.700424 0.227422 -0.262247 (3 -1)

XX -0.638225 1.079510 -0.058786 C 5 C 4 0.285199 -0.693180 0.238059 -0.262483 (3 -1)
XX -0.632256 -1.081184 0.053637 C 6 C 1 0.285281 -0.693695 0.237474 -0.262629 (3 -1)
XX -1.242119 -0.002537 -0.001276 C 6 C 5 0.273245 -0.644497 0.163254 -0.240402 (3 -1)
XX 1.793691 -1.001665 -0.360111 C 7 C 2 0.263021 -0.660563 0.088364 -0.229736 (3 -1)
XX 2.688379 0.008053 0.001173 CAGE 0.010430 0.066440 -0.829454 0.003328 (3 3)
XX 1.787062 1.010851 0.359264 C 8 C 3 0.262992 -0.660276 0.088702 -0.229676 (3 -1)
XX -1.771335 0.992488 -0.444056 C 9 C 5 0.270363 -0.685795 0.144137 -0.245881 (3 -1)
XX -1.763144 -1.000775 0.445173 C 10 C 6 0.270260 -0.685137 0.144307 -0.245691 (3 -1)
XX -0.016410 -2.119283 -0.071803 C 11 C 1 0.287780 -0.743060 0.200429 -0.271468 (3 -1)
XX -1.768478 -2.220402 -0.249463 RING 0.009750 0.046230 -1.285911 0.002302 (3 1)
XX -0.404598 -3.117346 -0.319759 N 12 C 11 0.330491 -1.057211 0.161893 -0.523971 (3 -1)
XX 0.299990 -3.165223 0.091937 N 13 C 11 0.329713 -1.044537 0.169635 -0.523183 (3 -1)
XX -0.025762 2.121039 0.060802 C 14 C 4 0.288004 -0.743869 0.201426 -0.271791 (3 -1)
XX -1.771003 2.217203 0.240535 RING 0.009715 0.046129 -1.283596 0.002304 (3 1)
XX 0.288751 3.166161 -0.109220 N 15 C 14 0.329402 -1.043837 0.170185 -0.522255 (3 -1)
XX -0.415730 3.119760 0.303374 N 16 C 14 0.330308 -1.056937 0.162728 -0.523352 (3 -1)
XX 2.774812 0.339634 -0.236465 RING 0.010815 0.061918 -1.555398 0.002739 (3 1)
XX 2.761143 -1.055038 -0.856293 F 17 C 7 0.274630 -0.327478 0.118622 -0.409373 (3 -1)
XX 1.071203 -2.291654 -0.845793 RING 0.012259 0.060420 -2.582232 0.002430 (3 1)
XX 2.727945 -1.596031 -0.342035 F 18 C 7 0.256340 -0.413660 0.123793 -0.362439 (3 -1)
XX 2.991908 -0.590205 0.412713 RING 0.010495 0.058592 -2.024193 0.002307 (3 1)
XX 2.231714 -2.772070 0.603695 F 18 N 13 0.018328 0.068132 0.142506 0.000217 (3 -1)
XX 2.775756 -0.328633 0.243198 RING 0.010832 0.061979 -1.565353 0.002738 (3 1)
XX 2.373912 -1.664203 -1.000641 F 19 C 7 0.265879 -0.367025 0.122403 -0.388763 (3 -1)
XX 1.140844 -2.575960 -0.828982 F 19 C 11 0.012436 0.055139 3.700513 0.001710 (3 -1)
XX -2.837703 0.667891 0.562772 RING 0.008141 0.042084 -1.819905 0.001742 (3 1)
XX -2.653714 -1.018857 1.065876 F 20 C 10 0.270998 -0.325587 0.122813 -0.401298 (3 -1)
XX -2.836633 -0.680982 -0.549905 RING 0.008201 0.042416 -1.807810 0.001753 (3 1)
XX -2.722741 -1.560938 0.549107 F 21 C 10 0.251528 -0.432080 0.119255 -0.346766 (3 -1)
XX -2.333784 -2.615166 -0.566758 F 21 N 12 0.015487 0.062407 0.253711 0.001023 (3 -1)

XX -2.278136 -1.654743 1.155211 F 22 C 10 0.261369 -0.370154 0.130159 -0.377799 (3 -1)
XX -2.667743 1.004885 -1.056457 F 23 C 9 0.270922 -0.325845 0.122565 -0.401140 (3 -1)
XX -3.166723 -0.771555 -0.693371 F 23 F 21 0.008769 0.041905 0.367170 0.001038 (3 -1)
XX -2.734135 1.547917 -0.540179 F 24 C 9 0.251457 -0.431932 0.119503 -0.346579 (3 -1)
XX -2.344125 2.611765 0.566827 F 24 N 16 0.015650 0.062753 0.245411 0.000977 (3 -1)
XX -3.161987 0.755596 0.706912 F 24 F 20 0.008678 0.041573 0.381067 0.001050 (3 -1)
XX -2.296256 1.642033 -1.151150 F 25 C 9 0.261534 -0.369440 0.130048 -0.378202 (3 -1)
XX 1.055022 2.303220 0.843122 RING 0.012301 0.060280 -2.758709 0.002403 (3 1)
XX 2.359773 1.675150 1.004187 F 26 C 8 0.265984 -0.366403 0.122469 -0.389026 (3 -1)
XX 1.111468 2.557368 0.831685 F 26 C 14 0.012436 0.055372 3.255498 0.001736 (3 -1)
XX 2.750503 1.067487 0.862592 F 27 C 8 0.274410 -0.328912 0.118662 -0.408880 (3 -1)
XX 3.278320 0.006829 0.005058 F 27 F 17 0.013489 0.062462 0.059089 0.000897 (3 -1)
XX 3.195527 -0.750971 0.687440 F 27 F 18 0.011025 0.052330 0.542982 0.001034 (3 -1)
XX 2.719397 1.608760 0.348553 F 28 C 8 0.256491 -0.413617 0.123507 -0.362826 (3 -1)
XX 2.221527 2.777509 -0.608947 F 28 N 15 0.018060 0.067637 0.151503 0.000297 (3 -1)
XX 3.197962 0.764915 -0.677092 F 28 F 17 0.010992 0.052219 0.548536 0.001039 (3 -1)
XX -1.286664 4.212190 0.437409 H 29 N 16 0.340650 -1.809175 0.047098 -0.504866 (3 -1)
XX -1.608576 3.114628 1.051294 H 30 N 16 0.342957 -1.856315 0.043523 -0.512738 (3 -1)
XX 1.825321 2.666996 0.203139 RING 0.009382 0.045612 -1.357195 0.001957 (3 1)
XX 1.029289 4.355661 -0.198077 H 31 N 15 0.340883 -1.810237 0.048059 -0.505207 (3 -1)
XX 1.460743 3.338483 -0.883092 H 32 N 15 0.343806 -1.859050 0.044806 -0.514121 (3 -1)
XX -1.273822 -4.210500 -0.461472 H 33 N 12 0.340657 -1.809421 0.047053 -0.504895 (3 -1)
XX -1.598257 -3.108301 -1.066218 H 34 N 12 0.342899 -1.856076 0.043507 -0.512635 (3 -1)
XX 1.844733 -2.664350 -0.207243 RING 0.009450 0.046119 -1.363843 0.001980 (3 1)
XX 1.040291 -4.355213 0.175882 H 35 N 13 0.340938 -1.810759 0.047977 -0.505320 (3 -1)
XX 1.474249 -3.339390 0.861877 H 36 N 13 0.343780 -1.859257 0.044716 -0.514138 (3 -1)

M2d Singlet state
XX 0.518028 -1.064753 0.300252 C 2 C 1 0.261624 -0.604616 0.121932 -0.222785 (3 -1)
XX -0.000474 -0.000410 0.297205 RING 0.022824 0.153463 -1.137494 0.007639 (3 1)

XX 1.240916 -0.100490 0.012137 C 3 C 2 0.326668 -0.878409 0.366665 -0.344363 (3 -1)
XX 0.689747 0.971285 0.293255 C 4 C 3 0.260582 -0.600524 0.115815 -0.221004 (3 -1)
XX -0.519261 1.063697 0.298632 C 5 C 4 0.261776 -0.605282 0.122415 -0.223014 (3 -1)
XX -0.690791 -0.972130 0.294541 C 6 C 1 0.260453 -0.600005 0.114888 -0.220807 (3 -1)
XX -1.242056 0.098993 0.011407 C 6 C 5 0.326645 -0.878276 0.366586 -0.344310 (3 -1)
XX 1.628942 -1.217624 -0.284081 C 7 C 2 0.260406 -0.655358 0.045166 -0.221941 (3 -1)
XX 1.819567 0.939542 -0.258940 C 8 C 3 0.263017 -0.668780 0.049286 -0.227155 (3 -1)
XX -1.629998 1.217066 -0.284838 C 9 C 5 0.260284 -0.654817 0.044884 -0.221713 (3 -1)
XX -1.819016 -0.941727 -0.260766 C 10 C 6 0.263087 -0.669079 0.049542 -0.227268 (3 -1)
XX -0.134267 -1.765701 1.008846 C 11 C 1 0.315828 -0.849452 0.421375 -0.331637 (3 -1)
XX -1.855386 -1.842920 0.852452 RING 0.007520 0.038355 -1.429839 0.002202 (3 1)
XX 1.004623 -2.607945 0.652630 RING 0.010588 0.048738 -2.328384 0.001724 (3 1)
XX -0.577990 -2.491339 1.722042 N 12 C 11 0.319274 -1.084890 0.145037 -0.475680 (3 -1)
XX 0.268646 -2.401314 1.827155 N 13 C 11 0.315164 -1.080009 0.128030 -0.451697 (3 -1)
XX 1.852959 1.859659 0.835940 RING 0.007485 0.038265 -1.415006 0.002208 (3 1)
XX 0.132597 1.767136 1.004865 C 14 C 4 0.315726 -0.849174 0.419747 -0.331355 (3 -1)
XX -1.007416 2.615964 0.655300 RING 0.010680 0.048962 -2.438156 0.001715 (3 1)
XX 0.574065 2.498058 1.713187 N 15 C 14 0.319474 -1.084241 0.145535 -0.476997 (3 -1)
XX -0.270308 2.402002 1.822862 N 16 C 14 0.315232 -1.079872 0.128214 -0.452508 (3 -1)
XX 2.118804 -1.702907 -1.117788 F 17 C 7 0.273448 -0.365028 0.129973 -0.405627 (3 -1)
XX 2.366906 -0.328016 -1.021537 RING 0.008762 0.052122 -1.214919 0.002707 (3 1)
XX 2.589947 -1.639800 -0.543176 F 18 C 7 0.268389 -0.360504 0.128162 -0.394437 (3 -1)
XX 2.073333 -2.166797 -0.547836 F 19 C 7 0.270079 -0.336519 0.121478 -0.399052 (3 -1)
XX 1.456389 -2.785378 0.937717 F 19 N 13 0.011865 0.046088 0.525262 0.001074 (3 -1)
XX -2.357259 0.327854 -1.029128 RING 0.008792 0.052199 -1.240532 0.002704 (3 1)
XX -2.754956 0.525269 -1.250184 RING 0.009809 0.057100 -1.549044 0.002304 (3 1)
XX -2.631779 -1.176659 -0.948796 F 20 C 10 0.279586 -0.307505 0.125953 -0.419794 (3 -1)
XX -2.300017 -1.789233 -0.719709 F 21 C 10 0.272361 -0.343282 0.125579 -0.404025 (3 -1)
XX -2.780828 -1.441579 -0.268382 F 22 C 10 0.256455 -0.394546 0.133038 -0.364519 (3 -1)
XX -2.624585 -2.234695 1.085188 F 22 N 12 0.017013 0.058903 0.021174 -0.000271 (3 -1)

XX -2.122391 1.701600 -1.117882 F 23 C 9 0.273427 -0.365375 0.129711 -0.405579 (3 -1)
XX -2.526922 0.425999 -1.787343 F 23 F 20 0.012594 0.055708 0.132550 0.000580 (3 -1)
XX -2.590489 1.642884 -0.540152 F 24 C 9 0.268305 -0.360889 0.128106 -0.394235 (3 -1)
XX -3.195564 0.341197 -0.971711 F 24 F 20 0.012080 0.054977 0.128722 0.000775 (3 -1)
XX -2.071089 2.167119 -0.550112 F 25 C 9 0.270233 -0.335913 0.121453 -0.399411 (3 -1)
XX -1.441363 2.786839 0.930334 F 25 N 16 0.011809 0.045545 0.511727 0.001029 (3 -1)
XX 2.784905 1.432546 -0.265182 F 26 C 8 0.256813 -0.395172 0.132815 -0.365372 (3 -1)
XX 2.623825 2.238507 1.072795 F 26 N 15 0.016360 0.057389 0.017833 -0.000140 (3 -1)
XX 2.743010 -0.524087 -1.263817 RING 0.009778 0.056923 -1.544563 0.002299 (3 1)
XX 2.631253 1.175550 -0.947976 F 27 C 8 0.279491 -0.308962 0.126045 -0.419577 (3 -1)
XX 2.519314 -0.426707 -1.788650 F 27 F 17 0.012384 0.054980 0.138619 0.000607 (3 -1)
XX 3.193143 -0.334936 -0.978287 F 27 F 18 0.012210 0.055420 0.115232 0.000758 (3 -1)
XX 2.305288 1.788407 -0.710733 F 28 C 8 0.272035 -0.343321 0.125534 -0.403303 (3 -1)
XX -1.049485 3.348145 2.434677 H 29 N 16 0.340122 -1.792409 0.049967 -0.502615 (3 -1)
XX -1.606696 2.248808 2.122971 H 30 N 16 0.344771 -1.831907 0.048061 -0.511606 (3 -1)
XX 1.320477 3.061515 2.724391 H 31 N 15 0.342025 -1.809260 0.051975 -0.506670 (3 -1)
XX 1.577269 2.326282 0.509641 CAGE 0.006756 0.035561 -0.915315 0.001874 (3 3)
XX 1.955502 2.762145 1.647306 H 32 N 15 0.344693 -1.848034 0.047211 -0.514035 (3 -1)
XX -1.321378 -3.045982 2.738730 H 33 N 12 0.341988 -1.808508 0.051921 -0.506553 (3 -1)
XX -1.962036 -2.740444 1.667468 H 34 N 12 0.344569 -1.847177 0.047104 -0.513852 (3 -1)
XX 1.039715 -3.351653 2.439887 H 35 N 13 0.340148 -1.792271 0.049882 -0.502638 (3 -1)
XX 1.608682 -2.263802 2.114084 H 36 N 13 0.344889 -1.832575 0.047825 -0.511817 (3 -1)

M2e Triplet state
XX 1.099773 0.618585 0.009218 C 2 C 1 0.285516 -0.706254 0.223429 -0.261966 (3 -1)
XX 0.000125 0.000074 -0.000734 RING 0.017140 0.126622 -1.198492 0.007464 (3 1)
XX -0.000153 1.224307 -0.000768 C 3 C 2 0.281435 -0.689380 0.226040 -0.254939 (3 -1)
XX -1.099704 0.618168 -0.009781 C 4 C 3 0.285570 -0.706525 0.223385 -0.262061 (3 -1)
XX -1.099553 -0.618412 0.009061 C 5 C 4 0.285553 -0.706433 0.223437 -0.262033 (3 -1)
XX 1.099949 -0.618005 -0.009982 C 6 C 1 0.285496 -0.706128 0.223393 -0.261926 (3 -1)

```
XX  0.000260 -1.224151 -0.001077 C   6 C   5  0.281379 -0.689129  0.225909 -0.254841 (3 -1)
XX  2.271374  1.386009 -0.139750 RING         0.012352  0.071114 -1.285670  0.004162 (3 1)
XX  1.014201  1.805995  0.078486 C   7 C   2  0.288680 -0.772948  0.153653 -0.287700 (3 -1)
XX -1.014986  1.805421 -0.081883 C   8 C   3  0.288639 -0.772672  0.153552 -0.287565 (3 -1)
XX -2.270993  1.386503  0.143412 RING         0.012373  0.071034 -1.286098  0.004150 (3 1)
XX -2.271257 -1.386360 -0.141280 RING         0.012363  0.071040 -1.286042  0.004153 (3 1)
XX -1.014335 -1.805750  0.079151 C   9 C   5  0.288668 -0.772821  0.153677 -0.287644 (3 -1)
XX  2.145883  0.000256  0.001546 C  10 C   1  0.286174 -0.737774  0.202507 -0.268081 (3 -1)
XX  2.270110 -1.385776  0.142736 RING         0.012383  0.071255 -1.285055  0.004168 (3 1)
XX  2.512395  2.111631 -0.360019 N  11 C   7  0.020040  0.070022  0.226241  0.001551 (3 -1)
XX  3.173936  0.371890 -0.166867 N  11 C  10  0.332664 -1.085006  0.158907 -0.527162 (3 -1)
XX  3.172807 -0.372205  0.174581 N  12 C  10  0.332648 -1.085441  0.158771 -0.527041 (3 -1)
XX -2.145509 -0.000119  0.001136 C  13 C   4  0.286268 -0.738246  0.202459 -0.268251 (3 -1)
XX -2.508920  2.106151  0.365234 N  14 C   8  0.019833  0.069724  0.219112  0.001596 (3 -1)
XX -3.172500  0.371360  0.174976 N  14 C  13  0.332646 -1.084752  0.158883 -0.527149 (3 -1)
XX -2.510695 -2.107973 -0.363083 N  15 C   9  0.019906  0.069814  0.222019  0.001580 (3 -1)
XX -3.173225 -0.371475 -0.169096 N  15 C  13  0.332647 -1.084988  0.158963 -0.527106 (3 -1)
XX  1.014861 -1.805298 -0.082417 C  16 C   6  0.288676 -0.772851  0.153746 -0.287670 (3 -1)
XX  2.508791 -2.109330  0.361970 C  16 N  12  0.019994  0.070010  0.220913  0.001562 (3 -1)
XX -4.353288 -1.117365 -0.253340 H  17 N  15  0.341535 -1.819666  0.046208 -0.507039 (3 -1)
XX -3.284225 -1.665592 -0.748358 H  18 N  15  0.341721 -1.854123  0.041648 -0.512047 (3 -1)
XX -4.351839  1.118137  0.263110 H  19 N  14  0.341521 -1.819596  0.046233 -0.507018 (3 -1)
XX -3.281161  1.664779  0.756243 H  20 N  14  0.341756 -1.854317  0.041673 -0.512088 (3 -1)
XX  4.352203 -1.118667  0.261492 H  21 N  12  0.341541 -1.819598  0.046223 -0.507039 (3 -1)
XX  3.280770 -1.668459  0.749571 H  22 N  12  0.341740 -1.854214  0.041645 -0.512089 (3 -1)
XX  4.355263  1.115873 -0.249866 H  23 N  11  0.341558 -1.819730  0.046211 -0.507070 (3 -1)
XX  3.286332  1.668901 -0.740176 H  24 N  11  0.341652 -1.853720  0.041630 -0.511952 (3 -1)
XX  1.538534  2.842471  0.172593 N  25 C   7  0.457901  0.110985  0.043749 -0.819651 (3 -1)
XX -1.540109  2.841071 -0.179627 N  26 C   8  0.457907  0.111873  0.043922 -0.819633 (3 -1)
XX  1.539367 -2.841270 -0.180377 N  27 C  16  0.457892  0.111261  0.043889 -0.819620 (3 -1)
```

XX -1.539008 -2.841841 0.174853 N 28 C   9 0.457898 0.111379 0.043843 -0.819627 (3 -1)

M2e Singlet state
XX 1.086458 0.589395 -0.017001 C   2 C   1 0.269678 -0.645731 0.161938 -0.236174 (3 -1)
XX -0.000063 0.000006 -0.152090 RING   0.018928 0.137276 -1.189409 0.007689 (3 1)
XX 0.006255 1.223300 -0.184768 C   3 C   2 0.320129 -0.865644 0.331326 -0.328720 (3 -1)
XX -1.082418 0.607836 -0.025586 C   4 C   3 0.267889 -0.639280 0.155792 -0.233072 (3 -1)
XX -1.086556 -0.589395 -0.017130 C   5 C   4 0.269657 -0.645614 0.161997 -0.236135 (3 -1)
XX 1.082315 -0.607755 -0.025200 C   6 C   1 0.267874 -0.639230 0.155679 -0.233051 (3 -1)
XX -0.006278 -1.223314 -0.184512 C   6 C   5 0.320147 -0.865744 0.331347 -0.328757 (3 -1)
XX 1.019103 1.764905 -0.363139 C   7 C   2 0.280851 -0.750660 0.083571 -0.270264 (3 -1)
XX -0.990939 1.779989 -0.376136 C   8 C   3 0.282618 -0.762059 0.087541 -0.275009 (3 -1)
XX -2.238988 1.430296 0.114728 RING   0.010365 0.057724 -1.338962 0.003458 (3 1)
XX -1.019082 -1.765075 -0.362746 C   9 C   5 0.280825 -0.750533 0.083556 -0.270217 (3 -1)
XX 2.238682 -1.430020 0.114700 RING   0.010375 0.057775 -1.339000 0.003460 (3 1)
XX 0.990942 -1.779814 -0.376124 C 10 C   6 0.282630 -0.762118 0.087541 -0.275032 (3 -1)
XX 2.060806 -0.004374 0.359391 C 11 C   1 0.308602 -0.815302 0.368309 -0.311384 (3 -1)
XX 2.111271 1.479666 0.375028 RING   0.011578 0.061405 -1.341343 0.003597 (3 1)
XX 2.281280 2.077042 0.409574 N 12 C   7 0.014555 0.056144 0.140548 0.002176 (3 -1)
XX 2.955275 0.430517 0.839057 N 12 C 11 0.318509 -1.087491 0.147910 -0.466196 (3 -1)
XX 3.043030 -0.393628 0.662334 N 13 C 11 0.322717 -1.080405 0.162109 -0.491273 (3 -1)
XX -2.060963 0.004449 0.358868 C 14 C   4 0.308553 -0.815099 0.368189 -0.311306 (3 -1)
XX -2.111472 -1.479511 0.374816 RING   0.011580 0.061419 -1.341387 0.003598 (3 1)
XX -3.043250 0.393476 0.661861 N 15 C 14 0.322764 -1.080222 0.162170 -0.491543 (3 -1)
XX -2.281422 -2.077099 0.409644 N 16 C   9 0.014561 0.056164 0.140491 0.002176 (3 -1)
XX -2.955573 -0.430526 0.838473 N 16 C 14 0.318502 -1.087490 0.147819 -0.466158 (3 -1)
XX -4.048244 -1.234583 1.062403 H 17 N 16 0.340388 -1.801385 0.049890 -0.504095 (3 -1)
XX -2.901356 -1.774858 1.210163 H 18 N 16 0.345127 -1.849337 0.046232 -0.514655 (3 -1)
XX -4.066576 1.114776 1.270025 H 19 N 15 0.342067 -1.815928 0.052371 -0.507434 (3 -1)
XX -3.381627 1.744679 0.355109 H 20 N 15 0.342783 -1.846296 0.047127 -0.512387 (3 -1)

XX 4.064892 -1.116105 1.271354 H 21 N 13 0.342057 -1.815814 0.052367 -0.507414 (3 -1)
XX 3.381433 -1.744282 0.354162 H 22 N 13 0.342776 -1.846208 0.047125 -0.512372 (3 -1)
XX 4.047928 1.234498 1.063109 H 23 N 12 0.340391 -1.801453 0.049872 -0.504106 (3 -1)
XX 2.900973 1.774843 1.210428 H 24 N 12 0.345113 -1.849220 0.046223 -0.514626 (3 -1)
XX 1.598988 2.728067 -0.679722 N 25 C 7 0.462410 0.226574 0.033682 -0.826665 (3 -1)
XX -1.557997 2.748267 -0.705993 N 26 C 8 0.462134 0.195702 0.028148 -0.827144 (3 -1)
XX -2.593282 2.174423 -0.088566 N 26 N 15 0.018037 0.061670 0.499126 0.001577 (3 -1)
XX -1.598679 -2.728493 -0.679210 N 27 C 9 0.462412 0.226664 0.033672 -0.826668 (3 -1)
XX 1.558309 -2.747728 -0.706456 N 28 C 10 0.462134 0.195628 0.028129 -0.827146 (3 -1)
XX 2.593377 -2.174121 -0.089424 N 28 N 13 0.018063 0.061746 0.500418 0.001576 (3 -1)

M3a Triplet state
XX 1.091390 -0.594657 -0.057701 C 2 C 1 0.296236 -0.760628 0.241845 -0.281743 (3 -1)
XX 0.010344 0.000146 -0.001650 RING 0.018477 0.142876 -1.171484 0.008243 (3 1)
XX 0.000861 -1.215813 -0.103032 C 3 C 2 0.309943 -0.834215 0.285840 -0.305393 (3 -1)
XX -1.069360 -0.599523 -0.034454 C 4 C 3 0.301687 -0.781443 0.267724 -0.291736 (3 -1)
XX -1.069051 0.600302 0.031363 C 5 C 4 0.301736 -0.781783 0.267560 -0.291846 (3 -1)
XX 1.091876 0.594293 0.054376 C 6 C 1 0.296271 -0.760733 0.242191 -0.281831 (3 -1)
XX 0.001794 1.216164 0.099052 C 6 C 5 0.309936 -0.834290 0.285446 -0.305380 (3 -1)
XX 0.952204 -1.677761 -0.212331 N 7 C 2 0.295649 -0.938203 0.097877 -0.399710 (3 -1)
XX -0.031695 -2.550332 -0.511544 RING 0.017099 0.093372 -2.365627 0.004658 (3 1)
XX -0.951505 -1.690732 -0.140686 N 8 C 3 0.284729 -0.871403 0.094031 -0.368197 (3 -1)
XX -0.950440 1.691532 0.136566 N 9 C 5 0.284800 -0.871845 0.094506 -0.368358 (3 -1)
XX -0.031379 2.555219 0.509299 RING 0.017018 0.092642 -2.429163 0.004619 (3 1)
XX 2.177712 -0.000781 -0.000572 C 10 C 1 0.273787 -0.656320 0.148070 -0.240584 (3 -1)
XX 0.953106 1.677728 0.208222 C 10 N 9 0.295516 -0.937351 0.097788 -0.399445 (3 -1)
XX 3.306584 -0.491760 0.415495 C 11 C 10 0.254406 -0.587734 0.075074 -0.210065 (3 -1)
XX 3.308804 0.489514 -0.411828 C 12 C 10 0.254388 -0.587643 0.075121 -0.210028 (3 -1)
XX -2.157982 0.000832 0.000390 C 13 C 4 0.265089 -0.626018 0.083739 -0.225989 (3 -1)
XX -3.290732 -0.333004 0.553687 C 14 C 13 0.254654 -0.587189 0.081390 -0.210602 (3 -1)

XX -3.293638 0.333954 -0.547802 C 15 C 13 0.254645 -0.587109 0.081507 -0.210590 (3 -1)
XX 4.124922 1.352664 -0.446484 H 16 C 12 0.268573 -0.881199 0.010398 -0.267085 (3 -1)
XX 4.124219 0.617878 -1.251330 H 17 C 12 0.269575 -0.891866 0.007458 -0.269256 (3 -1)
XX 3.315658 1.378447 -1.206590 H 18 C 12 0.280328 -0.981071 0.006528 -0.288617 (3 -1)
XX 4.114343 -0.618888 1.262503 H 19 C 11 0.269485 -0.891155 0.007467 -0.269090 (3 -1)
XX 3.308416 -1.381637 1.208769 H 20 C 11 0.280393 -0.981663 0.006494 -0.288755 (3 -1)
XX 4.124848 -1.352285 0.456290 H 21 C 11 0.268679 -0.882063 0.010360 -0.267283 (3 -1)
XX -4.139905 -1.104474 0.864905 H 22 C 14 0.269513 -0.889865 0.010083 -0.268766 (3 -1)
XX -3.273175 -0.981020 1.549546 H 23 C 14 0.278269 -0.962429 0.007728 -0.284700 (3 -1)
XX -4.043343 -0.191429 1.456746 H 24 C 14 0.268852 -0.888018 0.007520 -0.267929 (3 -1)
XX -4.146010 1.103341 -0.855905 H 25 C 15 0.269516 -0.889913 0.010130 -0.268769 (3 -1)
XX -3.281978 0.981553 -1.544342 H 26 C 15 0.278230 -0.962087 0.007769 -0.284618 (3 -1)
XX -4.049815 0.190329 -1.447709 H 27 C 15 0.268796 -0.887573 0.007538 -0.267823 (3 -1)
XX 0.904146 -2.919744 -0.738599 H 28 N 7 0.339408 -1.779846 0.046965 -0.501064 (3 -1)
XX -2.093490 -2.399375 -0.329086 H 28 H 22 0.343932 -1.793894 0.047492 -0.506586 (3 -1)
XX 2.032853 -2.391971 -0.622974 H 29 N 7 0.343651 -1.794249 0.046753 -0.506509 (3 -1)
XX -1.173727 -2.969004 0.265042 H 30 N 8 0.338332 -1.744970 0.050128 -0.495039 (3 -1)
XX -1.166077 2.971917 -0.265471 H 32 N 9 0.338360 -1.745472 0.050046 -0.495135 (3 -1)
XX -2.092990 2.401344 0.316687 H 33 N 9 0.343937 -1.794103 0.047417 -0.506625 (3 -1)
XX 0.907111 2.919003 0.737039 H 35 N 34 0.339408 -1.779442 0.047036 -0.501005 (3 -1)
XX 2.035279 2.392586 0.615161 H 36 N 34 0.343719 -1.794877 0.046752 -0.506656 (3 -1)

M3a Singlet state
XX -1.039412 0.642595 -0.281852 C 2 C 1 0.263278 -0.631662 0.123342 -0.222912 (3 -1)
XX 0.002263 0.014928 -0.490395 RING 0.019337 0.143047 -1.134962 0.007744 (3 1)
XX 0.020808 1.246728 -0.508132 C 3 C 2 0.333237 -0.919388 0.459292 -0.356300 (3 -1)
XX 1.068453 0.604109 -0.284397 C 4 C 3 0.264996 -0.639994 0.107427 -0.225687 (3 -1)
XX 1.054241 -0.617265 -0.291890 C 5 C 4 0.266112 -0.648368 0.101227 -0.227466 (3 -1)
XX -1.050846 -0.591198 -0.293572 C 6 C 1 0.264109 -0.629610 0.125496 -0.224138 (3 -1)
XX -0.019399 -1.217322 -0.528696 C 6 C 5 0.335883 -0.942508 0.450110 -0.362026 (3 -1)

XX -0.920540 1.692329 -0.624467 N 7 C 2 0.287711 -0.874382 0.105601 -0.393030 (3 -1)
XX 0.997526 1.643228 -0.696551 N 8 C 3 0.284200 -0.867336 0.111978 -0.369123 (3 -1)
XX 0.940657 -1.645201 -0.708871 N 9 C 5 0.294639 -0.928848 0.106848 -0.399766 (3 -1)
XX -0.030059 -2.422350 -1.240676 RING 0.016532 0.085904 -2.395244 0.004120 (3 1)
XX -1.982885 0.023272 0.267607 C 10 C 1 0.324268 -0.869803 0.354724 -0.338594 (3 -1)
XX -2.070849 1.464438 0.150385 RING 0.010940 0.059338 -1.285197 0.003235 (3 1)
XX -1.976893 -1.555689 0.234948 RING 0.011094 0.051646 -1.432002 0.002543 (3 1)
XX -2.276717 2.254480 0.157947 C 11 N 7 0.020177 0.062222 0.082170 0.000894 (3 -1)
XX -2.949325 0.640353 0.854752 C 11 C 10 0.252186 -0.588568 0.041680 -0.206813 (3 -1)
XX -2.889019 -0.630874 0.869190 C 12 C 10 0.250213 -0.580021 0.039226 -0.203264 (3 -1)
XX 1.982887 -0.019789 0.296860 C 13 C 4 0.326981 -0.882859 0.366068 -0.344603 (3 -1)
XX 1.988948 -1.536139 0.183260 RING 0.010554 0.055338 -1.380854 0.003101 (3 1)
XX 1.980364 1.543883 0.241121 RING 0.010562 0.054239 -1.415221 0.003019 (3 1)
XX 2.891993 0.610507 0.923658 C 14 C 13 0.251622 -0.586226 0.039284 -0.205673 (3 -1)
XX 2.901524 -0.669805 0.899091 C 15 C 13 0.252160 -0.588900 0.039523 -0.206617 (3 -1)
XX -2.778351 -1.784667 1.169488 H 16 C 12 0.280181 -0.972490 0.007553 -0.288555 (3 -1)
XX -3.468584 -1.148782 1.750286 H 17 C 12 0.275431 -0.934690 0.008912 -0.279645 (3 -1)
XX -3.739590 -1.454116 0.737530 H 18 C 12 0.270163 -0.893486 0.009730 -0.270317 (3 -1)
XX -3.374837 1.220049 1.768820 H 19 C 11 0.272056 -0.907601 0.010223 -0.273829 (3 -1)
XX -3.027191 1.833498 0.899406 H 20 C 11 0.286027 -1.037698 0.006860 -0.301230 (3 -1)
XX -3.962225 1.224341 0.851120 H 21 C 11 0.269881 -0.890560 0.012146 -0.270001 (3 -1)
XX 3.836129 1.316619 0.900764 H 22 C 14 0.270218 -0.893692 0.011344 -0.270495 (3 -1)
XX 2.851369 1.795203 1.093567 H 23 C 14 0.282742 -1.001945 0.007491 -0.293598 (3 -1)
XX 3.339568 1.150342 1.857916 H 24 C 14 0.273564 -0.921097 0.009315 -0.276519 (3 -1)
XX 2.899094 -1.863527 0.975837 H 25 C 15 0.283774 -1.013512 0.007325 -0.295970 (3 -1)
XX 3.871860 -1.330774 0.878824 H 26 C 15 0.270425 -0.895385 0.011329 -0.270922 (3 -1)
XX 3.317225 -1.258946 1.815450 H 27 C 15 0.272858 -0.915009 0.009695 -0.275212 (3 -1)
XX -0.944776 3.036825 -0.961749 H 28 N 7 0.343510 -1.790189 0.056141 -0.506001 (3 -1)
XX 2.172046 2.199679 0.194942 H 28 H 22 0.014188 0.053324 0.128124 0.002003 (3 -1)
XX -1.896880 2.332400 -1.369798 H 29 N 7 0.339636 -1.758764 0.056164 -0.498181 (3 -1)

XX 2.164001 2.135777 -1.165996 H 30 N   8 0.342851 -1.786151 0.047688 -0.504641 (3 -1)
XX 1.135122 2.652875 -1.582467 H 31 N   8 0.338214 -1.733410 0.049498 -0.493504 (3 -1)
XX 0.922608 -2.764819 -1.458714 H 32 N   9 0.337702 -1.770657 0.045654 -0.498009 (3 -1)
XX -0.961441 -1.642431 -0.697358 H 32 C 10 0.279372 -0.830201 0.102512 -0.357425 (3 -1)
XX 2.164021 -2.258172 0.132069 H 32 H 27 0.015939 0.053917 0.087132 0.001481 (3 -1)
XX 2.024302 -2.183087 -1.320739 H 33 N   9 0.341501 -1.773010 0.048134 -0.501443 (3 -1)
XX -2.112689 -2.071941 0.201623 N 34 C 12 0.012461 0.044418 0.269692 0.001764 (3 -1)
XX -1.351561 -2.951812 -0.604693 H 35 N 34 0.338384 -1.733864 0.051570 -0.493552 (3 -1)
XX -0.071478 -2.583007 -1.356317 H 36 H 32 0.016761 0.066864 1.039604 0.002297 (3 -1)
XX -2.097901 -2.185847 -1.201976 H 36 N 34 0.344182 -1.792452 0.048831 -0.506413 (3 -1)

M3b Triplet state
XX -1.064915 -0.610921 -0.010553 C   2 C   1 0.299691 -0.772636 0.215580 -0.287151 (3 -1)
XX -0.000059 0.000286 -0.001759 RING   0.019084 0.147994 -1.174952 0.008440 (3 1)
XX -0.000223 -1.223049 -0.001922 C   3 C   2 0.306899 -0.810036 0.218524 -0.300061 (3 -1)
XX 1.064665 -0.611374 0.007337 C   4 C   3 0.299698 -0.772651 0.215627 -0.287167 (3 -1)
XX 1.064855 0.611451 -0.010406 C   5 C   4 0.299735 -0.772856 0.215654 -0.287236 (3 -1)
XX -1.064779 0.611970 0.006853 C   6 C   1 0.299667 -0.772492 0.215625 -0.287108 (3 -1)
XX 0.000149 1.223599 -0.002228 C   6 C   5 0.306872 -0.809890 0.218485 -0.300007 (3 -1)
XX -1.074209 -1.885936 -0.064780 C   7 C   2 0.250239 -0.581652 0.035008 -0.203117 (3 -1)
XX 1.073670 -1.886313 0.060237 C   8 C   3 0.250336 -0.582108 0.035014 -0.203266 (3 -1)
XX 1.074276 1.886417 -0.064338 C   9 C   5 0.250247 -0.581687 0.035016 -0.203129 (3 -1)
XX 2.159516 0.000013 -0.000052 C 11 C   1 0.266744 -0.636471 0.088619 -0.228087 (3 -1)
XX -1.073473 1.887098 0.059847 C 10 C   6 0.250328 -0.582064 0.035015 -0.203253 (3 -1)
XX -2.159641 0.000456 0.000095 C 14 C   4 0.266720 -0.636311 0.088885 -0.228049 (3 -1)
XX -3.294516 0.311075 -0.558992 C 12 C 11 0.255774 -0.593049 0.078946 -0.212317 (3 -1)
XX -3.290799 -0.311671 0.564928 C 13 C 11 0.255816 -0.593252 0.078884 -0.212387 (3 -1)
XX 3.293826 -0.310768 -0.560065 C 15 C 14 0.255786 -0.593105 0.078915 -0.212337 (3 -1)
XX 3.291097 0.310516 0.564779 C 16 C 14 0.255790 -0.593120 0.078890 -0.212346 (3 -1)
XX 1.130860 2.964363 -0.551785 H 17 C   9 0.276183 -0.939641 0.008767 -0.281048 (3 -1)

XX 1.460724 2.866910 0.488943 H 18 C   9 0.271986 -0.908301 0.009593 -0.273521 (3 -1)
XX 2.080915 2.453249 -0.322350 H 19 C   9 0.279355 -0.965534 0.007234 -0.287016 (3 -1)
XX 2.080161 -2.453481 0.317743 H 20 C   8 0.279347 -0.965468 0.007213 -0.286989 (3 -1)
XX 1.129830 -2.964583 0.546785 H 21 C   8 0.276179 -0.939619 0.008746 -0.281038 (3 -1)
XX 1.459626 -2.866207 -0.493864 H 22 C   8 0.271988 -0.908315 0.009557 -0.273526 (3 -1)
XX -1.132322 -2.962259 -0.555567 H 23 C   7 0.276133 -0.939289 0.008764 -0.280957 (3 -1)
XX -1.457758 -2.868533 0.486945 H 24 C   7 0.271989 -0.908314 0.009598 -0.273517 (3 -1)
XX -2.081636 -2.452504 -0.320260 H 25 C   7 0.279401 -0.965846 0.007222 -0.287100 (3 -1)
XX -1.131151 2.963580 0.549959 H 26 C 10 0.276121 -0.939195 0.008753 -0.280933 (3 -1)
XX -1.455123 2.869484 -0.492835 H 27 C 10 0.272000 -0.908381 0.009569 -0.273546 (3 -1)
XX -2.080837 2.454544 0.313686 H 28 C 10 0.279412 -0.965990 0.007202 -0.287118 (3 -1)
XX 4.147473 1.060237 0.903489 H 29 C 16 0.270287 -0.896644 0.008640 -0.270100 (3 -1)
XX 3.278848 0.924570 1.580435 H 30 C 16 0.278095 -0.959305 0.007159 -0.284372 (3 -1)
XX 4.035543 0.127199 1.464835 H 31 C 16 0.268699 -0.886087 0.007482 -0.267595 (3 -1)
XX 4.150553 -1.062198 -0.894069 H 32 C 15 0.270257 -0.896395 0.008646 -0.270046 (3 -1)
XX 3.287023 -0.923835 -1.576712 H 33 C 15 0.278092 -0.959300 0.007172 -0.284365 (3 -1)
XX 4.044553 -0.128097 -1.454736 H 34 C 15 0.268734 -0.886343 0.007485 -0.267659 (3 -1)
XX -4.145973 -1.063359 0.902323 H 35 C 13 0.270258 -0.896402 0.008633 -0.270041 (3 -1)
XX -3.277702 -0.927274 1.579589 H 36 C 13 0.278106 -0.959381 0.007144 -0.284390 (3 -1)
XX -4.035720 -0.130921 1.465039 H 37 C 13 0.268718 -0.886248 0.007479 -0.267629 (3 -1)
XX -4.155552 1.058022 -0.891666 H 38 C 12 0.270311 -0.896808 0.008659 -0.270142 (3 -1)
XX -3.289655 0.928808 -1.573149 H 39 C 12 0.278065 -0.959099 0.007191 -0.284308 (3 -1)
XX -4.041687 0.127245 -1.456344 H 40 C 12 0.268689 -0.886008 0.007463 -0.267584 (3 -1)

M3b Singlet state
XX 1.026528 -0.625395 -0.316758 C   2 C   1 0.261001 -0.618481 0.079265 -0.219317 (3 -1)
XX -0.000166 0.000193 -0.452183 RING 0.020422 0.151339 -1.117908 0.008108 (3 1)
XX -0.001187 -1.237385 -0.569457 C   3 C   2 0.335108 -0.927633 0.366469 -0.360774 (3 -1)
XX -1.027722 -0.624023 -0.316080 C   4 C   3 0.261034 -0.618595 0.079379 -0.219369 (3 -1)
XX -1.026880 0.625787 -0.316664 C   5 C   4 0.261006 -0.618513 0.079208 -0.219325 (3 -1)

XX	1.027384	0.624435	-0.315896	C	6	C	1	0.261084	-0.618839	0.079375	-0.219448	(3 -1)
XX	0.000867	1.237809	-0.569149	C	6	C	5	0.335094	-0.927526	0.366522	-0.360745	(3 -1)
XX	1.876169	-1.711228	0.258386	RING				0.010008	0.046947	-2.126709	0.002718	(3 1)
XX	1.077825	-1.814508	-0.855030	C	7	C	2	0.248625	-0.572679	0.040802	-0.201275	(3 -1)
XX	-1.081361	-1.813432	-0.853181	C	8	C	3	0.248563	-0.572387	0.040796	-0.201183	(3 -1)
XX	-1.871494	-1.727994	0.264326	RING				0.010012	0.046617	-2.262202	0.002695	(3 1)
XX	-1.876657	1.708281	0.258374	RING				0.010019	0.047050	-2.117219	0.002724	(3 1)
XX	-1.078199	1.814884	-0.854932	C	9	C	5	0.248580	-0.572463	0.040786	-0.201207	(3 -1)
XX	1.874129	1.723917	0.263445	RING				0.009996	0.046620	-2.216521	0.002698	(3 1)
XX	1.081127	1.813834	-0.852696	C	10	C	6	0.248567	-0.572406	0.040796	-0.201189	(3 -1)
XX	1.898852	-0.001183	0.332640	C	11	C	1	0.328888	-0.893167	0.368487	-0.348813	(3 -1)
XX	2.117672	2.139537	0.135464	C	12	C	10	0.010725	0.045597	0.084293	0.002382	(3 -1)
XX	2.753616	0.634433	1.029856	C	12	C	11	0.252170	-0.589434	0.041173	-0.206251	(3 -1)
XX	2.133103	-2.146564	0.120213	C	13	C	7	0.010880	0.046200	0.162978	0.002399	(3 -1)
XX	2.754472	-0.636949	1.029068	C	13	C	11	0.252336	-0.590153	0.041491	-0.206502	(3 -1)
XX	-1.899102	0.001376	0.332899	C	14	C	4	0.328850	-0.892916	0.368538	-0.348727	(3 -1)
XX	-2.111782	-2.138256	0.137993	C	15	C	8	0.010706	0.045530	0.062257	0.002379	(3 -1)
XX	-2.753029	-0.634818	1.030193	C	15	C	14	0.252193	-0.589558	0.041059	-0.206287	(3 -1)
XX	-2.133769	2.150373	0.119138	C	16	C	9	0.010925	0.046325	0.150925	0.002393	(3 -1)
XX	-2.754700	0.636810	1.029706	C	16	C	14	0.252333	-0.590130	0.041569	-0.206499	(3 -1)
XX	-1.240600	2.522884	-1.788570	H	17	C	9	0.273387	-0.920116	0.008942	-0.275965	(3 -1)
XX	-2.131858	2.229511	-1.207735	H	18	C	9	0.279694	-0.968679	0.008634	-0.287316	(3 -1)
XX	-1.412150	2.960268	-0.798535	H	19	C	9	0.272818	-0.914859	0.010568	-0.274876	(3 -1)
XX	-2.135605	-2.225347	-1.207868	H	20	C	8	0.279594	-0.967962	0.008681	-0.287113	(3 -1)
XX	-1.243344	-2.524725	-1.784366	H	21	C	8	0.273426	-0.920375	0.008950	-0.276028	(3 -1)
XX	-1.420359	-2.957203	-0.793131	H	22	C	8	0.272777	-0.914555	0.010567	-0.274796	(3 -1)
XX	1.241744	-2.520777	-1.789609	H	23	C	7	0.273322	-0.919635	0.008933	-0.275836	(3 -1)
XX	2.131862	-2.230600	-1.205394	H	24	C	7	0.279691	-0.968644	0.008636	-0.287303	(3 -1)
XX	1.409293	-2.960545	-0.799869	H	25	C	7	0.272846	-0.915083	0.010552	-0.274930	(3 -1)
XX	1.243946	2.524269	-1.784296	H	26	C	10	0.273423	-0.920340	0.008940	-0.276026	(3 -1)

XX 1.419334 2.957886 -0.793241 H 27 C 10 0.272783 -0.914603 0.010570 -0.274803 (3 -1)
XX 2.135585 2.226051 -1.206369 H 28 C 10 0.279619 -0.968134 0.008675 -0.287162 (3 -1)
XX -3.136053 1.200489 1.985389 H 29 C 16 0.273838 -0.922488 0.008669 -0.276875 (3 -1)
XX -2.716276 1.821869 1.176422 H 30 C 16 0.281393 -0.982366 0.007176 -0.290819 (3 -1)
XX -3.705325 1.340251 1.061723 H 31 C 16 0.271058 -0.902281 0.009156 -0.271919 (3 -1)
XX -3.692786 -1.354489 1.045595 H 32 C 15 0.271034 -0.902087 0.009130 -0.271881 (3 -1)
XX -2.699099 -1.816373 1.195916 H 33 C 15 0.281166 -0.980667 0.007216 -0.290379 (3 -1)
XX -3.152989 -1.190919 1.983028 H 34 C 15 0.274038 -0.923881 0.008739 -0.277221 (3 -1)
XX 3.140106 -1.199216 1.983960 H 35 C 13 0.273867 -0.922679 0.008696 -0.276918 (3 -1)
XX 2.713454 -1.821464 1.179297 H 36 C 13 0.281349 -0.982035 0.007188 -0.290734 (3 -1)
XX 3.703254 -1.343170 1.057197 H 37 C 13 0.271043 -0.902161 0.009152 -0.271892 (3 -1)
XX 3.695668 1.350825 1.047779 H 38 C 12 0.271025 -0.902019 0.009153 -0.271861 (3 -1)
XX 2.703240 1.816876 1.193801 H 39 C 12 0.281149 -0.980561 0.007248 -0.290333 (3 -1)
XX 3.151654 1.190545 1.983289 H 40 C 12 0.274035 -0.923843 0.008746 -0.277222 (3 -1)

M3c Triplet state
XX -1.074713 0.602330 0.000183 C 2 C 1 0.301286 -0.795303 0.176267 -0.289608 (3 -1)
XX 0.000002 -0.000000 -0.000022 RING 0.019876 0.154184 -1.208694 0.008600 (3 1)
XX -0.000288 1.196789 0.000241 C 3 C 2 0.313767 -0.855849 0.221023 -0.314989 (3 -1)
XX 1.074457 0.602998 0.000090 C 4 C 3 0.301274 -0.795241 0.176267 -0.289585 (3 -1)
XX 1.074716 -0.602331 -0.000182 C 5 C 4 0.301285 -0.795295 0.176265 -0.289605 (3 -1)
XX -1.074455 -0.602998 -0.000078 C 6 C 1 0.301276 -0.795253 0.176271 -0.289589 (3 -1)
XX 0.000288 -1.196789 -0.000303 C 6 C 5 0.313766 -0.855842 0.221022 -0.314987 (3 -1)
XX -1.019187 1.798279 0.000476 H 7 C 2 0.285293 -1.020606 0.019761 -0.298318 (3 -1)
XX 1.018091 1.799042 0.000322 H 8 C 3 0.285268 -1.020447 0.019759 -0.298273 (3 -1)
XX 1.019190 -1.798279 -0.000579 H 9 C 5 0.285293 -1.020607 0.019761 -0.298319 (3 -1)
XX -1.018086 -1.799042 -0.000414 H 10 C 6 0.285269 -1.020453 0.019759 -0.298275 (3 -1)
XX -2.175721 -0.000364 0.000108 C 11 C 1 0.279977 -0.693317 0.153377 -0.250322 (3 -1)
XX -3.280164 -0.653292 0.000189 C 12 C 11 0.257079 -0.602370 0.068892 -0.213833 (3 -1)
XX -3.279290 0.653410 -0.000086 C 13 C 11 0.257053 -0.602240 0.068889 -0.213791 (3 -1)

XX 2.175714 0.000364 -0.000114 C 14 C   4 0.279988 -0.693371 0.153385 -0.250342 (3 -1)
XX 3.280158 0.653296 -0.000179 C 15 C 14 0.257071 -0.602336 0.068888 -0.213821 (3 -1)
XX 3.279283 -0.653414 0.000028 C 16 C 14 0.257046 -0.602207 0.068886 -0.213780 (3 -1)
XX -4.326269 1.188323 -0.000295 H 17 C 13 0.277770 -0.954382 0.008286 -0.283925 (3 -1)
XX -3.502897 1.681340 0.547798 H 18 C 13 0.270639 -0.901028 0.010055 -0.270984 (3 -1)
XX -3.502813 1.681022 -0.548593 H 19 C 13 0.270637 -0.901017 0.010056 -0.270979 (3 -1)
XX -4.327872 -1.186709 0.000712 H 20 C 12 0.277775 -0.954448 0.008297 -0.283937 (3 -1)
XX -3.505443 -1.680558 -0.548133 H 21 C 12 0.270616 -0.900880 0.010064 -0.270943 (3 -1)
XX -3.504706 -1.680868 0.548241 H 22 C 12 0.270629 -0.900975 0.010065 -0.270963 (3 -1)
XX 4.327873 1.186700 -0.000943 H 23 C 15 0.277777 -0.954460 0.008296 -0.283940 (3 -1)
XX 3.504495 1.681107 -0.547889 H 24 C 15 0.270639 -0.901045 0.010063 -0.270981 (3 -1)
XX 3.505669 1.680324 0.548487 H 25 C 15 0.270609 -0.900831 0.010064 -0.270930 (3 -1)
XX 4.326268 -1.188319 -0.000002 H 26 C 16 0.277772 -0.954395 0.008285 -0.283929 (3 -1)
XX 3.503046 -1.680870 0.548740 H 27 C 16 0.270635 -0.901001 0.010055 -0.270976 (3 -1)
XX 3.502665 -1.681494 -0.547652 H 28 C 16 0.270645 -0.901075 0.010054 -0.270995 (3 -1)

M3c Singlet state
XX 1.081952 0.612141 -0.000818 C   2 C   1 0.277525 -0.699638 0.100374 -0.246508 (3 -1)
XX -0.000001 -0.000003 -0.000118 RING   0.018818 0.143739 -1.211874 0.008104 (3 1)
XX -0.000027 1.213574 -0.001446 C   3 C   2 0.335554 -0.954698 0.313562 -0.362142 (3 -1)
XX -1.081966 0.611997 -0.000604 C   4 C   3 0.277510 -0.699557 0.100368 -0.246479 (3 -1)
XX -1.081955 -0.612148 0.000658 C   5 C   4 0.277527 -0.699646 0.100373 -0.246510 (3 -1)
XX 1.081963 -0.612005 0.000353 C   6 C   1 0.277512 -0.699567 0.100369 -0.246482 (3 -1)
XX 0.000025 -1.213579 0.001203 C   6 C   5 0.335554 -0.954694 0.313561 -0.362140 (3 -1)
XX 2.159904 1.492189 0.003959 RING   0.011597 0.063974 -1.655418 0.004040 (3 1)
XX 0.994955 1.820836 -0.003072 H   7 C   2 0.286114 -1.025651 0.018615 -0.300038 (3 -1)
XX -0.995240 1.820625 -0.002603 H   8 C   3 0.286125 -1.025748 0.018608 -0.300061 (3 -1)
XX -2.160222 1.492336 0.002688 RING   0.011592 0.063929 -1.656059 0.004038 (3 1)
XX -2.159902 -1.492168 -0.002484 RING   0.011597 0.063978 -1.655335 0.004041 (3 1)
XX -0.994950 -1.820843 0.002775 H   9 C   5 0.286115 -1.025656 0.018615 -0.300039 (3 -1)

XX 2.160244 -1.492406 -0.004485 RING 0.011591 0.063919 -1.656247 0.004037 (3 1)
XX 0.995241 -1.820632 0.002353 H 10 C 6 0.286124 -1.025744 0.018608 -0.300059 (3 -1)
XX 2.161077 0.000061 -0.000166 C 11 C 1 0.322416 -0.868540 0.318323 -0.333002 (3 -1)
XX 2.154534 -2.020236 -0.007278 C 12 H 10 0.013737 0.053715 0.414973 0.002504 (3 -1)
XX 3.269773 -0.634567 -0.000954 C 12 C 11 0.254501 -0.598238 0.045852 -0.209719 (3 -1)
XX 2.153973 2.020317 0.006401 C 13 H 7 0.013750 0.053755 0.414219 0.002503 (3 -1)
XX 3.269756 0.634630 0.001001 C 13 C 11 0.254536 -0.598400 0.045844 -0.209775 (3 -1)
XX -2.161081 -0.000067 0.000078 C 14 C 4 0.322417 -0.868544 0.318323 -0.333004 (3 -1)
XX -2.154495 2.020303 0.004504 C 15 H 8 0.013741 0.053723 0.414792 0.002503 (3 -1)
XX -3.269767 0.634569 0.000526 C 15 C 14 0.254504 -0.598252 0.045852 -0.209724 (3 -1)
XX -2.153963 -2.020365 -0.004165 C 16 H 9 0.013751 0.053757 0.414141 0.002503 (3 -1)
XX -3.269763 -0.634628 -0.000368 C 16 C 14 0.254537 -0.598404 0.045845 -0.209776 (3 -1)
XX 4.110417 1.250803 0.544717 H 17 C 13 0.272180 -0.911928 0.008796 -0.273967 (3 -1)
XX 4.097236 1.262397 -0.550391 H 18 C 13 0.272049 -0.911023 0.008773 -0.273740 (3 -1)
XX 3.334724 1.827819 0.012651 H 19 C 13 0.280264 -0.972096 0.007415 -0.288729 (3 -1)
XX 4.111452 -1.250224 -0.543736 H 20 C 12 0.272171 -0.911856 0.008814 -0.273944 (3 -1)
XX 4.096474 -1.262562 0.551412 H 21 C 12 0.272020 -0.910813 0.008783 -0.273683 (3 -1)
XX 3.335343 -1.828049 -0.013301 H 22 C 12 0.280227 -0.971821 0.007454 -0.288651 (3 -1)
XX -4.098526 1.260724 -0.550751 H 23 C 15 0.272039 -0.910939 0.008788 -0.273715 (3 -1)
XX -4.109402 1.252060 0.544481 H 24 C 15 0.272152 -0.911727 0.008810 -0.273913 (3 -1)
XX -3.335281 1.828081 0.009144 H 25 C 15 0.280227 -0.971817 0.007454 -0.288650 (3 -1)
XX -4.098620 -1.260776 0.550687 H 26 C 16 0.272059 -0.911091 0.008774 -0.273756 (3 -1)
XX -4.109082 -1.252370 -0.544476 H 27 C 16 0.272169 -0.911854 0.008794 -0.273949 (3 -1)
XX -3.334717 -1.827841 -0.008767 H 28 C 16 0.280267 -0.972116 0.007414 -0.288735 (3 -1)

M3d Triplet state
XX -0.597908 1.073662 -0.045697 C 2 C 1 0.296659 -0.755546 0.205519 -0.282453 (3 -1)
XX 0.000124 0.000674 0.001137 RING 0.019312 0.147785 -1.178735 0.008310 (3 1)
XX -1.221923 -0.002489 0.000913 C 3 C 2 0.302044 -0.778184 0.223033 -0.291727 (3 -1)
XX -0.592811 -1.075489 0.050433 C 4 C 3 0.296840 -0.756385 0.206007 -0.282815 (3 -1)

XX 0.598145 -1.072283 -0.046003 C 5 C 4 0.296778 -0.756248 0.205308 -0.282669 (3 -1)
XX 0.593316 1.076619 0.049544 C 6 C 1 0.296730 -0.755976 0.205628 -0.282591 (3 -1)
XX 1.222264 0.003642 -0.000221 C 6 C 5 0.302219 -0.779127 0.223117 -0.292052 (3 -1)
XX -1.152377 2.185827 -0.583667 RING 0.015257 0.082175 -1.536518 0.003398 (3 1)
XX -1.817558 1.047049 -0.253792 C 7 C 2 0.258461 -0.647077 0.040481 -0.216476 (3 -1)
XX -2.685045 0.222833 0.257883 RING 0.010786 0.063772 -1.261777 0.003033 (3 1)
XX -1.813445 -1.055452 0.252141 C 8 C 3 0.258523 -0.647467 0.040178 -0.216559 (3 -1)
XX -1.144124 -2.188117 0.586964 RING 0.015278 0.082476 -1.531035 0.003417 (3 1)
XX -2.667780 -0.004353 -0.001056 CAGE 0.010619 0.067871 -0.915823 0.003456 (3 3)
XX 1.152026 -2.185964 -0.587392 RING 0.015226 0.081785 -1.538948 0.003378 (3 1)
XX 1.817144 -1.045881 -0.257193 C 9 C 5 0.258475 -0.647180 0.040408 -0.216489 (3 -1)
XX 0.007136 -2.198650 0.007800 C 9 C 8 0.269536 -0.648630 0.101396 -0.235626 (3 -1)
XX 1.144229 2.191135 0.592699 RING 0.015175 0.081532 -1.539525 0.003373 (3 1)
XX 2.683783 -0.227587 0.258976 RING 0.010858 0.064082 -1.279810 0.003034 (3 1)
XX 1.813775 1.055327 0.254034 C 10 C 6 0.258537 -0.647503 0.040299 -0.216593 (3 -1)
XX -0.005288 2.199802 0.004868 C 10 C 7 0.269621 -0.648907 0.102129 -0.235741 (3 -1)
XX 2.665638 0.011847 -0.011252 CAGE 0.010664 0.068245 -0.915808 0.003469 (3 3)
XX -1.140708 2.638899 -0.671495 C 12 C 7 0.017314 0.065531 0.212181 0.000780 (3 -1)
XX 0.346639 3.306270 -0.544750 C 12 C 11 0.256132 -0.597029 0.074306 -0.213844 (3 -1)
XX 1.131021 2.639118 0.683101 C 13 C 10 0.017164 0.064941 0.216143 0.000794 (3 -1)
XX -0.366217 3.301339 0.558340 C 13 C 11 0.256221 -0.597463 0.074261 -0.213977 (3 -1)
XX 1.140660 -2.633488 -0.675706 C 15 C 9 0.017206 0.065113 0.216578 0.000792 (3 -1)
XX -0.341077 -3.307699 -0.538964 C 15 C 14 0.256204 -0.597340 0.074333 -0.213955 (3 -1)
XX -1.129686 -2.644816 0.678240 C 16 C 8 0.017407 0.065812 0.205208 0.000766 (3 -1)
XX 0.366177 -3.296795 0.568054 C 16 C 14 0.256105 -0.596818 0.074636 -0.213805 (3 -1)
XX -1.684809 2.529291 0.436645 RING 0.006379 0.030890 -1.306434 0.001611 (3 1)
XX -0.242790 4.108097 1.386394 H 17 C 13 0.271885 -0.914776 0.009873 -0.273574 (3 -1)
XX -1.195432 4.085764 0.830497 H 18 C 13 0.272584 -0.919745 0.010869 -0.274591 (3 -1)
XX -0.967155 3.285247 1.570075 H 19 C 13 0.279722 -0.973559 0.009533 -0.287414 (3 -1)
XX 1.664517 2.535783 -0.429920 RING 0.006396 0.030928 -1.312719 0.001610 (3 1)

XX 1.165625 4.101604 -0.816905 H 20 C 12 0.272611 -0.919980 0.010926 -0.274644 (3 -1)
XX 0.947071 3.298905 -1.557145 H 21 C 12 0.279782 -0.974075 0.009542 -0.287534 (3 -1)
XX 0.212309 4.111952 -1.371824 H 22 C 12 0.271864 -0.914558 0.009852 -0.273539 (3 -1)
XX -1.665945 -2.541456 -0.434634 RING 0.006356 0.030790 -1.304275 0.001607 (3 1)
XX -1.157958 -4.105398 -0.809958 H 23 C 15 0.272615 -0.919978 0.010874 -0.274644 (3 -1)
XX -0.939453 -3.304481 -1.552159 H 24 C 15 0.279776 -0.973977 0.009510 -0.287528 (3 -1)
XX -2.682291 -0.238922 -0.268059 RING 0.010800 0.063781 -1.271412 0.003029 (3 1)
XX -0.203519 -4.115666 -1.363282 H 25 C 15 0.271890 -0.914786 0.009798 -0.273591 (3 -1)
XX 1.678118 -2.520708 0.440513 RING 0.006417 0.030915 -1.329508 0.001602 (3 1)
XX 1.188166 -4.085898 0.849961 H 26 C 16 0.272623 -0.920069 0.010908 -0.274654 (3 -1)
XX 2.680559 0.234708 -0.262531 RING 0.010823 0.064087 -1.255012 0.003046 (3 1)
XX 0.967243 -3.274243 1.579646 H 27 C 16 0.279761 -0.973859 0.009505 -0.287495 (3 -1)
XX 0.235244 -4.092327 1.405711 H 28 C 16 0.271795 -0.914076 0.009811 -0.273415 (3 -1)
XX -2.790415 1.170377 -0.688531 F 29 C 7 0.275022 -0.342972 0.116764 -0.409942 (3 -1)
XX -3.066825 -0.544454 -0.471207 RING 0.010855 0.061203 -1.932310 0.002309 (3 1)
XX -2.720134 1.627159 -0.109385 F 30 C 7 0.273643 -0.365579 0.117401 -0.405990 (3 -1)
XX -1.976025 2.712012 1.023471 F 30 C 13 0.007951 0.035716 0.805965 0.001696 (3 -1)
XX -2.404998 1.790269 -0.759219 F 31 C 7 0.270295 -0.356210 0.118295 -0.399097 (3 -1)
XX 3.073984 -0.538292 0.466572 RING 0.010862 0.061079 -1.981873 0.002290 (3 1)
XX 2.788629 1.181157 0.683411 F 32 C 10 0.274973 -0.343296 0.116741 -0.409830 (3 -1)
XX 2.711491 1.642495 0.108670 F 33 C 10 0.273672 -0.365112 0.117446 -0.406062 (3 -1)
XX 1.957717 2.726363 -1.023597 F 33 C 12 0.008036 0.036104 0.789196 0.001709 (3 -1)
XX 2.399680 1.797729 0.761941 F 34 C 10 0.270396 -0.355725 0.118350 -0.399332 (3 -1)
XX 2.789713 -1.168575 -0.692636 F 35 C 9 0.275032 -0.342648 0.116729 -0.409968 (3 -1)
XX 3.333492 0.006910 -0.006729 F 35 F 32 0.015559 0.069844 0.030203 0.000651 (3 -1)
XX 3.187445 0.660133 -0.779458 F 35 F 33 0.011265 0.053533 0.604691 0.001056 (3 -1)
XX 2.718275 -1.629230 -0.116740 F 36 C 9 0.273664 -0.365243 0.117452 -0.406040 (3 -1)
XX 1.973712 -2.709700 1.025211 F 36 C 16 0.008013 0.036021 0.803164 0.001705 (3 -1)
XX 3.195737 -0.645551 0.768794 F 36 F 32 0.011346 0.053810 0.589911 0.001044 (3 -1)
XX 2.402662 -1.786879 -0.767790 F 37 C 9 0.270274 -0.356975 0.118281 -0.399037 (3 -1)

XX -2.399855 -1.802214 0.753396 F 38 C 8 0.270241 -0.356714 0.118443 -0.398962 (3 -1)
XX -3.075079 0.527638 0.458881 RING 0.010849 0.061189 -1.927272 0.002309 (3 1)
XX -2.788992 -1.185029 0.679291 F 39 C 8 0.275130 -0.342133 0.116770 -0.410184 (3 -1)
XX -3.333232 -0.009235 -0.006970 F 39 F 29 0.015412 0.069284 0.031705 0.000665 (3 -1)
XX -3.199779 0.638893 0.776360 F 39 F 30 0.011403 0.053852 0.539216 0.001016 (3 -1)
XX -2.710616 -1.641316 0.100739 F 40 C 8 0.273592 -0.365990 0.117641 -0.405873 (3 -1)
XX -1.953060 -2.722510 -1.031559 F 40 C 15 0.007958 0.035748 0.766025 0.001702 (3 -1)
XX -3.188357 -0.656332 -0.788875 F 40 F 29 0.011405 0.053869 0.543074 0.001018 (3 -1)

M3d Singlet state
XX -0.608184 1.003188 0.303786 C 2 C 1 0.255916 -0.591894 0.077784 -0.212709 (3 -1)
XX 0.000143 -0.004150 0.296734 RING 0.022708 0.159647 -1.112517 0.007987 (3 1)
XX -1.238968 -0.021742 0.024193 C 3 C 2 0.332969 -0.908498 0.369980 -0.358208 (3 -1)
XX -0.602369 -1.016872 0.371512 C 4 C 3 0.257407 -0.599931 0.068365 -0.215262 (3 -1)
XX 0.605144 -1.015725 0.372914 C 5 C 4 0.257307 -0.599428 0.068362 -0.215110 (3 -1)
XX 0.605862 1.004469 0.304435 C 6 C 1 0.255920 -0.591975 0.077500 -0.212711 (3 -1)
XX 1.239704 -0.019264 0.026304 C 6 C 5 0.332965 -0.908496 0.369933 -0.358188 (3 -1)
XX -1.734359 1.050491 -0.300093 C 7 C 2 0.261054 -0.659685 0.036719 -0.221050 (3 -1)
XX -3.092542 0.200593 -0.622619 RING 0.007645 0.044910 -1.503701 0.002140 (3 1)
XX -1.696285 -1.121686 -0.273366 C 8 C 3 0.262589 -0.666681 0.035496 -0.223308 (3 -1)
XX 1.701222 -1.117369 -0.269686 C 9 C 5 0.262639 -0.666938 0.035635 -0.223390 (3 -1)
XX 3.088129 0.208741 -0.626104 RING 0.007674 0.045100 -1.499269 0.002148 (3 1)
XX 1.731561 1.054332 -0.299793 C 10 C 6 0.260940 -0.659094 0.036634 -0.220866 (3 -1)
XX -0.002350 1.807536 1.039103 C 11 C 1 0.331434 -0.909537 0.368136 -0.353887 (3 -1)
XX -1.865710 2.284488 0.633082 RING 0.007039 0.035421 -1.253432 0.002050 (3 1)
XX -0.653471 2.572828 1.823607 C 12 C 11 0.255623 -0.608733 0.031459 -0.212453 (3 -1)
XX 1.662602 2.268311 0.613776 CAGE 0.007068 0.035368 -0.738011 0.001965 (3 3)
XX 0.646088 2.574889 1.824132 C 13 C 11 0.255680 -0.608967 0.031647 -0.212541 (3 -1)
XX 0.000557 -1.688081 1.227139 C 14 C 4 0.331492 -0.906071 0.379628 -0.354626 (3 -1)
XX -1.291855 -2.434113 0.882188 RING 0.008633 0.041920 -1.531387 0.001922 (3 1)

XX 1.295917 -2.431582 0.884491 RING 0.008591 0.041726 -1.530734 0.001914 (3 1)
XX -0.650983 -2.284257 2.143827 C 15 C 14 0.255307 -0.607523 0.032193 -0.211860 (3 -1)
XX 0.649798 -2.285102 2.144832 C 16 C 14 0.255290 -0.607472 0.032009 -0.211839 (3 -1)
XX 1.416724 2.639096 0.564860 RING 0.007451 0.035051 -2.619744 0.001623 (3 1)
XX 1.611459 2.682145 0.605748 H 17 C 10 0.007496 0.034839 1.287129 0.001623 (3 -1)
XX 1.313678 3.520126 1.941132 H 17 C 13 0.273333 -0.928417 0.009398 -0.276668 (3 -1)
XX 1.205032 2.841911 2.802510 H 18 C 13 0.274920 -0.935085 0.011773 -0.279217 (3 -1)
XX 2.071792 1.793604 0.846456 RING 0.008127 0.038371 -1.673445 0.002286 (3 1)
XX 1.823838 2.531009 1.935296 H 19 C 13 0.282767 -0.997539 0.009361 -0.293472 (3 -1)
XX -2.080087 1.780408 0.853999 RING 0.008089 0.038176 -1.657658 0.002272 (3 1)
XX -1.592730 2.690040 0.603742 H 20 C 7 0.007489 0.034749 1.306118 0.001596 (3 -1)
XX -1.332674 3.511606 1.926946 H 20 C 12 0.273305 -0.928294 0.009351 -0.276629 (3 -1)
XX -2.654221 2.009882 1.063506 H 21 C 7 0.010889 0.043386 0.110942 0.001123 (3 -1)
XX -1.829420 2.515816 1.944402 H 21 C 12 0.282623 -0.996396 0.009473 -0.293197 (3 -1)
XX -1.208960 2.852135 2.800936 H 22 C 12 0.275055 -0.936018 0.011868 -0.279434 (3 -1)
XX -1.210949 -2.342274 3.156331 H 23 C 15 0.275087 -0.936205 0.011639 -0.279410 (3 -1)
XX -1.825846 -2.203433 2.242570 H 24 C 15 0.281384 -0.985712 0.008973 -0.290648 (3 -1)
XX -1.334170 -3.178713 2.448584 H 25 C 15 0.273853 -0.932684 0.008596 -0.277570 (3 -1)
XX 1.339549 -3.178845 2.436277 H 26 C 16 0.273856 -0.932801 0.008462 -0.277593 (3 -1)
XX 1.822553 -2.194754 2.253448 H 27 C 16 0.281259 -0.984725 0.009052 -0.290427 (3 -1)
XX 1.203241 -2.358168 3.160255 H 28 C 16 0.275282 -0.937614 0.011685 -0.279752 (3 -1)
XX -2.157932 1.932020 -0.730324 F 29 C 7 0.273236 -0.340453 0.118543 -0.405936 (3 -1)
XX -2.257449 -0.236739 -1.234433 RING 0.007920 0.046085 -1.094776 0.002390 (3 1)
XX -1.703193 2.260744 0.621111 CAGE 0.007025 0.035143 -0.807873 0.001971 (3 3)
XX -2.403865 1.337518 -1.088827 F 30 C 7 0.275500 -0.350322 0.122135 -0.410660 (3 -1)
XX -2.694076 1.518742 -0.430371 F 31 C 7 0.269695 -0.374828 0.120588 -0.396916 (3 -1)
XX 2.689665 1.525894 -0.432956 F 32 C 10 0.269717 -0.374815 0.120413 -0.396964 (3 -1)
XX 2.650664 2.025701 1.054264 F 32 C 13 0.010966 0.043558 0.113652 0.001104 (3 -1)
XX 2.238807 -0.231395 -1.241331 RING 0.007939 0.046057 -1.155748 0.002381 (3 1)
XX 2.398296 1.342617 -1.090282 F 33 C 10 0.275528 -0.350023 0.122181 -0.410730 (3 -1)

XX 2.151227 1.936716 -0.732037 F 34 C 10 0.273268 -0.340299 0.118508 -0.406010 (3 -1)
XX 2.132688 -1.614896 -1.109647 F 35 C 9 0.272980 -0.376020 0.124525 -0.404387 (3 -1)
XX 2.272949 -0.308722 -1.883234 F 35 F 33 0.009954 0.046092 0.151567 0.000885 (3 -1)
XX 2.659395 -1.459117 -0.608108 F 36 C 9 0.274032 -0.346379 0.120816 -0.407337 (3 -1)
XX 1.938022 -2.520843 1.106397 F 36 H 26 0.010824 0.049640 0.779216 0.002039 (3 -1)
XX 3.418492 0.189135 -0.256838 F 36 F 32 0.008848 0.042205 0.195638 0.001030 (3 -1)
XX 3.012149 -0.080768 -1.180223 F 36 F 33 0.009948 0.047454 0.043842 0.001068 (3 -1)
XX 2.205369 -2.030572 -0.504583 F 37 C 9 0.272395 -0.340439 0.116261 -0.404021 (3 -1)
XX -2.652274 -1.467720 -0.614239 F 38 C 8 0.274139 -0.345465 0.120778 -0.407580 (3 -1)
XX -3.013404 -0.089345 -1.184027 F 38 F 30 0.009996 0.047589 0.042666 0.001057 (3 -1)
XX -3.416492 0.177762 -0.259105 F 38 F 31 0.008760 0.041909 0.202891 0.001043 (3 -1)
XX -2.123398 -1.620475 -1.114381 F 39 C 8 0.272961 -0.375962 0.124762 -0.404348 (3 -1)
XX -2.270287 -0.313162 -1.883758 F 39 F 30 0.009867 0.045804 0.157686 0.000899 (3 -1)
XX -2.195929 -2.037091 -0.509891 F 40 C 8 0.272361 -0.340947 0.116228 -0.403940 (3 -1)
XX -1.946226 -2.518676 1.097913 F 40 C 15 0.010964 0.050177 0.724348 0.002045 (3 -1)

M3e Triplet state
XX -1.093806 0.592405 0.006655 C 2 C 1 0.296669 -0.768898 0.196296 -0.282295 (3 -1)
XX 0.000209 0.000153 0.000098 RING 0.018698 0.142250 -1.201251 0.008078 (3 1)
XX 0.000426 1.206398 0.000180 C 3 C 2 0.300344 -0.782476 0.228665 -0.288319 (3 -1)
XX 1.094433 0.591992 -0.006146 C 4 C 3 0.296610 -0.768619 0.196188 -0.282183 (3 -1)
XX 1.094161 -0.592143 0.006269 C 5 C 4 0.296577 -0.768448 0.196165 -0.282122 (3 -1)
XX -1.094052 -0.591642 -0.005977 C 6 C 1 0.296659 -0.768859 0.196230 -0.282277 (3 -1)
XX -0.000118 -1.206112 -0.000015 C 6 C 5 0.300378 -0.782652 0.228689 -0.288385 (3 -1)
XX -1.021653 1.795192 0.070211 C 7 C 2 0.283049 -0.763039 0.075048 -0.270225 (3 -1)
XX 1.022524 1.794873 -0.070350 C 8 C 3 0.283057 -0.763059 0.075112 -0.270245 (3 -1)
XX 1.021714 -1.795065 0.070296 C 9 C 5 0.283044 -0.762983 0.075080 -0.270215 (3 -1)
XX -1.022391 -1.794409 -0.070692 C 10 C 6 0.283049 -0.763041 0.075063 -0.270227 (3 -1)
XX -2.217519 0.000562 0.000749 C 11 C 1 0.277584 -0.685681 0.131355 -0.248690 (3 -1)
XX -3.320147 -0.471985 0.461703 C 12 C 11 0.256396 -0.601625 0.064792 -0.214609 (3 -1)

XX -3.320602 0.472619 -0.459896 C 13 C 11 0.256421 -0.601751 0.064795 -0.214654 (3 -1)
XX 2.217839 -0.000415 0.000246 C 14 C 4 0.277652 -0.685939 0.131692 -0.248801 (3 -1)
XX 3.320762 0.472724 0.460009 C 15 C 14 0.256457 -0.601944 0.064644 -0.214708 (3 -1)
XX 3.320632 -0.474041 -0.459374 C 16 C 14 0.256390 -0.601622 0.064716 -0.214600 (3 -1)
XX -4.195749 0.535027 -1.213463 H 17 C 13 0.272974 -0.924650 0.011455 -0.275757 (3 -1)
XX -4.037811 1.397664 -0.540369 H 18 C 13 0.272703 -0.926356 0.010507 -0.275359 (3 -1)
XX -3.324504 1.210378 -1.374565 H 19 C 13 0.279563 -0.973341 0.010209 -0.287206 (3 -1)
XX -4.036007 -1.398058 0.541609 H 20 C 12 0.272704 -0.926361 0.010483 -0.275360 (3 -1)
XX -3.323598 -1.209989 1.376344 H 21 C 12 0.279554 -0.973256 0.010217 -0.287189 (3 -1)
XX -4.195537 -0.535727 1.214953 H 22 C 12 0.272972 -0.924638 0.011440 -0.275747 (3 -1)
XX 4.033354 1.401554 0.538411 H 23 C 15 0.272687 -0.926297 0.010466 -0.275341 (3 -1)
XX 3.324937 1.209028 1.375528 H 24 C 15 0.279547 -0.973178 0.010218 -0.287183 (3 -1)
XX 4.199100 0.538688 1.209584 H 25 C 15 0.273032 -0.925085 0.011458 -0.275852 (3 -1)
XX 4.036439 -1.400212 -0.537425 H 26 C 16 0.272708 -0.926414 0.010478 -0.275372 (3 -1)
XX 3.324380 -1.213390 -1.372820 H 27 C 16 0.279598 -0.973595 0.010200 -0.287278 (3 -1)
XX 4.196397 -0.539097 -1.212201 H 28 C 16 0.272958 -0.924550 0.011445 -0.275720 (3 -1)
XX 1.562422 -2.813914 0.183563 N 29 C 9 0.463504 0.276361 0.035545 -0.827516 (3 -1)
XX -1.564036 -2.812682 -0.184675 N 30 C 10 0.463508 0.276410 0.035520 -0.827522 (3 -1)
XX 1.563855 2.813374 -0.183772 N 31 C 8 0.463501 0.276256 0.035556 -0.827512 (3 -1)
XX -1.562999 2.813789 0.182732 N 32 C 7 0.463509 0.276393 0.035525 -0.827525 (3 -1)

M3e Singlet state
XX -1.085197 -0.560680 -0.041859 C 2 C 1 0.264320 -0.635035 0.106151 -0.226506 (3 -1)
XX 0.000104 0.000135 -0.214988 RING 0.019567 0.142399 -1.173482 0.007773 (3 1)
XX -0.040118 -1.222395 -0.263469 C 3 C 2 0.325425 -0.890749 0.333972 -0.339744 (3 -1)
XX 1.047626 -0.634939 -0.042129 C 4 C 3 0.264258 -0.634945 0.105412 -0.226395 (3 -1)
XX 1.085426 0.560854 -0.041718 C 5 C 4 0.264248 -0.634684 0.106166 -0.226390 (3 -1)
XX -1.047469 0.635135 -0.042262 C 6 C 1 0.264273 -0.635010 0.105463 -0.226418 (3 -1)
XX 0.040300 1.222620 -0.263414 C 6 C 5 0.325371 -0.890426 0.333974 -0.339631 (3 -1)
XX -1.078374 -1.713136 -0.496364 C 7 C 2 0.283197 -0.763235 0.071917 -0.271244 (3 -1)

XX 0.958615 -1.786146 -0.499065 C 8 C 3 0.283042 -0.762715 0.071387 -0.270908 (3 -1)
XX 1.078448 1.713561 -0.496172 C 9 C 5 0.283175 -0.763095 0.071943 -0.271197 (3 -1)
XX -0.958640 1.786416 -0.498734 C 10 C 6 0.283049 -0.762756 0.071386 -0.270918 (3 -1)
XX -2.018695 0.061223 0.490469 C 11 C 1 0.326390 -0.888142 0.330785 -0.342244 (3 -1)
XX -2.095360 -1.548970 0.405933 RING 0.010212 0.050773 -1.504925 0.003119 (3 1)
XX -1.996616 1.607289 0.380990 RING 0.010237 0.053201 -1.446923 0.003347 (3 1)
XX -2.948230 0.728601 1.067738 C 12 C 11 0.256409 -0.611760 0.035224 -0.214396 (3 -1)
XX -2.255199 -1.973927 0.381490 C 13 C 7 0.011014 0.045916 0.088627 0.002639 (3 -1)
XX -2.974156 -0.579152 1.048544 C 13 C 11 0.254237 -0.602633 0.029521 -0.210963 (3 -1)
XX 1.996461 -1.606721 0.380292 RING 0.010250 0.053300 -1.446893 0.003353 (3 1)
XX 2.018972 -0.061329 0.490603 C 14 C 4 0.326406 -0.888240 0.330806 -0.342278 (3 -1)
XX 2.095527 1.548329 0.405883 RING 0.010209 0.050779 -1.504019 0.003121 (3 1)
XX 2.948030 -0.729200 1.067979 C 15 C 14 0.256363 -0.611537 0.035221 -0.214322 (3 -1)
XX 2.255826 1.975740 0.381325 C 16 C 9 0.011024 0.045932 0.089955 0.002639 (3 -1)
XX 2.975021 0.578561 1.048002 C 16 C 14 0.254320 -0.603022 0.029556 -0.211095 (3 -1)
XX -3.691843 -1.058450 1.819823 H 17 C 13 0.278020 -0.959226 0.013204 -0.284511 (3 -1)
XX -3.780639 -1.370547 0.759876 H 18 C 13 0.273249 -0.932589 0.010061 -0.276929 (3 -1)
XX -2.915267 -1.712592 1.364695 H 19 C 13 0.280196 -0.978624 0.012417 -0.288852 (3 -1)
XX -3.378391 1.295768 1.971839 H 20 C 12 0.274253 -0.932725 0.012384 -0.278343 (3 -1)
XX -2.945895 1.910458 1.149039 H 21 C 12 0.284446 -1.014015 0.009658 -0.296936 (3 -1)
XX -3.910608 1.369249 1.009952 H 22 C 12 0.272970 -0.927886 0.010843 -0.276242 (3 -1)
XX 3.374436 -1.298209 1.972981 H 23 C 15 0.274156 -0.932062 0.012375 -0.278163 (3 -1)
XX 2.946268 -1.911244 1.146530 H 24 C 15 0.284458 -1.014131 0.009642 -0.296956 (3 -1)
XX 3.911258 -1.368875 1.013533 H 25 C 15 0.273002 -0.928068 0.010872 -0.276291 (3 -1)
XX 3.782520 1.368254 0.758714 H 26 C 16 0.273261 -0.932657 0.010011 -0.276962 (3 -1)
XX 2.917361 1.712452 1.362687 H 27 C 16 0.280237 -0.978963 0.012372 -0.288933 (3 -1)
XX 3.692839 1.057724 1.818950 H 28 C 16 0.278048 -0.959440 0.013171 -0.284580 (3 -1)
XX 1.695406 2.595716 -0.925149 N 29 C 9 0.463006 0.268133 0.032975 -0.826704 (3 -1)
XX -1.500619 2.713556 -0.934454 N 30 C 10 0.462980 0.266430 0.032479 -0.826732 (3 -1)
XX -2.188086 2.268591 0.273745 N 30 C 12 0.013660 0.051035 0.249115 0.002186 (3 -1)

XX 1.500185 -2.713306 -0.935296 N 31 C 8 0.462979 0.266383 0.032466 -0.826731 (3 -1)
XX 2.188490 -2.269504 0.271580 N 31 C 15 0.013709 0.051158 0.249676 0.002179 (3 -1)
XX -1.695675 -2.594866 -0.925692 N 32 C 7 0.463010 0.268166 0.032962 -0.826712 (3 -1)

M4a Triplet state
XX -1.073765 -0.511510 0.236881 C 2 C 1 0.306853 -0.817097 0.276369 -0.300779 (3 -1)
XX -0.002974 0.025254 -0.023462 RING 0.018110 0.139652 -1.171425 0.008121 (3 1)
XX -0.059025 -1.072309 0.546678 C 3 C 2 0.310571 -0.836472 0.291561 -0.307871 (3 -1)
XX 1.052253 -0.534377 0.273216 C 4 C 3 0.286684 -0.720261 0.210531 -0.263259 (3 -1)
XX 1.072322 0.546132 -0.292881 C 5 C 4 0.288656 -0.730916 0.211417 -0.266782 (3 -1)
XX -1.066135 0.604796 -0.303071 C 6 C 1 0.310809 -0.827188 0.291572 -0.308959 (3 -1)
XX -0.021366 1.122472 -0.587430 C 6 C 5 0.305855 -0.821702 0.261316 -0.297822 (3 -1)
XX -1.045969 -1.470252 0.723629 N 7 C 2 0.286154 -0.875099 0.070960 -0.363758 (3 -1)
XX 0.888581 -1.451716 0.820756 N 8 C 3 0.310694 -0.991041 0.097703 -0.460602 (3 -1)
XX 0.935267 1.457043 -0.848567 N 9 C 5 0.318309 -1.005018 0.104101 -0.486074 (3 -1)
XX -0.008517 2.173396 -1.376091 RING 0.019137 0.111619 -1.819786 0.005635 (3 1)
XX -2.120864 0.041268 -0.031545 C 10 C 1 0.263299 -0.632063 0.021347 -0.226118 (3 -1)
XX -3.264498 -0.328395 -0.517884 C 11 C 10 0.270459 -0.687999 0.077921 -0.234765 (3 -1)
XX -2.678900 -1.063612 1.600264 RING 0.006063 0.027350 -7.704763 0.001204 (3 1)
XX -3.252141 0.341502 0.497089 C 12 C 10 0.271014 -0.690895 0.079022 -0.237015 (3 -1)
XX 2.209841 -1.648711 0.083334 CAGE 0.007539 0.038712 -0.799220 0.001877 (3 3)
XX 2.124677 -0.003925 -0.001920 C 13 C 4 0.279842 -0.674360 0.178357 -0.253057 (3 -1)
XX 2.276995 1.659104 -0.110282 CAGE 0.007578 0.037830 -0.857197 0.001808 (3 3)
XX 2.957058 -2.003799 1.099285 C 14 C 2 0.016748 0.061816 0.036301 0.000112 (3 -1)
XX 1.990488 -1.690081 -0.132055 RING 0.007653 0.037826 -1.419983 0.001649 (3 1)
XX 2.429258 -1.875351 0.227332 RING 0.007684 0.038946 -1.417339 0.001971 (3 1)
XX 3.240136 -0.570341 -0.187003 C 14 C 13 0.268335 -0.674605 0.107915 -0.234713 (3 -1)
XX 2.029344 1.690312 0.125705 RING 0.007701 0.037617 -1.465900 0.001623 (3 1)
XX 3.002079 1.972186 -1.116239 C 15 C 6 0.015816 0.060236 0.060978 0.000386 (3 -1)
XX 3.247156 0.543851 0.199015 C 15 C 13 0.268045 -0.673221 0.107352 -0.234123 (3 -1)

XX -4.082128 0.787921 0.984996 F 16 C 12 0.276416 -0.339854 0.117943 -0.412772 (3 -1)
XX -2.671979 -1.094001 1.691906 F 17 N 7 0.006067 0.028227 6.553995 0.001308 (3 -1)
XX -3.650764 0.432733 1.477934 F 17 C 12 0.266739 -0.395013 0.125368 -0.388812 (3 -1)
XX -3.449177 1.098650 1.211140 F 18 C 12 0.271562 -0.360848 0.117756 -0.401597 (3 -1)
XX -3.874519 -0.413244 -1.374044 F 19 C 11 0.263941 -0.408953 0.122094 -0.381651 (3 -1)
XX -3.403958 -0.988139 -1.327861 F 20 C 11 0.278567 -0.329892 0.111977 -0.417677 (3 -1)
XX -4.008509 -0.977283 -0.901570 F 21 C 11 0.275757 -0.357578 0.117759 -0.410862 (3 -1)
XX 2.647312 -1.388365 0.757762 RING 0.009709 0.047401 -1.705495 0.002538 (3 1)
XX 3.835377 -1.469152 -0.063134 F 22 C 14 0.259972 -0.413379 0.120425 -0.371249 (3 -1)
XX 2.130034 -2.092204 -0.018091 F 23 N 8 0.008284 0.034455 0.079350 0.001089 (3 -1)
XX 3.430733 -1.492149 -0.691258 F 23 C 14 0.275820 -0.333961 0.113418 -0.411594 (3 -1)
XX 4.044733 -1.079794 -0.667814 F 24 C 14 0.271921 -0.366457 0.121642 -0.402493 (3 -1)
XX 3.858033 1.432882 0.083942 F 25 C 15 0.261147 -0.411593 0.120053 -0.374334 (3 -1)
XX 2.165741 2.057211 0.016354 F 26 N 9 0.008179 0.033551 0.089713 0.001037 (3 -1)
XX 3.447551 1.461498 0.707563 F 26 C 15 0.275414 -0.334903 0.113570 -0.410694 (3 -1)
XX 2.430315 1.793459 -0.216826 RING 0.007610 0.037269 -1.237054 0.001829 (3 1)
XX 4.054913 1.038797 0.688786 F 27 C 15 0.271402 -0.368657 0.121615 -0.401283 (3 -1)
XX -1.269033 -2.811196 0.914546 H 28 N 7 0.341276 -1.781765 0.055561 -0.502400 (3 -1)
XX -1.804995 -2.026290 1.736016 H 29 N 7 0.338071 -1.757048 0.057028 -0.496559 (3 -1)
XX 2.003547 -2.054328 1.435518 H 30 C 3 0.344193 -1.836262 0.047576 -0.512497 (3 -1)
XX 0.840769 -2.486486 1.739609 H 31 N 8 0.342969 -1.807967 0.051596 -0.507732 (3 -1)
XX 0.871976 2.524648 -1.729447 H 32 N 9 0.339501 -1.814837 0.046628 -0.505254 (3 -1)
XX -0.967264 1.575696 -0.814424 H 32 C 10 0.287566 -0.880434 0.081169 -0.358846 (3 -1)
XX 2.684667 1.383711 -0.763398 RING 0.009412 0.045915 -1.595961 0.002480 (3 1)
XX 2.056333 2.029254 -1.494677 H 33 N 9 0.344383 -1.840487 0.045696 -0.512972 (3 -1)
XX -0.070145 2.418193 -1.582681 N 34 H 32 0.020278 0.076985 0.473686 0.002136 (3 -1)
XX -2.048005 2.099793 -1.437317 H 35 N 34 0.341714 -1.786377 0.048562 -0.503234 (3 -1)
XX -1.336323 2.872752 -0.789311 H 36 N 34 0.337265 -1.747531 0.049883 -0.494243 (3 -1)

M4a Singlet state

```
XX -1.072527 -0.560987 -0.506207 C   2 C   1  0.261531 -0.638804  0.062587 -0.219799 (3 -1)
XX  0.000621  0.000293 -0.624408 RING         0.018271  0.135820 -1.188049  0.007547 (3 1)
XX  0.067172 -1.216647 -0.643572 C   3 C   2  0.311572 -0.846681  0.322854 -0.314715 (3 -1)
XX  1.094666 -0.672833 -0.492726 C   4 C   3  0.297834 -0.771643  0.253417 -0.285158 (3 -1)
XX  1.074243  0.561417 -0.508564 C   5 C   4  0.261623 -0.639271  0.062444 -0.219946 (3 -1)
XX -1.092947  0.673115 -0.487297 C   6 C   1  0.298132 -0.773070  0.253741 -0.285627 (3 -1)
XX -0.065797  1.217338 -0.640565 C   6 C   5  0.311610 -0.847140  0.321914 -0.314767 (3 -1)
XX -2.573004 -1.601137 -0.361696 RING         0.010999  0.052879 -1.775813  0.002808 (3 1)
XX -0.904561 -1.572388 -0.788067 N   7 C   2  0.334473 -0.900253  0.097712 -0.547122 (3 -1)
XX -0.045993 -2.467057 -0.894436 RING         0.021683  0.131504 -1.699100  0.006531 (3 1)
XX  0.959398 -1.776452 -0.632159 N   8 C   3  0.284910 -0.842418  0.037531 -0.339210 (3 -1)
XX  2.409949 -1.387560 -0.701015 RING         0.008650  0.043992 -1.073678  0.002081 (3 1)
XX  2.574786  1.607225 -0.364697 RING         0.010890  0.052405 -1.748161  0.002789 (3 1)
XX  0.905078  1.572627 -0.790836 N   9 C   5  0.334358 -0.898499  0.097810 -0.546896 (3 -1)
XX  0.045359  2.467519 -0.895251 RING         0.021669  0.131364 -1.701148  0.006525 (3 1)
XX -2.405819  1.385318 -0.699755 RING         0.008676  0.044351 -1.071238  0.002112 (3 1)
XX -0.957531  1.776957 -0.623339 N  10 C   6  0.285079 -0.843846  0.040732 -0.339877 (3 -1)
XX -2.141977 -0.006283 -0.180378 C  11 C   1  0.300560 -0.756882  0.295229 -0.290915 (3 -1)
XX -1.620945 -1.312345  0.435556 RING         0.011465  0.058387 -2.230077  0.002431 (3 1)
XX -3.027657 -0.591494  0.408924 C  12 C  11  0.266053 -0.660914  0.134523 -0.233133 (3 -1)
XX -2.743950  2.071375 -0.367748 RING         0.009203  0.042045 -1.594316  0.001660 (3 1)
XX -3.288362  0.411867 -0.117264 C  13 C  11  0.269377 -0.682168  0.123220 -0.238487 (3 -1)
XX -4.300805 -0.163688  0.633714 RING         0.014468  0.085063 -1.617024  0.003001 (3 1)
XX  2.143512  0.006879 -0.183592 C  14 C   4  0.300873 -0.758342  0.295743 -0.291536 (3 -1)
XX  3.289123 -0.412404 -0.117106 C  15 C  14  0.269330 -0.682058  0.122575 -0.238344 (3 -1)
XX  1.621359  1.307092  0.433937 RING         0.011558  0.058900 -2.243719  0.002449 (3 1)
XX  2.325847  1.841394  0.009920 RING         0.010127  0.051231 -1.303736  0.002556 (3 1)
XX  3.028017  0.592814  0.406459 C  16 C  14  0.265962 -0.660558  0.133947 -0.232939 (3 -1)
XX  4.294976  0.163109  0.636557 RING         0.014541  0.085747 -1.597482  0.003036 (3 1)
XX -2.691924  2.135971 -1.316997 F  17 N  10  0.021238  0.076378  0.110242 -0.000347 (3 -1)
```

XX -3.808106 1.140343 -0.717979 F 17 C 13 0.260756 -0.376235 0.115892 -0.376554 (3 -1)
XX -4.282104 0.774661 -0.281052 F 18 C 13 0.274035 -0.353198 0.114487 -0.407952 (3 -1)
XX -3.875495 1.328245 0.006698 F 19 C 13 0.265050 -0.415633 0.117964 -0.383521 (3 -1)
XX -2.921354 -2.271129 -0.502897 F 20 N 7 0.017204 0.063049 0.073652 0.000091 (3 -1)
XX -3.520890 -1.538055 0.664771 F 20 C 12 0.257084 -0.427448 0.115107 -0.362753 (3 -1)
XX -1.746703 -1.970489 0.308653 F 21 N 7 0.012464 0.050824 1.070050 0.001448 (3 -1)
XX -2.940075 -1.398100 1.120190 F 21 C 12 0.270537 -0.351769 0.110527 -0.399788 (3 -1)
XX -3.574598 -1.033680 1.219088 F 22 C 12 0.273850 -0.364690 0.115646 -0.407150 (3 -1)
XX -4.566323 -0.160697 0.763331 F 22 F 18 0.015094 0.070982 0.370933 0.000999 (3 -1)
XX 4.282667 -0.778251 -0.273836 F 23 C 15 0.274155 -0.352076 0.114452 -0.408227 (3 -1)
XX 2.738582 -2.057485 -0.383649 RING 0.009175 0.041818 -1.541240 0.001640 (3 1)
XX 3.871853 -1.330929 0.009928 F 24 C 15 0.264843 -0.415596 0.118132 -0.383026 (3 -1)
XX 2.689290 -2.133453 -1.330355 F 25 N 8 0.020660 0.074982 0.120701 -0.000207 (3 -1)
XX 3.811112 -1.141372 -0.715002 F 25 C 15 0.261051 -0.376110 0.115835 -0.377243 (3 -1)
XX 2.921564 2.271044 -0.509586 F 26 N 9 0.016878 0.062261 0.075989 0.000160 (3 -1)
XX 3.521170 1.538999 0.662310 F 26 C 16 0.257293 -0.427025 0.115118 -0.363354 (3 -1)
XX 1.740484 1.967662 0.304083 F 27 N 9 0.012515 0.051140 1.179382 0.001467 (3 -1)
XX 2.938863 1.400510 1.116246 F 27 C 16 0.270481 -0.351873 0.110604 -0.399660 (3 -1)
XX 3.572740 1.035957 1.217750 F 28 C 16 0.273886 -0.364736 0.115532 -0.407231 (3 -1)
XX 4.567150 0.159267 0.771737 F 28 F 23 0.015227 0.071386 0.349320 0.000973 (3 -1)
XX -1.459052 2.846817 0.007947 H 29 N 10 0.336885 -1.745810 0.050495 -0.493755 (3 -1)
XX -2.619858 2.271445 -0.003336 H 29 F 19 0.009807 0.037351 0.982044 0.001025 (3 -1)
XX -1.871842 2.623491 -1.132733 H 30 N 10 0.344221 -1.818734 0.046614 -0.510719 (3 -1)
XX 0.884586 2.928928 -1.170114 H 31 N 9 0.337368 -1.829611 0.039722 -0.504639 (3 -1)
XX -0.006274 2.830901 -0.983820 H 31 N 10 0.023731 0.084529 0.372438 0.001761 (3 -1)
XX 2.163709 1.610208 0.006964 CAGE 0.010035 0.051343 -0.784190 0.002604 (3 3)
XX 2.095017 2.343185 -1.070287 H 32 N 9 0.344251 -1.864184 0.040837 -0.515211 (3 -1)
XX 1.861426 -2.624497 -1.159087 H 33 N 8 0.343928 -1.815686 0.046661 -0.510014 (3 -1)
XX 1.480670 -2.839804 -0.006699 H 34 N 8 0.337009 -1.747316 0.050338 -0.494087 (3 -1)
XX 2.608038 -2.295219 0.028671 H 34 F 24 0.010003 0.038586 1.058424 0.001100 (3 -1)

XX -0.887047 -2.930465 -1.160618 H 35 N   7 0.337369 -1.829647 0.039665 -0.504655 (3 -1)
XX 0.004911 -2.832091 -0.981528 H 35 N   8 0.023766 0.084533 0.369906 0.001751 (3 -1)
XX -2.142929 -1.595189 0.022847 CAGE   0.010058 0.051757 -0.772122 0.002617 (3 3)
XX -2.322027 -1.848333 0.023151 RING   0.010187 0.051539 -1.334344 0.002557 (3 1)
XX -2.096257 -2.342383 -1.059177 H 36 N   7 0.344305 -1.864513 0.040783 -0.515332 (3 -1)

M4b Triplet state
XX 1.049244 -0.451937 0.425911 C   2 C   1 0.302549 -0.786894 0.227360 -0.293128 (3 -1)
XX 0.003548 -0.892442 0.848680 C   3 C   2 0.307489 -0.816821 0.204949 -0.300996 (3 -1)
XX -0.000014 0.000099 -0.000016 RING   0.019350 0.150089 -1.179742 0.008538 (3 1)
XX -1.045667 -0.455407 0.431115 C   4 C   3 0.302602 -0.787201 0.227432 -0.293205 (3 -1)
XX -1.049275 0.452126 -0.425950 C   5 C   4 0.302553 -0.786912 0.227367 -0.293134 (3 -1)
XX 1.045644 0.455593 -0.431154 C   6 C   1 0.302608 -0.787231 0.227445 -0.293217 (3 -1)
XX -0.003575 0.892644 -0.848700 C   6 C   5 0.307479 -0.816767 0.204940 -0.300976 (3 -1)
XX 1.088785 -1.372563 1.313592 C   7 C   2 0.251926 -0.590466 0.034941 -0.206309 (3 -1)
XX -1.078144 -1.374229 1.320620 C   8 C   3 0.251920 -0.590455 0.034977 -0.206286 (3 -1)
XX -1.088824 1.372796 -1.313581 C   9 C   5 0.251928 -0.590475 0.034941 -0.206312 (3 -1)
XX -2.098454 -0.003794 0.004912 C   9 C   8 0.261940 -0.624914 0.026595 -0.225082 (3 -1)
XX 1.078142 1.374446 -1.320601 C 10 C   6 0.251921 -0.590462 0.034977 -0.206289 (3 -1)
XX 2.098426 0.003920 -0.004969 C 10 C   7 0.261941 -0.624921 0.026571 -0.225084 (3 -1)
XX 3.249160 0.478833 0.370929 C 12 C 11 0.269686 -0.684026 0.075806 -0.234348 (3 -1)
XX 3.250494 -0.471047 -0.379442 C 13 C 11 0.269623 -0.683569 0.076101 -0.234218 (3 -1)
XX -3.250575 0.471094 0.379329 C 15 C 14 0.269629 -0.683597 0.076111 -0.234227 (3 -1)
XX -3.249114 -0.478905 -0.370902 C 16 C 14 0.269685 -0.684022 0.075808 -0.234347 (3 -1)
XX 3.812366 -0.825367 -1.207807 F 17 C 13 0.269515 -0.391543 0.123239 -0.395587 (3 -1)
XX 4.033467 -1.158196 -0.578252 F 18 C 13 0.272826 -0.362684 0.121449 -0.404204 (3 -1)
XX 3.406440 -1.371659 -0.910531 F 19 C 13 0.275119 -0.338241 0.116164 -0.409923 (3 -1)
XX 3.785184 0.833499 1.215839 F 20 C 12 0.269193 -0.392346 0.123852 -0.394781 (3 -1)
XX 4.044082 1.145560 0.590308 F 21 C 12 0.273380 -0.359040 0.120809 -0.405557 (3 -1)
XX 3.410509 1.385987 0.889732 F 22 C 12 0.274882 -0.340387 0.116355 -0.409356 (3 -1)

XX -4.033680 1.158087 0.578155 F 23 C 15 0.272837 -0.362602 0.121436 -0.404231 (3 -1)
XX -3.812375 0.825431 1.207740 F 24 C 15 0.269496 -0.391685 0.123235 -0.395538 (3 -1)
XX -3.406649 1.371774 0.910253 F 25 C 15 0.275121 -0.338219 0.116159 -0.409928 (3 -1)
XX -4.043996 -1.145691 -0.590252 F 26 C 16 0.273389 -0.358959 0.120804 -0.405580 (3 -1)
XX -3.784944 -0.833828 -1.215829 F 27 C 16 0.269179 -0.392453 0.123852 -0.394743 (3 -1)
XX -3.410375 -1.386238 -0.889439 F 28 C 16 0.274884 -0.340370 0.116351 -0.409360 (3 -1)
XX 1.249218 1.734568 -2.425427 H 29 C 10 0.275259 -0.938150 0.011056 -0.279662 (3 -1)
XX 1.288531 2.490866 -1.622722 H 30 C 10 0.275292 -0.939813 0.010814 -0.279691 (3 -1)
XX 2.110882 1.758101 -1.736867 H 31 C 10 0.280098 -0.972421 0.009339 -0.288418 (3 -1)
XX -1.304147 2.489477 -1.610057 H 32 C   9 0.275240 -0.939302 0.010884 -0.279597 (3 -1)
XX -1.264959 1.736809 -2.416134 H 33 C   9 0.275343 -0.938795 0.011044 -0.279843 (3 -1)
XX -2.124430 1.754626 -1.724903 H 34 C   9 0.280262 -0.973670 0.009372 -0.288723 (3 -1)
XX 1.265071 -1.736337 2.416202 H 35 C   7 0.275340 -0.938775 0.011043 -0.279837 (3 -1)
XX 1.303937 -2.489219 1.610298 H 36 C   7 0.275240 -0.939299 0.010883 -0.279596 (3 -1)
XX 2.124398 -1.754516 1.724798 H 37 C   7 0.280263 -0.973676 0.009371 -0.288725 (3 -1)
XX -2.110861 -1.757903 1.736944 H 38 C   8 0.280101 -0.972443 0.009337 -0.288424 (3 -1)
XX -1.288469 -2.490631 1.622838 H 39 C   8 0.275293 -0.939820 0.010812 -0.279693 (3 -1)
XX -1.249181 -1.734247 2.425475 H 40 C   8 0.275256 -0.938131 0.011054 -0.279656 (3 -1)

M4b Singlet state
XX -0.947634 -0.691355 1.124120 C   2 C   1 0.258274 -0.610625 0.057360 -0.216350 (3 -1)
XX 0.000489 0.001613 1.221134 RING 0.021986 0.163609 -1.092900 0.008369 (3 1)
XX 0.078037 -1.223100 1.432573 C   3 C   2 0.337513 -0.936334 0.382932 -0.366840 (3 -1)
XX 1.036825 -0.584639 1.146211 C   4 C   3 0.256373 -0.602007 0.052342 -0.213658 (3 -1)
XX 0.948969 0.694179 1.124161 C   5 C   4 0.258283 -0.610685 0.057301 -0.216368 (3 -1)
XX -1.035678 0.587622 1.143207 C   6 C   1 0.256229 -0.601290 0.052412 -0.213429 (3 -1)
XX -0.077102 1.226732 1.429654 C   6 C   5 0.337492 -0.936215 0.382816 -0.366788 (3 -1)
XX -1.004832 -1.835469 1.741075 C   7 C   2 0.248484 -0.574942 0.037439 -0.202550 (3 -1)
XX 1.201419 -1.723627 1.782171 C   8 C   3 0.250253 -0.581183 0.039844 -0.205664 (3 -1)
XX 2.380375 -1.396591 0.901034 RING 0.006449 0.033372 -1.130385 0.001824 (3 1)

XX 1.005466 1.839380 1.739124 C 9 C 5 0.248517 -0.575087 0.037453 -0.202603 (3 -1)
XX -2.380393 1.397684 0.897377 RING 0.006458 0.033444 -1.125078 0.001830 (3 1)
XX -1.200429 1.729243 1.775371 C 10 C 6 0.250288 -0.581335 0.039863 -0.205728 (3 -1)
XX -1.784644 -0.094946 0.361893 C 11 C 1 0.327455 -0.881254 0.354575 -0.346990 (3 -1)
XX -1.474744 -1.444878 0.227533 RING 0.012999 0.072482 -1.592522 0.003216 (3 1)
XX -1.551284 1.485483 0.211262 RING 0.009991 0.047512 -3.262345 0.001982 (3 1)
XX -1.752442 2.102410 0.512431 RING 0.008865 0.041767 -2.576635 0.001972 (3 1)
XX -2.614975 0.464100 -0.344548 C 12 C 11 0.261057 -0.654394 0.060518 -0.221273 (3 -1)
XX -2.440764 -0.707812 -0.489279 C 13 C 11 0.263107 -0.661938 0.067282 -0.223958 (3 -1)
XX 1.786024 0.095850 0.363514 C 14 C 4 0.327499 -0.881478 0.354714 -0.347095 (3 -1)
XX 1.479868 1.446053 0.228799 RING 0.013009 0.072599 -1.590148 0.003222 (3 1)
XX 1.547098 -1.491436 0.214555 RING 0.009943 0.047027 -3.573846 0.001950 (3 1)
XX 2.615129 -0.465112 -0.343092 C 15 C 14 0.261054 -0.654395 0.060494 -0.221266 (3 -1)
XX 2.442081 0.706598 -0.489141 C 16 C 14 0.263090 -0.661843 0.067354 -0.223934 (3 -1)
XX 1.265110 2.366444 2.743631 H 17 C 9 0.273908 -0.927918 0.012944 -0.277167 (3 -1)
XX 2.057213 2.297926 1.973792 H 18 C 9 0.280943 -0.982258 0.011332 -0.289733 (3 -1)
XX 1.204568 2.996749 1.833990 H 19 C 9 0.278104 -0.964654 0.011556 -0.284766 (3 -1)
XX 2.186785 -1.938639 2.368754 H 20 C 8 0.279299 -0.971046 0.012939 -0.286978 (3 -1)
XX 1.268642 -2.504389 2.637108 H 21 C 8 0.277852 -0.956353 0.012286 -0.284247 (3 -1)
XX 1.803971 -2.730013 1.693552 H 22 C 8 0.276856 -0.956508 0.012624 -0.283005 (3 -1)
XX 2.293234 -1.572873 0.759241 CAGE 0.006402 0.034189 -0.785293 0.001948 (3 3)
XX 2.447286 -2.142052 0.681897 RING 0.006966 0.037224 -1.259262 0.002017 (3 1)
XX -1.270360 -2.357523 2.746684 H 23 C 7 0.273906 -0.927929 0.012936 -0.277183 (3 -1)
XX -2.056537 -2.297509 1.970504 H 24 C 7 0.280977 -0.982540 0.011326 -0.289806 (3 -1)
XX -1.199419 -2.992927 1.841226 H 25 C 7 0.278131 -0.964831 0.011557 -0.284819 (3 -1)
XX -1.268470 2.511247 2.628715 H 26 C 10 0.277843 -0.956252 0.012283 -0.284236 (3 -1)
XX -1.798935 2.737434 1.682490 H 27 C 10 0.276833 -0.956307 0.012623 -0.282950 (3 -1)
XX -2.297989 1.564407 0.762162 CAGE 0.006418 0.034278 -0.796424 0.001950 (3 3)
XX -2.451689 2.136037 0.683609 RING 0.006989 0.037348 -1.257610 0.002019 (3 1)
XX -2.187701 1.948416 2.357117 H 28 C 10 0.279388 -0.971742 0.012908 -0.287146 (3 -1)

XX 3.031155 0.163560 -1.512876 RING 0.014708 0.087959 -1.559325 0.003167 (3 1)
XX 2.575479 1.303533 -1.361721 F 29 C 16 0.273814 -0.379321 0.115257 -0.406536 (3 -1)
XX 1.285522 1.895381 0.424409 F 30 C 5 0.015196 0.064421 0.831412 0.001496 (3 -1)
XX 2.393641 1.734884 -0.796918 F 30 C 16 0.275035 -0.312427 0.112955 -0.409946 (3 -1)
XX 3.086127 1.479721 -0.847577 F 31 C 16 0.270169 -0.385752 0.118312 -0.397771 (3 -1)
XX 2.891305 -1.829600 1.359273 F 32 C 8 0.009941 0.044820 1.308850 0.001919 (3 -1)
XX 3.307874 -1.279599 -0.246371 F 32 C 15 0.267870 -0.383385 0.117357 -0.392516 (3 -1)
XX 1.508325 -1.604321 0.245846 F 33 C 3 0.009964 0.044129 2.154808 0.001623 (3 -1)
XX 1.758353 -2.107619 0.517676 RING 0.008749 0.041219 -2.585530 0.001958 (3 1)
XX 2.732895 -1.481547 -0.674687 F 33 C 15 0.271977 -0.359258 0.118313 -0.402519 (3 -1)
XX 1.977679 -2.463587 0.499321 F 33 H 22 0.009257 0.042473 0.823925 0.001837 (3 -1)
XX 3.271576 -1.060093 -0.949374 F 34 C 15 0.277827 -0.334245 0.115058 -0.416211 (3 -1)
XX 3.105079 0.134723 -1.921031 F 34 F 29 0.016433 0.073863 0.227644 0.000600 (3 -1)
XX -3.031698 -0.164104 -1.509035 RING 0.014734 0.088347 -1.546808 0.003195 (3 1)
XX -2.578508 -1.306164 -1.360454 F 35 C 13 0.273901 -0.378954 0.115014 -0.406749 (3 -1)
XX -1.279862 -1.891179 0.421961 F 36 C 2 0.015158 0.064182 0.802666 0.001488 (3 -1)
XX -2.389981 -1.735937 -0.796950 F 36 C 13 0.275108 -0.312220 0.112899 -0.410106 (3 -1)
XX -3.084257 -1.483862 -0.841859 F 37 C 13 0.269984 -0.386665 0.118479 -0.397310 (3 -1)
XX -2.902065 1.822006 1.365060 F 38 C 10 0.010011 0.045106 1.213454 0.001930 (3 -1)
XX -3.309561 1.276770 -0.246956 F 38 C 12 0.267801 -0.383948 0.117445 -0.392337 (3 -1)
XX -1.977043 2.470075 0.493668 F 38 H 19 0.009425 0.043154 0.800266 0.001844 (3 -1)
XX -1.508045 1.613167 0.246831 F 39 C 6 0.010021 0.044266 1.856250 0.001612 (3 -1)
XX -2.734751 1.480581 -0.675009 F 39 C 12 0.271876 -0.359826 0.118410 -0.402282 (3 -1)
XX -3.272762 1.058525 -0.950271 F 40 C 12 0.277918 -0.333609 0.114974 -0.416407 (3 -1)
XX -3.110972 -0.136663 -1.922757 F 40 F 35 0.016560 0.074278 0.213465 0.000581 (3 -1)

M4c Triplet state
XX -1.056805 0.617702 -0.012350 C 2 C 1 0.302701 -0.803158 0.179959 -0.293003 (3 -1)
XX 0.000343 0.000386 -0.006854 RING 0.019917 0.155534 -1.199961 0.008707 (3 1)
XX 0.001951 1.204274 -0.007171 C 3 C 2 0.316695 -0.874593 0.210652 -0.320506 (3 -1)

XX 1.059000 0.614327 -0.001928 C 4 C 3 0.302699 -0.803005 0.179941 -0.292990 (3 -1)
XX 1.057492 -0.616951 -0.012173 C 5 C 4 0.302735 -0.803327 0.179998 -0.293069 (3 -1)
XX -1.058289 -0.613567 -0.001404 C 6 C 1 0.302685 -0.802945 0.179888 -0.292960 (3 -1)
XX -0.001199 -1.203512 -0.006498 C 6 C 5 0.316716 -0.874722 0.210645 -0.320549 (3 -1)
XX -1.023519 1.813838 -0.003928 H 7 C 2 0.289393 -1.070794 0.014222 -0.306707 (3 -1)
XX 1.029436 1.810465 -0.010946 H 8 C 3 0.289416 -1.070941 0.014220 -0.306760 (3 -1)
XX 1.024235 -1.813073 -0.003073 H 9 C 5 0.289401 -1.070852 0.014215 -0.306722 (3 -1)
XX -1.028667 -1.809685 -0.009792 H 10 C 6 0.289437 -1.071077 0.014211 -0.306800 (3 -1)
XX -2.129683 0.002176 -0.005188 C 11 C 1 0.276090 -0.682244 0.119944 -0.245926 (3 -1)
XX -3.250586 -0.524208 0.307879 C 12 C 11 0.269563 -0.686567 0.076603 -0.233316 (3 -1)
XX -3.257997 0.523671 -0.310063 C 13 C 11 0.269639 -0.686799 0.076957 -0.233389 (3 -1)
XX -4.507259 -0.009360 -0.015790 RING 0.012743 0.074004 -1.690031 0.002698 (3 1)
XX 2.130412 -0.001574 -0.005605 C 14 C 4 0.276023 -0.681955 0.119763 -0.245808 (3 -1)
XX 3.251278 0.524576 0.307423 C 15 C 14 0.269611 -0.686809 0.076634 -0.233394 (3 -1)
XX 3.258383 -0.523687 -0.310405 C 16 C 14 0.269653 -0.686857 0.077010 -0.233409 (3 -1)
XX 4.505336 0.008801 -0.017045 RING 0.012771 0.074298 -1.676302 0.002715 (3 1)
XX -1.924036 1.373663 -0.829504 RING 0.010697 0.051308 -6.049680 0.002222 (3 1)
XX -4.071971 0.954651 -0.828339 F 17 C 13 0.276731 -0.355442 0.112074 -0.413363 (3 -1)
XX -3.773469 1.449336 -0.356584 F 18 C 13 0.269085 -0.400887 0.116328 -0.394162 (3 -1)
XX -3.441906 1.297711 -1.005946 F 19 C 13 0.273169 -0.355651 0.111611 -0.405377 (3 -1)
XX -4.065388 -0.969562 0.813513 F 20 C 12 0.277128 -0.351271 0.111933 -0.414301 (3 -1)
XX -4.794955 -0.015585 -0.022720 F 20 F 17 0.013277 0.062441 0.379954 0.000975 (3 -1)
XX -3.734518 -1.466633 0.366602 F 21 C 12 0.268628 -0.401318 0.116723 -0.393053 (3 -1)
XX -3.429252 -1.283639 1.021111 F 22 C 12 0.273018 -0.359154 0.112074 -0.404957 (3 -1)
XX 4.066521 0.970674 0.811670 F 23 C 15 0.277206 -0.350624 0.111871 -0.414480 (3 -1)
XX 3.733105 1.467975 0.366907 F 24 C 15 0.268549 -0.401661 0.116730 -0.392854 (3 -1)
XX 3.430221 1.282569 1.021968 F 25 C 15 0.273001 -0.359562 0.112107 -0.404909 (3 -1)
XX 1.923774 -1.375040 -0.829486 RING 0.010702 0.051304 -6.254555 0.002220 (3 1)
XX 4.072117 -0.956033 -0.828017 F 26 C 16 0.276799 -0.354995 0.111973 -0.413519 (3 -1)
XX 4.798227 0.015355 -0.024632 F 26 F 23 0.013336 0.062592 0.364754 0.000959 (3 -1)

XX 3.772376 -1.450146 -0.356403 F 27 C 16 0.269007 -0.401322 0.116346 -0.393961 (3 -1)
XX 3.441630 -1.297937 -1.006139 F 28 C 16 0.273169 -0.355759 0.111593 -0.405376 (3 -1)

M4c Singlet state
XX -1.080500 -0.596521 -0.075220 C   2 C   1 0.277580 -0.704381 0.080326 -0.246943 (3 -1)
XX -0.000022 0.000116 -0.000207 RING   0.018894 0.144265 -1.209006 0.008077 (3 1)
XX -0.024765 -1.205316 -0.157462 C   3 C   2 0.338400 -0.975108 0.296898 -0.367572 (3 -1)
XX 1.053519 -0.652857 -0.078512 C   4 C   3 0.278090 -0.708075 0.081006 -0.248136 (3 -1)
XX 1.080456 0.596738 0.074852 C   5 C   4 0.277575 -0.704355 0.080327 -0.246934 (3 -1)
XX -1.053564 0.653059 0.078263 C   6 C   1 0.278080 -0.708024 0.081002 -0.248118 (3 -1)
XX 0.024724 1.205549 0.157025 C   6 C   5 0.338400 -0.975112 0.296897 -0.367574 (3 -1)
XX -2.262126 -1.823143 -0.423029 RING   0.009815 0.050790 -2.393657 0.002616 (3 1)
XX -1.041044 -1.789882 -0.265182 H   7 C   2 0.292752 -1.099650 0.013309 -0.313781 (3 -1)
XX 0.958719 -1.843323 -0.269772 H   8 C   3 0.294254 -1.116957 0.015600 -0.317707 (3 -1)
XX 2.124233 -1.419520 0.082034 RING   0.011593 0.071472 -1.340919 0.003696 (3 1)
XX 2.261948 1.824089 0.425534 RING   0.009802 0.050667 -2.439340 0.002607 (3 1)
XX 1.041004 1.790141 0.264533 H   9 C   5 0.292756 -1.099682 0.013310 -0.313791 (3 -1)
XX -2.124409 1.419636 -0.081871 RING   0.011593 0.071472 -1.340947 0.003696 (3 1)
XX -0.958765 1.843569 0.269289 H 10 C   6 0.294258 -1.116998 0.015602 -0.317716 (3 -1)
XX -2.144950 0.035066 0.002457 C 11 C   1 0.320976 -0.863369 0.298002 -0.330810 (3 -1)
XX -2.122755 -1.431286 0.233667 RING   0.011548 0.067348 -1.266976 0.003617 (3 1)
XX -1.939899 2.171475 0.023938 C 12 H 10 0.020435 0.082181 0.193652 0.001079 (3 -1)
XX -3.261038 0.608581 -0.008866 C 12 C 11 0.264187 -0.666778 0.063742 -0.224755 (3 -1)
XX -3.204475 -0.591711 0.013884 C 13 C 11 0.261778 -0.655748 0.060759 -0.221414 (3 -1)
XX -4.437197 0.018426 -0.184694 RING   0.014408 0.085306 -1.577416 0.003071 (3 1)
XX 2.144902 -0.034924 -0.002571 C 14 C   4 0.320972 -0.863345 0.298003 -0.330801 (3 -1)
XX 2.122824 1.431171 -0.234036 RING   0.011548 0.067356 -1.267048 0.003618 (3 1)
XX 1.939751 -2.171238 -0.023858 C 15 H   8 0.020428 0.082164 0.194020 0.001080 (3 -1)
XX 3.260903 -0.608566 0.008942 C 15 C 14 0.264190 -0.666793 0.063733 -0.224759 (3 -1)
XX 3.204552 0.591743 -0.013914 C 16 C 14 0.261761 -0.655666 0.060750 -0.221387 (3 -1)

XX 4.436989 -0.018425 0.184568 RING 0.014411 0.085335 -1.576588 0.003072 (3 1)
XX -4.049136 -1.235586 0.123329 F 17 C 13 0.279148 -0.333021 0.111823 -0.419009 (3 -1)
XX -2.224949 -1.889238 -0.581773 F 18 H 7 0.009878 0.046829 1.168176 0.002099 (3 -1)
XX -3.576747 -1.518222 -0.373653 F 18 C 13 0.269776 -0.386879 0.116850 -0.396553 (3 -1)
XX -2.012704 -1.972591 0.281754 F 19 H 7 0.013996 0.062958 0.295662 0.002156 (3 -1)
XX -3.439695 -1.578125 0.356200 F 19 C 13 0.270348 -0.375049 0.113599 -0.398558 (3 -1)
XX -4.069002 1.204702 -0.345732 F 20 C 12 0.274500 -0.378928 0.112388 -0.408229 (3 -1)
XX -4.767869 -0.002651 -0.311948 F 20 F 17 0.015461 0.071166 0.298723 0.000816 (3 -1)
XX -3.946450 1.324407 0.379664 F 21 C 12 0.273031 -0.389544 0.113032 -0.404208 (3 -1)
XX -3.517213 1.642441 -0.132550 F 22 C 12 0.272430 -0.331816 0.108530 -0.404034 (3 -1)
XX 3.946095 -1.324622 -0.379523 F 23 C 15 0.273022 -0.389591 0.113050 -0.404186 (3 -1)
XX 4.068847 -1.204694 0.345812 F 24 C 15 0.274504 -0.378887 0.112395 -0.408240 (3 -1)
XX 3.516952 -1.642423 0.132943 F 25 C 15 0.272435 -0.331813 0.108532 -0.404043 (3 -1)
XX 4.049324 1.235473 -0.123477 F 26 C 16 0.279144 -0.333074 0.111811 -0.419002 (3 -1)
XX 4.767984 0.002607 0.311740 F 26 F 24 0.015467 0.071188 0.297915 0.000816 (3 -1)
XX 2.012741 1.973032 -0.281986 F 27 H 9 0.014003 0.062981 0.295741 0.002155 (3 -1)
XX 3.439901 1.578193 -0.356126 F 27 C 16 0.270343 -0.375077 0.113583 -0.398545 (3 -1)
XX 2.225700 1.888452 0.580015 F 28 H 9 0.009860 0.046800 1.213428 0.002104 (3 -1)
XX 3.577135 1.518106 0.373691 F 28 C 16 0.269781 -0.386887 0.116841 -0.396564 (3 -1)

M4d Triplet state
XX 1.062911 -0.606937 0.042283 C 2 C 1 0.300705 -0.773635 0.220071 -0.289233 (3 -1)
XX 0.000021 0.000024 -0.003611 RING 0.019586 0.149371 -1.180597 0.008420 (3 1)
XX -0.001086 -1.222450 -0.004733 C 3 C 2 0.303324 -0.788222 0.212786 -0.293733 (3 -1)
XX -1.063942 -0.604633 -0.049866 C 4 C 3 0.300672 -0.773489 0.219921 -0.289163 (3 -1)
XX -1.062911 0.606840 0.043272 C 5 C 4 0.300672 -0.773430 0.220126 -0.289174 (3 -1)
XX 1.064038 0.604618 -0.049646 C 6 C 1 0.300651 -0.773334 0.220040 -0.289133 (3 -1)
XX 0.001137 1.222445 -0.003458 C 6 C 5 0.303206 -0.787552 0.212816 -0.293513 (3 -1)
XX 2.176000 -1.166377 0.489391 RING 0.015867 0.089110 -1.452998 0.003845 (3 1)
XX 1.063902 -1.839336 0.211924 C 7 C 2 0.256670 -0.638727 0.030554 -0.210958 (3 -1)

XX -0.194997 -2.664938 0.256133 RING 0.010738 0.064767 -1.195057 0.003198 (3 1)

XX 0.184488 -2.665452 -0.262660 RING 0.010712 0.064797 -1.176318 0.003209 (3 1)

XX -1.067549 -1.836598 -0.222222 C 8 C 3 0.256668 -0.638700 0.030541 -0.210953 (3 -1)

XX -2.179222 -1.161589 -0.494200 RING 0.015887 0.089234 -1.452067 0.003849 (3 1)

XX 0.002437 -2.665759 -0.014238 CAGE 0.010610 0.068293 -0.936892 0.003562 (3 3)

XX -2.176164 1.166986 0.486475 RING 0.015883 0.089311 -1.451611 0.003857 (3 1)

XX -1.064291 1.839374 0.211992 C 9 C 5 0.256626 -0.638507 0.030535 -0.210885 (3 -1)

XX 0.189215 2.664801 0.253792 RING 0.010711 0.064787 -1.178069 0.003210 (3 1)

XX -2.161234 0.001194 -0.000281 C 14 C 4 0.258936 -0.602905 0.039705 -0.215580 (3 -1)

XX 2.180069 1.162657 -0.490968 RING 0.015880 0.089202 -1.452248 0.003849 (3 1)

XX -0.183459 2.665045 -0.260152 RING 0.010711 0.064799 -1.178334 0.003211 (3 1)

XX 1.068167 1.836975 -0.218471 C 10 C 6 0.256636 -0.638550 0.030543 -0.210899 (3 -1)

XX 2.161296 -0.001963 -0.001491 C 11 C 1 0.258928 -0.602903 0.039402 -0.215568 (3 -1)

XX 0.002949 2.665881 -0.002823 CAGE 0.010602 0.068236 -0.938803 0.003562 (3 3)

XX 2.698202 -1.153049 0.588472 C 12 C 7 0.019451 0.071832 0.121061 0.000565 (3 -1)

XX 3.286824 0.194895 0.594210 C 12 C 11 0.265343 -0.662657 0.073036 -0.224699 (3 -1)

XX 2.703037 1.148341 -0.587835 C 13 C 10 0.019473 0.071915 0.121069 0.000563 (3 -1)

XX 3.291669 -0.198553 -0.589983 C 13 C 11 0.265366 -0.662797 0.073018 -0.224726 (3 -1)

XX -2.701140 1.153055 0.585220 C 15 C 9 0.019535 0.072061 0.118000 0.000551 (3 -1)

XX -3.286732 -0.198938 0.594962 C 15 C 14 0.265385 -0.662860 0.073126 -0.224771 (3 -1)

XX -2.701764 -1.147406 -0.590958 C 16 C 8 0.019473 0.071908 0.121880 0.000563 (3 -1)

XX -3.291866 0.200100 -0.587098 C 16 C 14 0.265365 -0.662801 0.072947 -0.224724 (3 -1)

XX 4.030333 -0.099241 -1.325823 F 17 C 13 0.277949 -0.369414 0.109028 -0.415372 (3 -1)

XX 3.083795 -2.111801 -0.305498 RING 0.005532 0.033085 -1.395999 0.001707 (3 1)

XX 3.908823 -0.814360 -1.179608 F 18 C 13 0.277025 -0.382669 0.106727 -0.413015 (3 -1)

XX 3.425046 -0.435785 -1.596676 F 19 C 13 0.275011 -0.354153 0.107469 -0.409276 (3 -1)

XX 3.084938 2.107138 0.303035 RING 0.005624 0.033604 -1.399773 0.001716 (3 1)

XX 3.898260 0.812163 1.188238 F 20 C 12 0.277112 -0.381936 0.106924 -0.413225 (3 -1)

XX 3.411112 0.433749 1.601425 F 21 C 12 0.274934 -0.354234 0.107768 -0.409106 (3 -1)

XX 4.019526 0.097538 1.336783 F 22 C 12 0.277919 -0.369747 0.109005 -0.415291 (3 -1)

XX -3.084642 -2.108708 0.295944 RING 0.005605 0.033492 -1.398320 0.001714 (3 1)
XX -3.898874 -0.818391 1.186080 F 23 C 15 0.277073 -0.382547 0.106845 -0.413122 (3 -1)
XX -3.411578 -0.442246 1.601181 F 24 C 15 0.274970 -0.353832 0.107656 -0.409191 (3 -1)
XX -4.019343 -0.104164 1.337745 F 25 C 15 0.277883 -0.369877 0.109062 -0.415210 (3 -1)
XX -3.079276 2.128630 -0.313782 RING 0.005681 0.033964 -1.408679 0.001723 (3 1)
XX -3.906818 0.820313 -1.174611 F 26 C 16 0.277022 -0.382668 0.106824 -0.413002 (3 -1)
XX -3.426118 0.439599 -1.593144 F 27 C 16 0.275007 -0.354190 0.107530 -0.409265 (3 -1)
XX -4.032680 0.105975 -1.321402 F 28 C 16 0.277934 -0.369339 0.109090 -0.415342 (3 -1)
XX 1.205029 -2.812697 0.608386 F 29 C 7 0.279883 -0.343173 0.106685 -0.420329 (3 -1)
XX -0.485178 -3.096617 0.440091 RING 0.011433 0.064767 -1.800931 0.002378 (3 1)
XX 1.618322 -2.722407 0.000619 F 30 C 7 0.280328 -0.360622 0.106733 -0.420976 (3 -1)
XX 0.585856 -3.199428 -0.842717 F 30 C 8 0.012283 0.056674 0.398112 0.000877 (3 -1)
XX 3.090713 -2.323241 -0.887487 F 30 F 18 0.007784 0.037737 0.013582 0.001066 (3 -1)
XX 0.481493 -3.096001 -0.460722 RING 0.011361 0.064317 -1.811275 0.002367 (3 1)
XX 1.827688 -2.428207 0.645445 F 31 C 7 0.273472 -0.352867 0.109772 -0.406077 (3 -1)
XX -0.476799 3.099577 -0.451642 RING 0.011423 0.064718 -1.795081 0.002374 (3 1)
XX 1.213303 2.810241 -0.613973 F 32 C 10 0.279900 -0.343165 0.106649 -0.420361 (3 -1)
XX 0.589842 3.199303 0.835503 F 33 C 9 0.012330 0.056824 0.392588 0.000869 (3 -1)
XX 1.623701 2.718485 -0.004249 F 33 C 10 0.280295 -0.360957 0.106788 -0.420899 (3 -1)
XX 3.087291 2.320205 0.892232 F 33 F 20 0.008009 0.038556 0.011571 0.001041 (3 -1)
XX 0.486251 3.096664 0.450654 RING 0.011387 0.064471 -1.807114 0.002369 (3 1)
XX 1.835274 2.424547 -0.648446 F 34 C 10 0.273450 -0.353095 0.109584 -0.406025 (3 -1)
XX -1.206807 2.812564 0.608415 F 35 C 9 0.279848 -0.343639 0.106644 -0.420249 (3 -1)
XX 0.003873 3.354089 -0.002113 F 35 F 32 0.015908 0.071500 0.036932 0.000663 (3 -1)
XX -1.616487 2.722991 -0.002051 F 36 C 9 0.280334 -0.360730 0.106739 -0.420991 (3 -1)
XX -0.581268 3.202990 -0.841933 F 36 C 10 0.012411 0.057084 0.379551 0.000855 (3 -1)
XX -3.084159 2.328190 -0.886485 F 36 F 26 0.007880 0.038105 0.013035 0.001057 (3 -1)
XX -1.830245 2.429061 0.641403 F 37 C 9 0.273479 -0.352843 0.109530 -0.406091 (3 -1)
XX -1.833576 -2.422819 -0.655680 F 38 C 8 0.273403 -0.353340 0.109694 -0.405915 (3 -1)
XX -1.211251 -2.808239 -0.621780 F 39 C 8 0.279836 -0.343484 0.106738 -0.420220 (3 -1)

XX -0.004225 -3.352306 -0.008377 F 39 F 29 0.015879 0.071398 0.037682 0.000667 (3 -1)
XX -0.591731 -3.202254 0.829386 F 40 C 7 0.012416 0.057133 0.383375 0.000858 (3 -1)
XX -1.622807 -2.719539 -0.012520 F 40 C 8 0.280411 -0.360103 0.106727 -0.421162 (3 -1)
XX -3.087935 -2.325089 0.884155 F 40 F 23 0.007981 0.038451 0.011879 0.001044 (3 -1)

M4d Singlet state
XX 1.017353 0.552548 -0.467547 C 2 C 1 0.252532 -0.581391 0.038145 -0.206394 (3 -1)
XX -0.002878 0.002345 -0.447682 RING 0.024301 0.167138 -1.109116 0.007811 (3 1)
XX 0.076346 1.228437 -0.808251 C 3 C 2 0.338281 -0.931829 0.393431 -0.370248 (3 -1)
XX -0.939927 0.694728 -0.458420 C 4 C 3 0.250901 -0.572897 0.043057 -0.203936 (3 -1)
XX -1.023327 -0.547747 -0.470091 C 5 C 4 0.252747 -0.582404 0.037866 -0.206732 (3 -1)
XX 0.933675 -0.690353 -0.467563 C 6 C 1 0.250888 -0.572805 0.043289 -0.203911 (3 -1)
XX -0.083699 -1.220353 -0.819850 C 6 C 5 0.338315 -0.931923 0.393784 -0.370339 (3 -1)
XX 1.212755 1.582296 -1.191215 C 7 C 2 0.261697 -0.661750 0.033784 -0.218884 (3 -1)
XX 0.042259 2.702937 -1.401588 RING 0.007168 0.042082 -1.315494 0.002315 (3 1)
XX -0.991404 1.760431 -1.170875 C 8 C 3 0.258566 -0.646248 0.036478 -0.213878 (3 -1)
XX -1.221444 -1.568128 -1.204794 C 9 C 5 0.261786 -0.662148 0.033852 -0.219034 (3 -1)
XX -0.055049 -2.678411 -1.431508 RING 0.007248 0.042660 -1.312846 0.002344 (3 1)
XX 0.982788 -1.749480 -1.189516 C 10 C 6 0.258631 -0.646598 0.036457 -0.213985 (3 -1)
XX 1.639213 -0.101807 0.419061 C 11 C 1 0.334500 -0.914376 0.392367 -0.362442 (3 -1)
XX 1.478232 1.265422 0.478392 RING 0.013812 0.076651 -1.577285 0.003413 (3 1)
XX 1.513593 -1.501915 0.332015 RING 0.012774 0.068998 -1.718684 0.003061 (3 1)
XX 2.909430 1.705344 0.260469 RING 0.007761 0.045968 -1.515398 0.002073 (3 1)
XX 2.269258 0.488130 1.310640 C 12 C 11 0.259461 -0.648866 0.034855 -0.215227 (3 -1)
XX 1.367119 -1.871705 0.140253 C 13 C 10 0.014111 0.058024 0.346334 0.001410 (3 -1)
XX 2.160456 -0.729462 1.350308 C 13 C 11 0.261865 -0.660218 0.037247 -0.218973 (3 -1)
XX -1.522696 1.505526 0.345540 RING 0.012594 0.067645 -1.741532 0.003004 (3 1)
XX -1.640097 0.096983 0.426482 C 14 C 4 0.334559 -0.914640 0.392710 -0.362594 (3 -1)
XX -1.481705 -1.268941 0.469310 RING 0.013875 0.077215 -1.570502 0.003439 (3 1)
XX -1.383639 1.867812 0.161741 C 15 C 8 0.013794 0.057005 0.371683 0.001439 (3 -1)

XX -2.155615 0.714275 1.367811 C 15 C 14 0.261774 -0.659816 0.037171 -0.218837 (3 -1)
XX -2.261744 -0.502867 1.317136 C 16 C 14 0.259544 -0.649206 0.035074 -0.215365 (3 -1)
XX 2.750563 -1.396803 1.906169 F 17 C 13 0.279187 -0.359566 0.110316 -0.418668 (3 -1)
XX 2.080740 -0.222391 2.639440 RING 0.012144 0.067165 -2.194870 0.002235 (3 1)
XX 2.043588 -1.420346 2.132043 F 18 C 13 0.276131 -0.381596 0.110715 -0.411414 (3 -1)
XX 1.614567 -2.437964 -0.233855 RING 0.010944 0.060920 -1.857928 0.002538 (3 1)
XX 2.248210 -1.769934 1.517203 F 19 C 13 0.278783 -0.317702 0.108760 -0.417838 (3 -1)
XX 2.996636 1.202718 1.575809 F 20 C 12 0.279838 -0.369289 0.107715 -0.419873 (3 -1)
XX 0.156107 1.695917 1.896886 F 20 C 15 0.000740 0.004354 2.041676 0.000426 (3 -1)
XX 1.311157 1.701855 0.284293 F 21 C 2 0.016278 0.062743 0.138247 0.001015 (3 -1)
XX 1.984160 1.934723 -0.086906 RING 0.009455 0.051379 -1.566769 0.002170 (3 1)
XX 2.327218 1.518676 1.546916 F 21 C 12 0.274987 -0.338722 0.109878 -0.409363 (3 -1)
XX 0.125048 1.644457 1.802967 RING 0.000739 0.004195 -3.230659 0.000408 (3 1)
XX 2.514166 1.103936 2.126818 F 22 C 12 0.278648 -0.362689 0.109278 -0.417551 (3 -1)
XX 2.009759 -0.225422 2.848696 F 22 F 18 0.012354 0.059030 0.810478 0.001081 (3 -1)
XX -2.026893 1.400762 2.151171 F 23 C 15 0.276020 -0.381375 0.111000 -0.411162 (3 -1)
XX -2.042266 0.198135 2.668301 RING 0.011930 0.065238 -2.459907 0.002123 (3 1)
XX -1.622489 2.418772 -0.201839 RING 0.010857 0.060371 -1.836968 0.002553 (3 1)
XX -2.251159 1.752755 1.544226 F 24 C 15 0.278851 -0.318698 0.108549 -0.417982 (3 -1)
XX -2.739032 1.371401 1.943088 F 25 C 15 0.279249 -0.359157 0.110087 -0.418823 (3 -1)
XX -2.985855 -1.218681 1.587807 F 26 C 16 0.279850 -0.369217 0.107801 -0.419903 (3 -1)
XX -1.314164 -1.706932 0.267213 F 27 C 5 0.016433 0.063225 0.131354 0.000996 (3 -1)
XX -1.984250 -1.934427 -0.104440 RING 0.009573 0.052035 -1.560688 0.002186 (3 1)
XX -2.318572 -1.536536 1.541061 F 27 C 16 0.275028 -0.338134 0.109831 -0.409460 (3 -1)
XX -2.491400 -1.128349 2.130005 F 28 C 16 0.278463 -0.363782 0.109603 -0.417124 (3 -1)
XX -1.978139 0.198962 2.842625 F 28 F 23 0.012052 0.058340 1.077103 0.001170 (3 -1)
XX 2.141116 1.977600 -1.478961 F 29 C 7 0.278134 -0.347937 0.105114 -0.416495 (3 -1)
XX 3.338854 1.602866 0.117344 F 29 F 20 0.008974 0.043034 0.125409 0.000987 (3 -1)
XX 2.401351 2.057870 0.055807 F 29 F 21 0.010839 0.050528 0.147411 0.000921 (3 -1)
XX 0.295270 1.847268 -2.324351 RING 0.006914 0.038670 -1.362754 0.001985 (3 1)

XX 1.713895 1.849240 -2.068117 F 30 C 7 0.274221 -0.389502 0.112646 -0.406736 (3 -1)
XX 1.600696 2.450369 -1.646681 F 31 C 7 0.282335 -0.334784 0.106102 -0.425562 (3 -1)
XX 1.370531 -2.690802 -1.487010 F 32 C 10 0.282421 -0.354707 0.100637 -0.425628 (3 -1)
XX 1.697438 -2.935417 -0.099789 F 32 F 19 0.012581 0.058898 0.248464 0.000885 (3 -1)
XX -0.309433 -1.820385 -2.357621 RING 0.006779 0.037835 -1.375115 0.001945 (3 1)
XX 1.281374 -2.240967 -2.067025 F 33 C 10 0.275663 -0.377749 0.108523 -0.410435 (3 -1)
XX 2.047606 -2.243990 -0.322949 RING 0.010096 0.056714 -2.245892 0.002234 (3 1)
XX 1.855724 -2.145723 -1.609441 F 34 C 10 0.275973 -0.356839 0.106012 -0.411724 (3 -1)
XX 2.335392 -2.196639 -0.279426 F 34 C 13 0.010365 0.050840 0.830602 0.001226 (3 -1)
XX -1.727791 -1.819353 -2.082816 F 35 C 9 0.274137 -0.389174 0.113025 -0.406557 (3 -1)
XX -0.353375 -1.856104 -2.843828 F 35 F 33 0.007813 0.038206 0.163613 0.001123 (3 -1)
XX -1.609843 -2.428535 -1.674650 F 36 C 9 0.282398 -0.334269 0.106161 -0.425695 (3 -1)
XX -2.405724 -2.063247 0.040225 F 36 F 26 0.011043 0.051201 0.141007 0.000887 (3 -1)
XX -0.046642 -3.359064 -1.470198 F 36 F 32 0.010330 0.047012 0.063392 0.000789 (3 -1)
XX -0.163468 -2.695801 -2.240841 F 36 F 33 0.007313 0.037970 0.238443 0.001422 (3 -1)
XX -2.150684 -1.961131 -1.493436 F 37 C 9 0.278058 -0.348636 0.105199 -0.416314 (3 -1)
XX -2.939843 -1.695670 0.256801 RING 0.007583 0.044857 -1.545969 0.002041 (3 1)
XX -3.333414 -1.603469 0.129069 F 37 F 26 0.008482 0.041374 0.168348 0.001058 (3 -1)
XX -1.384517 2.703385 -1.455255 F 38 C 8 0.282442 -0.354512 0.100604 -0.425676 (3 -1)
XX -1.718656 2.936890 -0.061153 F 38 F 24 0.012731 0.059317 0.217373 0.000855 (3 -1)
XX 0.032439 3.373798 -1.424760 F 38 F 29 0.010022 0.045935 0.069780 0.000829 (3 -1)
XX -1.289120 2.264055 -2.042162 F 39 C 8 0.275715 -0.377974 0.108209 -0.410551 (3 -1)
XX 0.341417 1.897634 -2.831165 F 39 F 30 0.008140 0.039374 0.139731 0.001082 (3 -1)
XX 0.161334 2.722639 -2.208580 F 39 F 31 0.007304 0.037898 0.187722 0.001419 (3 -1)
XX -1.864851 2.156503 -1.589124 F 40 C 8 0.275970 -0.356966 0.106038 -0.411717 (3 -1)
XX -2.345673 2.193251 -0.259254 F 40 C 15 0.010142 0.050107 0.909036 0.001262 (3 -1)

M4e Triplet state
XX -1.069508 -0.602840 -0.002739 C 2 C 1 0.304820 -0.805747 0.222950 -0.296881 (3 -1)
XX -0.000044 0.000181 -0.003738 RING 0.018972 0.146696 -1.184085 0.008384 (3 1)

XX 0.000558 -1.215921 -0.003937 C 3 C 2 0.301533 -0.794557 0.211680 -0.289992 (3 -1)
XX 1.070040 -0.601937 -0.004476 C 4 C 3 0.304817 -0.805727 0.222942 -0.296880 (3 -1)
XX 1.069425 0.603200 -0.002648 C 5 C 4 0.304822 -0.805757 0.222958 -0.296885 (3 -1)
XX -1.070131 0.602297 -0.004807 C 6 C 1 0.304816 -0.805713 0.222957 -0.296876 (3 -1)
XX -0.000645 1.216279 -0.004183 C 6 C 5 0.301524 -0.794509 0.211678 -0.289975 (3 -1)
XX -1.043288 -1.806307 -0.012973 C 7 C 2 0.284135 -0.768329 0.062616 -0.268085 (3 -1)
XX 1.044888 -1.805469 0.005060 C 8 C 3 0.284143 -0.768369 0.062608 -0.268102 (3 -1)
XX 1.043233 1.806648 -0.013014 C 9 C 5 0.284134 -0.768321 0.062621 -0.268084 (3 -1)
XX 2.161275 0.001221 -0.001943 C 9 C 8 0.265975 -0.639699 0.035705 -0.226645 (3 -1)
XX -1.044988 1.805832 0.004236 C 10 C 6 0.284143 -0.768367 0.062609 -0.268102 (3 -1)
XX -2.161367 -0.000959 -0.002040 C 10 C 7 0.265973 -0.639694 0.035658 -0.226641 (3 -1)
XX -3.278605 0.105128 -0.618803 C 12 C 11 0.266986 -0.671508 0.069491 -0.227079 (3 -1)
XX -3.274495 -0.107380 0.620656 C 13 C 11 0.266984 -0.671524 0.069389 -0.227091 (3 -1)
XX 3.278252 -0.105467 -0.618977 C 15 C 14 0.266989 -0.671535 0.069469 -0.227086 (3 -1)
XX 3.274715 0.107708 0.620354 C 16 C 14 0.266967 -0.671434 0.069395 -0.227061 (3 -1)
XX -3.793291 0.189963 1.474103 F 17 C 13 0.276272 -0.391414 0.108517 -0.411217 (3 -1)
XX -4.045699 -0.462464 1.225432 F 18 C 13 0.281517 -0.353578 0.105727 -0.423780 (3 -1)
XX -3.404933 -0.430961 1.599093 F 19 C 13 0.274939 -0.360411 0.107206 -0.409084 (3 -1)
XX -3.808575 -0.190720 -1.465797 F 20 C 12 0.276403 -0.391021 0.108317 -0.411534 (3 -1)
XX -4.050900 0.465942 -1.218643 F 21 C 12 0.281417 -0.354568 0.105723 -0.423549 (3 -1)
XX -3.413753 0.424684 -1.597822 F 22 C 12 0.274925 -0.360524 0.107127 -0.409053 (3 -1)
XX 3.807574 0.189763 -1.466597 F 23 C 15 0.276396 -0.391033 0.108322 -0.411515 (3 -1)
XX 4.050496 -0.466427 -1.218758 F 24 C 15 0.281426 -0.354462 0.105741 -0.423570 (3 -1)
XX 3.413169 -0.426090 -1.597733 F 25 C 15 0.274917 -0.360607 0.107118 -0.409033 (3 -1)
XX 3.794865 -0.189327 1.473095 F 26 C 16 0.276292 -0.391367 0.108485 -0.411264 (3 -1)
XX 4.045731 0.463822 1.224789 F 27 C 16 0.281496 -0.353790 0.105714 -0.423732 (3 -1)
XX 3.405338 0.430658 1.598982 F 28 C 16 0.274946 -0.360340 0.107173 -0.409100 (3 -1)
XX 1.612630 2.802485 -0.031337 N 29 C 9 0.463922 0.326142 0.033096 -0.827073 (3 -1)
XX -1.614859 2.801395 0.021284 N 30 C 10 0.463927 0.326284 0.033143 -0.827079 (3 -1)
XX -1.612593 -2.802193 -0.031428 N 31 C 7 0.463922 0.326153 0.033091 -0.827073 (3 -1)

XX 1.614776 -2.801009 0.022943 N 32 C 8 0.463928 0.326289 0.033140 -0.827080 (3 -1)

M4e Singlet state
XX -0.984834 -0.663928 0.861400 C 2 C 1 0.257331 -0.607764 0.063771 -0.213605 (3 -1)
XX 0.001342 0.006156 1.053831 RING 0.020989 0.153234 -1.123675 0.008012 (3 1)
XX 0.071842 -1.210163 1.163621 C 3 C 2 0.330107 -0.909416 0.360608 -0.350127 (3 -1)
XX 1.064913 -0.555765 0.874596 C 4 C 3 0.256597 -0.603683 0.063409 -0.212623 (3 -1)
XX 0.988854 0.674115 0.859458 C 5 C 4 0.257276 -0.607568 0.063474 -0.213526 (3 -1)
XX -1.061646 0.566348 0.866134 C 6 C 1 0.256422 -0.602781 0.063814 -0.212350 (3 -1)
XX -0.068913 1.223018 1.152950 C 6 C 5 0.329862 -0.907971 0.360375 -0.349605 (3 -1)
XX -0.948551 -1.747897 1.464148 C 7 C 2 0.286161 -0.773288 0.070946 -0.271414 (3 -1)
XX 1.133516 -1.652852 1.470740 C 8 C 3 0.286375 -0.773111 0.071046 -0.271993 (3 -1)
XX 0.950163 1.762878 1.454588 C 9 C 5 0.286214 -0.773449 0.071101 -0.271462 (3 -1)
XX -1.130757 1.670984 1.449949 C 10 C 6 0.286339 -0.772846 0.071106 -0.271905 (3 -1)
XX -1.852478 -0.090326 0.132505 C 11 C 1 0.332547 -0.908325 0.373553 -0.357562 (3 -1)
XX -1.597888 -1.457731 0.071360 RING 0.012786 0.071604 -1.606119 0.003246 (3 1)
XX -1.622992 1.372582 -0.019569 RING 0.011523 0.059548 -1.863019 0.002674 (3 1)
XX -2.697144 0.502403 -0.548218 C 12 C 11 0.257830 -0.641342 0.031506 -0.212405 (3 -1)
XX -2.563350 -0.723458 -0.672711 C 13 C 11 0.261143 -0.655861 0.037269 -0.217352 (3 -1)
XX 1.855478 0.092658 0.134439 C 14 C 4 0.332608 -0.908504 0.374314 -0.357739 (3 -1)
XX 1.617199 1.464945 0.076898 RING 0.012705 0.071145 -1.607317 0.003231 (3 1)
XX 1.622312 -1.361678 -0.006694 RING 0.011697 0.060811 -1.823873 0.002732 (3 1)
XX 2.693813 -0.509282 -0.546135 C 15 C 14 0.257792 -0.641140 0.031603 -0.212352 (3 -1)
XX 2.568318 0.716391 -0.676210 C 16 C 14 0.261207 -0.656236 0.037330 -0.217454 (3 -1)
XX 3.094426 0.147787 -1.742633 RING 0.013808 0.080963 -1.653199 0.002887 (3 1)
XX 2.719251 1.323784 -1.511464 F 17 C 16 0.277734 -0.381831 0.105645 -0.415193 (3 -1)
XX 1.685155 2.280157 0.527180 F 18 C 9 0.015896 0.070067 2.208707 0.002074 (3 -1)
XX 2.597521 1.742539 -0.919697 F 18 C 16 0.278528 -0.324042 0.104041 -0.417300 (3 -1)
XX 3.258864 1.426370 -1.009879 F 19 C 16 0.280162 -0.369525 0.106209 -0.420786 (3 -1)
XX 2.813673 -1.906632 1.007850 F 20 C 8 0.007639 0.031987 0.412901 0.001394 (3 -1)

XX 3.374853 -1.300094 -0.449466 F 20 C 15 0.279069 -0.375039 0.105924 -0.418150 (3 -1)
XX 1.509461 -1.684812 0.099079 F 21 C 3 0.012307 0.052747 0.676657 0.001697 (3 -1)
XX 2.776659 -1.518842 -0.834003 F 21 C 15 0.274518 -0.363667 0.107405 -0.407968 (3 -1)
XX 2.495906 -1.872480 0.616501 RING 0.006701 0.033735 -1.394474 0.001863 (3 1)
XX 3.301265 -1.114847 -1.159147 F 22 C 15 0.283065 -0.340183 0.102591 -0.427478 (3 -1)
XX 3.140448 0.126982 -2.101450 F 22 F 17 0.014864 0.067877 0.321819 0.000744 (3 -1)
XX -3.106794 -0.157668 -1.720592 RING 0.014014 0.082916 -1.599507 0.002992 (3 1)
XX -2.723932 -1.332461 -1.505113 F 23 C 13 0.277904 -0.381372 0.105387 -0.415606 (3 -1)
XX -1.649268 -2.253799 0.518535 F 24 C 7 0.015713 0.069921 3.046410 0.002189 (3 -1)
XX -2.578237 -1.749559 -0.917868 F 24 C 13 0.278344 -0.325085 0.104033 -0.416892 (3 -1)
XX -3.248252 -1.446234 -0.989951 F 25 C 13 0.280099 -0.370104 0.106310 -0.420624 (3 -1)
XX -2.810035 1.917790 0.998745 F 26 C 10 0.007905 0.033002 0.392304 0.001405 (3 -1)
XX -3.378859 1.291841 -0.448188 F 26 C 12 0.279003 -0.375155 0.105901 -0.418004 (3 -1)
XX -1.517864 1.686288 0.079991 F 27 C 6 0.012052 0.051939 0.740960 0.001721 (3 -1)
XX -2.787628 1.508401 -0.844927 F 27 C 12 0.274534 -0.364220 0.107450 -0.407986 (3 -1)
XX -3.168512 -0.139469 -2.099772 F 27 F 23 0.015327 0.069450 0.266548 0.000678 (3 -1)
XX -3.316993 1.100125 -1.156916 F 28 C 12 0.283217 -0.339105 0.102349 -0.427808 (3 -1)
XX -1.753606 2.465250 1.978671 N 29 C 10 0.463167 0.327982 0.037827 -0.825315 (3 -1)
XX 1.514556 2.580799 2.013269 N 30 C 9 0.463084 0.325329 0.033603 -0.825298 (3 -1)
XX 1.757793 -2.437784 2.011696 N 31 C 8 0.463159 0.327425 0.037546 -0.825313 (3 -1)
XX -1.516925 -2.561669 2.025266 N 32 C 7 0.463085 0.325217 0.033428 -0.825300 (3 -1)

M5a Triplet state
XX -0.015982 -0.148429 0.653414 C 2 C 1 0.292752 -0.755743 0.208511 -0.274299 (3 -1)
XX 1.231188 0.018801 0.738662 RING 0.017490 0.133648 -1.175667 0.007682 (3 1)
XX 0.636622 -0.305785 1.737532 C 3 C 2 0.294008 -0.769884 0.209633 -0.273880 (3 -1)
XX 1.895203 -0.135671 1.805032 C 4 C 3 0.294078 -0.760670 0.212512 -0.276703 (3 -1)
XX 2.478370 0.185969 0.823935 C 5 C 4 0.292767 -0.755824 0.208524 -0.274329 (3 -1)
XX 0.567188 0.173256 -0.327712 C 6 C 1 0.294058 -0.760569 0.212493 -0.276665 (3 -1)
XX 1.825780 0.343381 -0.260189 C 6 C 5 0.294000 -0.769845 0.209615 -0.273865 (3 -1)

```
XX -1.417901 -0.335274  0.718099 RING         0.012314  0.072159 -1.329042  0.004318 (3 1)
XX -0.388041 -0.330651  1.641422 N   7 C   2  0.318283 -0.950781  0.129710 -0.494609 (3 -1)
XX  1.221265 -0.467306  2.575355 N   8 C   3  0.317204 -0.977534  0.118081 -0.486485 (3 -1)
XX  2.536200 -0.456940  3.006720 RING         0.011909  0.071775 -1.313969  0.004322 (3 1)
XX  3.880372  0.372808  0.759312 RING         0.012316  0.072161 -1.329173  0.004318 (3 1)
XX  2.850434  0.368187 -0.164037 N   9 C   5  0.318297 -0.950682  0.129710 -0.494669 (3 -1)
XX -0.659515 -0.021532 -0.397796 C  10 C   1  0.279493 -0.674600  0.233755 -0.251569 (3 -1)
XX -0.073764  0.494495 -1.529403 RING         0.011907  0.071765 -1.313943  0.004322 (3 1)
XX  1.241137  0.505022 -1.097981 C  11 N   9  0.317203 -0.977480  0.118111 -0.486491 (3 -1)
XX -1.376636  0.289925 -1.320006 C  11 C  10  0.286804 -0.757272  0.195486 -0.284031 (3 -1)
XX -2.155295 -0.636808  0.920536 C  12 N   7  0.021401  0.070566  0.268105  0.000887 (3 -1)
XX -1.795218 -0.388292 -0.562147 C  12 C  10  0.287139 -0.757779  0.195773 -0.284186 (3 -1)
XX  3.121902  0.059026  1.875119 C  13 C   4  0.279490 -0.674589  0.233702 -0.251563 (3 -1)
XX  2.733625 -0.752468  3.790069 C  14 N   8  0.023400  0.071708  0.271260  0.000396 (3 -1)
XX  3.839099 -0.252832  2.797101 C  14 C  13  0.286805 -0.757271  0.195519 -0.284033 (3 -1)
XX  4.617465  0.674630  0.557107 C  15 N   9  0.021392  0.070550  0.268042  0.000889 (3 -1)
XX  4.257452  0.426056  2.039648 C  15 C  13  0.287138 -0.757770  0.195812 -0.284187 (3 -1)
XX -1.091655 -0.221934  2.857171 H  16 N   7  0.342041 -1.824391  0.047301 -0.508730 (3 -1)
XX -1.812665 -0.461757  1.792815 H  17 N   7  0.341570 -1.846043  0.041291 -0.511078 (3 -1)
XX  0.608942 -1.197613  3.596738 H  18 N   8  0.341221 -1.815425  0.045115 -0.506850 (3 -1)
XX  1.789970 -0.741789  3.854036 H  19 N   8  0.341197 -1.841104  0.038917 -0.510420 (3 -1)
XX  3.554144  0.259663 -1.379800 H  20 N   9  0.342051 -1.824478  0.047303 -0.508751 (3 -1)
XX  4.275073  0.499331 -0.315292 H  21 N   9  0.341569 -1.846045  0.041296 -0.511074 (3 -1)
XX -0.271107  0.789830 -2.313068 N  22 C  11  0.023406  0.071720  0.271526  0.000395 (3 -1)
XX  0.672515  0.779642 -2.376720 H  23 N  22  0.341192 -1.841081  0.038917 -0.510414 (3 -1)
XX  1.853478  1.235539 -2.119218 H  24 N  22  0.341222 -1.815425  0.045117 -0.506850 (3 -1)
XX  4.028703 -0.835886  3.785524 N  25 C  14  0.458375  0.117323  0.043672 -0.821042 (3 -1)
XX  5.173631  1.053016  1.697702 N  26 C  15  0.458242  0.125187  0.049458 -0.820414 (3 -1)
XX -2.711709 -1.014651 -0.219946 N  27 C  12  0.458245  0.125214  0.049447 -0.820420 (3 -1)
XX -1.566124  0.872342 -2.308830 N  28 C  11  0.458375  0.117304  0.043652 -0.821043 (3 -1)
```

M5a Singlet state

XX -0.002546 0.110227 0.695533 C 2 C 1 0.267225 -0.664304 0.075958 -0.228388 (3 -1)
XX 1.208527 -0.150326 0.708235 RING 0.017350 0.133210 -1.155596 0.007664 (3 1)
XX 0.727919 0.306694 1.745522 C 3 C 2 0.312252 -0.847709 0.324018 -0.314542 (3 -1)
XX 1.908491 0.198305 1.692625 C 4 C 3 0.297214 -0.767800 0.248542 -0.283427 (3 -1)
XX 2.433917 -0.331530 0.689818 C 5 C 4 0.267189 -0.664112 0.075962 -0.228328 (3 -1)
XX 0.535706 -0.348895 -0.334967 C 6 C 1 0.297152 -0.767498 0.248488 -0.283312 (3 -1)
XX 1.678148 -0.668536 -0.305082 C 6 C 5 0.312311 -0.848032 0.324161 -0.314671 (3 -1)
XX -1.357843 0.459131 0.794367 RING 0.011293 0.066783 -1.321604 0.003999 (3 1)
XX -0.312681 0.343904 1.693253 N 7 C 2 0.333267 -0.935083 0.106078 -0.540820 (3 -1)
XX 0.131199 0.580453 2.822007 RING 0.022298 0.138518 -1.644892 0.006866 (3 1)
XX 1.334528 0.510055 2.586455 N 8 C 3 0.284996 -0.845614 0.036809 -0.342472 (3 -1)
XX 2.660090 0.765136 2.774146 RING 0.013155 0.062090 -1.557372 0.002980 (3 1)
XX 3.808892 -0.569819 0.548252 RING 0.011305 0.066894 -1.321155 0.004007 (3 1)
XX 2.679271 -0.924950 -0.166744 N 9 C 5 0.333275 -0.935154 0.106106 -0.540841 (3 -1)
XX 2.197685 -1.374158 -1.212402 RING 0.022287 0.138390 -1.646183 0.006860 (3 1)
XX -0.131120 -0.447037 -1.600136 RING 0.013157 0.062111 -1.557182 0.002982 (3 1)
XX 1.066615 -0.900520 -1.134839 N 10 C 6 0.284943 -0.845214 0.036780 -0.342311 (3 -1)
XX -0.635281 0.098498 -0.380120 C 11 C 1 0.300341 -0.770267 0.269220 -0.289508 (3 -1)
XX -2.159746 0.590383 1.100509 C 12 N 7 0.021201 0.070458 0.335441 0.001206 (3 -1)
XX -1.788211 0.446072 -0.478877 C 12 C 11 0.284898 -0.757532 0.151933 -0.278127 (3 -1)
XX -0.133071 -0.541775 -2.088231 C 13 N 10 0.014784 0.051100 0.286351 0.001760 (3 -1)
XX -1.245512 0.217989 -1.417695 C 13 C 11 0.282373 -0.743513 0.143121 -0.270606 (3 -1)
XX 3.156717 0.179176 1.569807 C 14 C 4 0.300281 -0.769945 0.269158 -0.289393 (3 -1)
XX 2.691358 1.022382 3.197906 C 15 N 8 0.014776 0.051089 0.288020 0.001761 (3 -1)
XX 3.893688 0.762134 2.331571 C 15 C 14 0.282375 -0.743506 0.143188 -0.270616 (3 -1)
XX 4.570288 -0.926793 0.331074 C 16 N 9 0.021250 0.070570 0.334353 0.001198 (3 -1)
XX 4.363896 0.131755 1.550894 C 16 C 14 0.284901 -0.757534 0.151994 -0.278137 (3 -1)
XX 2.281734 -1.603233 -1.534910 H 17 N 9 0.024982 0.087441 0.313934 0.001592 (3 -1)

XX 1.089409 -0.830897 -2.485286 H 17 N 10 0.338353 -1.769114 0.048426 -0.498259 (3 -1)
XX 0.448408 -1.748449 -1.974966 H 18 N 10 0.340687 -1.781255 0.048651 -0.501722 (3 -1)
XX 3.121941 -1.727671 -1.227771 H 19 N 9 0.336779 -1.824166 0.039906 -0.503691 (3 -1)
XX 4.034520 -1.436814 -0.274289 H 20 N 9 0.342983 -1.858144 0.039748 -0.514137 (3 -1)
XX 0.020620 0.662287 3.202876 H 21 N 7 0.025007 0.087476 0.312679 0.001586 (3 -1)
XX 1.762140 0.296012 3.842594 H 21 N 8 0.340696 -1.781339 0.048626 -0.501742 (3 -1)
XX 1.495377 1.457560 3.537968 H 22 N 8 0.338329 -1.768938 0.048408 -0.498212 (3 -1)
XX -0.847689 0.629575 2.957321 H 23 N 7 0.336752 -1.823978 0.039902 -0.503644 (3 -1)
XX -1.736372 0.477538 1.950524 H 24 N 7 0.342956 -1.858029 0.039735 -0.514090 (3 -1)
XX 4.103179 1.497194 3.201445 N 25 C 15 0.460965 0.209826 0.036839 -0.824159 (3 -1)
XX 5.370943 -0.242085 1.107534 N 26 C 16 0.460661 0.176202 0.036531 -0.824542 (3 -1)
XX -2.800459 0.790326 -0.023567 N 27 C 12 0.460663 0.176261 0.036503 -0.824546 (3 -1)

M5b Triplet state
XX -0.005269 -0.011069 0.718442 C 2 C 1 0.300666 -0.780841 0.212519 -0.289353 (3 -1)
XX 1.214676 -0.013883 0.679127 RING 0.019243 0.148560 -1.180782 0.008464 (3 1)
XX 0.611957 -0.003726 1.757762 C 3 C 2 0.308043 -0.818654 0.208619 -0.302083 (3 -1)
XX 1.820558 -0.006180 1.738722 C 4 C 3 0.300626 -0.780634 0.212452 -0.289276 (3 -1)
XX 2.434336 -0.040339 0.639883 C 5 C 4 0.300652 -0.780783 0.212461 -0.289326 (3 -1)
XX 0.609076 0.002215 -0.380537 C 6 C 1 0.300631 -0.780645 0.212505 -0.289287 (3 -1)
XX 1.817378 -0.024232 -0.399517 C 6 C 5 0.308043 -0.818651 0.208633 -0.302084 (3 -1)
XX -0.664882 -0.067925 1.817417 C 7 C 2 0.250743 -0.585302 0.031514 -0.204985 (3 -1)
XX 1.231163 0.071501 2.875491 C 8 C 3 0.250804 -0.585585 0.031515 -0.205083 (3 -1)
XX 3.092326 -0.119134 -0.458768 C 9 C 5 0.250728 -0.585231 0.031506 -0.204964 (3 -1)
XX 3.046885 -0.032481 1.703163 C 14 C 4 0.260757 -0.624592 0.082075 -0.223591 (3 -1)
XX 1.200005 0.059354 -1.517680 C 10 C 6 0.250810 -0.585617 0.031514 -0.205091 (3 -1)
XX -0.617551 0.005477 -0.344891 C 11 C 1 0.260722 -0.624458 0.081881 -0.223536 (3 -1)
XX -1.444306 -0.446964 -1.179426 C 12 C 11 0.294023 -0.798574 0.143760 -0.291769 (3 -1)
XX -1.753737 0.479387 -0.607529 C 13 C 11 0.294020 -0.798562 0.143754 -0.291764 (3 -1)
XX 3.862219 -0.499577 2.540787 C 15 C 14 0.294019 -0.798565 0.143745 -0.291761 (3 -1)

XX 4.194405 0.414996 1.962750 C 16 C 14 0.294017 -0.798556 0.143741 -0.291757 (3 -1)
XX 1.726195 0.496640 -2.466534 H 17 C 10 0.278825 -0.965024 0.011484 -0.286127 (3 -1)
XX 1.260382 -0.504639 -2.549826 H 18 C 10 0.274207 -0.934239 0.012227 -0.277881 (3 -1)
XX 0.626328 0.400050 -2.482370 H 19 C 10 0.280123 -0.973495 0.010849 -0.288558 (3 -1)
XX 3.621503 -0.579053 -1.395135 H 20 C 9 0.278789 -0.964776 0.011482 -0.286072 (3 -1)
XX 3.940855 0.428523 -1.064654 H 21 C 9 0.274219 -0.934307 0.012215 -0.277901 (3 -1)
XX 4.213464 -0.464451 -0.470120 H 22 C 9 0.280167 -0.973828 0.010812 -0.288628 (3 -1)
XX -1.204858 -0.509953 2.756217 H 23 C 7 0.278789 -0.964781 0.011482 -0.286073 (3 -1)
XX -1.500066 0.503135 2.420259 H 24 C 7 0.274214 -0.934261 0.012216 -0.277890 (3 -1)
XX -1.793934 -0.386257 1.830517 H 25 C 7 0.280170 -0.973851 0.010812 -0.288637 (3 -1)
XX 1.812889 0.403678 3.838339 H 26 C 8 0.280125 -0.973509 0.010843 -0.288557 (3 -1)
XX 0.715665 0.526778 3.821754 H 27 C 8 0.278818 -0.964973 0.011484 -0.286114 (3 -1)
XX 1.157061 -0.485002 3.910781 H 28 C 8 0.274208 -0.934254 0.012222 -0.277884 (3 -1)
XX 4.092023 -1.310139 3.318043 N 29 C 15 0.458496 0.156819 0.059709 -0.819529 (3 -1)
XX 4.989004 1.215393 1.757981 N 30 C 16 0.458495 0.156836 0.059730 -0.819526 (3 -1)
XX -1.694054 -1.256798 -1.951259 N 31 C 12 0.458495 0.156776 0.059723 -0.819529 (3 -1)
XX -2.528226 1.300572 -0.408119 N 32 C 13 0.458493 0.156791 0.059739 -0.819523 (3 -1)

M5b Singlet state
XX -0.053327 0.072322 0.803056 C 2 C 1 0.271688 -0.670436 0.087520 -0.237398 (3 -1)
XX 1.117485 0.355306 0.723102 RING 0.019751 0.149380 -1.147108 0.008142 (3 1)
XX 0.596652 -0.011698 1.808024 C 3 C 2 0.330319 -0.910391 0.322629 -0.349637 (3 -1)
XX 1.788078 0.036788 1.674980 C 4 C 3 0.271700 -0.670493 0.087493 -0.237414 (3 -1)
XX 2.301378 0.563959 0.612704 C 5 C 4 0.271756 -0.670786 0.087486 -0.237510 (3 -1)
XX 0.460024 0.599423 -0.259423 C 6 C 1 0.271712 -0.670539 0.087588 -0.237438 (3 -1)
XX 1.588467 1.006755 -0.244697 C 6 C 5 0.330299 -0.910249 0.322694 -0.349597 (3 -1)
XX -1.410624 -0.585269 0.877446 RING 0.010411 0.052156 -1.465447 0.002993 (3 1)
XX -0.677882 -0.081831 1.930857 C 7 C 2 0.250421 -0.583466 0.035525 -0.205982 (3 -1)
XX -0.060090 0.132179 3.203594 RING 0.011672 0.060286 -2.033560 0.003726 (3 1)
XX 1.306268 -0.119160 2.870626 C 8 C 3 0.250420 -0.583486 0.035491 -0.205982 (3 -1)

XX	2.803057	1.417654	-0.227231	C	9	C	5	0.250377	-0.583271	0.035499	-0.205913	(3 -1)
XX	-0.208023	0.650259	-1.612255	RING				0.010412	0.052186	-1.462773	0.002997	(3 1)
XX	2.002766	2.247611	-1.068830	RING				0.011709	0.060615	-2.013402	0.003745	(3 1)
XX	0.819370	1.454870	-1.167721	C	10	C	6	0.250403	-0.583388	0.035504	-0.205953	(3 -1)
XX	-0.550473	-0.138689	-0.327788	C	11	C	1	0.315800	-0.846340	0.294856	-0.321063	(3 -1)
XX	-0.367337	0.826127	-2.010687	C	12	C	10	0.011328	0.046912	0.511776	0.002520	(3 -1)
XX	-1.044790	-0.433812	-1.396527	C	12	C	11	0.281412	-0.748202	0.107784	-0.267263	(3 -1)
XX	-1.833867	-0.683865	1.031687	C	13	C	7	0.011313	0.046851	0.507398	0.002513	(3 -1)
XX	-1.491036	-0.892386	-0.472321	C	13	C	11	0.281410	-0.748189	0.107789	-0.267259	(3 -1)
XX	2.970383	-0.207814	1.339002	C	14	C	4	0.315825	-0.846434	0.294989	-0.321113	(3 -1)
XX	3.772845	0.573069	0.270530	RING				0.010393	0.052004	-1.467739	0.002984	(3 1)
XX	2.569847	-0.661166	2.761722	RING				0.010412	0.052173	-1.458954	0.002999	(3 1)
XX	2.721699	-0.767997	3.188707	C	15	C	8	0.011335	0.046955	0.516343	0.002532	(3 -1)
XX	3.652660	-0.994566	1.962357	C	15	C	14	0.281414	-0.748219	0.107838	-0.267262	(3 -1)
XX	4.184417	0.737079	0.144647	C	16	C	4	0.011287	0.046725	0.507340	0.002510	(3 -1)
XX	4.099899	-0.535253	1.038686	C	16	C	14	0.281426	-0.748292	0.107824	-0.267287	(3 -1)
XX	3.118472	2.448090	-0.651264	H	17	C	9	0.277097	-0.951993	0.012699	-0.283242	(3 -1)
XX	3.839079	1.909160	-0.003618	H	18	C	9	0.281012	-0.983930	0.012568	-0.290106	(3 -1)
XX	3.643833	1.551897	-1.038410	H	19	C	9	0.276265	-0.955036	0.012542	-0.281985	(3 -1)
XX	2.004064	0.018518	3.797275	H	20	C	8	0.281121	-0.984756	0.012493	-0.290315	(3 -1)
XX	0.914671	0.186735	3.916466	H	21	C	8	0.277024	-0.951475	0.012667	-0.283115	(3 -1)
XX	1.302872	-0.844675	3.796023	H	22	C	8	0.276271	-0.955038	0.012525	-0.281983	(3 -1)
XX	-1.227275	0.219542	2.904853	H	23	C	7	0.277107	-0.952108	0.012694	-0.283258	(3 -1)
XX	-0.203553	0.282459	3.514707	H	23	H	21	0.012301	0.051070	0.942072	0.002876	(3 -1)
XX	-1.830895	0.097361	1.982736	H	24	C	7	0.281020	-0.983959	0.012561	-0.290127	(3 -1)
XX	-1.418153	-0.795276	2.501678	H	25	C	7	0.276270	-0.955088	0.012537	-0.282004	(3 -1)
XX	2.059601	2.607613	-1.174089	H	26	C	9	0.012370	0.051269	0.919800	0.002874	(3 -1)
XX	0.981054	2.487215	-1.666880	H	26	C	10	0.277160	-0.952461	0.012687	-0.283376	(3 -1)
XX	0.918579	1.601308	-2.330214	H	27	C	10	0.276273	-0.955067	0.012539	-0.282004	(3 -1)
XX	0.006391	1.981622	-1.820609	H	28	C	10	0.281054	-0.984226	0.012555	-0.290193	(3 -1)

XX 5.019036 -0.532710 0.336627 N 29 C 16 0.462662 0.270315 0.037316 -0.826039 (3 -1)
XX 3.821199 -1.762925 2.810269 N 30 C 15 0.462658 0.270339 0.037289 -0.826029 (3 -1)
XX -1.084807 -0.412736 -2.552247 N 31 C 12 0.462658 0.270261 0.037325 -0.826033 (3 -1)
XX -2.279613 -1.640741 -0.077565 N 32 C 13 0.462661 0.270355 0.037320 -0.826037 (3 -1)

M5c Triplet state
XX 0.003248 0.000197 0.727600 C 2 C 1 0.304957 -0.817550 0.173397 -0.297015 (3 -1)
XX 1.222888 -0.000000 0.744290 RING 0.020015 0.156627 -1.193033 0.008807 (3 1)
XX 0.593791 0.000383 1.777708 C 3 C 2 0.316663 -0.873905 0.209138 -0.320438 (3 -1)
XX 1.797810 0.000197 1.820056 C 4 C 3 0.304940 -0.817450 0.173377 -0.296980 (3 -1)
XX 2.442528 -0.000197 0.760980 C 5 C 4 0.304956 -0.817547 0.173397 -0.297014 (3 -1)
XX 0.647966 -0.000198 -0.331476 C 6 C 1 0.304940 -0.817449 0.173377 -0.296980 (3 -1)
XX 1.851985 -0.000383 -0.289128 C 6 C 5 0.316662 -0.873902 0.209138 -0.320437 (3 -1)
XX -0.605526 0.000575 1.756202 H 7 C 2 0.288131 -1.066381 0.014047 -0.304763 (3 -1)
XX 1.163695 0.000572 2.833231 H 8 C 3 0.288140 -1.066462 0.014048 -0.304784 (3 -1)
XX 3.051302 -0.000575 -0.267623 H 9 C 5 0.288131 -1.066386 0.014047 -0.304765 (3 -1)
XX 1.282081 -0.000572 -1.344651 H 10 C 6 0.288140 -1.066462 0.014048 -0.304784 (3 -1)
XX -0.585092 -0.000001 -0.356423 C 11 C 1 0.272859 -0.675175 0.132910 -0.241311 (3 -1)
XX -1.231281 -0.000228 -1.416703 C 12 C 11 0.293542 -0.798575 0.135763 -0.289397 (3 -1)
XX -1.823704 0.000236 -0.443965 C 13 C 11 0.293545 -0.798586 0.135766 -0.289401 (3 -1)
XX 3.030867 0.000000 1.845003 C 14 C 4 0.272859 -0.675176 0.132910 -0.241312 (3 -1)
XX 3.677057 0.000214 2.905282 C 15 C 14 0.293542 -0.798575 0.135763 -0.289397 (3 -1)
XX 4.269480 -0.000223 1.932545 C 16 C 14 0.293545 -0.798587 0.135766 -0.289401 (3 -1)
XX -2.836458 0.000645 0.088080 N 17 C 13 0.459365 0.180311 0.058736 -0.820755 (3 -1)
XX -1.223265 -0.000620 -2.560683 N 18 C 12 0.459366 0.180329 0.058726 -0.820758 (3 -1)
XX 3.669041 0.000584 4.049263 N 19 C 15 0.459365 0.180317 0.058726 -0.820757 (3 -1)
XX 5.282234 -0.000609 1.400500 N 20 C 16 0.459364 0.180295 0.058737 -0.820753 (3 -1)

M5c Singlet state
XX -0.000855 0.000280 0.740983 C 2 C 1 0.286249 -0.744345 0.097472 -0.262043 (3 -1)

XX 1.222888 0.000004 0.744290 RING 0.019129 0.147888 -1.185571 0.008374 (3 1)
XX 0.582371 0.000429 1.796406 C 3 C 2 0.335290 -0.961689 0.279192 -0.360393 (3 -1)
XX 1.787785 0.000165 1.829812 C 4 C 3 0.286227 -0.744224 0.097459 -0.262002 (3 -1)
XX 2.446632 -0.000272 0.747597 C 5 C 4 0.286250 -0.744347 0.097472 -0.262044 (3 -1)
XX 0.657991 -0.000157 -0.341232 C 6 C 1 0.286228 -0.744227 0.097459 -0.262002 (3 -1)
XX 1.863405 -0.000422 -0.307826 C 6 C 5 0.335289 -0.961684 0.279191 -0.360392 (3 -1)
XX -0.610389 0.000694 1.779138 H 7 C 2 0.288781 -1.076741 0.013359 -0.306457 (3 -1)
XX 1.145543 0.000588 2.848030 H 8 C 3 0.288792 -1.076834 0.013357 -0.306481 (3 -1)
XX 3.056165 -0.000687 -0.290557 H 9 C 5 0.288781 -1.076745 0.013359 -0.306458 (3 -1)
XX 1.300234 -0.000579 -1.359451 H 10 C 6 0.288792 -1.076834 0.013357 -0.306481 (3 -1)
XX -0.575692 0.000111 -0.350718 C 11 C 1 0.314005 -0.848522 0.260395 -0.316150 (3 -1)
XX -1.187923 -0.000110 -1.394458 C 12 C 11 0.284876 -0.765165 0.105145 -0.272315 (3 -1)
XX -1.784015 0.000272 -0.415717 C 13 C 11 0.284872 -0.765147 0.105138 -0.272308 (3 -1)
XX 3.021468 -0.000104 1.839297 C 14 C 4 0.314005 -0.848522 0.260395 -0.316150 (3 -1)
XX 3.633700 0.000112 2.883037 C 15 C 14 0.284876 -0.765165 0.105145 -0.272315 (3 -1)
XX 4.229791 -0.000275 1.904296 C 16 C 14 0.284873 -0.765150 0.105138 -0.272309 (3 -1)
XX -2.810504 0.000527 0.105000 N 17 C 13 0.462378 0.265044 0.039023 -0.825350 (3 -1)
XX -1.196332 -0.000568 -2.545439 N 18 C 12 0.462379 0.265018 0.039019 -0.825352 (3 -1)
XX 3.642108 0.000556 4.034019 N 19 C 15 0.462379 0.265018 0.039019 -0.825352 (3 -1)
XX 5.256280 -0.000536 1.383579 N 20 C 16 0.462379 0.265043 0.039023 -0.825350 (3 -1)

M5d Triplet state
XX -1.056705 0.609444 0.048441 C 2 C 1 0.302031 -0.780959 0.219816 -0.291602 (3 -1)
XX -0.000230 -0.000074 0.000262 RING 0.019672 0.150675 -1.176183 0.008498 (3 1)
XX -0.000261 1.227115 -0.000282 C 3 C 2 0.303453 -0.789697 0.211141 -0.294104 (3 -1)
XX 1.056216 0.609523 -0.047792 C 4 C 3 0.302042 -0.781053 0.219694 -0.291617 (3 -1)
XX 1.056233 -0.609550 0.048704 C 5 C 4 0.301992 -0.780825 0.219565 -0.291522 (3 -1)
XX -1.056625 -0.609687 -0.047587 C 6 C 1 0.301954 -0.780619 0.219554 -0.291444 (3 -1)
XX -0.000156 -1.227290 0.000198 C 6 C 5 0.303532 -0.790086 0.211290 -0.294253 (3 -1)
XX -2.183040 1.151288 0.527430 RING 0.015276 0.085367 -1.460764 0.003705 (3 1)

XX -1.069478 1.841011 0.231118 C   7 C   2 0.255896 -0.635032 0.029689 -0.209491 (3 -1)
XX 0.173530 2.665359 0.221199 RING   0.010674 0.065154 -1.141426 0.003240 (3 1)
XX -0.167073 2.664566 -0.218434 RING   0.010651 0.065259 -1.122284 0.003256 (3 1)
XX 1.068957 1.840675 -0.233149 C   8 C   3 0.255875 -0.634920 0.029688 -0.209457 (3 -1)
XX 2.182153 1.149846 -0.526472 RING   0.015315 0.085636 -1.460525 0.003713 (3 1)
XX -0.007173 2.660949 -0.012229 CAGE   0.010592 0.068236 -0.952315 0.003539 (3 3)
XX 2.182856 -1.150483 0.530006 RING   0.015261 0.085199 -1.462224 0.003696 (3 1)
XX 1.068995 -1.840953 0.232633 C   9 C   5 0.255882 -0.634967 0.029666 -0.209470 (3 -1)
XX -0.170507 -2.664572 0.217994 RING   0.010683 0.065337 -1.133437 0.003252 (3 1)
XX 2.150733 0.000110 0.001421 C   9 C   8 0.255670 -0.599645 0.054168 -0.211048 (3 -1)
XX -2.183341 -1.150017 -0.528031 RING   0.015274 0.085288 -1.462530 0.003698 (3 1)
XX 0.169419 -2.663962 -0.219801 RING   0.010678 0.065358 -1.128901 0.003255 (3 1)
XX -1.068998 -1.840934 -0.233388 C 10 C   6 0.255874 -0.634920 0.029688 -0.209458 (3 -1)
XX -2.151189 -0.000452 0.002043 C 10 C   7 0.255661 -0.599633 0.053978 -0.211036 (3 -1)
XX 0.001787 -2.660210 -0.004138 CAGE   0.010611 0.068397 -0.952387 0.003545 (3 3)
XX -2.692697 1.141942 0.628673 C 12 C   7 0.018386 0.069317 0.115989 0.000764 (3 -1)
XX -2.617625 -1.695531 0.576674 RING   0.005829 0.026970 -2.202723 0.001500 (3 1)
XX -3.246758 -0.254249 0.524834 C 12 C 11 0.296407 -0.804766 0.152355 -0.292731 (3 -1)
XX -2.620694 1.693098 -0.574473 RING   0.005834 0.027031 -2.180807 0.001504 (3 1)
XX -2.691692 -1.140878 -0.627789 C 13 C 10 0.018347 0.069206 0.118843 0.000770 (3 -1)
XX -3.249594 0.252019 -0.516279 C 13 C 11 0.296406 -0.804794 0.152191 -0.292710 (3 -1)
XX 2.617691 1.696098 0.574733 RING   0.005841 0.027071 -2.171456 0.001506 (3 1)
XX 2.690462 -1.141314 0.631045 C 15 C   9 0.018325 0.069114 0.118739 0.000771 (3 -1)
XX 3.247066 0.254900 0.522337 C 15 C 14 0.296428 -0.804912 0.152257 -0.292765 (3 -1)
XX 2.693081 1.140339 -0.626301 C 16 C   8 0.018450 0.069557 0.115993 0.000759 (3 -1)
XX 2.616422 -1.689530 -0.565110 RING   0.005885 0.027310 -2.125017 0.001519 (3 1)
XX 3.248283 -0.253866 -0.517574 C 16 C 14 0.296422 -0.804870 0.152239 -0.292747 (3 -1)
XX -1.207177 2.810600 0.630161 F 17 C   7 0.280541 -0.344851 0.104848 -0.421925 (3 -1)
XX 0.532807 3.099939 0.505825 RING   0.010878 0.060777 -2.082459 0.002228 (3 1)
XX -1.640899 2.714502 0.037157 F 18 C   7 0.281009 -0.362323 0.104653 -0.422526 (3 -1)

XX -0.613600 3.183531 -0.795740 F 18 C 8 0.011167 0.053426 0.712341 0.001107 (3 -1)
XX -2.763591 1.820253 -0.849494 F 18 C 13 0.005996 0.026086 1.136731 0.001332 (3 -1)
XX -0.535306 3.098879 -0.517609 RING 0.010815 0.060378 -2.104522 0.002214 (3 1)
XX -1.824833 2.419252 0.689961 F 19 C 7 0.273358 -0.361633 0.107569 -0.405702 (3 -1)
XX -1.205885 -2.808943 -0.636414 F 20 C 10 0.280474 -0.345419 0.104868 -0.421774 (3 -1)
XX -0.614209 -3.187478 0.787462 F 21 C 9 0.011211 0.053625 0.717038 0.001106 (3 -1)
XX -1.640225 -2.715627 -0.043297 F 21 C 10 0.281026 -0.362335 0.104584 -0.422559 (3 -1)
XX -2.759636 -1.822873 0.849028 F 21 C 12 0.005987 0.026051 1.152631 0.001332 (3 -1)
XX -0.536002 -3.101539 0.510446 RING 0.010861 0.060605 -2.111527 0.002215 (3 1)
XX -1.823703 -2.417606 -0.694967 F 22 C 10 0.273363 -0.361656 0.107597 -0.405716 (3 -1)
XX 1.206603 -2.810506 0.632030 F 23 C 9 0.280617 -0.344143 0.104783 -0.422095 (3 -1)
XX 0.000440 -3.359447 -0.003945 F 23 F 20 0.016420 0.073220 0.024496 0.000597 (3 -1)
XX 1.641942 -2.713826 0.040266 F 24 C 9 0.280947 -0.362902 0.104672 -0.422381 (3 -1)
XX 0.616082 -3.181814 -0.791083 F 24 C 10 0.011127 0.053382 0.754718 0.001125 (3 -1)
XX 2.765987 -1.822378 -0.851036 F 24 C 16 0.006070 0.026375 1.077618 0.001338 (3 -1)
XX 0.540008 -3.098927 -0.522129 RING 0.010808 0.060225 -2.147620 0.002201 (3 1)
XX 1.823417 -2.418326 0.693780 F 25 C 9 0.273341 -0.361881 0.107603 -0.405661 (3 -1)
XX 1.824981 2.417291 -0.692799 F 26 C 8 0.273294 -0.362048 0.107629 -0.405553 (3 -1)
XX 1.207069 2.808699 -0.635835 F 27 C 8 0.280579 -0.344498 0.104881 -0.422009 (3 -1)
XX 0.000395 3.358841 -0.004486 F 27 F 17 0.016343 0.072915 0.025221 0.000602 (3 -1)
XX 0.612987 3.187628 0.790378 F 28 C 7 0.011256 0.053714 0.686757 0.001092 (3 -1)
XX 1.639689 2.715201 -0.041438 F 28 C 8 0.280940 -0.363290 0.104675 -0.422363 (3 -1)
XX 2.760180 1.824584 0.850758 F 28 C 15 0.006005 0.026119 1.129300 0.001332 (3 -1)
XX 3.820781 -0.670634 -1.403038 N 29 C 16 0.458716 0.186493 0.065451 -0.819290 (3 -1)
XX 3.817511 0.672917 1.408558 N 30 C 15 0.458720 0.186466 0.065452 -0.819299 (3 -1)
XX -3.815751 -0.670065 1.413064 N 31 C 12 0.458712 0.186235 0.065492 -0.819290 (3 -1)
XX -3.823455 0.667119 -1.401659 N 32 C 13 0.458722 0.186615 0.065432 -0.819300 (3 -1)

M5d Singlet state
XX 0.003733 -0.005309 0.784711 C 2 C 1 0.261989 -0.622855 0.065805 -0.220721 (3 -1)

XX 1.149497 -0.249872 0.679936 RING 0.022340 0.159096 -1.145022 0.008036 (3 1)

XX 0.627639 -0.029203 1.821314 C 3 C 2 0.333842 -0.915019 0.363439 -0.359956 (3 -1)

XX 1.818155 0.009026 1.622704 C 4 C 3 0.262288 -0.623191 0.068138 -0.221222 (3 -1)

XX 2.299315 -0.470512 0.566482 C 5 C 4 0.262021 -0.623018 0.065726 -0.220771 (3 -1)

XX 0.487530 -0.469496 -0.277372 C 6 C 1 0.262333 -0.623371 0.068415 -0.221295 (3 -1)

XX 1.588575 -0.965428 -0.278591 C 6 C 5 0.333847 -0.915099 0.363278 -0.359961 (3 -1)

XX -0.615938 -0.140477 1.890355 C 7 C 2 0.257998 -0.643460 0.033436 -0.212942 (3 -1)

XX -0.072070 -0.777301 3.026211 RING 0.007812 0.045654 -1.219031 0.002411 (3 1)

XX 1.384432 -0.056053 2.821858 C 8 C 3 0.257728 -0.642508 0.031653 -0.212293 (3 -1)

XX 3.931270 -0.357323 0.604243 RING 0.010264 0.049961 -2.133892 0.002317 (3 1)

XX 2.732352 -1.447593 -0.128665 C 9 C 5 0.258002 -0.643475 0.033459 -0.212946 (3 -1)

XX 0.754965 -1.400730 -1.109248 C 10 C 6 0.257665 -0.642208 0.031670 -0.212199 (3 -1)

XX 1.901181 -2.467297 -0.641947 RING 0.007822 0.045657 -1.232291 0.002406 (3 1)

XX -0.334685 0.474068 -0.319132 C 11 C 1 0.325931 -0.891121 0.355456 -0.341909 (3 -1)

XX -1.518076 0.519100 0.509853 RING 0.010308 0.050219 -2.136019 0.002325 (3 1)

XX 0.133253 -0.397572 -2.272268 RING 0.005579 0.025595 -1.700052 0.001487 (3 1)

XX -0.628236 1.003663 -1.364209 C 12 C 11 0.282553 -0.758087 0.078869 -0.265066 (3 -1)

XX -0.688737 -0.395423 -1.678076 RING 0.008151 0.038573 -3.716093 0.001871 (3 1)

XX -1.034889 1.456942 -0.381261 C 13 C 11 0.282408 -0.757326 0.079592 -0.264549 (3 -1)

XX 2.876314 0.476745 1.142986 C 14 C 4 0.326000 -0.891471 0.355737 -0.342060 (3 -1)

XX 2.350081 1.564596 3.325684 C 15 C 8 0.005804 0.023794 1.191134 0.001163 (3 -1)

XX 3.419292 1.435564 1.637746 C 15 C 14 0.282541 -0.758030 0.078907 -0.265048 (3 -1)

XX 3.831392 1.019592 0.640542 C 16 C 14 0.282374 -0.757142 0.079637 -0.264503 (3 -1)

XX -1.319868 -0.731796 2.386212 F 17 C 7 0.275968 -0.386722 0.108569 -0.410856 (3 -1)

XX -1.324738 -0.041968 2.662621 F 18 C 7 0.283174 -0.343591 0.102464 -0.427469 (3 -1)

XX -1.656847 -0.186924 2.020336 F 19 C 7 0.278537 -0.345636 0.101644 -0.417397 (3 -1)

XX -1.886553 1.000361 0.750191 F 19 C 13 0.012446 0.052141 0.451908 0.001475 (3 -1)

XX 0.628390 -2.325562 -1.588554 F 20 C 10 0.279386 -0.359619 0.105238 -0.419019 (3 -1)

XX 0.057020 -1.876561 -1.723067 F 21 C 10 0.275929 -0.360875 0.104127 -0.411470 (3 -1)

XX -0.686210 -0.400924 -1.851477 F 21 C 12 0.008186 0.037217 2.231243 0.001682 (3 -1)

XX 0.694889 -1.783414 -2.087382 F 22 C 10 0.282893 -0.363233 0.100155 -0.426717 (3 -1)
XX 0.250821 -0.284252 -2.620584 F 22 C 12 0.005769 0.023678 1.258375 0.001162 (3 -1)
XX 3.177799 -2.390145 -0.061302 F 23 C 9 0.276031 -0.386421 0.108338 -0.411007 (3 -1)
XX 1.907038 -3.109446 -0.547100 F 23 F 20 0.010511 0.047896 0.098709 0.000797 (3 -1)
XX 2.146774 -2.649877 -1.453008 F 23 F 21 0.009348 0.045416 0.074655 0.001166 (3 -1)
XX 3.354152 -2.069086 -0.707792 F 24 C 9 0.283140 -0.344123 0.102446 -0.427395 (3 -1)
XX 2.017122 -2.141054 -1.782052 RING 0.007367 0.043235 -1.517781 0.002127 (3 1)
XX 2.226922 -1.894406 -2.158143 F 24 F 22 0.008446 0.040852 0.221676 0.001095 (3 -1)
XX 3.694928 -1.864760 -0.086368 F 25 C 9 0.278566 -0.345315 0.101622 -0.417458 (3 -1)
XX 4.389011 -0.273982 0.158279 F 25 C 16 0.012321 0.051674 0.458105 0.001481 (3 -1)
XX 1.438583 0.272045 3.819886 F 26 C 8 0.282764 -0.364192 0.100273 -0.426419 (3 -1)
XX 0.045393 0.218265 3.675057 RING 0.007366 0.043177 -1.533001 0.002121 (3 1)
XX -0.041616 0.674932 3.781802 F 26 F 18 0.008325 0.040465 0.244831 0.001117 (3 -1)
XX 1.288732 -0.451815 3.788967 F 27 C 8 0.279478 -0.358803 0.105219 -0.419231 (3 -1)
XX -0.267619 -1.304265 3.348543 F 27 F 17 0.010377 0.047470 0.108131 0.000818 (3 -1)
XX -0.265336 -0.334093 3.733293 F 27 F 18 0.009510 0.045949 0.067296 0.001139 (3 -1)
XX 1.977065 -0.212842 3.666965 F 28 C 8 0.275966 -0.360629 0.104184 -0.411560 (3 -1)
XX 3.110091 0.626884 2.794098 RING 0.008031 0.037805 -4.855367 0.001828 (3 1)
XX 3.120218 0.702375 2.891078 F 28 C 15 0.008043 0.036810 3.372046 0.001696 (3 -1)
XX -0.560811 1.114269 -2.501448 N 29 C 12 0.463425 0.339220 0.037468 -0.825662 (3 -1)
XX -1.617165 2.319472 0.094700 N 30 C 13 0.463332 0.336745 0.033758 -0.825582 (3 -1)
XX 4.582189 1.167469 -0.210434 N 31 C 16 0.463331 0.336761 0.033733 -0.825577 (3 -1)
XX 3.508788 2.255949 2.430870 N 32 C 15 0.463425 0.339246 0.037519 -0.825660 (3 -1)
XX 2.389998 1.209116 3.126647 RING 0.005577 0.025681 -1.626473 0.001501 (3 1)
XX -0.342500 -0.705881 3.379362 RING 0.008092 0.047301 -1.516213 0.002182 (3 1)

M5e Triplet state
XX -0.004630 0.000371 0.700725 C 2 C 1 0.303809 -0.801746 0.216903 -0.294611 (3 -1)
XX 1.225427 -0.000415 0.723341 RING 0.018908 0.145587 -1.185455 0.008329 (3 1)
XX 0.607390 0.000643 1.770775 C 3 C 2 0.301191 -0.791880 0.213936 -0.289410 (3 -1)

XX 1.839905 -0.000044 1.789267 C   4 C   3 0.303793 -0.801659 0.216878 -0.294580 (3 -1)
XX 2.455480 -0.002865 0.745953 C   5 C   4 0.303802 -0.801712 0.216888 -0.294597 (3 -1)
XX 0.610946 0.000927 -0.342584 C   6 C   1 0.303799 -0.801687 0.216892 -0.294592 (3 -1)
XX 1.843455 -0.001635 -0.324098 C   6 C   5 0.301190 -0.791879 0.213937 -0.289409 (3 -1)
XX -0.590604 -0.027755 1.754806 C   7 C   2 0.284335 -0.767099 0.063691 -0.268015 (3 -1)
XX 1.200402 0.030195 2.811794 C   8 C   3 0.284338 -0.767114 0.063683 -0.268019 (3 -1)
XX 3.041360 -0.033222 -0.308134 C   9 C   5 0.284336 -0.767101 0.063693 -0.268017 (3 -1)
XX 1.250499 0.028811 -1.365133 C 10 C   6 0.284337 -0.767114 0.063681 -0.268019 (3 -1)
XX -0.638365 0.002111 -0.376408 C 11 C   1 0.263434 -0.634918 0.082875 -0.222967 (3 -1)
XX -1.413273 -0.480450 -1.205153 C 12 C 11 0.296240 -0.804798 0.148321 -0.292091 (3 -1)
XX -1.737133 0.488031 -0.653179 C 13 C 11 0.296224 -0.804712 0.148307 -0.292062 (3 -1)
XX 3.089210 -0.002155 1.823087 C 14 C   4 0.263444 -0.634959 0.082939 -0.222984 (3 -1)
XX 3.862713 -0.486122 2.652325 C 15 C 14 0.296237 -0.804785 0.148307 -0.292085 (3 -1)
XX 4.189388 0.480850 2.099382 C 16 C 14 0.296222 -0.804705 0.148294 -0.292057 (3 -1)
XX 4.086932 -1.313427 3.392479 N 17 C 15 0.458379 0.182570 0.073715 -0.818616 (3 -1)
XX 4.946051 1.307910 1.938134 N 18 C 16 0.458379 0.182627 0.073694 -0.818613 (3 -1)
XX -1.639846 -1.307857 -1.944474 N 19 C 12 0.458379 0.182542 0.073719 -0.818615 (3 -1)
XX -2.491359 1.317464 -0.492706 N 20 C 13 0.458378 0.182595 0.073697 -0.818612 (3 -1)
XX 4.026880 -0.088813 -0.888618 N 21 C   9 0.463860 0.332188 0.036023 -0.826720 (3 -1)
XX 1.282454 0.082337 -2.508545 N 22 C 10 0.463861 0.332218 0.036021 -0.826720 (3 -1)
XX 1.168526 0.084575 3.955166 N 23 C   8 0.463860 0.332215 0.036024 -0.826719 (3 -1)
XX -1.576307 -0.080315 2.335265 N 24 C   7 0.463861 0.332197 0.036020 -0.826722 (3 -1)

M5e Singlet state

XX -0.028555 -0.100700 0.689589 C   2 C   1 0.274811 -0.683782 0.112678 -0.241480 (3 -1)
XX 1.160542 0.228807 0.713184 RING 0.018587 0.139587 -1.172380 0.007861 (3 1)
XX 0.631203 -0.220165 1.728891 C   3 C   2 0.321601 -0.875139 0.311845 -0.331010 (3 -1)
XX 1.862722 -0.126115 1.664070 C   4 C   3 0.274834 -0.683888 0.112709 -0.241518 (3 -1)
XX 2.384758 0.377063 0.663920 C   5 C   4 0.274802 -0.683731 0.112670 -0.241464 (3 -1)
XX 0.493483 0.402542 -0.310551 C   6 C   1 0.274815 -0.683810 0.112660 -0.241487 (3 -1)

XX 1.669870 0.781037 -0.261032 C 6 C 5 0.321611 -0.875185 0.311876 -0.331030 (3 -1)
XX -1.393338 -0.691614 0.785096 RING 0.010819 0.053492 -1.804061 0.003203 (3 1)
XX -0.551435 -0.293886 1.786996 C 7 C 2 0.285100 -0.766672 0.068435 -0.269127 (3 -1)
XX 1.268454 -0.318548 2.724665 C 8 C 3 0.285096 -0.766654 0.068421 -0.269121 (3 -1)
XX 2.564304 -0.744230 2.824183 RING 0.010824 0.053529 -1.802873 0.003205 (3 1)
XX 3.842564 0.488033 0.375930 RING 0.010822 0.053512 -1.802971 0.003204 (3 1)
XX 2.794759 1.152772 -0.199308 C 9 C 5 0.285094 -0.766641 0.068425 -0.269118 (3 -1)
XX 0.974830 1.177544 -1.136844 C 10 C 6 0.285109 -0.766722 0.068439 -0.269144 (3 -1)
XX -0.589569 -0.197540 -0.414896 C 11 C 1 0.317069 -0.853891 0.302654 -0.321853 (3 -1)
XX -0.115058 0.540999 -1.663949 RING 0.010818 0.053469 -1.804804 0.003201 (3 1)
XX -0.149677 0.685897 -2.037648 C 12 C 10 0.011486 0.043209 0.629010 0.002107 (3 -1)
XX -1.139011 -0.353219 -1.490902 C 12 C 11 0.284223 -0.760182 0.085876 -0.267477 (3 -1)
XX -1.721354 -0.829614 0.973259 C 13 C 7 0.011489 0.043224 0.628262 0.002107 (3 -1)
XX -1.625325 -0.822299 -0.559060 C 13 C 11 0.284232 -0.760231 0.085878 -0.267494 (3 -1)
XX 3.085460 -0.246535 1.478859 C 14 C 4 0.317085 -0.853996 0.302620 -0.321884 (3 -1)
XX 2.598540 -0.887288 3.199489 C 15 C 8 0.011497 0.043246 0.627060 0.002107 (3 -1)
XX 3.790667 -0.894140 2.232003 C 15 C 14 0.284224 -0.760180 0.085867 -0.267480 (3 -1)
XX 4.170622 0.628259 0.188421 C 16 C 9 0.011494 0.043235 0.627295 0.002107 (3 -1)
XX 4.277208 -0.425079 1.300315 C 16 C 14 0.284219 -0.760156 0.085865 -0.267470 (3 -1)
XX 3.984768 -1.569054 3.128485 N 17 C 15 0.462620 0.324226 0.044588 -0.824295 (3 -1)
XX 5.248072 -0.351147 0.709705 N 18 C 16 0.462620 0.324240 0.044586 -0.824295 (3 -1)
XX -1.219271 -0.265460 -2.623480 N 19 C 12 0.462622 0.324245 0.044575 -0.824299 (3 -1)
XX -2.481886 -1.483466 -0.204080 N 20 C 13 0.462622 0.324239 0.044580 -0.824300 (3 -1)
XX 3.618437 1.826774 -0.609535 N 21 C 9 0.463231 0.334192 0.040207 -0.825271 (3 -1)
XX 0.845125 1.864642 -2.037971 N 22 C 10 0.463231 0.334183 0.040191 -0.825270 (3 -1)
XX 1.284940 -0.422717 3.860387 N 23 C 8 0.463231 0.334196 0.040216 -0.825270 (3 -1)
XX -1.488042 -0.384959 2.431545 N 24 C 7 0.463233 0.334241 0.040199 -0.825274 (3 -1)

M7a Triplet state
XX -1.084141 0.045774 -0.597925 C 2 C 1 0.301224 -0.780096 0.267199 -0.291596 (3 -1)

XX -0.001905 -0.007009 -0.010003 RING 0.018806 0.145563 -1.169169 0.008316 (3 1)
XX -0.001122 0.102213 -1.222096 C 3 C 2 0.307900 -0.820606 0.286306 -0.301427 (3 -1)
XX 1.072818 0.042231 -0.607075 C 4 C 3 0.301524 -0.774765 0.280850 -0.291715 (3 -1)
XX 1.079868 -0.062190 0.583422 C 5 C 4 0.303416 -0.785772 0.278932 -0.295848 (3 -1)
XX -1.081879 -0.067243 0.582234 C 6 C 1 0.303346 -0.786808 0.278500 -0.296237 (3 -1)
XX 0.004318 -0.118784 1.201300 C 6 C 5 0.308385 -0.827706 0.270655 -0.302020 (3 -1)
XX -0.963271 0.178089 -1.671581 N 7 C 2 0.293732 -0.921332 0.094262 -0.403377 (3 -1)
XX 0.015391 0.311224 -2.620177 RING 0.016263 0.086271 -2.507146 0.004238 (3 1)
XX 0.957055 0.130913 -1.705794 N 8 C 3 0.281612 -0.852765 0.080982 -0.354413 (3 -1)
XX 0.955295 -0.159484 1.676252 N 9 C 5 0.287423 -0.887246 0.098450 -0.375833 (3 -1)
XX -0.948031 -0.159220 1.672647 N 10 C 6 0.291314 -0.912946 0.091639 -0.387742 (3 -1)
XX -2.185786 -0.009164 -0.002538 C 11 C 1 0.254789 -0.577441 0.026921 -0.209694 (3 -1)
XX -3.303321 0.690540 0.049591 C 12 C 11 0.234280 -0.493530 0.070439 -0.179869 (3 -1)
XX -3.309086 -0.691923 -0.049269 C 13 C 11 0.234027 -0.491872 0.071712 -0.179338 (3 -1)
XX -4.773978 0.009163 0.030538 RING 0.006717 0.029411 -1.202105 0.001530 (3 1)
XX 2.179297 -0.005452 -0.013774 C 14 C 4 0.254643 -0.577921 0.029887 -0.209471 (3 -1)
XX 1.795103 1.347983 -1.417228 RING 0.006893 0.028435 -1.872852 0.001415 (3 1)
XX 3.294210 0.697989 0.032346 C 15 C 14 0.233777 -0.490941 0.069977 -0.179140 (3 -1)
XX 3.308792 -0.686620 -0.048403 C 16 C 14 0.233974 -0.491694 0.070401 -0.179294 (3 -1)
XX 4.767051 0.010921 0.066537 RING 0.006736 0.029417 -1.207934 0.001526 (3 1)
XX 1.754431 -1.416808 1.339178 RING 0.007529 0.027118 -3.184118 0.001127 (3 1)
XX 4.240080 -1.421363 0.431870 C 17 C 16 0.236131 -0.517457 0.003811 -0.183055 (3 -1)
XX 3.173838 -1.947641 0.055697 C 18 C 16 0.238587 -0.528873 0.003864 -0.186098 (3 -1)
XX 2.813641 -0.715953 -2.221682 C 19 N 8 0.007357 0.026861 0.458041 0.001457 (3 -1)
XX 4.437664 -0.023157 -0.949092 RING 0.007388 0.032444 -1.279498 0.001636 (3 1)
XX 3.969862 -1.492378 -0.785151 C 19 C 16 0.232606 -0.501680 0.004929 -0.178660 (3 -1)
XX 4.261808 1.437801 -0.360861 C 20 C 15 0.236992 -0.521343 0.003626 -0.184145 (3 -1)
XX 2.858637 0.490462 1.703864 RING 0.006590 0.026030 -1.453505 0.001331 (3 1)
XX 3.149187 1.952335 -0.129704 C 21 C 15 0.237730 -0.524902 0.003522 -0.185005 (3 -1)
XX 2.661634 0.763347 2.264761 C 22 N 9 0.008251 0.034233 1.034979 0.001937 (3 -1)

XX 4.356952 0.058380 1.069198 RING   0.007058 0.030079 -1.283701 0.001498 (3 1)
XX 3.848635 1.534266 0.815813 C 22 C 15 0.232121 -0.499284 0.004603 -0.178098 (3 -1)
XX -4.433180 0.178953 -0.947138 RING   0.007287 0.031888 -1.282113 0.001615 (3 1)
XX -4.274652 -1.358834 -0.564872 C 23 C 13 0.237015 -0.521354 0.003427 -0.184197 (3 -1)
XX -1.837447 -1.132778 -1.618353 RING   0.006385 0.024223 -1.852923 0.001130 (3 1)
XX -3.182540 -1.918120 -0.360819 C 24 C 13 0.238077 -0.526549 0.003266 -0.185428 (3 -1)
XX -2.670467 -1.124069 2.159098 C 25 N   9 0.008307 0.033556 1.140587 0.001849 (3 -1)
XX -3.907397 -1.612868 0.603247 C 25 C 13 0.232258 -0.499912 0.004582 -0.178243 (3 -1)
XX -4.389518 -0.176063 1.022970 RING   0.007143 0.030689 -1.280724 0.001536 (3 1)
XX -4.232457 1.369000 0.598582 C 26 C 12 0.236379 -0.518590 0.003945 -0.183405 (3 -1)
XX -1.791442 1.156672 1.626886 RING   0.006982 0.024331 -4.318841 0.000970 (3 1)
XX -3.147974 1.917985 0.327663 C 27 C 12 0.238491 -0.528571 0.004046 -0.186011 (3 -1)
XX -2.722833 0.852854 -1.520293 RING   0.006362 0.022437 -1.336236 0.000831 (3 1)
XX -2.589077 1.274758 -2.156248 C 28 N   7 0.009837 0.029654 0.108547 0.000868 (3 -1)
XX -3.929109 1.601176 -0.594009 C 28 C 12 0.232322 -0.500358 0.005415 -0.178324 (3 -1)
XX -5.155653 -1.986072 -1.067472 H 29 C 23 0.275605 -0.933373 0.004518 -0.279613 (3 -1)
XX -5.378219 -0.936280 -0.833941 H 30 C 23 0.278670 -0.955503 0.005383 -0.285620 (3 -1)
XX -5.037126 0.181825 -1.312268 H 30 C 28 0.009777 0.039550 1.178728 0.002256 (3 -1)
XX -4.713239 -1.182656 -1.674106 H 31 C 23 0.275560 -0.931684 0.006374 -0.279648 (3 -1)
XX -3.046865 -3.061807 -0.653020 H 32 C 24 0.275557 -0.931700 0.006368 -0.279602 (3 -1)
XX -2.484765 -2.331189 -1.249616 H 33 C 24 0.275669 -0.931819 0.007027 -0.279889 (3 -1)
XX -2.167485 -2.552870 -0.216751 H 34 C 24 0.279344 -0.967728 0.004719 -0.286693 (3 -1)
XX -4.456553 -2.467768 1.231216 H 35 C 25 0.274248 -0.922673 0.007005 -0.277217 (3 -1)
XX -4.964295 -0.182147 1.386995 H 35 C 26 0.009300 0.037227 0.964209 0.002142 (3 -1)
XX -3.620674 -1.946293 1.727751 H 36 C 25 0.278653 -0.960951 0.007561 -0.285429 (3 -1)
XX -2.872261 -0.750374 1.631670 RING   0.006507 0.025669 -1.435553 0.001318 (3 1)
XX -4.593384 -1.436176 1.585659 H 37 C 25 0.278673 -0.956037 0.008630 -0.285699 (3 -1)
XX -4.479186 2.459123 -1.208256 H 38 C 28 0.274077 -0.920370 0.008136 -0.276930 (3 -1)
XX -3.696244 1.882648 -1.742551 H 39 C 28 0.280246 -0.978997 0.007064 -0.288659 (3 -1)
XX -4.683509 1.427474 -1.520669 H 40 C 28 0.278624 -0.953926 0.009682 -0.285735 (3 -1)

XX	-1.863502	1.168197	1.761509	H	41	N	9	0.006998	0.023076	2.352406	0.000857	(3 -1)
XX	-5.088120	2.008586	1.134960	H	41	C	26	0.275609	-0.934142	0.004253	-0.279644	(3 -1)
XX	-5.543150	0.007174	0.059895	H	41	H	29	0.009974	0.036396	0.133318	0.001757	(3 -1)
XX	-5.321407	0.953730	0.939344	H	42	C	26	0.278171	-0.951033	0.005544	-0.284665	(3 -1)
XX	-4.611931	1.221033	1.735209	H	43	C	26	0.275078	-0.927395	0.006349	-0.278802	(3 -1)
XX	-2.989049	3.060868	0.598059	H	44	C	27	0.275459	-0.930453	0.006815	-0.279462	(3 -1)
XX	-2.394178	2.330835	1.166170	H	45	C	27	0.276570	-0.940122	0.007067	-0.281472	(3 -1)
XX	-2.143305	2.542365	0.110156	H	46	C	27	0.279429	-0.969264	0.005097	-0.286831	(3 -1)
XX	3.033020	-3.120872	0.152579	H	47	C	18	0.275583	-0.931578	0.006580	-0.279651	(3 -1)
XX	2.414386	-2.491751	0.811806	H	48	C	18	0.277540	-0.949094	0.006638	-0.283284	(3 -1)
XX	2.184798	-2.550555	-0.265726	H	49	C	18	0.278430	-0.958862	0.005830	-0.284960	(3 -1)
XX	4.570891	-2.245581	-1.494779	H	50	C	19	0.274785	-0.927553	0.006232	-0.278137	(3 -1)
XX	5.038461	0.045805	-1.308731	H	50	C	20	0.009966	0.040452	1.414274	0.002277	(3 -1)
XX	3.785038	-1.639333	-1.966578	H	51	C	19	0.276561	-0.939625	0.008025	-0.281682	(3 -1)
XX	2.916606	-0.550790	-1.760475	RING				0.006537	0.023440	-1.557555	0.001149	(3 1)
XX	4.739017	-1.171447	-1.669673	H	52	C	19	0.279917	-0.966915	0.007213	-0.287994	(3 -1)
XX	1.880296	-1.474803	1.596441	H	53	N	10	0.007642	0.024476	1.008937	0.000833	(3 -1)
XX	5.093596	-2.112904	0.900503	H	53	C	17	0.275616	-0.934147	0.004804	-0.279640	(3 -1)
XX	5.309734	-1.036326	0.854978	H	54	C	17	0.278635	-0.955494	0.005819	-0.285525	(3 -1)
XX	4.904672	0.026553	1.442694	H	54	C	22	0.009022	0.036021	0.969177	0.002089	(3 -1)
XX	4.584950	-1.421358	1.587045	H	55	C	17	0.275478	-0.930929	0.006825	-0.279535	(3 -1)
XX	5.145911	2.139738	-0.745789	H	56	C	20	0.275918	-0.936390	0.004282	-0.280204	(3 -1)
XX	5.540025	0.021111	0.120180	H	56	H	53	0.010075	0.036683	0.135006	0.001758	(3 -1)
XX	5.383448	1.073367	-0.643231	H	57	C	20	0.278125	-0.950179	0.005896	-0.284587	(3 -1)
XX	4.740656	1.416710	-1.467594	H	58	C	20	0.275668	-0.932712	0.006372	-0.279813	(3 -1)
XX	4.357542	2.311097	1.566858	H	59	C	22	0.274579	-0.925905	0.006826	-0.277871	(3 -1)
XX	3.515971	1.717708	1.962973	H	60	C	22	0.278549	-0.960347	0.007571	-0.285190	(3 -1)
XX	4.503036	1.245424	1.793491	H	61	C	22	0.278277	-0.952270	0.008817	-0.284945	(3 -1)
XX	3.006043	3.116794	-0.283889	H	62	C	21	0.275408	-0.930060	0.007408	-0.279376	(3 -1)
XX	2.468822	2.457559	-0.984688	H	63	C	21	0.277844	-0.952777	0.007632	-0.283744	(3 -1)

XX 2.124063 2.548904 0.062100 H 64 C 21 0.279078 -0.964994 0.006302 -0.286162 (3 -1)
XX 2.096327 0.298748 -2.401911 H 65 N 8 0.343222 -1.784234 0.046523 -0.504964 (3 -1)
XX 2.008819 1.445898 -1.802499 H 65 H 63 0.007401 0.029666 0.325015 0.001499 (3 -1)
XX 1.227647 -0.377334 -2.924049 H 66 N 8 0.337014 -1.715163 0.050693 -0.489889 (3 -1)
XX -2.015843 -0.071963 -2.539488 H 66 H 40 0.342824 -1.785003 0.054888 -0.504975 (3 -1)
XX -0.938807 0.372599 -3.038235 H 67 N 7 0.344218 -1.814389 0.053079 -0.509877 (3 -1)
XX 0.052888 0.342716 -2.806078 H 67 H 65 0.016444 0.071483 1.044968 0.002775 (3 -1)
XX -2.129293 -1.191587 -1.993450 H 68 H 33 0.006943 0.026455 0.334475 0.001407 (3 -1)
XX -0.997491 0.157722 2.988395 H 69 N 10 0.339066 -1.765311 0.047920 -0.498554 (3 -1)
XX 2.091870 -0.278946 2.405637 H 69 H 55 0.343735 -1.790926 0.046483 -0.506191 (3 -1)
XX -2.076612 -0.103685 2.425174 H 70 N 10 0.343433 -1.792303 0.046111 -0.506075 (3 -1)
XX 1.054442 -0.750285 2.890182 H 71 N 9 0.338515 -1.753034 0.048513 -0.496350 (3 -1)

M7a Singlet state
XX 1.078536 -0.573891 -1.086306 C 2 C 1 0.246711 -0.551238 0.105086 -0.198159 (3 -1)
XX 0.005568 -0.014522 -1.414972 RING 0.023684 0.158353 -1.250792 0.007492 (3 1)
XX 0.099073 -1.200939 -1.395707 C 3 C 2 0.337457 -0.938394 0.492088 -0.368261 (3 -1)
XX -0.987437 -0.651906 -1.017428 C 4 C 3 0.255521 -0.597143 0.054064 -0.211372 (3 -1)
XX -1.089676 0.545562 -1.065229 C 5 C 4 0.248183 -0.561332 0.072134 -0.200554 (3 -1)
XX 0.975986 0.634106 -1.037850 C 6 C 1 0.255117 -0.595553 0.058424 -0.210426 (3 -1)
XX -0.112299 1.175195 -1.397100 C 6 C 5 0.337313 -0.936710 0.477020 -0.365996 (3 -1)
XX 2.483320 -0.977020 -1.320544 RING 0.007743 0.034751 -1.322554 0.001711 (3 1)
XX 1.041183 -1.550996 -1.694483 N 7 C 2 0.279486 -0.825171 0.095513 -0.356010 (3 -1)
XX -0.887082 -1.578626 -1.616493 N 8 C 3 0.291294 -0.903796 0.101341 -0.397306 (3 -1)
XX -2.463330 0.883661 -1.439978 RING 0.008050 0.035372 -1.430617 0.001718 (3 1)
XX -1.055617 1.458629 -1.762613 N 9 C 5 0.279908 -0.822898 0.092360 -0.356812 (3 -1)
XX 0.854356 1.560609 -1.626108 N 10 C 6 0.287321 -0.870435 0.109242 -0.393088 (3 -1)
XX 1.860096 0.115476 -0.294596 C 11 C 1 0.317132 -0.819683 0.367925 -0.324835 (3 -1)
XX 1.285520 1.451010 -0.027975 RING 0.011948 0.059817 -1.431745 0.003140 (3 1)
XX 2.457284 0.847678 0.603559 C 12 C 11 0.224940 -0.466566 0.032322 -0.166502 (3 -1)

XX 1.781231 -1.377258 -0.198934 RING 0.009753 0.048569 -1.389861 0.002794 (3 1)
XX 2.378137 -1.423122 -0.850784 CAGE 0.006866 0.032041 -0.517888 0.001403 (3 3)
XX 2.864881 -0.439854 0.311092 C 13 C 11 0.222113 -0.457151 0.034104 -0.163144 (3 -1)
XX 3.951766 0.611904 1.055304 RING 0.007529 0.033812 -1.186132 0.001751 (3 1)
XX -1.276908 -1.468976 -0.009970 RING 0.011633 0.057955 -1.484647 0.003078 (3 1)
XX -1.856006 -0.126214 -0.253194 C 14 C 4 0.321258 -0.840385 0.380118 -0.333463 (3 -1)
XX -2.462165 -0.840801 0.651883 C 15 C 14 0.227513 -0.477973 0.031933 -0.170241 (3 -1)
XX -1.803452 1.334001 -0.212068 RING 0.010975 0.055800 -1.360237 0.003181 (3 1)
XX -2.839030 0.449933 0.332385 C 16 C 14 0.222815 -0.459529 0.032998 -0.163828 (3 -1)
XX -3.966146 -0.559848 1.080575 RING 0.007258 0.032143 -1.180073 0.001651 (3 1)
XX -3.351425 -0.098334 1.649435 RING 0.009802 0.046916 -1.398361 0.002399 (3 1)
XX -3.850817 1.026576 0.973282 C 17 C 16 0.235311 -0.513608 0.004016 -0.181687 (3 -1)
XX -1.346698 1.972232 -0.516935 C 18 C 6 0.019097 0.059523 0.052093 0.000971 (3 -1)
XX -0.663173 2.266658 0.776707 C 18 C 12 0.004408 0.015909 2.996710 0.000981 (3 -1)
XX -2.925628 1.713033 0.552201 C 18 C 16 0.235944 -0.515653 0.009277 -0.182664 (3 -1)
XX -2.745413 1.468276 -2.046965 C 19 N 9 0.016772 0.055462 0.193200 0.001292 (3 -1)
XX -3.734544 1.119969 -0.252296 C 19 C 16 0.233232 -0.504805 0.013283 -0.179238 (3 -1)
XX -2.440281 -2.495943 -0.681347 RING 0.004932 0.017844 -1.561670 0.000766 (3 1)
XX 0.776590 -2.201587 1.172366 C 20 C 13 0.003881 0.013036 3.301795 0.000801 (3 -1)
XX -3.204965 -1.850527 0.850587 C 20 C 15 0.236521 -0.519222 0.006636 -0.183569 (3 -1)
XX 0.601480 -2.172725 0.523730 RING 0.003658 0.012064 -2.165735 0.000658 (3 1)
XX -1.973439 -1.982592 1.048513 C 21 C 15 0.237153 -0.521710 0.003642 -0.184255 (3 -1)
XX -2.633696 -1.288124 1.831324 C 22 C 15 0.230897 -0.496729 0.007762 -0.175988 (3 -1)
XX 3.347922 0.137734 1.618677 RING 0.010143 0.048603 -1.407664 0.002468 (3 1)
XX 3.886544 -0.967557 0.967459 C 23 C 13 0.233921 -0.507265 0.003873 -0.179805 (3 -1)
XX 1.738939 -2.321436 -0.807554 C 24 N 7 0.016046 0.061048 1.667498 0.001970 (3 -1)
XX 2.965994 -1.689542 0.588237 C 24 C 13 0.236401 -0.518465 0.006994 -0.183404 (3 -1)
XX 2.769845 -1.705260 -1.858043 C 25 N 7 0.019151 0.057621 0.076642 0.000708 (3 -1)
XX 3.765484 -1.119362 -0.250545 C 25 C 13 0.234158 -0.509495 0.011084 -0.180602 (3 -1)
XX 3.184001 1.875538 0.814522 C 26 C 12 0.236213 -0.517869 0.006387 -0.183079 (3 -1)

XX -0.538853 2.185418 0.461051 RING 0.004320 0.014996 -2.465593 0.000830 (3 1)
XX 0.958696 2.167158 -0.465781 C 27 C 6 0.016999 0.067741 1.888914 0.002574 (3 -1)
XX 1.955414 1.990157 1.007747 C 27 C 12 0.237038 -0.521234 0.002052 -0.183938 (3 -1)
XX 2.613004 1.301601 1.790348 C 28 C 12 0.229439 -0.490921 0.007708 -0.174075 (3 -1)
XX -4.806516 1.554474 1.420193 H 29 C 17 0.276302 -0.935161 0.006252 -0.280809 (3 -1)
XX -4.779658 0.466608 1.519708 H 30 C 17 0.279443 -0.959761 0.007637 -0.287091 (3 -1)
XX -4.778738 -0.725943 1.061123 H 30 C 20 0.011800 0.042155 0.120917 0.001756 (3 -1)
XX -4.131087 1.099069 2.149705 H 31 C 17 0.280527 -0.968426 0.007307 -0.288712 (3 -1)
XX -4.525918 1.745562 -0.889577 H 32 C 19 0.273367 -0.914102 0.010538 -0.275564 (3 -1)
XX -3.771814 1.166235 -1.469059 H 33 C 19 0.284841 -1.015894 0.009084 -0.297731 (3 -1)
XX -4.592668 0.652107 -0.936060 H 34 C 19 0.274850 -0.925913 0.011182 -0.278475 (3 -1)
XX -2.972239 2.893303 0.705993 H 35 C 18 0.274391 -0.921987 0.008784 -0.277453 (3 -1)
XX -2.273321 2.328475 1.338674 H 36 C 18 0.275869 -0.934311 0.009239 -0.280207 (3 -1)
XX 0.788294 2.195188 1.196671 H 36 C 26 0.279277 -0.965649 0.007206 -0.286533 (3 -1)
XX -2.030623 2.495494 0.283899 H 37 C 18 0.282551 -0.988006 0.008725 -0.293559 (3 -1)
XX -3.830576 0.043619 2.446840 H 38 C 17 0.015536 0.068728 0.756598 0.003532 (3 -1)
XX -2.525723 -1.670673 2.956676 H 38 C 22 0.277614 -0.945930 0.010001 -0.283368 (3 -1)
XX -2.246922 -0.626265 2.764965 H 39 C 22 0.275486 -0.932956 0.010963 -0.279608 (3 -1)
XX -3.306556 -0.928516 2.780714 H 40 C 22 0.283333 -0.988407 0.009237 -0.294530 (3 -1)
XX -1.539928 -2.975901 1.490593 H 41 C 21 0.275432 -0.924860 0.009382 -0.279540 (3 -1)
XX -1.298487 -2.625435 -0.743376 H 42 N 8 0.018071 0.062450 0.599695 0.001521 (3 -1)
XX -1.318733 -2.790106 0.430926 H 42 C 21 0.285918 -1.020469 0.007509 -0.299791 (3 -1)
XX -0.812126 -2.196200 1.226161 H 43 C 21 0.277822 -0.951667 0.010355 -0.283851 (3 -1)
XX -3.956887 -2.757627 1.050307 H 44 C 20 0.274613 -0.926863 0.005767 -0.277843 (3 -1)
XX -4.514860 -0.448642 0.068621 RING 0.004676 0.017386 -1.601177 0.000916 (3 1)
XX -4.391266 -1.849448 0.609338 H 45 C 20 0.278025 -0.949567 0.007057 -0.284524 (3 -1)
XX -4.599909 -0.558192 -0.091253 H 45 H 34 0.004715 0.017069 0.622173 0.000991 (3 -1)
XX -3.746412 -2.501027 -0.000560 H 46 C 20 0.276823 -0.944261 0.006492 -0.281931 (3 -1)
XX 4.862385 -1.456437 1.418966 H 47 C 23 0.276995 -0.940439 0.006218 -0.282086 (3 -1)
XX 4.807968 -0.369284 1.493697 H 48 C 23 0.279021 -0.954672 0.008091 -0.286419 (3 -1)

XX	4.783427	0.801244	1.023663	H	48	C	26	0.012833	0.045393	0.117016	0.001729	(3 -1)
XX	3.828699	0.025688	2.439102	H	48	C	28	0.015932	0.070957	0.571321	0.003655	(3 -1)
XX	4.183894	-1.002827	2.144553	H	49	C	23	0.280671	-0.968653	0.007676	-0.288990	(3 -1)
XX	4.592528	-1.739546	-0.838639	H	50	C	25	0.274351	-0.922213	0.009393	-0.277373	(3 -1)
XX	3.786045	-1.286430	-1.447734	H	51	C	25	0.286680	-1.031757	0.005400	-0.301770	(3 -1)
XX	4.575226	-0.652342	-0.987411	H	52	C	25	0.274071	-0.918565	0.011328	-0.277046	(3 -1)
XX	3.041263	-2.845310	0.831547	H	53	C	24	0.274491	-0.922672	0.009455	-0.277743	(3 -1)
XX	2.295504	-2.259785	1.385132	H	54	C	24	0.275089	-0.926852	0.010159	-0.278729	(3 -1)
XX	2.578652	-1.846991	-0.871230	RING				0.007422	0.031749	-1.271123	0.001377	(3 1)
XX	2.118899	-2.514983	0.325071	H	55	C	24	0.286467	-1.024317	0.006891	-0.301213	(3 -1)
XX	2.455087	1.664754	2.916662	H	56	C	28	0.277796	-0.946441	0.011377	-0.283671	(3 -1)
XX	2.254361	0.605102	2.709983	H	57	C	28	0.276413	-0.940939	0.011829	-0.281293	(3 -1)
XX	3.290469	0.981787	2.748777	H	58	C	28	0.283759	-0.992681	0.010026	-0.295324	(3 -1)
XX	1.514244	2.981579	1.459239	H	59	C	27	0.276192	-0.931626	0.008263	-0.280769	(3 -1)
XX	1.280032	2.797965	0.411718	H	60	C	27	0.283335	-0.988127	0.007957	-0.294656	(3 -1)
XX	2.382228	2.365851	-0.631561	RING				0.005413	0.019684	-1.168153	0.000846	(3 1)
XX	3.931739	2.784675	1.036286	H	62	C	26	0.274863	-0.929131	0.005907	-0.278257	(3 -1)
XX	2.876916	2.394018	-1.105232	H	63	N	10	0.007245	0.027274	0.106709	0.001503	(3 -1)
XX	4.374219	1.889200	0.571849	H	63	C	26	0.279398	-0.962510	0.006591	-0.287044	(3 -1)
XX	3.728880	2.551879	-0.016540	H	64	C	26	0.274669	-0.922814	0.007575	-0.278203	(3 -1)
XX	1.043672	2.826249	-2.153381	H	65	N	10	0.339738	-1.755323	0.050742	-0.497716	(3 -1)
XX	2.054295	2.115964	-1.993446	H	66	N	10	0.344379	-1.807224	0.049894	-0.508730	(3 -1)
XX	-1.919853	2.481087	-2.098999	H	67	N	9	0.339256	-1.742056	0.053369	-0.495904	(3 -1)
XX	-1.027791	2.191178	-2.900002	H	68	N	9	0.340611	-1.764329	0.051895	-0.499700	(3 -1)
XX	1.179656	-2.816676	-2.183318	H	69	N	7	0.340586	-1.752155	0.057003	-0.498187	(3 -1)
XX	1.666956	-1.908983	-2.855275	H	70	N	7	0.335782	-1.711172	0.057421	-0.488232	(3 -1)
XX	-2.061485	-1.942149	-2.198882	H	71	N	8	0.342431	-1.794570	0.049413	-0.505344	(3 -1)
XX	-2.702095	-2.451203	-1.026894	H	71	C	20	0.005264	0.018190	0.620836	0.000879	(3 -1)
XX	-1.022397	-2.435626	-2.673115	H	72	N	8	0.339346	-1.755579	0.050023	-0.497414	(3 -1)

M7b Triplet state

```
XX -1.072092 -0.015608 -0.605652 C   2 C   1  0.300775 -0.774103  0.224982 -0.289308 (3 -1)
XX  0.000249 -0.009894  0.000413 RING         0.019312  0.148316 -1.183263  0.008378 (3 1)
XX -0.000989 -0.012468 -1.214438 C   3 C   2  0.304792 -0.798203  0.214369 -0.295791 (3 -1)
XX  1.071464 -0.004548 -0.607916 C   4 C   3  0.300681 -0.773572  0.224972 -0.289123 (3 -1)
XX  1.072611 -0.014389  0.606358 C   5 C   4  0.300575 -0.773026  0.224907 -0.288934 (3 -1)
XX -1.071033 -0.002602  0.608639 C   6 C   1  0.300686 -0.773632  0.224937 -0.289129 (3 -1)
XX  0.001281 -0.009284  1.215234 C   6 C   5  0.304752 -0.797935  0.214575 -0.295721 (3 -1)
XX -1.066851 -0.034891 -1.883588 C   7 C   2  0.250736 -0.583271  0.036466 -0.203814 (3 -1)
XX  1.062736  0.004524 -1.886451 C   8 C   3  0.250685 -0.583077  0.036555 -0.203732 (3 -1)
XX  1.066707 -0.029625  1.884698 C   9 C   5  0.250734 -0.583292  0.036543 -0.203812 (3 -1)
XX -1.062901  0.011676  1.886945 C  10 C   6  0.250637 -0.582856  0.036526 -0.203660 (3 -1)
XX -2.186862 -0.004331  0.002048 C  11 C   1  0.254422 -0.579885  0.037264 -0.208682 (3 -1)
XX -3.304266  0.693407  0.020396 C  12 C  11  0.232998 -0.487343  0.072230 -0.177937 (3 -1)
XX -3.313027 -0.689357 -0.018225 C  13 C  11  0.233031 -0.487466  0.072251 -0.177983 (3 -1)
XX -4.778842  0.010885 -0.011086 RING         0.006729  0.029375 -1.206025  0.001526 (3 1)
XX  2.187442 -0.004334 -0.002286 C  14 C   4  0.254484 -0.580167  0.037326 -0.208778 (3 -1)
XX  3.304489  0.693661 -0.023794 C  15 C  14  0.233108 -0.487824  0.072339 -0.178090 (3 -1)
XX  3.313201 -0.688724  0.018987 C  16 C  14  0.233282 -0.488510  0.072288 -0.178333 (3 -1)
XX  4.777074  0.013740  0.014802 RING         0.006749  0.029504 -1.204026  0.001534 (3 1)
XX  1.316398  0.533873  2.899358 H  17 C   9  0.272798 -0.913406  0.008760 -0.275033 (3 -1)
XX -1.820415  1.246537  1.489941 RING         0.007031  0.027973 -2.042137  0.001517 (3 1)
XX  3.003110  0.636956  1.678165 RING         0.006856  0.025738 -1.527602  0.001316 (3 1)
XX  1.191183 -0.551731  2.941694 H  18 C   9  0.274549 -0.926258  0.008297 -0.278072 (3 -1)
XX  2.091929 -0.120829  2.472875 H  19 C   9  0.281827 -0.983121  0.006278 -0.291751 (3 -1)
XX  2.086607  0.092054 -2.478102 H  20 C   8  0.281752 -0.982656  0.006353 -0.291624 (3 -1)
XX  3.020376 -0.638736 -1.677211 RING         0.006897  0.025835 -1.522374  0.001323 (3 1)
XX  1.309456 -0.564784 -2.898563 H  21 C   8  0.272715 -0.912846  0.008849 -0.274878 (3 -1)
XX -1.833398 -1.268013 -1.487960 RING         0.006968  0.027680 -2.070377  0.001500 (3 1)
XX  1.184989  0.520538 -2.947250 H  22 C   8  0.274527 -0.926086  0.008387 -0.278021 (3 -1)
```

XX -1.310984 0.521081 -2.903862 H 23 C 7 0.272824 -0.913589 0.008741 -0.275062 (3 -1)
XX -3.009240 0.635834 -1.676504 RING 0.006924 0.026066 -1.532968 0.001332 (3 1)
XX 1.827345 1.242316 -1.497219 RING 0.006952 0.027605 -2.021739 0.001496 (3 1)
XX -1.198874 -0.566349 -2.935331 H 24 C 7 0.274384 -0.925088 0.008300 -0.277761 (3 -1)
XX -2.093500 -0.119893 -2.468759 H 25 C 7 0.281918 -0.983809 0.006177 -0.291932 (3 -1)
XX -1.309072 -0.550923 2.903178 H 26 C 10 0.272754 -0.913148 0.008860 -0.274945 (3 -1)
XX 1.829958 -1.261679 1.492470 RING 0.007047 0.028036 -2.091272 0.001521 (3 1)
XX -1.187927 0.535202 2.943987 H 27 C 10 0.274423 -0.925318 0.008410 -0.277815 (3 -1)
XX -2.087483 0.100873 2.476937 H 28 C 10 0.281781 -0.982837 0.006359 -0.291681 (3 -1)
XX -3.021246 -0.633612 1.678612 RING 0.006896 0.025829 -1.524469 0.001322 (3 1)
XX 3.389280 -0.622107 1.789861 RING 0.005727 0.021077 -1.987468 0.001111 (3 1)
XX 4.400359 0.143320 1.001610 RING 0.007224 0.031369 -1.276498 0.001580 (3 1)
XX 4.265224 -1.378529 0.531274 C 29 C 16 0.236233 -0.517959 0.004025 -0.183155 (3 -1)
XX 3.459102 -0.665096 2.092990 C 29 H 17 0.005915 0.021680 0.664692 0.001262 (3 -1)
XX 3.186120 -1.934448 0.255179 C 30 C 16 0.238051 -0.526393 0.003153 -0.185386 (3 -1)
XX 2.917977 -0.878547 -2.182085 C 31 C 8 0.008116 0.031867 0.550604 0.001939 (3 -1)
XX 4.414585 -0.122046 -0.984545 RING 0.007288 0.031809 -1.278685 0.001604 (3 1)
XX 3.943976 -1.571223 -0.659554 C 31 C 16 0.232382 -0.500428 0.005070 -0.178344 (3 -1)
XX 4.258245 1.393125 -0.520914 C 32 C 15 0.236334 -0.518474 0.004002 -0.183271 (3 -1)
XX 3.459887 0.647930 -2.047529 C 32 H 21 0.005445 0.019934 1.308174 0.001167 (3 -1)
XX 3.166643 1.936768 -0.271424 C 33 C 15 0.237990 -0.526115 0.003038 -0.185312 (3 -1)
XX 2.881712 0.881929 2.170072 C 34 C 9 0.007949 0.031398 0.700576 0.001938 (3 -1)
XX 3.908766 1.588875 0.661522 C 34 C 15 0.232326 -0.500144 0.005044 -0.178278 (3 -1)
XX -3.393756 -0.618882 -1.791534 RING 0.005738 0.021120 -2.023701 0.001116 (3 1)
XX -4.399221 0.139997 -1.005699 RING 0.007213 0.031294 -1.278193 0.001574 (3 1)
XX -4.265518 -1.380134 -0.528138 C 35 C 13 0.236368 -0.518581 0.004007 -0.183339 (3 -1)
XX -3.460758 -0.660519 -2.085959 C 35 H 23 0.005910 0.021680 0.698085 0.001261 (3 -1)
XX -3.186399 -1.936013 -0.250588 C 36 C 13 0.238051 -0.526391 0.003130 -0.185386 (3 -1)
XX -2.919133 -0.871311 2.182129 C 37 C 10 0.008098 0.031788 0.551974 0.001936 (3 -1)
XX -4.410835 -0.118650 0.985862 RING 0.007304 0.031893 -1.278168 0.001607 (3 1)

XX -3.943759 -1.569746 0.663123 C 37 C 13 0.232418 -0.500572 0.005035 -0.178394 (3 -1)
XX -4.257130 1.394280 0.517000 C 38 C 12 0.236298 -0.518298 0.004049 -0.183227 (3 -1)
XX -3.459728 0.654085 2.052124 C 38 H 26 0.005508 0.020170 1.152424 0.001181 (3 -1)
XX -3.165728 1.936850 0.264294 C 39 C 12 0.237912 -0.525750 0.003129 -0.185201 (3 -1)
XX -2.892021 0.877126 -2.169554 C 40 C 7 0.008036 0.031742 0.660478 0.001951 (3 -1)
XX -3.910254 1.587524 -0.666610 C 40 C 12 0.232292 -0.499995 0.005021 -0.178223 (3 -1)
XX -2.079680 -1.322616 -1.839362 H 41 H 21 0.007333 0.029117 0.420484 0.001658 (3 -1)
XX -4.987648 -0.086896 1.373206 H 41 H 28 0.009782 0.039635 1.337117 0.002246 (3 -1)
XX -5.132581 -2.030142 -1.026727 H 41 C 35 0.275472 -0.932059 0.004740 -0.279381 (3 -1)
XX -3.410084 0.625075 1.840702 RING 0.005448 0.019947 -2.483215 0.001076 (3 1)
XX -5.356070 -0.969139 -0.859122 H 42 C 35 0.278273 -0.951667 0.005853 -0.284904 (3 -1)
XX -4.964414 0.120362 -1.392927 H 42 C 40 0.009534 0.038486 1.219101 0.002198 (3 -1)
XX -4.662871 -1.264334 -1.661566 H 43 C 35 0.276438 -0.939328 0.006442 -0.281231 (3 -1)
XX -3.051330 -3.097695 -0.450739 H 44 C 36 0.275547 -0.931183 0.006204 -0.279609 (3 -1)
XX -2.473496 -2.417404 -1.092948 H 45 C 36 0.277840 -0.950124 0.006669 -0.283841 (3 -1)
XX -2.179586 -2.560850 -0.039522 H 46 C 36 0.278439 -0.958171 0.005436 -0.284971 (3 -1)
XX -4.530410 -2.383939 1.311224 H 47 C 37 0.274635 -0.925172 0.006102 -0.277872 (3 -1)
XX -3.708607 -1.851479 1.812059 H 48 C 37 0.278103 -0.952547 0.007292 -0.284435 (3 -1)
XX -4.662148 -1.334326 1.612797 H 49 C 37 0.279558 -0.963346 0.007543 -0.287350 (3 -1)
XX -4.480379 2.411042 -1.317325 H 50 C 40 0.274732 -0.925948 0.006006 -0.278081 (3 -1)
XX -3.649799 1.882138 -1.807514 H 51 C 40 0.278050 -0.952218 0.007190 -0.284293 (3 -1)
XX -4.608638 1.365460 -1.634184 H 52 C 40 0.279438 -0.962478 0.007554 -0.287140 (3 -1)
XX -5.122427 2.054427 1.004930 H 53 C 38 0.275514 -0.932282 0.004715 -0.279451 (3 -1)
XX -5.553913 0.016930 -0.019657 H 53 H 41 0.010052 0.036602 0.136198 0.001755 (3 -1)
XX -5.360966 0.998992 0.824753 H 54 C 38 0.278184 -0.950988 0.005893 -0.284711 (3 -1)
XX -4.677000 1.276033 1.641760 H 55 C 38 0.276519 -0.939961 0.006384 -0.281390 (3 -1)
XX -2.083026 1.300884 1.863873 H 56 H 26 0.007472 0.029725 0.445493 0.001697 (3 -1)
XX -3.020350 3.094735 0.480054 H 56 C 39 0.275514 -0.930924 0.006212 -0.279542 (3 -1)
XX -2.461357 2.400002 1.123154 H 57 C 39 0.277937 -0.950739 0.006645 -0.284043 (3 -1)
XX -2.148276 2.552550 0.076299 H 58 C 39 0.278448 -0.958311 0.005451 -0.284968 (3 -1)

XX 2.087600 -1.313114 1.843280 H 59 H 17 0.007428 0.029558 0.478896 0.001688 (3 -1)
XX 3.052376 -3.094698 0.464706 H 59 C 30 0.275523 -0.931021 0.006167 -0.279556 (3 -1)
XX 2.469765 -2.409527 1.097555 H 60 C 30 0.277928 -0.950751 0.006579 -0.284008 (3 -1)
XX 2.181582 -2.563654 0.044118 H 61 C 30 0.278372 -0.957641 0.005468 -0.284835 (3 -1)
XX 4.529555 -2.388376 -1.304781 H 62 C 31 0.274665 -0.925371 0.006148 -0.277935 (3 -1)
XX 4.991398 -0.092437 -1.370357 H 62 C 32 0.009741 0.039452 1.314090 0.002240 (3 -1)
XX 3.709857 -1.855425 -1.808454 H 63 C 31 0.278047 -0.952114 0.007362 -0.284310 (3 -1)
XX 3.414208 0.621980 -1.853434 RING 0.005400 0.019759 -2.640567 0.001072 (3 1)
XX 4.664232 -1.340122 -1.609322 H 64 C 31 0.279511 -0.962997 0.007621 -0.287264 (3 -1)
XX 5.135553 -2.026247 1.027466 H 65 C 29 0.275494 -0.932235 0.004750 -0.279421 (3 -1)
XX 5.353151 -0.963144 0.864791 H 66 C 29 0.278398 -0.952581 0.005790 -0.285164 (3 -1)
XX 4.972765 0.129849 1.385113 H 66 C 34 0.009579 0.038662 1.111939 0.002210 (3 -1)
XX 4.661507 -1.265804 1.665570 H 67 C 29 0.276414 -0.939149 0.006459 -0.281192 (3 -1)
XX 2.089955 1.294902 -1.867784 H 68 H 23 0.007379 0.029309 0.448354 0.001681 (3 -1)
XX 5.125220 2.051786 -1.007696 H 68 C 32 0.275498 -0.932163 0.004745 -0.279416 (3 -1)
XX 5.552764 0.019397 0.023019 H 68 H 65 0.010110 0.036799 0.134172 0.001756 (3 -1)
XX 5.362312 0.996367 -0.825464 H 69 C 32 0.278204 -0.951120 0.005908 -0.284754 (3 -1)
XX 4.680908 1.272848 -1.644639 H 70 C 32 0.276494 -0.939802 0.006427 -0.281341 (3 -1)
XX 4.477263 2.413523 1.312868 H 71 C 34 0.274636 -0.925196 0.006078 -0.277879 (3 -1)
XX 3.645091 1.885279 1.800832 H 72 C 34 0.278139 -0.952827 0.007209 -0.284478 (3 -1)
XX 4.604591 1.368595 1.631971 H 73 C 34 0.279371 -0.961972 0.007665 -0.286993 (3 -1)
XX 3.022829 3.094310 -0.487256 H 74 C 33 0.275562 -0.931231 0.006254 -0.279645 (3 -1)
XX 2.465467 2.400837 -1.133130 H 75 C 33 0.277802 -0.949784 0.006765 -0.283771 (3 -1)
XX 2.148585 2.553135 -0.087527 H 76 C 33 0.278476 -0.958532 0.005517 -0.285032 (3 -1)

M7b Singlet state
XX 1.040572 0.569440 1.196786 C 2 C 1 0.246392 -0.550868 0.037864 -0.197835 (3 -1)
XX 0.000847 0.009788 1.575512 RING 0.025483 0.169522 -1.259444 0.007792 (3 1)
XX 0.058642 1.183585 1.568414 C 3 C 2 0.338712 -0.936993 0.395933 -0.368964 (3 -1)
XX -0.976550 0.659783 1.194057 C 4 C 3 0.245661 -0.547997 0.036189 -0.196584 (3 -1)

XX -1.038952 -0.554143 1.203252 C 5 C 4 0.246547 -0.551565 0.038002 -0.198061 (3 -1)
XX 0.977988 -0.644524 1.201066 C 6 C 1 0.245856 -0.548851 0.036242 -0.196870 (3 -1)
XX -0.056858 -1.164036 1.581524 C 6 C 5 0.338657 -0.936625 0.395916 -0.368835 (3 -1)
XX 2.695129 1.210349 1.157498 RING 0.004808 0.018127 -1.090631 0.000789 (3 1)
XX 1.149959 1.562410 2.048355 C 7 C 2 0.248460 -0.568947 0.049121 -0.201318 (3 -1)
XX -0.997011 1.649535 2.056347 C 8 C 3 0.246347 -0.560372 0.046919 -0.198264 (3 -1)
XX -2.692705 -1.196602 1.170530 RING 0.004815 0.018151 -1.092940 0.000791 (3 1)
XX -1.148096 -1.537797 2.065310 C 9 C 5 0.248484 -0.569039 0.049162 -0.201354 (3 -1)
XX 0.999372 -1.623971 2.074431 C 10 C 6 0.246379 -0.560522 0.046997 -0.198310 (3 -1)
XX 1.757878 -0.074833 0.325324 C 11 C 1 0.320855 -0.835600 0.387089 -0.333383 (3 -1)
XX 1.587403 1.400570 0.298443 RING 0.011459 0.056235 -1.468078 0.002982 (3 1)
XX 1.540479 -1.528236 0.371116 RING 0.011398 0.057055 -1.440035 0.003108 (3 1)
XX 2.381557 -0.780565 -0.573268 C 12 C 11 0.224275 -0.465157 0.034692 -0.165566 (3 -1)
XX 2.625175 1.357556 1.062218 CAGE 0.004791 0.018855 -0.874367 0.000799 (3 3)
XX 2.525281 0.577058 -0.492920 C 13 C 11 0.220306 -0.449919 0.033787 -0.160239 (3 -1)
XX 3.923197 -0.034814 -0.839392 RING 0.007625 0.034313 -1.182024 0.001799 (3 1)
XX -1.545633 1.533210 0.357649 RING 0.011380 0.057034 -1.438172 0.003112 (3 1)
XX -1.756686 0.079245 0.324691 C 14 C 4 0.320747 -0.835013 0.386893 -0.333146 (3 -1)
XX -2.383659 0.773814 -0.580789 C 15 C 14 0.224020 -0.464123 0.034652 -0.165224 (3 -1)
XX -1.584737 -1.397711 0.314412 RING 0.011437 0.056051 -1.469625 0.002973 (3 1)
XX -2.619727 -1.350523 1.073943 CAGE 0.004796 0.018899 -0.869836 0.000801 (3 3)
XX -2.522138 -0.583471 -0.486613 C 16 C 14 0.220443 -0.450472 0.033824 -0.160418 (3 -1)
XX -3.922516 0.019219 -0.838782 RING 0.007654 0.034471 -1.183540 0.001807 (3 1)
XX -1.484116 -1.631176 3.194773 H 17 C 9 0.270464 -0.898268 0.008338 -0.270711 (3 -1)
XX -2.216437 -1.919695 2.428217 H 18 C 9 0.280532 -0.973164 0.008614 -0.289249 (3 -1)
XX -1.345879 -2.566694 2.635412 H 19 C 9 0.275396 -0.934842 0.008895 -0.279453 (3 -1)
XX -2.039034 1.955209 2.534733 H 20 C 8 0.279755 -0.968675 0.009202 -0.287582 (3 -1)
XX -1.165995 1.885616 3.203110 H 21 C 8 0.272686 -0.913389 0.009713 -0.274399 (3 -1)
XX -1.251985 2.729446 2.505341 H 22 C 8 0.274602 -0.929419 0.009509 -0.278042 (3 -1)
XX 1.487067 1.666347 3.176501 H 23 C 7 0.270455 -0.898202 0.008347 -0.270694 (3 -1)

XX 2.218252 1.948668 2.406513 H 24 C 7 0.280524 -0.973120 0.008621 -0.289232 (3 -1)
XX 1.347326 2.596757 2.609080 H 25 C 7 0.275389 -0.934825 0.008906 -0.279423 (3 -1)
XX 1.173985 -1.841416 3.224002 H 26 C 10 0.272551 -0.912411 0.009723 -0.274152 (3 -1)
XX 1.250048 -2.697836 2.540766 H 27 C 10 0.274541 -0.929008 0.009529 -0.277914 (3 -1)
XX 2.042470 -1.928475 2.551896 H 28 C 10 0.279774 -0.968852 0.009221 -0.287624 (3 -1)
XX -2.619647 0.114286 -1.909749 RING 0.009164 0.043398 -1.246248 0.002271 (3 1)
XX -3.003486 -1.254265 -1.511721 C 29 C 16 0.233126 -0.504188 0.004665 -0.178702 (3 -1)
XX -0.069422 -2.307338 -0.015084 RING 0.004886 0.016054 -1.595126 0.000835 (3 1)
XX -1.409868 -2.076369 0.825612 C 30 C 9 0.015837 0.062851 1.572961 0.002763 (3 -1)
XX 0.305108 -2.239481 -1.055198 RING 0.003748 0.012680 -1.400935 0.000770 (3 1)
XX -2.425930 -1.883218 -0.638055 C 30 C 16 0.235709 -0.515748 0.004329 -0.182195 (3 -1)
XX -2.814920 -1.983746 1.060351 RING 0.005507 0.021801 -1.233205 0.001171 (3 1)
XX -3.533363 -1.346891 -0.380066 C 31 C 16 0.234383 -0.510062 0.009359 -0.180768 (3 -1)
XX -3.305848 -1.683868 1.666258 C 31 H 17 0.010515 0.035864 0.055078 0.001428 (3 -1)
XX -1.730253 2.351656 1.163947 RING 0.011282 0.052421 -2.070829 0.002915 (3 1)
XX -3.331003 1.502918 -1.065044 C 32 C 15 0.234370 -0.509814 0.005902 -0.180499 (3 -1)
XX -0.992249 2.121400 0.616343 C 32 H 24 0.017255 0.061096 0.395919 0.002209 (3 -1)
XX -3.369717 0.164811 -1.571900 RING 0.009022 0.043011 -1.165393 0.002304 (3 1)
XX -0.051939 2.588261 -0.468928 C 33 C 13 0.005498 0.019999 0.240629 0.001215 (3 -1)
XX -2.362332 2.086150 -0.595492 C 33 C 15 0.238807 -0.528509 0.001267 -0.186646 (3 -1)
XX 0.056693 2.319021 -0.052852 RING 0.004788 0.015801 -1.640649 0.000832 (3 1)
XX -2.194564 1.454155 -1.640676 C 34 C 15 0.233334 -0.505476 0.008749 -0.179406 (3 -1)
XX -0.305839 2.249001 -1.054031 RING 0.003731 0.012619 -1.403970 0.000769 (3 1)
XX 2.618683 -0.130770 -1.908934 RING 0.009151 0.043322 -1.254736 0.002265 (3 1)
XX 3.009731 1.237090 -1.524335 C 35 C 13 0.233297 -0.504911 0.004448 -0.178941 (3 -1)
XX -0.012997 -0.001523 -2.128715 C 35 C 29 0.001106 0.003346 0.173021 0.000210 (3 -1)
XX 1.414745 2.084715 0.802276 C 36 C 7 0.015841 0.062952 1.600489 0.002770 (3 -1)
XX 2.431430 1.874529 -0.657581 C 36 C 13 0.235936 -0.516766 0.004394 -0.182513 (3 -1)
XX 0.120354 1.947339 -1.532615 C 36 C 34 0.005549 0.019288 0.090158 0.001166 (3 -1)
XX 2.822361 1.993139 1.039643 RING 0.005513 0.021825 -1.234531 0.001173 (3 1)

XX 3.538207 1.338772 -0.393662 C 37 C 13 0.234479 -0.510482 0.009341 -0.180903 (3 -1)
XX 3.308808 1.700058 1.647784 C 37 H 23 0.010453 0.035701 0.055316 0.001433 (3 -1)
XX 3.324951 -1.519727 -1.049670 C 38 C 12 0.234472 -0.510265 0.005861 -0.180634 (3 -1)
XX 1.722368 -2.331349 1.182798 RING 0.011292 0.052412 -2.078676 0.002921 (3 1)
XX -0.117779 -1.946070 -1.516603 C 38 C 29 0.005414 0.018846 0.100176 0.001152 (3 -1)
XX 3.378258 -0.182433 -1.566773 RING 0.008960 0.042595 -1.166592 0.002279 (3 1)
XX 0.985867 -2.110740 0.632164 C 39 C 6 0.017156 0.060924 0.423473 0.002240 (3 -1)
XX 2.351159 -2.093266 -0.577921 C 39 C 12 0.238511 -0.527213 0.001169 -0.186224 (3 -1)
XX 0.045515 -2.597202 -0.453240 C 39 C 16 0.005769 0.020986 0.205285 0.001256 (3 -1)
XX 2.197349 -2.477098 1.482834 C 39 H 27 0.012532 0.053301 1.099986 0.002754 (3 -1)
XX 2.190206 -1.466998 -1.627992 C 40 C 12 0.233166 -0.504764 0.008567 -0.179182 (3 -1)
XX -0.145379 -0.110290 -1.605496 RING 0.001011 0.003096 -1.129342 0.000208 (3 1)
XX -3.505910 -1.864336 -2.395117 H 41 C 29 0.276537 -0.937227 0.006897 -0.281283 (3 -1)
XX -3.507216 -0.780057 -2.522311 H 42 C 29 0.283365 -0.987682 0.007904 -0.294762 (3 -1)
XX -2.571088 -1.344537 -2.632977 H 43 C 29 0.277083 -0.942980 0.008407 -0.282332 (3 -1)
XX -2.559424 -0.066704 -2.716786 H 43 C 34 0.014561 0.061257 1.755141 0.002882 (3 -1)
XX 1.137843 -1.474340 -2.196953 H 43 C 38 0.276974 -0.944585 0.009421 -0.282274 (3 -1)
XX -4.463702 -2.069378 -0.180184 H 44 C 31 0.274627 -0.925798 0.007432 -0.277798 (3 -1)
XX -4.636452 0.105096 -0.848644 H 44 C 32 0.010418 0.042146 0.411690 0.002358 (3 -1)
XX -4.131312 -1.441337 0.661765 H 45 C 31 0.278017 -0.951046 0.008889 -0.284413 (3 -1)
XX -4.672504 -0.989300 -0.179428 H 46 C 31 0.279201 -0.960575 0.007842 -0.286608 (3 -1)
XX -2.296848 -3.025374 -0.910041 H 47 C 30 0.275874 -0.929996 0.008522 -0.280052 (3 -1)
XX -1.373996 -2.441725 -0.783655 H 48 C 30 0.278880 -0.959557 0.007894 -0.285938 (3 -1)
XX -1.974069 -2.740997 0.090809 H 49 C 30 0.284112 -0.995879 0.008187 -0.296382 (3 -1)
XX -1.501838 2.796577 -0.133398 H 50 H 22 0.281936 -0.984955 0.006401 -0.292038 (3 -1)
XX -1.964039 2.082816 -2.625430 H 50 C 34 0.274774 -0.925286 0.008888 -0.278189 (3 -1)
XX -1.140939 1.482704 -2.207091 H 51 C 34 0.277015 -0.944766 0.009435 -0.282358 (3 -1)
XX -1.963769 0.992765 -2.745856 H 52 C 34 0.283450 -0.991822 0.009416 -0.295039 (3 -1)
XX -2.341468 3.259628 -0.671195 H 53 C 33 0.275754 -0.929071 0.006918 -0.279943 (3 -1)
XX -2.206363 2.490067 1.459474 H 54 H 20 0.012474 0.053135 1.138274 0.002764 (3 -1)

XX -2.509529 2.841368 0.323444 H 54 C 33 0.280609 -0.966682 0.007200 -0.289110 (3 -1)
XX -4.207530 2.186398 -1.494335 H 56 C 32 0.275688 -0.932605 0.007080 -0.279793 (3 -1)
XX -4.087437 0.170267 -2.146458 H 56 H 41 0.017722 0.060180 0.092391 0.001410 (3 -1)
XX -4.395947 1.109798 -1.521044 H 57 C 32 0.282253 -0.979239 0.008208 -0.292741 (3 -1)
XX -4.412009 1.641404 -0.561787 H 58 C 32 0.275811 -0.934490 0.008196 -0.280047 (3 -1)
XX 3.510720 1.841006 -2.412085 H 59 C 35 0.276567 -0.937335 0.006889 -0.281331 (3 -1)
XX 3.519777 0.755910 -2.528506 H 60 C 35 0.283125 -0.985878 0.007965 -0.294275 (3 -1)
XX 2.580011 1.313193 -2.648032 H 61 C 35 0.277150 -0.943597 0.008377 -0.282458 (3 -1)
XX 2.569806 0.040035 -2.722118 H 61 C 40 0.014577 0.061329 1.763446 0.002883 (3 -1)
XX 4.471418 2.059722 -0.200840 H 62 C 37 0.274623 -0.925806 0.007410 -0.277796 (3 -1)
XX 1.981734 2.740519 0.062307 H 62 H 55 0.284166 -0.996175 0.008227 -0.296505 (3 -1)
XX 4.135918 1.441633 0.647459 H 63 C 37 0.278041 -0.951271 0.008851 -0.284464 (3 -1)
XX 4.675387 0.978894 -0.189033 H 64 C 37 0.279181 -0.960406 0.007771 -0.286579 (3 -1)
XX 2.300645 3.013554 -0.942754 H 65 C 36 0.275853 -0.929831 0.008604 -0.280002 (3 -1)
XX 1.378850 2.430303 -0.805968 H 66 C 36 0.278883 -0.959572 0.007946 -0.285953 (3 -1)
XX 1.484154 -2.796300 -0.115458 H 68 H 27 0.281879 -0.984641 0.006472 -0.291924 (3 -1)
XX 1.943465 -2.107595 -2.601248 H 68 C 40 0.274880 -0.926058 0.008893 -0.278389 (3 -1)
XX 1.977281 -1.020925 -2.742962 H 70 C 40 0.283480 -0.991997 0.009366 -0.295102 (3 -1)
XX 2.324869 -3.267019 -0.644997 H 71 C 39 0.275851 -0.929718 0.006967 -0.280136 (3 -1)
XX 2.489002 -2.842871 0.347538 H 72 C 39 0.280662 -0.967093 0.007212 -0.289218 (3 -1)
XX 4.197281 -2.211587 -1.473770 H 74 C 38 0.275731 -0.932923 0.007062 -0.279891 (3 -1)
XX 4.094482 -0.196504 -2.141311 H 74 H 59 0.017533 0.059603 0.094193 0.001426 (3 -1)
XX 4.637953 -0.121698 -0.849571 H 75 C 37 0.010397 0.041975 0.388913 0.002345 (3 -1)
XX 4.393365 -1.136626 -1.506130 H 75 C 38 0.282131 -0.978327 0.008218 -0.292499 (3 -1)
XX 4.404426 -1.662824 -0.544004 H 76 C 38 0.275782 -0.934304 0.008203 -0.280000 (3 -1)

M7d Triplet state
XX -1.097949 0.588779 -0.059668 C 2 C 1 0.294118 -0.738334 0.210934 -0.277780 (3 -1)
XX -0.000149 0.000560 -0.003979 RING 0.019413 0.145476 -1.198911 0.008060 (3 1)
XX -0.000061 1.202251 -0.004748 C 3 C 2 0.295999 -0.745004 0.221712 -0.279901 (3 -1)

XX 1.097779 0.587993 0.052652 C 4 C 3 0.294103 -0.738143 0.211244 -0.277780 (3 -1)
XX 1.097659 -0.587569 -0.060532 C 5 C 4 0.294013 -0.737895 0.210569 -0.277586 (3 -1)
XX -1.097940 -0.586851 0.053636 C 6 C 1 0.293980 -0.737646 0.210869 -0.277555 (3 -1)
XX -0.000241 -1.201151 -0.004592 C 6 C 5 0.296094 -0.745400 0.222137 -0.280081 (3 -1)
XX -2.231196 1.160971 -0.588444 RING 0.014003 0.072445 -1.622238 0.002923 (3 1)
XX -1.026371 1.814129 -0.284135 C 7 C 2 0.255595 -0.632675 0.040792 -0.212331 (3 -1)
XX 0.340337 2.616032 -0.444496 RING 0.011631 0.065377 -1.704251 0.002909 (3 1)
XX -0.333843 2.615518 0.420652 RING 0.011701 0.065815 -1.702649 0.002929 (3 1)
XX 1.026720 1.814123 0.270824 C 8 C 3 0.255885 -0.634162 0.040520 -0.212782 (3 -1)
XX 2.229077 1.162388 0.584012 RING 0.013990 0.072410 -1.617848 0.002927 (3 1)
XX 0.005131 2.721068 -0.017030 CAGE 0.010542 0.065688 -0.653826 0.003314 (3 3)
XX 2.233198 -1.159396 -0.593246 RING 0.013926 0.071785 -1.631209 0.002892 (3 1)
XX 1.025843 -1.812922 -0.284916 C 9 C 5 0.255611 -0.632783 0.040671 -0.212356 (3 -1)
XX -0.337286 -2.615052 -0.437353 RING 0.011696 0.065806 -1.705782 0.002926 (3 1)
XX -2.232093 -1.162625 0.587703 RING 0.013874 0.071558 -1.624540 0.002893 (3 1)
XX 0.333320 -2.615701 0.415325 RING 0.011764 0.066174 -1.713660 0.002938 (3 1)
XX -1.026459 -1.813338 0.272041 C 10 C 6 0.255754 -0.633551 0.040498 -0.212582 (3 -1)
XX -0.004756 -2.711867 -0.016094 CAGE 0.010655 0.066616 -0.675339 0.003361 (3 3)
XX -2.270385 0.001626 0.001206 C 11 C 1 0.243160 -0.528784 0.068741 -0.195711 (3 -1)
XX -2.673803 -1.170395 0.668086 C 12 C 10 0.015457 0.058570 0.258514 0.000901 (3 -1)
XX -3.374755 0.245984 0.672991 C 12 C 11 0.227376 -0.466952 0.069290 -0.171194 (3 -1)
XX -2.679195 1.168818 -0.664538 C 13 C 7 0.015656 0.059314 0.254894 0.000891 (3 -1)
XX -3.383332 -0.241406 -0.658992 C 13 C 11 0.227198 -0.466252 0.069150 -0.170951 (3 -1)
XX -5.014135 0.044433 0.023554 CAGE 0.005152 0.020328 -0.950896 0.000961 (3 3)
XX 2.270427 -0.000413 0.000642 C 14 C 4 0.242988 -0.528045 0.068736 -0.195458 (3 -1)
XX 2.676981 1.169891 0.664417 C 15 C 8 0.015656 0.059281 0.250020 0.000887 (3 -1)
XX 2.443323 -1.714945 0.801010 CAGE 0.004093 0.017791 -0.449479 0.000809 (3 3)
XX 3.374156 -0.245035 0.673193 C 15 C 14 0.227332 -0.466775 0.069274 -0.171124 (3 -1)
XX 2.675040 -1.167643 -0.669179 C 16 C 9 0.015497 0.058769 0.265375 0.000905 (3 -1)
XX 3.383843 0.241567 -0.658958 C 16 C 14 0.227487 -0.467438 0.069208 -0.171353 (3 -1)

XX	5.020516	-0.024304	0.023708	CAGE				0.005106	0.020089	-0.940926	0.000948	(3 3)
XX	1.711469	-1.822439	1.190694	RING				0.005633	0.024346	-1.487648	0.001307	(3 1)
XX	1.542356	-2.765478	-0.168103	F	17	C	9	0.274168	-0.353430	0.115670	-0.407182	(3 -1)
XX	0.627355	-3.248874	0.828886	F	17	C	10	0.017688	0.076956	0.060067	0.000370	(3 -1)
XX	2.604593	-2.095386	1.328748	RING				0.004945	0.022341	-1.342589	0.001237	(3 1)
XX	1.105660	-2.773088	-0.763277	F	18	C	9	0.274088	-0.346447	0.115937	-0.407679	(3 -1)
XX	-1.712161	-1.795133	-1.184802	RING				0.005823	0.025116	-1.454037	0.001345	(3 1)
XX	0.274501	-3.090607	0.147030	RING				0.011802	0.066059	-1.963084	0.002332	(3 1)
XX	1.748612	-2.430969	-0.789762	F	19	C	9	0.273780	-0.322758	0.116937	-0.407147	(3 -1)
XX	3.245819	-1.836945	-0.043288	RING				0.005634	0.025220	-1.553970	0.001274	(3 1)
XX	3.361607	-1.149789	-1.229115	RING				0.008445	0.041252	-1.399923	0.001852	(3 1)
XX	1.750266	2.434785	0.770608	F	20	C	8	0.273796	-0.322211	0.117384	-0.407174	(3 -1)
XX	3.358426	1.150577	1.241537	RING				0.008404	0.041034	-1.406367	0.001843	(3 1)
XX	3.260352	1.820654	0.049150	RING				0.005784	0.025921	-1.552309	0.001298	(3 1)
XX	1.714181	1.798103	-1.188675	RING				0.005782	0.024944	-1.459823	0.001337	(3 1)
XX	0.627852	3.236521	-0.861750	F	21	C	7	0.017424	0.075677	0.057977	0.000360	(3 -1)
XX	1.544617	2.763837	0.145353	F	21	C	8	0.274010	-0.354385	0.116256	-0.406820	(3 -1)
XX	2.647809	2.094550	-1.323629	RING				0.005062	0.022942	-1.325821	0.001268	(3 1)
XX	-0.255792	3.095630	0.133796	RING				0.011608	0.064761	-2.034765	0.002293	(3 1)
XX	1.108688	2.778452	0.740837	F	22	C	8	0.274376	-0.344021	0.115952	-0.408335	(3 -1)
XX	-1.716347	1.829074	1.198037	RING				0.005532	0.023900	-1.502023	0.001286	(3 1)
XX	0.267112	3.086097	-0.172132	RING				0.011546	0.064622	-1.976217	0.002318	(3 1)
XX	-1.539842	2.768519	-0.167358	F	23	C	7	0.274197	-0.353137	0.115427	-0.407247	(3 -1)
XX	-0.621480	3.252096	0.831613	F	23	C	8	0.017811	0.077404	0.052416	0.000359	(3 -1)
XX	-2.436790	1.719211	0.809795	CAGE				0.004050	0.017577	-0.458023	0.000801	(3 3)
XX	-0.002057	3.204414	-0.015005	F	23	F	21	0.011972	0.059813	0.661000	0.001469	(3 -1)
XX	-1.106844	2.772650	-0.765190	F	24	C	7	0.274016	-0.346819	0.115960	-0.407515	(3 -1)
XX	-1.751067	2.432245	-0.786051	F	25	C	7	0.273926	-0.321787	0.116880	-0.407471	(3 -1)
XX	-3.250270	1.846530	-0.032957	RING				0.005630	0.025297	-1.590136	0.001278	(3 1)
XX	-3.364187	1.148839	-1.230503	RING				0.008466	0.041389	-1.404931	0.001857	(3 1)

```
XX -2.469792 -1.706739 -0.776866 CAGE         0.004157  0.018129 -0.474639  0.000822 (3 3)
XX -0.630994 -3.236458 -0.857118 F  26 C   9  0.017431  0.075796  0.062879  0.000369 (3 -1)
XX -1.545373 -2.762615  0.146939 F  26 C  10  0.274024 -0.354356  0.116273 -0.406859 (3 -1)
XX -2.655417 -2.091146 -1.315129 RING         0.005083  0.023079 -1.321338  0.001272 (3 1)
XX  0.001815 -3.212196 -0.013993 F  26 F  17  0.012277  0.060824  0.557612  0.001412 (3 -1)
XX -1.106721 -2.778696  0.740482 F  27 C  10  0.274312 -0.344693  0.115933 -0.408191 (3 -1)
XX -1.748184 -2.435155  0.774020 F  28 C  10  0.273705 -0.322962  0.117133 -0.406974 (3 -1)
XX -3.360048 -1.154354  1.237911 RING         0.008328  0.040599 -1.400202  0.001828 (3 1)
XX -3.255869 -1.813565  0.057931 RING         0.005795  0.025893 -1.528747  0.001295 (3 1)
XX -0.282424 -3.084151 -0.180881 RING         0.011748  0.065912 -1.921997  0.002348 (3 1)
XX  3.589961 -1.796509 -1.569974 C  29 C   9  0.013540  0.057195  0.143403  0.001333 (3 -1)
XX  4.504319 -0.888855 -0.205393 RING         0.007388  0.032964 -1.329565  0.001699 (3 1)
XX  4.293118 -0.050637 -1.508812 C  29 C  16  0.233424 -0.505514  0.005941 -0.179515 (3 -1)
XX  2.469181  1.709417 -0.781299 CAGE         0.004133  0.018020 -0.476185  0.000819 (3 3)
XX  3.246063  0.543622 -1.879833 C  30 C  16  0.237685 -0.524524  0.006761 -0.184731 (3 -1)
XX  2.670400 -0.817631 -1.560829 RING         0.007494  0.036157 -1.352930  0.001652 (3 1)
XX  4.505174  0.875334  0.235832 RING         0.007408  0.033090 -1.328779  0.001707 (3 1)
XX  4.126186  1.147583 -1.211263 C  31 C  16  0.230902 -0.494117  0.005591 -0.176263 (3 -1)
XX  3.031082  2.849344 -0.725625 C  31 F  21  0.016795  0.060743  0.071545  0.000231 (3 -1)
XX  3.582214  1.792022  1.583178 C  32 C   8  0.013318  0.056478  0.144768  0.001354 (3 -1)
XX  4.275077  0.042194  1.535844 C  32 C  15  0.233093 -0.504042  0.005864 -0.179054 (3 -1)
XX  3.219277 -0.543360  1.893070 C  33 C  15  0.237617 -0.524207  0.006772 -0.184636 (3 -1)
XX  2.650474  0.822810  1.562839 RING         0.007456  0.035927 -1.353573  0.001645 (3 1)
XX  4.103851 -1.154995  1.237273 C  34 C  15  0.230962 -0.494346  0.005729 -0.176353 (3 -1)
XX  3.015754 -2.856174  0.726395 C  34 F  17  0.017252  0.062006  0.069499  0.000154 (3 -1)
XX -3.590035  1.794252 -1.575796 C  35 C   7  0.013566  0.057418  0.142598  0.001346 (3 -1)
XX -4.497920  0.888574 -0.210034 RING         0.007407  0.033062 -1.322593  0.001706 (3 1)
XX -4.291679  0.049027 -1.511611 C  35 C  13  0.233458 -0.505627  0.005741 -0.179563 (3 -1)
XX -3.244409 -0.546479 -1.879197 C  36 C  13  0.237679 -0.524505  0.006838 -0.184722 (3 -1)
XX -2.666136  0.818386 -1.565251 RING         0.007446  0.035900 -1.358277  0.001644 (3 1)
```

XX -4.505187 -0.876819 0.232642 RING 0.007454 0.033344 -1.330088 0.001716 (3 1)
XX -4.126042 -1.148032 -1.209721 C 37 C 13 0.230929 -0.494264 0.005733 -0.176301 (3 -1)
XX -3.035856 -2.845567 -0.710764 C 37 F 26 0.016858 0.061004 0.073613 0.000231 (3 -1)
XX -3.586596 -1.793878 1.572528 C 38 C 10 0.013127 0.055623 0.146096 0.001344 (3 -1)
XX -4.279031 -0.041099 1.530494 C 38 C 12 0.233207 -0.504596 0.006163 -0.179209 (3 -1)
XX -2.585143 2.104141 1.344482 RING 0.004918 0.022161 -1.357561 0.001235 (3 1)
XX -3.222960 0.540625 1.894406 C 39 C 12 0.237704 -0.524570 0.006531 -0.184758 (3 -1)
XX -2.655608 -0.824109 1.559853 RING 0.007457 0.035916 -1.351640 0.001644 (3 1)
XX -4.102108 1.157188 1.237692 C 40 C 12 0.231099 -0.494868 0.005628 -0.176545 (3 -1)
XX -3.002538 2.863262 0.749147 C 40 F 23 0.017311 0.062048 0.064984 0.000129 (3 -1)
XX -5.071064 0.341924 -2.352013 H 41 C 35 0.276086 -0.936357 0.007570 -0.280547 (3 -1)
XX -5.486851 0.013311 0.014615 H 41 C 38 0.005565 0.020373 0.121710 0.001167 (3 -1)
XX -5.364009 0.550902 -1.318254 H 42 C 35 0.278673 -0.953458 0.009027 -0.285546 (3 -1)
XX -5.291836 0.341867 0.154179 RING 0.005240 0.019521 -1.306921 0.001001 (3 1)
XX -4.603647 1.182690 -1.810678 H 43 C 35 0.281796 -0.984938 0.008921 -0.291389 (3 -1)
XX -2.350922 1.090123 -2.032102 H 44 C 7 0.009549 0.040552 0.225134 0.001392 (3 -1)
XX -3.112160 -0.800688 -3.029573 H 44 C 36 0.276872 -0.943557 0.007222 -0.282114 (3 -1)
XX -1.996313 -2.156905 -1.495527 H 45 F 26 0.007278 0.031505 0.030715 0.001350 (3 -1)
XX -2.422011 -0.054367 -2.606905 H 45 C 36 0.278315 -0.954622 0.008400 -0.284667 (3 -1)
XX -2.305333 -1.124652 -2.359566 H 46 C 36 0.278606 -0.955116 0.008166 -0.285257 (3 -1)
XX -4.758584 -1.977607 -1.762542 H 47 C 37 0.275333 -0.929097 0.010153 -0.279262 (3 -1)
XX -4.108084 -2.332839 -0.950135 H 48 C 37 0.284554 -1.005320 0.010312 -0.297177 (3 -1)
XX -5.030498 -1.774049 -0.720623 H 49 C 37 0.280945 -0.971857 0.010820 -0.289968 (3 -1)
XX -1.972400 2.148278 1.481172 H 50 F 22 0.006481 0.028500 0.060442 0.001340 (3 -1)
XX -4.747910 1.972114 1.795592 H 50 C 40 0.275312 -0.928848 0.010076 -0.279237 (3 -1)
XX -5.326421 1.200682 -0.312294 H 50 H 41 0.012462 0.047901 0.324751 0.002305 (3 -1)
XX -4.063937 2.349354 1.021838 H 51 C 40 0.284563 -1.005487 0.010240 -0.297192 (3 -1)
XX -5.316861 -0.310769 -0.124627 RING 0.005267 0.019589 -1.340344 0.001013 (3 1)
XX -4.982492 1.808722 0.737323 H 52 C 40 0.281025 -0.972767 0.010829 -0.290173 (3 -1)
XX -5.048088 -0.338312 2.379057 H 53 C 38 0.276184 -0.937024 0.007533 -0.280730 (3 -1)

XX -5.351774 -1.163115 0.331693 H 53 H 47 0.012342 0.047680 0.349798 0.002334 (3 -1)
XX -5.362286 -0.523517 1.347068 H 54 C 38 0.278633 -0.953223 0.008998 -0.285462 (3 -1)
XX -4.603288 -1.175918 1.814201 H 55 C 38 0.281661 -0.983960 0.008946 -0.291105 (3 -1)
XX -2.336510 -1.107017 2.036649 H 56 C 10 0.009736 0.041164 0.217105 0.001376 (3 -1)
XX -3.079253 0.788278 3.045092 H 56 C 39 0.276865 -0.943535 0.007167 -0.282099 (3 -1)
XX -2.403524 0.032928 2.614474 H 57 C 39 0.278449 -0.955737 0.008318 -0.284943 (3 -1)
XX -2.271697 1.102277 2.371922 H 58 C 39 0.278343 -0.952903 0.008232 -0.284753 (3 -1)
XX 2.349982 -1.093420 -2.039099 H 59 C 9 0.009760 0.041272 0.219304 0.001379 (3 -1)
XX 3.114723 0.797880 -3.030408 H 59 C 30 0.276859 -0.943477 0.007253 -0.282091 (3 -1)
XX 1.994585 2.153843 -1.496263 H 60 F 21 0.007159 0.031058 0.033985 0.001350 (3 -1)
XX 2.426841 0.048607 -2.609070 H 60 C 30 0.278421 -0.955457 0.008397 -0.284882 (3 -1)
XX 2.305492 1.118044 -2.361480 H 61 C 30 0.278598 -0.954958 0.008178 -0.285250 (3 -1)
XX 4.769551 1.969095 -1.763323 H 62 C 31 0.275326 -0.929028 0.010113 -0.279252 (3 -1)
XX 4.104160 2.335981 -0.968439 H 63 C 31 0.284466 -1.004713 0.010326 -0.297000 (3 -1)
XX 5.021893 1.780196 -0.713782 H 64 C 31 0.280953 -0.972015 0.010827 -0.290006 (3 -1)
XX 5.304810 -0.324926 0.156130 RING 0.005200 0.019310 -1.333814 0.000994 (3 1)
XX 5.069225 -0.350286 -2.350011 H 65 C 29 0.276083 -0.936285 0.007631 -0.280543 (3 -1)
XX 5.481318 -0.005250 0.019833 H 65 C 32 0.005481 0.020043 0.149063 0.001145 (3 -1)
XX 5.368209 -0.547120 -1.315767 H 66 C 29 0.278650 -0.953309 0.009107 -0.285509 (3 -1)
XX 4.607242 -1.186512 -1.797123 H 67 C 29 0.281834 -0.985248 0.008973 -0.291464 (3 -1)
XX 5.045101 0.332763 2.385775 H 68 C 32 0.276143 -0.936709 0.007596 -0.280649 (3 -1)
XX 5.346650 1.172619 0.339969 H 68 H 62 0.012438 0.047784 0.323256 0.002303 (3 -1)
XX 5.313485 0.309509 -0.116609 RING 0.005214 0.019360 -1.351909 0.000999 (3 1)
XX 5.354827 0.534016 1.355627 H 69 C 32 0.278731 -0.953963 0.009044 -0.285667 (3 -1)
XX 4.593081 1.175112 1.833898 H 70 C 32 0.281698 -0.984262 0.008981 -0.291194 (3 -1)
XX 1.974246 -2.151390 1.480627 H 71 F 27 0.006697 0.029343 0.047484 0.001347 (3 -1)
XX 4.731783 -1.980655 1.800259 H 71 C 34 0.275379 -0.929392 0.010133 -0.279369 (3 -1)
XX 5.342741 -1.186767 -0.297558 H 71 H 65 0.012260 0.047215 0.332833 0.002307 (3 -1)
XX 4.077658 -2.342951 0.994097 H 72 C 34 0.284624 -1.005996 0.010280 -0.297322 (3 -1)
XX 5.003596 -1.793252 0.755180 H 73 C 34 0.280859 -0.971357 0.010852 -0.289819 (3 -1)

XX 2.333168 1.101351 2.028801 H 74 C   8 0.009611 0.040752 0.220829 0.001385 (3 -1)
XX 3.072123 -0.790641 3.043479 H 74 C 33 0.276902 -0.943799 0.007166 -0.282166 (3 -1)
XX 2.393552 -0.040281 2.608971 H 75 C 33 0.278359 -0.954931 0.008318 -0.284752 (3 -1)
XX 2.269446 -1.110843 2.367146 H 76 C 33 0.278392 -0.953369 0.008208 -0.284838 (3 -1)

M7d Singlet state
XX 1.050986 -0.339312 0.537026 C   2 C   1 0.238689 -0.511822 0.044631 -0.187515 (3 -1)
XX 0.077481 -0.732155 1.182505 C   3 C   2 0.333047 -0.899966 0.386116 -0.358060 (3 -1)
XX 0.000271 -0.678945 0.002069 RING   0.025404 0.173333 -1.167853 0.008060 (3 1)
XX -0.965665 -0.322526 0.641019 C   4 C   3 0.246713 -0.548964 0.042015 -0.199175 (3 -1)
XX -1.049956 -0.340041 -0.535310 C   5 C   4 0.238943 -0.512868 0.044629 -0.187893 (3 -1)
XX 0.966877 -0.330862 -0.640017 C   6 C   1 0.246514 -0.548143 0.041740 -0.198866 (3 -1)
XX -0.078112 -0.741325 -1.177819 C   6 C   5 0.332926 -0.899252 0.386045 -0.357781 (3 -1)
XX 1.156691 -1.193499 1.504727 C   7 C   2 0.258103 -0.640834 0.046317 -0.216849 (3 -1)
XX -0.018362 -2.250297 1.456474 RING   0.008321 0.046370 -1.280048 0.002222 (3 1)
XX -1.002422 -1.169325 1.582187 C   8 C   3 0.257788 -0.640140 0.037215 -0.216250 (3 -1)
XX 0.021255 -2.261057 -1.447853 RING   0.008379 0.046738 -1.283140 0.002243 (3 1)
XX -1.159137 -1.202585 -1.495150 C   9 C   5 0.257892 -0.639779 0.046316 -0.216519 (3 -1)
XX 0.999256 -1.185824 -1.574533 C 10 C   6 0.257845 -0.640437 0.037483 -0.216349 (3 -1)
XX 1.712526 0.573735 -0.122479 C 11 C   1 0.319006 -0.827425 0.380566 -0.329807 (3 -1)
XX 1.645005 0.536389 1.361046 RING   0.010981 0.052828 -1.486385 0.002796 (3 1)
XX 1.373950 0.550173 -1.555235 RING   0.011699 0.057978 -1.439176 0.003168 (3 1)
XX -0.146496 1.474745 -1.173831 CAGE   0.002060 0.007218 -0.556095 0.000360 (3 3)
XX 2.195190 1.522904 -0.880356 C 12 C 11 0.221604 -0.455584 0.027048 -0.162897 (3 -1)
XX 2.771888 -0.059348 1.187799 CAGE   0.004868 0.022305 -0.484090 0.001032 (3 3)
XX 2.493352 1.433226 0.476131 C 13 C 11 0.222549 -0.460082 0.028331 -0.164211 (3 -1)
XX 3.736223 1.895905 -0.308651 RING   0.008109 0.036774 -1.233742 0.001913 (3 1)
XX -1.373775 0.563555 1.551843 RING   0.011686 0.057851 -1.441084 0.003162 (3 1)
XX -1.709490 0.579119 0.117171 C 14 C   4 0.319224 -0.828584 0.380908 -0.330260 (3 -1)
XX 0.140889 1.455454 1.168192 CAGE   0.002149 0.007537 -0.570579 0.000370 (3 3)

XX 0.544551 2.370805 0.299673 RING 0.002138 0.006797 -1.305039 0.000369 (3 1)
XX -0.242050 1.800149 -0.671550 RING 0.002255 0.006654 -2.294740 0.000351 (3 1)
XX 0.233402 1.771816 0.648629 RING 0.002366 0.006950 -2.188151 0.000360 (3 1)
XX -0.015399 2.092464 -0.044116 CAGE 0.001935 0.005786 -0.722810 0.000310 (3 3)
XX -2.189208 1.534567 0.867837 C 15 C 14 0.221820 -0.456405 0.027431 -0.163186 (3 -1)
XX -2.770654 -0.067389 -1.190458 CAGE 0.004842 0.022122 -0.455464 0.001023 (3 3)
XX -1.647463 0.526159 -1.369199 RING 0.010921 0.052383 -1.492524 0.002772 (3 1)
XX -2.490293 1.434584 -0.486432 C 16 C 14 0.222928 -0.461575 0.028552 -0.164719 (3 -1)
XX -3.724555 1.911575 0.297901 RING 0.008175 0.037153 -1.228523 0.001935 (3 1)
XX -1.126618 -2.201227 -0.083009 RING 0.003473 0.017796 -1.361118 0.001101 (3 1)
XX -1.487728 -2.187896 -1.780097 F 17 C 9 0.275205 -0.363225 0.119674 -0.409651 (3 -1)
XX 0.788478 0.236778 -2.122916 F 17 C 12 0.018618 0.063205 0.458295 0.002136 (3 -1)
XX -2.059099 -1.725295 -1.753949 F 18 C 9 0.271485 -0.335664 0.116283 -0.401906 (3 -1)
XX -0.180416 -1.532665 -2.522176 RING 0.007173 0.042258 -1.251925 0.002302 (3 1)
XX -1.604998 -1.717840 -2.338629 F 19 C 9 0.271324 -0.367458 0.116700 -0.400566 (3 -1)
XX -2.949863 -0.187363 -0.824369 RING 0.005070 0.022028 -1.197995 0.001104 (3 1)
XX -2.058658 -0.478638 -2.916439 F 19 C 16 0.015831 0.059621 0.139143 0.000574 (3 -1)
XX 0.164589 -1.498446 2.540977 RING 0.007104 0.041816 -1.247367 0.002276 (3 1)
XX -1.908221 -1.604837 1.967435 F 20 C 8 0.271148 -0.345043 0.114777 -0.400981 (3 -1)
XX -1.431611 -2.131012 1.777131 F 21 C 8 0.273217 -0.374518 0.124446 -0.404948 (3 -1)
XX -1.308654 -2.909809 -0.096644 F 21 F 17 0.005408 0.028504 0.036917 0.001212 (3 -1)
XX -1.343929 -1.765125 2.416548 F 22 C 8 0.274975 -0.338257 0.120441 -0.409109 (3 -1)
XX 1.513968 0.210199 1.892591 F 22 C 13 0.013160 0.050679 1.661326 0.002397 (3 -1)
XX -1.743877 -0.256718 2.307913 RING 0.009245 0.045921 -1.444940 0.002634 (3 1)
XX 1.126251 -2.188286 0.099699 RING 0.003561 0.018228 -1.354746 0.001118 (3 1)
XX 1.486630 -2.177229 1.793992 F 23 C 7 0.275177 -0.364135 0.119557 -0.409561 (3 -1)
XX -0.065437 -2.936576 1.508068 F 23 F 21 0.013177 0.056986 0.029439 0.000428 (3 -1)
XX 2.055807 -1.711701 1.774501 F 24 C 7 0.271580 -0.334474 0.116309 -0.402125 (3 -1)
XX 1.594699 -1.705740 2.353438 F 25 C 7 0.271313 -0.367107 0.116838 -0.400550 (3 -1)
XX 2.947467 -0.179963 0.836219 RING 0.005071 0.022096 -1.192785 0.001105 (3 1)

XX 2.040486 -0.463266 2.917699 F 25 C 13 0.015432 0.058599 0.163621 0.000650 (3 -1)
XX -0.792317 0.246787 2.125517 F 25 C 15 0.018705 0.063215 0.436892 0.002099 (3 -1)
XX 0.219686 -1.741439 3.222273 F 25 F 22 0.011690 0.051514 0.024714 0.000586 (3 -1)
XX 1.428613 -2.149814 -1.757451 F 26 C 10 0.273169 -0.375448 0.124613 -0.404822 (3 -1)
XX 0.070309 -2.946562 -1.485106 F 26 F 17 0.013163 0.056983 0.024931 0.000435 (3 -1)
XX 1.313582 -2.912251 0.119226 F 26 F 23 0.005723 0.029767 0.034834 0.001193 (3 -1)
XX 1.331104 -1.795484 -2.402082 F 27 C 10 0.275013 -0.337492 0.120787 -0.409216 (3 -1)
XX 1.731861 -0.276852 -2.305232 RING 0.009320 0.046281 -1.450379 0.002659 (3 1)
XX -1.515821 0.203862 -1.891398 F 27 C 16 0.013013 0.050047 1.589543 0.002366 (3 -1)
XX -0.242638 -1.770578 -3.204622 F 27 F 19 0.011701 0.051594 0.020883 0.000589 (3 -1)
XX 1.901537 -1.626409 -1.964133 F 28 C 10 0.271011 -0.345477 0.114663 -0.400664 (3 -1)
XX -0.848385 3.279728 -1.406399 C 29 C 12 0.003936 0.013591 0.119961 0.000891 (3 -1)
XX -0.519107 2.343847 -0.350369 RING 0.002084 0.006593 -1.316434 0.000360 (3 1)
XX -2.313609 2.840635 0.147988 RING 0.009354 0.044325 -1.238649 0.002329 (3 1)
XX -2.929270 2.483890 -1.124242 C 29 C 16 0.233460 -0.505495 0.006552 -0.179076 (3 -1)
XX -3.146243 -0.093054 -1.685592 RING 0.005637 0.026496 -1.230691 0.001448 (3 1)
XX -2.373140 0.693425 -2.644139 C 29 F 19 0.286598 -1.014298 0.012403 -0.301183 (3 -1)
XX -0.209032 1.228659 -2.655029 C 29 F 27 0.008790 0.030335 0.084297 0.001362 (3 -1)
XX -1.832389 -0.356246 -2.213094 RING 0.011578 0.054068 -2.453334 0.002991 (3 1)
XX -0.001746 1.906661 -2.201529 RING 0.004411 0.015188 -1.489330 0.000907 (3 1)
XX -2.558119 1.509162 -1.776475 C 30 C 16 0.238321 -0.526743 0.004793 -0.185812 (3 -1)
XX -3.604990 1.410933 -1.076351 C 31 C 16 0.232215 -0.500776 0.007103 -0.178295 (3 -1)
XX -3.006621 2.134826 1.665025 C 32 C 15 0.234491 -0.510460 0.005913 -0.180675 (3 -1)
XX -3.143627 2.602080 0.312579 RING 0.008990 0.042476 -1.150909 0.002258 (3 1)
XX -4.456977 2.052005 0.459971 C 32 C 31 0.011649 0.047683 0.523433 0.002585 (3 -1)
XX 0.198500 1.242732 2.643383 C 33 C 13 0.008611 0.029910 0.087903 0.001375 (3 -1)
XX -2.031091 1.575101 2.175248 C 33 C 15 0.236948 -0.519870 0.003499 -0.184029 (3 -1)
XX -2.533417 -0.307605 2.583399 C 33 F 20 0.016479 0.064002 0.112294 0.000923 (3 -1)
XX 0.990512 2.798948 1.631431 RING 0.003316 0.011215 -1.478482 0.000651 (3 1)
XX -1.814511 2.572221 1.469486 C 34 C 15 0.232231 -0.500416 0.009187 -0.178162 (3 -1)

XX 0.297605 0.708962 2.250042 RING 0.005916 0.020236 -1.447678 0.001036 (3 1)
XX -0.834769 2.578617 -0.576251 CAGE 0.002012 0.006986 -0.310931 0.000377 (3 3)
XX 2.330906 2.833314 -0.173464 RING 0.009381 0.044474 -1.242310 0.002335 (3 1)
XX 2.935666 2.486673 1.105356 C 35 C 13 0.233653 -0.506303 0.006239 -0.179347 (3 -1)
XX 3.143077 -0.074008 1.686013 RING 0.005660 0.026704 -1.231036 0.001453 (3 1)
XX 2.359977 0.712970 2.637704 C 35 F 25 0.286518 -1.013548 0.012236 -0.301030 (3 -1)
XX 0.010909 3.041734 -0.018921 C 35 C 29 0.003063 0.009696 1.078222 0.000558 (3 -1)
XX -0.415034 2.225009 -1.963688 C 35 C 30 0.005453 0.019258 0.228352 0.001171 (3 -1)
XX 0.425054 2.230766 1.931380 C 35 C 32 0.005857 0.020744 0.186460 0.001232 (3 -1)
XX 0.867293 3.276161 1.368480 C 35 C 34 0.003962 0.013720 0.136142 0.000896 (3 -1)
XX 1.835403 -0.354379 2.225497 RING 0.011672 0.054697 -2.481873 0.002996 (3 1)
XX 2.554421 1.519903 1.764974 C 36 C 13 0.238442 -0.527315 0.005054 -0.185979 (3 -1)
XX -0.018388 1.895579 2.192328 RING 0.004502 0.015611 -1.443790 0.000929 (3 1)
XX 2.476524 -0.512461 2.228447 C 36 F 24 0.011389 0.052277 4.583725 0.001916 (3 -1)
XX 3.606637 1.412184 1.072233 C 37 C 13 0.231917 -0.499577 0.007123 -0.177864 (3 -1)
XX 3.018307 2.104255 -1.683067 C 38 C 12 0.234993 -0.512663 0.005911 -0.181389 (3 -1)
XX 2.520740 -0.334934 -2.593793 C 38 F 27 0.016947 0.065306 0.105846 0.000835 (3 -1)
XX 0.755490 3.294776 0.517376 RING 0.002487 0.008052 -1.738598 0.000455 (3 1)
XX 3.150582 2.596866 -0.338104 RING 0.008929 0.042089 -1.149858 0.002234 (3 1)
XX -0.309053 0.690008 -2.252923 RING 0.005933 0.020280 -1.442575 0.001039 (3 1)
XX 2.032652 1.555282 -2.187297 C 39 C 12 0.236804 -0.519224 0.003395 -0.183830 (3 -1)
XX -0.969112 2.782625 -1.675285 RING 0.003235 0.010867 -1.471106 0.000633 (3 1)
XX 1.833485 2.562220 -1.492473 C 40 C 12 0.232024 -0.499550 0.009034 -0.177862 (3 -1)
XX 0.857262 2.606645 0.527954 CAGE 0.002061 0.007162 -0.339918 0.000383 (3 3)
XX -3.408777 3.389216 -1.710951 H 41 C 29 0.277406 -0.946090 0.007464 -0.282881 (3 -1)
XX -3.242704 3.563066 -0.644113 H 42 C 29 0.283756 -0.991803 0.008905 -0.295394 (3 -1)
XX -2.388576 3.536819 -1.336766 H 43 C 29 0.277132 -0.943138 0.009868 -0.282421 (3 -1)
XX -2.227572 3.634995 -0.044089 H 43 C 34 0.015053 0.062904 1.386446 0.002869 (3 -1)
XX 0.732856 3.019974 -1.424196 H 43 C 38 0.277827 -0.948892 0.010617 -0.283874 (3 -1)
XX -3.667722 -0.618174 -1.219449 H 44 F 18 0.012075 0.048321 0.028923 0.001010 (3 -1)

XX -4.618938 1.275284 -1.667229 H 44 C 31 0.275287 -0.931586 0.010422 -0.279187 (3 -1)
XX -1.592698 1.460128 -2.485022 H 44 C 39 0.278558 -0.953648 0.011225 -0.285546 (3 -1)
XX -4.311131 0.441755 -1.006324 H 45 C 31 0.281967 -0.990077 0.010128 -0.291870 (3 -1)
XX -4.699489 1.384425 -0.576091 H 46 C 31 0.280840 -0.972543 0.011520 -0.289692 (3 -1)
XX -2.555771 1.722605 -2.935497 H 47 C 30 0.276890 -0.940386 0.009852 -0.282150 (3 -1)
XX -1.168497 1.038970 2.826671 H 50 F 22 0.281643 -0.977003 0.012836 -0.291912 (3 -1)
XX -1.432739 3.556122 2.013389 H 50 C 34 0.275753 -0.934969 0.009283 -0.280028 (3 -1)
XX -0.737943 3.304339 -0.561811 RING 0.002487 0.008031 -1.751171 0.000456 (3 1)
XX -0.707813 3.010695 1.388322 H 51 C 34 0.277952 -0.949821 0.010414 -0.284105 (3 -1)
XX -1.497674 3.611689 0.917186 H 52 C 34 0.284394 -1.000032 0.010336 -0.296715 (3 -1)
XX -1.860278 1.717076 3.326332 H 53 C 33 0.276588 -0.937419 0.010127 -0.281683 (3 -1)
XX -2.217845 0.730528 3.012534 H 54 C 33 0.283840 -0.997402 0.011598 -0.295200 (3 -1)
XX -3.738975 2.669939 2.423573 H 56 C 32 0.276776 -0.941935 0.008754 -0.281822 (3 -1)
XX -3.750168 3.280739 0.379761 H 56 H 41 0.017206 0.059236 0.097508 0.001527 (3 -1)
XX -4.034084 2.715961 1.371130 H 57 C 32 0.283012 -0.985892 0.009890 -0.294118 (3 -1)
XX -4.115434 1.764890 1.920540 H 58 C 32 0.277516 -0.950743 0.009510 -0.283130 (3 -1)
XX 3.416056 3.394669 1.686255 H 59 C 35 0.277490 -0.946723 0.007243 -0.283053 (3 -1)
XX 3.262772 3.556052 0.615619 H 60 C 35 0.283651 -0.991055 0.008676 -0.295172 (3 -1)
XX 2.400730 3.545996 1.299985 H 61 C 35 0.277244 -0.944065 0.009656 -0.282636 (3 -1)
XX 3.669531 -0.609027 1.230905 H 62 F 24 0.012072 0.048488 0.035077 0.001034 (3 -1)
XX 1.583247 1.480685 2.465293 H 62 C 33 0.278715 -0.954837 0.011061 -0.285838 (3 -1)
XX 4.613585 1.272573 1.674387 H 62 C 37 0.275286 -0.931519 0.010539 -0.279173 (3 -1)
XX 4.315712 0.442836 1.004867 H 63 C 37 0.281989 -0.990306 0.010331 -0.291932 (3 -1)
XX 4.471051 2.042799 -0.463036 H 63 C 38 0.011528 0.047059 0.497994 0.002560 (3 -1)
XX 4.707424 1.388770 0.585288 H 64 C 37 0.280662 -0.971116 0.011646 -0.289316 (3 -1)
XX 2.543164 1.744207 2.922253 H 65 C 36 0.276827 -0.940030 0.009801 -0.282015 (3 -1)
XX 2.407674 -0.532465 2.252945 RING 0.011383 0.054250 -5.999998 0.002164 (3 1)
XX 1.158869 1.027720 -2.827986 H 68 F 27 0.281771 -0.977856 0.012610 -0.292186 (3 -1)
XX 1.471924 3.547285 -2.047015 H 68 C 40 0.275738 -0.934693 0.009402 -0.279997 (3 -1)
XX 1.528455 3.611992 -0.951292 H 70 C 40 0.284668 -1.002154 0.010467 -0.297288 (3 -1)

XX 2.245075 3.634528 0.017451 H 70 H 61 0.015215 0.063597 1.461893 0.002875 (3 -1)
XX 1.858315 1.689753 -3.338969 H 71 C 39 0.276636 -0.937802 0.010085 -0.281780 (3 -1)
XX 2.201718 0.700302 -3.018452 H 72 C 39 0.284022 -0.998809 0.011447 -0.295575 (3 -1)
XX 3.759561 2.612866 -2.450580 H 74 C 38 0.276793 -0.942093 0.008666 -0.281853 (3 -1)
XX 3.770728 3.258741 -0.409236 H 74 H 59 0.016946 0.058411 0.100423 0.001540 (3 -1)
XX 4.050378 2.679335 -1.398022 H 75 C 38 0.282718 -0.983588 0.009816 -0.293519 (3 -1)
XX 4.123271 1.714891 -1.925438 H 76 C 38 0.277567 -0.951326 0.009400 -0.283235 (3 -1)

M7e Triplet state
XX -1.092568 0.588204 -0.008789 C 2 C 1 0.302414 -0.793599 0.207169 -0.293505 (3 -1)
XX 0.000025 0.002325 -0.009203 RING 0.019254 0.146233 -1.201491 0.008185 (3 1)
XX -0.000045 1.203315 -0.008292 C 3 C 2 0.298845 -0.775852 0.220250 -0.285299 (3 -1)
XX 1.092423 0.587997 -0.006131 C 4 C 3 0.302443 -0.793750 0.207128 -0.293545 (3 -1)
XX 1.092714 -0.583514 -0.011998 C 5 C 4 0.302449 -0.793717 0.207368 -0.293592 (3 -1)
XX -1.092468 -0.583320 -0.006587 C 6 C 1 0.302410 -0.793496 0.207293 -0.293501 (3 -1)
XX 0.000147 -1.198569 -0.012086 C 6 C 5 0.298765 -0.775455 0.220057 -0.285148 (3 -1)
XX -1.027706 1.790452 -0.011269 C 7 C 2 0.282165 -0.762425 0.068991 -0.268797 (3 -1)
XX 1.027939 1.790018 -0.004893 C 8 C 3 0.282192 -0.762599 0.069066 -0.268858 (3 -1)
XX 1.027528 -1.785894 -0.022677 C 9 C 5 0.282131 -0.762213 0.069006 -0.268744 (3 -1)
XX -1.027399 -1.785571 -0.005581 C 10 C 6 0.282192 -0.762559 0.069075 -0.268865 (3 -1)
XX -2.244139 0.002075 -0.002661 C 11 C 1 0.259836 -0.609855 0.051035 -0.222188 (3 -1)
XX -2.695994 1.690768 0.764638 RING 0.005165 0.017434 -1.460699 0.000829 (3 1)
XX -3.314132 -0.041661 0.712565 C 12 C 11 0.232879 -0.489915 0.068503 -0.179455 (3 -1)
XX -2.699085 -1.681444 -0.761344 RING 0.005223 0.017685 -1.450938 0.000840 (3 1)
XX -3.321278 0.042400 -0.707318 C 13 C 11 0.232895 -0.489971 0.068519 -0.179476 (3 -1)
XX -4.775685 -0.001906 0.012319 RING 0.006596 0.028633 -1.196725 0.001488 (3 1)
XX 2.244173 0.003338 -0.002825 C 14 C 4 0.259812 -0.609760 0.050882 -0.222150 (3 -1)
XX 2.673506 -1.676519 0.757512 RING 0.005242 0.017829 -1.442123 0.000847 (3 1)
XX 3.311801 0.040273 0.715368 C 15 C 14 0.232806 -0.489598 0.068475 -0.179342 (3 -1)
XX 2.724710 1.701787 -0.751906 RING 0.005156 0.017338 -1.466429 0.000823 (3 1)

XX 3.323622 -0.035931 -0.705023 C 16 C 14 0.232916 -0.490067 0.068478 -0.179514 (3 -1)
XX 4.777406 -0.023234 0.014646 RING   0.006573 0.028485 -1.197879 0.001479 (3 1)
XX 4.259374 -0.579716 -1.369554 C 17 C 16 0.236957 -0.521629 0.004690 -0.184246 (3 -1)
XX 1.771720 -1.479932 -1.274486 RING   0.007383 0.028888 -3.142381 0.001729 (3 1)
XX 1.899020 -1.783761 -1.319049 C 18 C   9 0.007535 0.028397 1.342613 0.001644 (3 -1)
XX 3.176090 -0.288016 -1.930383 C 18 C 16 0.237949 -0.525556 0.002649 -0.185209 (3 -1)
XX 3.949378 0.633058 -1.578421 C 19 C 16 0.232798 -0.502909 0.006831 -0.179045 (3 -1)
XX 4.257603 0.553466 1.391787 C 20 C 15 0.237289 -0.523175 0.004506 -0.184668 (3 -1)
XX 4.415361 0.960844 -0.134333 RING   0.007310 0.032301 -1.282433 0.001662 (3 1)
XX 1.771002 1.469749 1.245229 RING   0.007378 0.028979 -2.733178 0.001731 (3 1)
XX 1.921145 1.842096 1.294372 C 21 C   8 0.007653 0.028692 1.003965 0.001651 (3 -1)
XX 3.152980 0.316202 1.935151 C 21 C 15 0.237779 -0.524760 0.002556 -0.184995 (3 -1)
XX 2.619914 -2.317052 1.047102 C 22 C   1 0.006682 0.022830 0.430801 0.001274 (3 -1)
XX 3.891409 -0.641717 1.608887 C 22 C 15 0.232863 -0.503087 0.006765 -0.179154 (3 -1)
XX 4.382569 -1.007288 0.166496 RING   0.007168 0.031342 -1.282950 0.001609 (3 1)
XX -4.267896 0.570385 -1.370510 C 23 C 13 0.237144 -0.522484 0.004621 -0.184483 (3 -1)
XX -1.777818 1.469480 -1.256550 RING   0.007383 0.028981 -2.787861 0.001731 (3 1)
XX -1.924904 1.832135 -1.304741 C 24 C   7 0.007638 0.028660 1.041158 0.001649 (3 -1)
XX -3.175211 0.314140 -1.929298 C 24 C 13 0.237898 -0.525298 0.002561 -0.185151 (3 -1)
XX -2.659971 -2.332367 -1.052199 C 25 C   4 0.006688 0.022737 0.487839 0.001257 (3 -1)
XX -3.925265 -0.631135 -1.591344 C 25 C 13 0.232760 -0.502687 0.006846 -0.179006 (3 -1)
XX -4.250463 -0.576784 1.384164 C 26 C 12 0.237091 -0.522272 0.004587 -0.184410 (3 -1)
XX -4.395090 -0.987675 -0.147776 RING   0.007251 0.031879 -1.282469 0.001638 (3 1)
XX -1.764366 -1.476903 1.250342 RING   0.007383 0.028942 -2.906198 0.001731 (3 1)
XX -1.904204 -1.819064 1.296972 C 27 C 10 0.007597 0.028549 1.138798 0.001648 (3 -1)
XX -3.154739 -0.311893 1.933361 C 27 C 12 0.237883 -0.525231 0.002527 -0.185130 (3 -1)
XX -3.914062 0.627685 1.602373 C 28 C 12 0.232797 -0.502868 0.006867 -0.179052 (3 -1)
XX -4.402661 0.977572 0.163458 RING   0.007247 0.031875 -1.280565 0.001640 (3 1)
XX -5.127972 1.072005 -2.007510 H 29 C 23 0.276091 -0.938514 0.006638 -0.280602 (3 -1)
XX -5.352321 0.872687 -0.950890 H 30 C 23 0.278839 -0.956928 0.008010 -0.285921 (3 -1)

XX -4.661161 1.694627 -1.220663 H 31 C 23 0.278541 -0.961262 0.007731 -0.284963 (3 -1)
XX -3.040115 0.553567 -3.084695 H 32 C 24 0.277204 -0.948329 0.006126 -0.282753 (3 -1)
XX -2.473661 1.183993 -2.381454 H 33 C 24 0.278020 -0.952722 0.008508 -0.283993 (3 -1)
XX -2.167632 0.136405 -2.562468 H 34 C 24 0.276460 -0.938138 0.008226 -0.281249 (3 -1)
XX -4.477517 -1.264266 -2.426434 H 35 C 25 0.275307 -0.932177 0.008037 -0.279149 (3 -1)
XX -3.669824 -1.773199 -1.870270 H 36 C 25 0.279024 -0.963094 0.009428 -0.286038 (3 -1)
XX -4.981875 -1.365104 -0.137817 H 36 C 26 0.009726 0.039305 1.093269 0.002251 (3 -1)
XX -4.641194 -1.579121 -1.381379 H 37 C 25 0.280785 -0.975557 0.009365 -0.289573 (3 -1)
XX -4.464439 1.257002 2.441634 H 38 C 28 0.275318 -0.932291 0.008043 -0.279176 (3 -1)
XX -4.995608 1.349189 0.162251 H 38 H 29 0.009742 0.039362 1.023280 0.002256 (3 -1)
XX -3.662163 1.770172 1.881201 H 39 C 28 0.278967 -0.962671 0.009418 -0.285928 (3 -1)
XX -4.635715 1.572788 1.398223 H 40 C 28 0.280844 -0.976047 0.009366 -0.289708 (3 -1)
XX -5.097430 -1.090234 2.029735 H 41 C 26 0.276037 -0.938095 0.006701 -0.280497 (3 -1)
XX -5.533807 -0.006693 0.015395 H 41 H 29 0.009588 0.035074 0.136674 0.001749 (3 -1)
XX -5.338969 -0.885621 0.978056 H 42 C 26 0.278678 -0.955796 0.008134 -0.285579 (3 -1)
XX -4.636050 -1.702996 1.232159 H 43 C 26 0.278593 -0.961664 0.007724 -0.285057 (3 -1)
XX -3.006743 -0.546311 3.088076 H 44 C 27 0.277260 -0.948720 0.006116 -0.282867 (3 -1)
XX -2.444007 -1.176796 2.382074 H 45 C 27 0.277999 -0.952572 0.008563 -0.283959 (3 -1)
XX -2.657010 2.332646 1.051200 H 46 C 7 0.006538 0.022167 0.490385 0.001229 (3 -1)
XX -2.141739 -0.126921 2.555612 H 46 C 27 0.276427 -0.937821 0.008253 -0.281174 (3 -1)
XX 3.039094 -0.509263 -3.089398 H 47 C 18 0.277256 -0.948776 0.006069 -0.282849 (3 -1)
XX 2.453160 -1.132894 -2.396444 H 48 C 18 0.277969 -0.952086 0.008449 -0.283908 (3 -1)
XX 3.194307 -2.135434 -0.658958 RING 0.003114 0.010137 -2.684883 0.000516 (3 1)
XX 2.176110 -0.075511 -2.564700 H 49 C 18 0.276432 -0.937996 0.008181 -0.281192 (3 -1)
XX 4.514786 1.261928 -2.407306 H 50 C 19 0.275314 -0.932131 0.008147 -0.279164 (3 -1)
XX 2.702519 2.363323 -1.034585 H 51 C 8 0.006591 0.022243 0.556651 0.001218 (3 -1)
XX 3.726258 1.786666 -1.837600 H 51 C 19 0.279103 -0.964154 0.009493 -0.286200 (3 -1)
XX 5.018664 1.329607 -0.117942 H 51 C 20 0.009908 0.040142 1.148965 0.002288 (3 -1)
XX 4.694932 1.555107 -1.359217 H 52 C 19 0.280856 -0.975821 0.009385 -0.289729 (3 -1)
XX 5.108133 -1.099031 -2.008095 H 53 C 17 0.276040 -0.938197 0.006677 -0.280517 (3 -1)

XX 5.334381 -0.913674 -0.949320 H 54 C 17 0.278718 -0.956044 0.008083 -0.285680 (3 -1)
XX 4.622332 -1.715050 -1.227133 H 55 C 17 0.278648 -0.962267 0.007725 -0.285180 (3 -1)
XX 5.111830 1.047133 2.042590 H 56 C 20 0.276094 -0.938447 0.006633 -0.280595 (3 -1)
XX 5.533485 -0.044912 0.025044 H 56 H 53 0.009518 0.034829 0.137883 0.001745 (3 -1)
XX 5.356397 0.834257 0.993354 H 57 C 20 0.278691 -0.955901 0.008073 -0.285598 (3 -1)
XX 4.671978 1.669325 1.240490 H 58 C 20 0.278580 -0.961410 0.007677 -0.285036 (3 -1)
XX 4.427583 -1.281975 2.449039 H 59 C 22 0.275346 -0.932577 0.007873 -0.279236 (3 -1)
XX 4.959005 -1.385154 0.166544 H 59 H 53 0.009515 0.038328 0.999007 0.002210 (3 -1)
XX 3.611388 -1.776388 1.892562 H 60 C 22 0.278950 -0.962193 0.009303 -0.285893 (3 -1)
XX 4.588016 -1.605529 1.405767 H 61 C 22 0.280765 -0.975666 0.009254 -0.289539 (3 -1)
XX 3.005541 0.559402 3.088300 H 62 C 21 0.277199 -0.948254 0.006177 -0.282747 (3 -1)
XX 2.458672 1.197968 2.376784 H 63 C 21 0.278024 -0.952936 0.008565 -0.284002 (3 -1)
XX 2.133999 0.155492 2.554527 H 64 C 21 0.276432 -0.937770 0.008268 -0.281179 (3 -1)
XX 1.573072 2.815357 0.002214 N 65 C 8 0.463984 0.273335 0.032555 -0.828905 (3 -1)
XX -1.572334 2.816035 -0.019375 N 66 C 7 0.463989 0.273822 0.032539 -0.828899 (3 -1)
XX 1.571322 -2.811756 -0.043711 N 67 C 9 0.463994 0.274219 0.032585 -0.828900 (3 -1)
XX -1.571322 -2.811495 0.006278 N 68 C 10 0.463987 0.273616 0.032609 -0.828904 (3 -1)

M7e Singlet state
XX -1.092384 0.588144 -0.008746 C 2 C 1 0.302487 -0.794081 0.207230 -0.293644 (3 -1)
XX 0.000028 0.002091 -0.008605 RING 0.019247 0.146178 -1.201264 0.008184 (3 1)
XX 0.000158 1.203180 -0.007895 C 3 C 2 0.298669 -0.774894 0.220539 -0.284998 (3 -1)
XX 1.092450 0.587605 -0.005582 C 4 C 3 0.302499 -0.794145 0.207181 -0.293656 (3 -1)
XX 1.092520 -0.583926 -0.011484 C 5 C 4 0.302512 -0.794160 0.207401 -0.293711 (3 -1)
XX -1.092481 -0.583392 -0.006001 C 6 C 1 0.302466 -0.793898 0.207318 -0.293610 (3 -1)
XX -0.000054 -1.198913 -0.011161 C 6 C 5 0.298604 -0.774570 0.220376 -0.284875 (3 -1)
XX -1.027611 1.790553 -0.010418 C 7 C 2 0.282091 -0.762028 0.069097 -0.268649 (3 -1)
XX 1.028373 1.789852 -0.004533 C 8 C 3 0.282116 -0.762184 0.069169 -0.268706 (3 -1)
XX 1.027455 -1.786472 -0.020293 C 9 C 5 0.282060 -0.761839 0.069110 -0.268602 (3 -1)
XX -1.027873 -1.785845 -0.005098 C 10 C 6 0.282118 -0.762161 0.069175 -0.268716 (3 -1)

```
XX -2.243622  0.002254 -0.002710 C  11 C   1  0.259960 -0.610364  0.051325 -0.222342 (3 -1)
XX -2.695552  1.691130  0.768489 RING         0.005157  0.017386 -1.464942  0.000827 (3 1)
XX -3.313991 -0.044298  0.712033 C  12 C  11  0.232892 -0.489990  0.068420 -0.179476 (3 -1)
XX -2.696869 -1.680476 -0.765582 RING         0.005219  0.017658 -1.453815  0.000839 (3 1)
XX -3.320675  0.045602 -0.707486 C  13 C  11  0.232914 -0.490069  0.068437 -0.179504 (3 -1)
XX -4.775478 -0.002473  0.011850 RING         0.006593  0.028612 -1.196472  0.001487 (3 1)
XX  2.243657  0.002586 -0.002826 C  14 C   4  0.259937 -0.610274  0.051181 -0.222306 (3 -1)
XX  2.675507 -1.678264  0.762339 RING         0.005227  0.017739 -1.448063  0.000843 (3 1)
XX  3.312024  0.043117  0.714498 C  15 C  14  0.232816 -0.489664  0.068396 -0.179362 (3 -1)
XX  2.719500  1.698571 -0.757660 RING         0.005158  0.017346 -1.468003  0.000825 (3 1)
XX  3.322695 -0.039931 -0.705523 C  16 C  14  0.232920 -0.490103  0.068398 -0.179520 (3 -1)
XX  4.777012 -0.019504  0.013516 RING         0.006572  0.028484 -1.197705  0.001479 (3 1)
XX  4.260217 -0.583362 -1.368441 C  17 C  16  0.237006 -0.521845  0.004671 -0.184306 (3 -1)
XX  1.769315 -1.471567 -1.269402 RING         0.007425  0.029106 -3.006932  0.001741 (3 1)
XX  1.905129 -1.800015 -1.315833 C  18 C   9  0.007616  0.028668  1.207501  0.001654 (3 -1)
XX  3.175170 -0.298995 -1.929669 C  18 C  16  0.237945 -0.525525  0.002638 -0.185206 (3 -1)
XX  3.944535  0.627062 -1.582472 C  19 C  16  0.232761 -0.502743  0.006812 -0.179005 (3 -1)
XX  4.256958  0.561432  1.388434 C  20 C  15  0.237276 -0.523111  0.004509 -0.184648 (3 -1)
XX  4.411336  0.964297 -0.139307 RING         0.007304  0.032255 -1.282488  0.001659 (3 1)
XX  1.768094  1.465278  1.244440 RING         0.007425  0.029197 -2.718708  0.001744 (3 1)
XX  1.921026  1.846062  1.293786 C  21 C   8  0.007719  0.028944  0.976044  0.001661 (3 -1)
XX  3.153637  0.321933  1.933791 C  21 C  15  0.237792 -0.524810  0.002552 -0.185012 (3 -1)
XX  2.624253 -2.314491  1.050588 C  22 C   1  0.006626  0.022608  0.437620  0.001262 (3 -1)
XX  3.894537 -0.634460  1.608939 C  22 C  15  0.232830 -0.502959  0.006757 -0.179115 (3 -1)
XX  4.384411 -1.002759  0.167872 RING         0.007184  0.031448 -1.282779  0.001615 (3 1)
XX -4.267802  0.574980 -1.369245 C  23 C  13  0.237166 -0.522581  0.004611 -0.184509 (3 -1)
XX -1.775639  1.464151 -1.254037 RING         0.007421  0.029175 -2.737691  0.001742 (3 1)
XX -1.927252  1.841048 -1.302891 C  24 C   7  0.007708  0.028909  0.987681  0.001658 (3 -1)
XX -3.174585  0.321834 -1.928621 C  24 C  13  0.237900 -0.525297  0.002558 -0.185154 (3 -1)
XX -2.657183 -2.325915 -1.055679 C  25 C   4  0.006650  0.022607  0.482114  0.001253 (3 -1)
```

XX -3.923363 -0.625416 -1.593663 C 25 C 13 0.232724 -0.502535 0.006824 -0.178966 (3 -1)
XX -4.250366 -0.582077 1.381771 C 26 C 12 0.237100 -0.522307 0.004582 -0.184419 (3 -1)
XX -4.393366 -0.987954 -0.151088 RING 0.007252 0.031883 -1.282618 0.001638 (3 1)
XX -1.762246 -1.471733 1.249126 RING 0.007425 0.029145 -2.866725 0.001743 (3 1)
XX -1.906022 -1.825426 1.296369 C 27 C 10 0.007662 0.028787 1.090220 0.001658 (3 -1)
XX -3.155007 -0.317873 1.932287 C 27 C 12 0.237888 -0.525247 0.002532 -0.185137 (3 -1)
XX -3.914557 0.622072 1.603098 C 28 C 12 0.232753 -0.502688 0.006848 -0.179000 (3 -1)
XX -4.402378 0.976025 0.165220 RING 0.007255 0.031928 -1.280587 0.001643 (3 1)
XX -5.128396 1.077280 -2.004943 H 29 C 23 0.276096 -0.938542 0.006639 -0.280609 (3 -1)
XX -5.352716 0.874459 -0.949000 H 30 C 23 0.278841 -0.956935 0.008008 -0.285924 (3 -1)
XX -4.662670 1.698205 -1.216143 H 31 C 23 0.278540 -0.961262 0.007729 -0.284962 (3 -1)
XX -3.039669 0.564507 -3.083258 H 32 C 24 0.277202 -0.948296 0.006143 -0.282750 (3 -1)
XX -2.474234 1.193932 -2.378340 H 33 C 24 0.278059 -0.953042 0.008524 -0.284070 (3 -1)
XX -2.166674 0.147203 -2.562002 H 34 C 24 0.276462 -0.938148 0.008240 -0.281251 (3 -1)
XX -4.474547 -1.256908 -2.430778 H 35 C 25 0.275309 -0.932225 0.008031 -0.279155 (3 -1)
XX -3.666056 -1.766203 -1.876082 H 36 C 25 0.278994 -0.962845 0.009440 -0.285976 (3 -1)
XX -4.980049 -1.365613 -0.142774 H 36 C 26 0.009729 0.039313 1.088341 0.002251 (3 -1)
XX -4.637790 -1.575265 -1.386663 H 37 C 25 0.280799 -0.975691 0.009362 -0.289599 (3 -1)
XX -4.464956 1.249335 2.443923 H 38 C 28 0.275320 -0.932325 0.008052 -0.279180 (3 -1)
XX -4.996672 1.347463 0.165111 H 38 H 29 0.009766 0.039467 1.023098 0.002261 (3 -1)
XX -2.657228 2.328373 1.053888 H 39 C 2 0.006498 0.022017 0.490578 0.001222 (3 -1)
XX -3.663209 1.764153 1.884173 H 39 C 28 0.278947 -0.962545 0.009440 -0.285889 (3 -1)
XX -4.636910 1.567212 1.401256 H 40 C 28 0.280861 -0.976173 0.009373 -0.289739 (3 -1)
XX -5.097455 -1.097415 2.025628 H 41 C 26 0.276038 -0.938098 0.006707 -0.280498 (3 -1)
XX -5.533714 -0.008444 0.014373 H 41 H 29 0.009584 0.035059 0.136457 0.001748 (3 -1)
XX -5.338474 -0.890443 0.974307 H 42 C 26 0.278674 -0.955756 0.008142 -0.285572 (3 -1)
XX -4.635325 -1.708103 1.226895 H 43 C 26 0.278598 -0.961726 0.007727 -0.285068 (3 -1)
XX -3.007602 -0.554262 3.086564 H 44 C 27 0.277262 -0.948726 0.006131 -0.282873 (3 -1)
XX -2.444375 -1.183472 2.379872 H 45 C 27 0.278031 -0.952818 0.008580 -0.284021 (3 -1)
XX -2.142365 -0.133814 2.555298 H 46 C 27 0.276430 -0.937856 0.008267 -0.281181 (3 -1)

XX	3.038365	-0.525569	-3.087555	H	47	C	18	0.277248	-0.948693	0.006091	-0.282835	(3 -1)
XX	2.456040	-1.149324	-2.391657	H	48	C	18	0.278012	-0.952470	0.008471	-0.283991	(3 -1)
XX	3.201903	-2.152967	-0.657660	RING				0.003088	0.010059	-2.939540	0.000515	(3 1)
XX	2.173694	-0.093911	-2.563951	H	49	C	18	0.276440	-0.938046	0.008196	-0.281209	(3 -1)
XX	4.507632	1.254615	-2.414059	H	50	C	19	0.275313	-0.932160	0.008125	-0.279162	(3 -1)
XX	2.694420	2.352849	-1.040683	H	51	C	8	0.006561	0.022157	0.542587	0.001218	(3 -1)
XX	3.715667	1.778071	-1.848061	H	51	C	19	0.279064	-0.963786	0.009495	-0.286122	(3 -1)
XX	4.684937	1.554321	-1.367209	H	52	C	19	0.280865	-0.975955	0.009376	-0.289744	(3 -1)
XX	5.012170	1.334659	-0.125165	H	52	C	20	0.009888	0.040049	1.139544	0.002283	(3 -1)
XX	5.110622	-1.101552	-2.005535	H	53	C	17	0.276052	-0.938272	0.006671	-0.280538	(3 -1)
XX	5.337057	-0.910708	-0.947796	H	54	C	17	0.278728	-0.956119	0.008075	-0.285701	(3 -1)
XX	4.628322	-1.716410	-1.221572	H	55	C	17	0.278640	-0.962189	0.007720	-0.285165	(3 -1)
XX	5.110667	1.059153	2.036841	H	56	C	20	0.276089	-0.938415	0.006647	-0.280586	(3 -1)
XX	5.533767	-0.037253	0.022672	H	56	H	53	0.009526	0.034855	0.137612	0.001745	(3 -1)
XX	5.354223	0.844859	0.987662	H	57	C	20	0.278687	-0.955865	0.008087	-0.285591	(3 -1)
XX	4.667546	1.678284	1.234149	H	58	C	20	0.278583	-0.961465	0.007685	-0.285041	(3 -1)
XX	4.432378	-1.271384	2.450572	H	59	C	22	0.275346	-0.932591	0.007897	-0.279237	(3 -1)
XX	4.963265	-1.380145	0.168500	H	59	H	53	0.009558	0.038525	1.008316	0.002218	(3 -1)
XX	3.618025	-1.769511	1.894542	H	60	C	22	0.278934	-0.962127	0.009333	-0.285860	(3 -1)
XX	4.594438	-1.596382	1.408047	H	61	C	22	0.280794	-0.975857	0.009269	-0.289595	(3 -1)
XX	3.006771	0.566587	3.086601	H	62	C	21	0.277205	-0.948286	0.006185	-0.282758	(3 -1)
XX	2.457445	1.202561	2.374747	H	63	C	21	0.278059	-0.953173	0.008577	-0.284070	(3 -1)
XX	2.135754	0.159465	2.554380	H	64	C	21	0.276433	-0.937804	0.008278	-0.281183	(3 -1)
XX	1.573436	2.815249	0.001946	N	65	C	8	0.463980	0.273383	0.032553	-0.828896	(3 -1)
XX	-1.572000	2.816299	-0.016812	N	66	C	7	0.463984	0.273791	0.032534	-0.828892	(3 -1)
XX	1.571099	-2.812499	-0.037995	N	67	C	9	0.463989	0.274140	0.032576	-0.828892	(3 -1)
XX	-1.571881	-2.811759	0.005567	N	68	C	10	0.463982	0.273621	0.032603	-0.828895	(3 -1)

References

(1) Kraka, E.; Cremer, D. Chemical Implication of Local Features of the Electron Density Distribution. In *Theoretical Models of Chemical Bonding. The concept of the Chemical Bond.*; Maksic, Z. B., Ed.; Springer-Verlag, 1990; Vol. 2.
(2) Bone, R. G. A.; Bader, R. F. W. Identifying and Analyzing Intermolecular Bonding Interactions in van Der Waals Molecules. *J Phys Chem* **1996**, *100* (26), 10892–10911. https://doi.org/10.1021/jp953512m.
(3) P. L. A. Popelier. Characterization of a Dihydrogen Bond on the Basis of the Electron Density. *J Phys Chem A* **1998**, *102*, 1873–1878.
